# Enzyme‐Catalysed Formation of Hydrocarbon Scaffolds from Geranylgeranyl Diphosphate Analogs with Shifted Double Bonds

**DOI:** 10.1002/chem.202500712

**Published:** 2025-03-21

**Authors:** Heng Li, Bernd Goldfuss, Jeroen S. Dickschat

**Affiliations:** ^1^ Kekulé-Institute for Organic Chemistry and Biochemistry University of Bonn Gerhard-Domagk-Straße 1 53121 Bonn Germany; ^2^ Department for Chemistry University of Cologne Greinstraße 4 50939 Cologne Germany

**Keywords:** biosynthesis, enzyme mechanisms, isotopes, substrate analogs, terpenes

## Abstract

Four analogs of geranylgeranyl diphosphate (GGPP) with shifted double bonds were synthesised and enzymatically converted with 14 diterpene synthases of previously reported function, including two newly characterised homologs of the benditerpe‐2,6,15‐triene synthase Bnd4 and the venezuelaene synthase VenA. In successful cases the products were isolated and structurally characterised by NMR spectroscopy, revealing the formation of various diterpenoids with skeletons that have not been reported from natural sources. Isotopic labelling experiments in conjunction with DFT calculations were performed to give insights into hydride migrations in the biosynthesis of the non‐natural diterpenes benditerpe‐2,7(19),15‐triene and venezuelaxenene and their natural counterparts from GGPP.

## Graphical abstract

Four new geranylgeranyl diphosphate analogs with shifted double bonds were synthesized and together with the known analog *iso*‐GGPP I converted with 14 diterpene synthases. While two substrate analogs were not accepted by the enzymes, the other three analogs yielded compounds with skeletons that are not observed in nature. Mechanistic key steps were investigated through isotopic labelling experiments and computational chemistry.

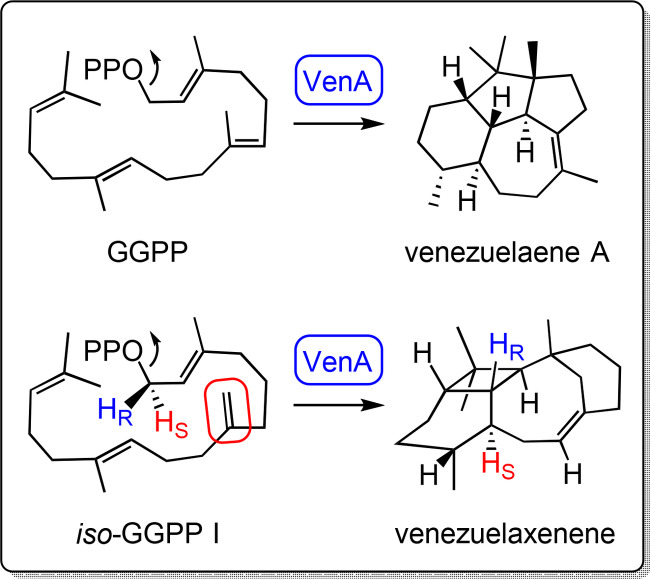

## Introduction

Terpene synthases are remarkable biocatalysts that can turn structurally simple substrates such as geranyl diphosphate (GPP), farnesyl diphosphate (FPP), geranylgeranyl diphosphate (GGPP) and even longer oligoprenyl diphosphates into a large diversity of terpene hydrocarbons or alcohols.[^1^, ^2^] The products of these reactions frequently contain multiple rings and stereogenic centres. During terpene cyclisation usually more than half of the carbons of the substrate undergo a change in the hybridisation and bonding state,^[3]^ with several carbon‐carbon bonds being formed. Overall, terpene cyclisations belong to the most complex transformations in nature catalysed by a single enzyme.

Recent research has demonstrated that terpene synthases cannot only act on their natural substrates, but many structural modifications realised in synthetic substrate analogs are tolerated. This includes hydroxylated oligoprenyl diphosphates,[^4^, ^5^] epoxides,^[6]^ compounds with heteroatoms inserted into the chain,[^7^, ^8^] ketones[^9^, ^10^] and halogenated[^11^, ^12^, ^13^, ^14^] and methoxy‐substituted^[15]^ compounds. Also the double bond functions have been manipulated, represented by partially saturated substrate analogs[^16^, ^17^, ^18^, ^19^] or derivatives with a shifted double bond.^[20]^ In addition, compounds with a changed alkylation pattern[^21^, ^22^] and cyclopropyl derivatives^[23]^ have been employed.

In an alternative approach, Tiefenbacher has used supramolecular capsules to convert analogs of terpene precursors.^[24]^ This method can also start from terpenoid acetate esters^[25]^ of even alcohols^[26]^ instead of diphosphate esters required by enzymes.

**Scheme 1:**
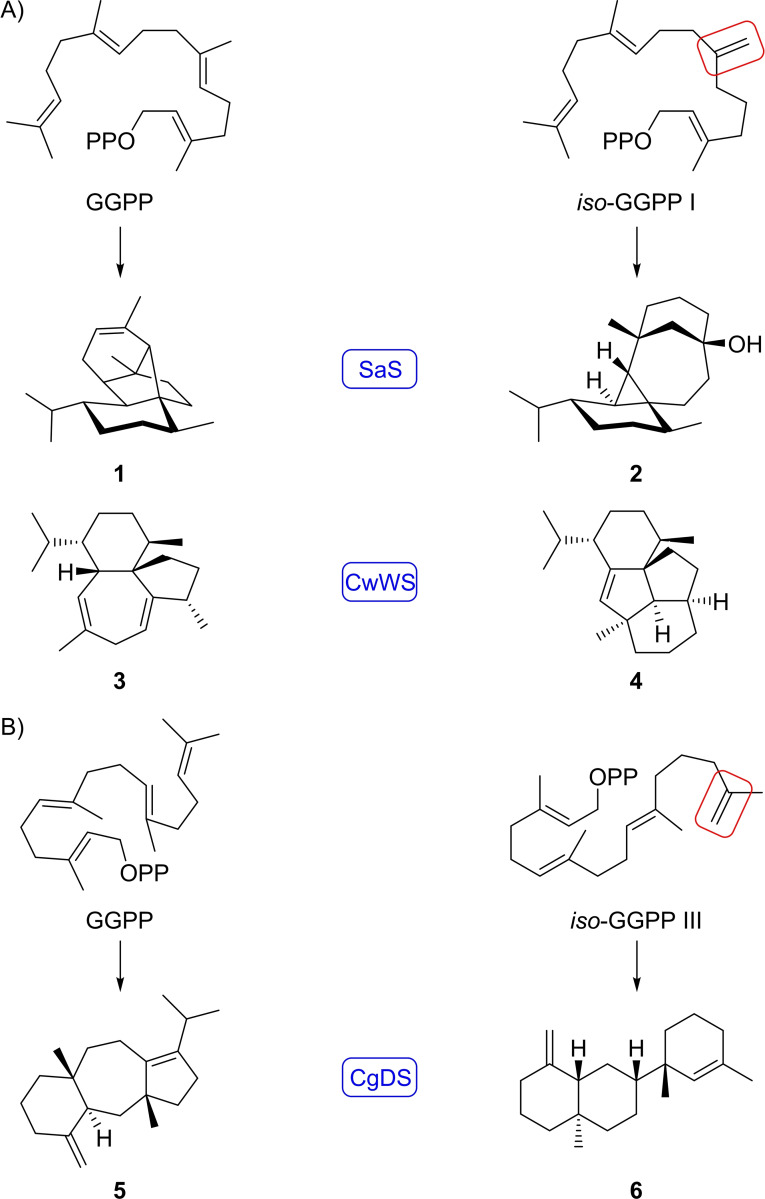
Diterpene hydrocarbons previously obtained from GGPP and its analogs with shifted double bonds. A) Diterpene hydrocarbons obtained from GGPP (left) and from *iso*‐GGPP I (right). B) Diterpene hydrocarbons obtained from GGPP (left) and *iso*‐GGPP III (right). The blue boxes indicate the enzymes used and the red boxes highlight the shifted double bonds in the substrate analogs.

We have recently demonstrated that substrate analogs with shifted double bonds can because of their changed reactivity result in very interesting compounds with novel skeletons, e. g. *iso*‐GGPP I, a GGPP isomer with a shifted C6=C7 double bond, can yield several unusual products with various type I terpene synthases. For instance, spiroalbatene synthase from *Allokutzneria albata* (SaS) generates spiroalbatene (**1**) from natural GGPP,^[27]^ but albataxenene (**2**) from *iso*‐GGPP I,^[28]^ or wanjudiene synthase from *Chryseobacterium wanjuense* (CwWS) converts GGPP into wanjudiene (**3**),^[29]^ but *iso*‐GGPP I into wanjuxenene^[28]^ (“xenene” after gr. ξϵνοσ=foreighn indicates the unusual carbon skeletons that cannot be formed from GGPP, Scheme 1). Furthermore, the dolasta‐1(15),8‐diene synthase from *Colletotrichum gloeosporioides* (CgDS) catalyses the formation of dolasta‐1(15),8‐diene (**5**) from GGPP,^[30]^ while *iso*‐GGPP III with a shifted C14=C15 double bond leads to dolastaxenene (**6**).^[31]^ Here we report on the synthesis of four more GGPP derivatives with two or even three shifted double bonds and on several new diterpenoids obtained enzymatically from these substrate analogs.

## Results and Discussion

**Scheme 2:**
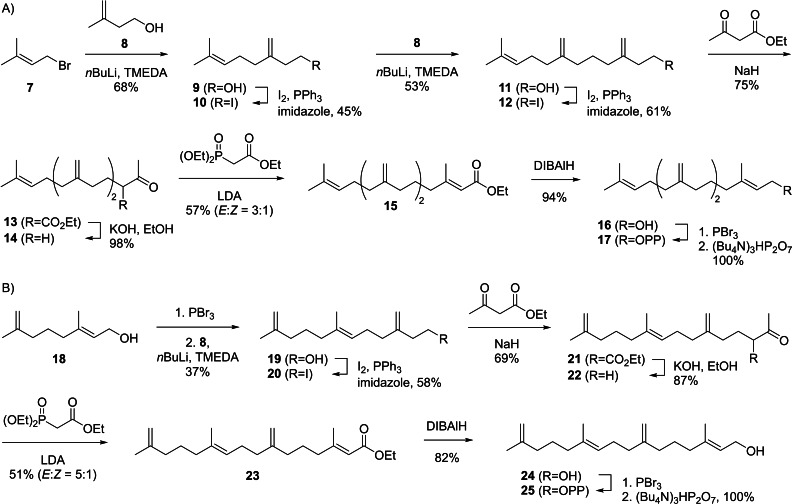
Synthesis of *iso*‐GGPP IV (**17**) and *iso*‐GGPP V (**25**).

The synthesis of *iso*‐GGPP IV (**17**, Scheme 2A) started from prenyl bromide (**7**) that underwent nucleophilic substitution to **9** with the dianion of isoprenol generated with two equivalents of BuLi in the presence of TMEDA.^[32]^ Conversion into the iodide **10** with iodine, PPh_3_ and imidazole was followed by another coupling with **8** to yield **11**. After transformation into the iodide **12** a nucleophilic substitution with the enolate anion of ethyl acetoacetate resulted in **13** that was saponified with spontaneous decarboxylation of the β‐keto acid to **14**. A Horner‐Wadsworth‐Emmons (HWE) reaction with triethyl phosphonoacetate gave access to **15** that was reduced with DIBAlH to **16** and subsequently converted into the target compound **17** through bromination and phosphorylation. The diphosphate **17** was obtained with a yield of 6 % over ten sequential steps.

Using a similar strategy, *iso*‐GGPP V (**25**, Scheme 2B) was prepared from known alcohol **18**.^[31]^ Bromination and elongation with **8** furnished alcohol **19** that was converted into the corresponding iodide **20**. This material was used to alkylate ethyl acetoactetat to yield ester **21** that was saponified with decarboxylation to **22**. A HWE reaction to **23**, DIBAlH reduction to **24**, bromination and phosphorylation completed the synthesis of **25** with an overall yield of 5 % through nine linear steps.

**Scheme 3:**
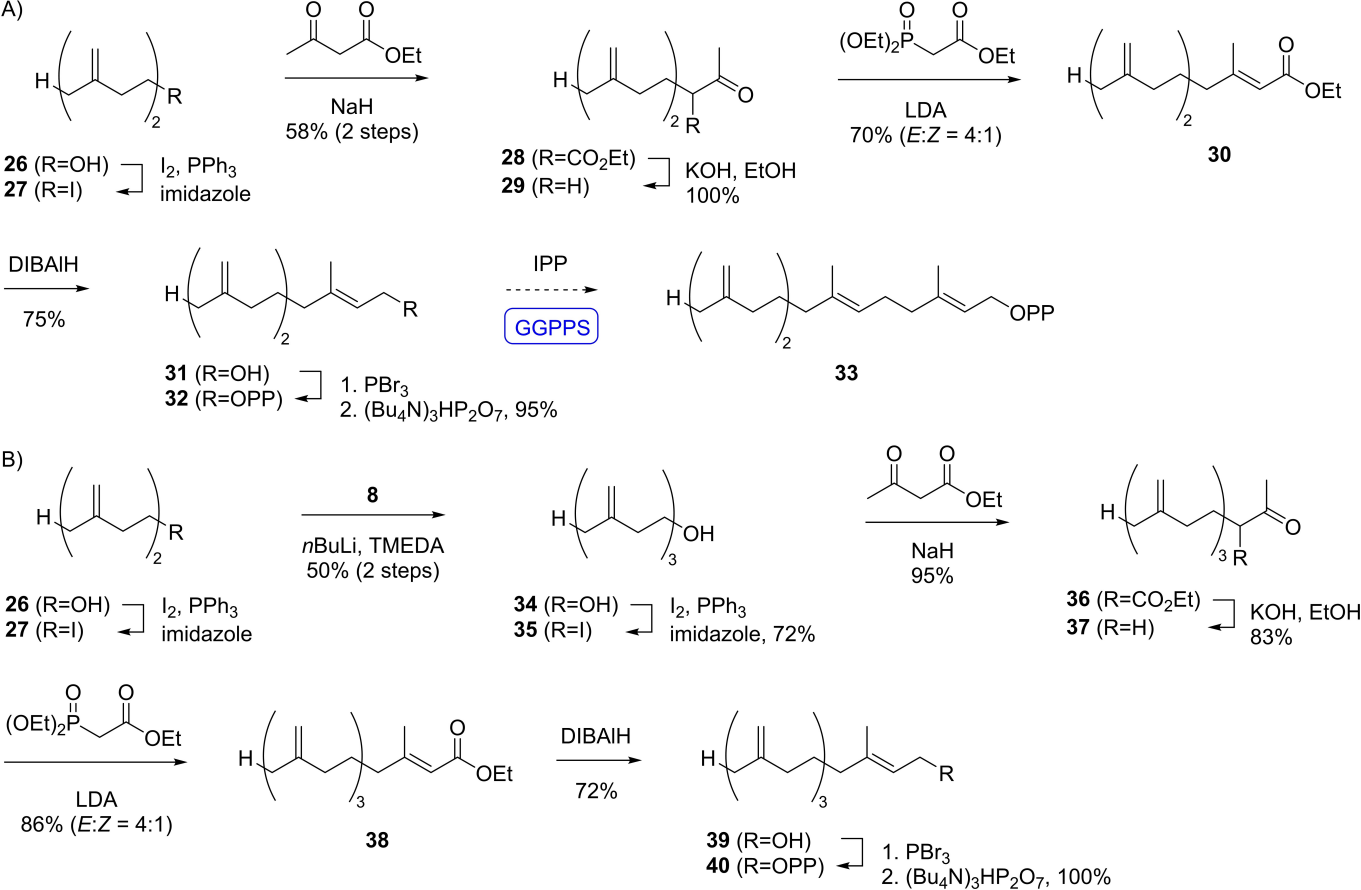
Synthesis of *iso*‐FPP III (**32**) and *iso*‐GGPP VII (**40**).

The third target compound *iso*‐GGPP VI (**33**, Scheme 3A) was made accessible in a combined synthetic and enzymatic approach. The C_15_ compound *iso*‐FPP III (**32**) was obtained from known alcohol **26**[^32^, ^33^] through conversion into the iodide **27** for the alkylation of ethyl acetoacetate to β‐ketoester **28**, followed by saponification and decarboxylation to **29**. Subsequent standard transformations furnished the diphosphate **32** that can be elongated with isopentenyl diphosphate (IPP) to **33** using the GGPP synthase (GGPPS) from *Streptomyces cyaneofuscatus*.^[34]^ The substrate analog **32** was obtained over seven sequential steps with a yield of 23 %.

The fourth compound *iso*‐GGPP VII (**40**, Scheme 3B) was likewise prepared from known alcohol **26**.[^32^, ^33^] After conversion into the iodide **27** and elongation with **8** to **34** through the dianion method another iodination to **35** allowed for the alkylation of ethyl acetoactetate to β‐ketoester **36**. Saponification and decarboxylation to **37**, HWE elongation to **38**, reduction and phosphorylation gave access to **40**. The material was made available with a yield of 14 % over nine transformations.

All four synthetic substrate analogs were screened for their conversion by various diterpene synthases (Table S1). While for *iso*‐GGPP V and *iso*‐GGPP VII no successful cases were observed with any of the tested diterpene synthases, *iso*‐GGPP IV and *iso*‐GGPP VI were accepted by several enzymes. Furthermore, the previously reported substrate analog *iso*‐GGPP I^[20]^ was converted by two newly characterised bacterial diterpene synthases. This included the benditerpe‐2,6,15‐triene synthase Bnd4 from *Streptomyces iakyrus* NRRL ISP‐5482 (accession number WP 033312626), which is a homolog of the previously reported Bnd4 from *Streptomyces* sp. CL12‐4,^[35]^ exhibiting a pairwise amino acid sequence identity of 92 %. The incubation of GGPP with the purified enzyme from *S. iakyrus* (Figure S1) showed the formation of one major thermally instable compound by GC/MS (Figure S2) that was isolated and structurally characterised by NMR spectroscopy as benditerpe‐2,6,15‐triene (**41**),^[35]^ a compound that can undergo a Cope rearrangement explaining its behaviour under the thermal impact during GC/MS analysis.

**Scheme 4:**
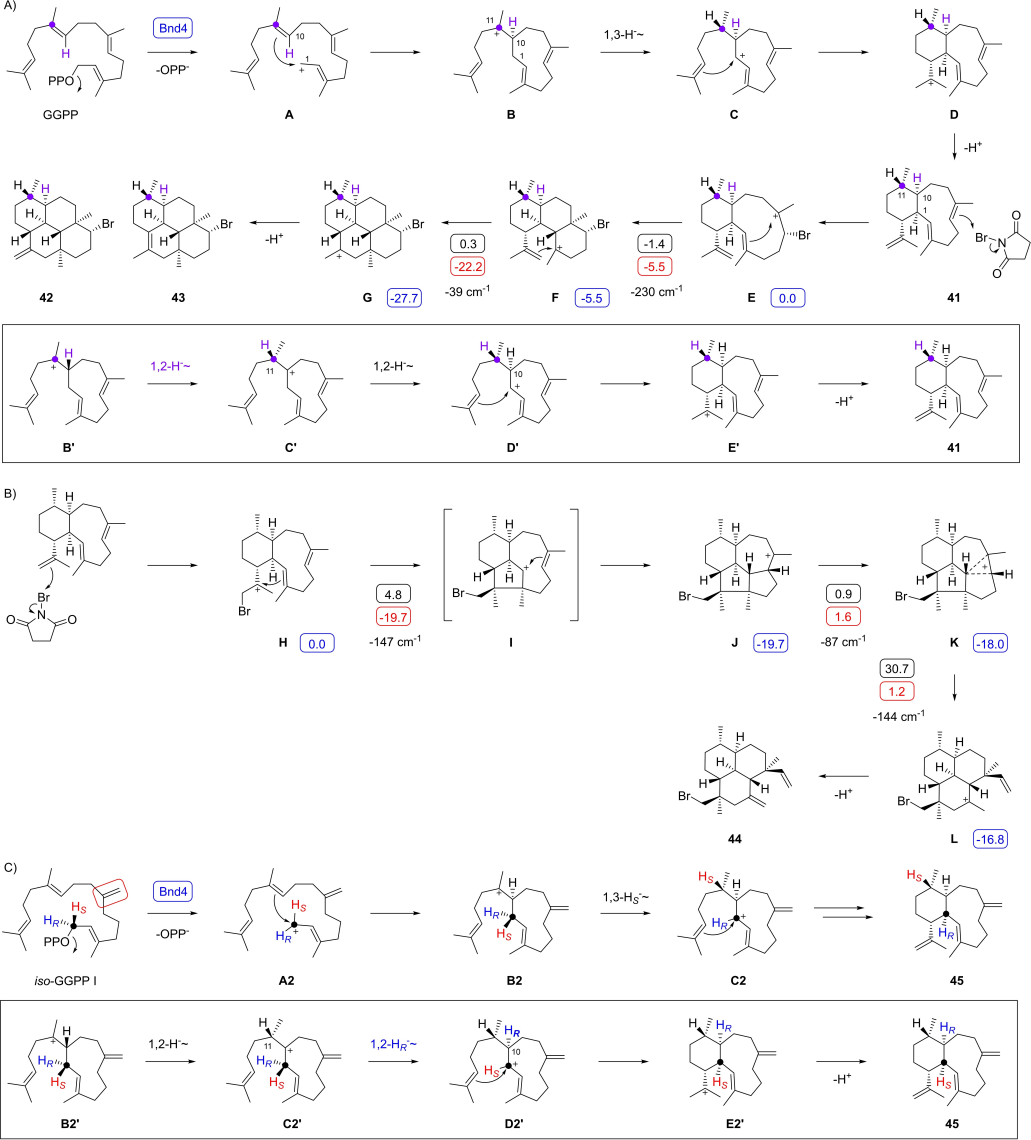
The diterpene synthase Bnd4 from *S. iakyrus*. A) Cyclisation mechanism for the cyclisation of GGPP to benditerpe‐2,6,15‐triene (**41**) with a 1,3‐hydride shift and formation of bromination products **42** and **43** with NBS. Box: Hypothetical sequence with two 1,2‐hydride shifts that is disfavoured by labelling experiments. B) Formation of bromination product **44**. Blue boxes indicate computed relative energies to **E** or **H** (set to 0.0 kcal/mol), black boxes indicate reaction barriers, and red boxes show Gibbs free energies (computed with the mPW1PW91/6‐311+G(d,p)//B97D3/6‐31G(d,p) method at 298 K). Imaginary frequencies of computationally localised transition state structures are given in cm^−1^ for each step. C) Cyclisation mechanism from *iso*‐GGPP I to **45**. Box: Hypothetical sequence with two 1,2‐hydride shifts. Purple and black dots indicate ^13^C‐labelled carbons.

The biosynthesis of **41** has been demonstrated to proceed through a 1,3‐hydride shift (Scheme 4A) through incubation of (1,1‐^2^H_2_)GGPP with Bnd4 from *Streptomyces* sp. CL12‐4, product isolation and NMR spectroscopy, and by DFT computations.^[36]^ A simpler experiment makes use of a double labelling strategy with deuterium labelling at C1 and a ^13^C‐labelling at the target position of the migrating deuterium (C11), using the substrate (11‐^13^C,1,1‐^2^H_2_)GGPP that can be prepared in situ from (7‐^13^C)FPP and (1,1‐^2^H_2_)IPP using GGPP synthase (GGPPS). Furthermore, it has not been investigated experimentally which of the two enantiotopic hydrogens at C1 of GGPP is migrating, which can be investigated using stereoselectively deuterated substrates. In the present case the experiment with (7‐^13^C)FPP^[37]^ and (1,1‐^2^H_2_)IPP^[38]^ was unsuccessful for two reasons: First, the signal for the deuterated carbon (C11) is expected to split into a triplet and will have a lower intensity compared to the signal of a non‐deuterated carbon as a result of the nuclear quadrupole moment that causes prolonged spin relaxation times. Second, the special molecular mechanics of **41** cause a line broadening in the ^13^C‐NMR even at elevated temperature (343 K). Both effects together made it impossible to detect the triplet signal to confirm the 1,3‐hydride shift. To overcome the second problem of line broadening, we tried to rigidify the skeleton of **41** through a derivatisation with N‐bromosuccinimide (NBS) that resulted in the formation of three compounds (**42**–**44**, Schemes 4A and 4B, Tables S2 – S4, Figures S3 – S25). While the major products **42** and **43** were expected, the minor formation of **44** was surprising.

Quantum chemical calculations (Table S5, Figure S26) showed two barrierless cyclisations from **E** via **F** to **G** towards **42** and **43**, while **44** was more difficult to explain. Its formation can be understood from the bromonium adduct **H** that can undergo two asynchronous concerted cyclisations to **J** through a low barrier. An almost barrierless rearrangement to **K** opens the path to a ring‐opening with simultaneous rearrangement to **L** that can undergo deprotonation to yield **44**. The high activation barrier for the **K**‐to‐**L** reaction of 30.7 kcal/mol explains the minor formation of **44**.

The first problem of the lowered signal intensity of a deuterated carbon was addressed by a change of strategy. The substrate combination of (7‐^13^C,6‐^2^H)FPP and IPP was converted with *Streptomyces cyaneofuscatus* GGPPS^[34]^ and Bnd4, followed by NBS treatment to obtain labelled **42** (labelling experiments are summarised in Table S6). If the 1,3‐hydride shift from **B** to **C** takes place in the biosynthesis of **41**, a proton will migrate from C1 to C11, while deuterium will stay at C10 and slightly influence the chemical shift of the neigbouring labelled carbon C11. The alternative of two sequential 1,2‐hydride shifts (box in Scheme 4A) would require intermediate **B’**, the enantiomer of **B**, to explain the correct configuration at C11 in **C’**, and would result in a triplet for C11 of **42**. In the experiment an upfiled shifted singlet for C11 of **42** was observed (Δδ=−0.06 ppm) revealing a deuterium effect from the neighboring position (Figure S27).

Bnd4 from *S. iakyrus* was also incubated with the substrate analog *iso*‐GGPP I,^[20]^ resulting in its conversion into one major diterpene hydrocarbon (Figure S28). This compound was isolated and structurally characterised by NMR spectroscopy as benditerpe‐2,7(19),15‐triene (**45**) (Table S7, Figures S29–S36). Its biosynthesis may proceed through ionisation of *iso*‐GGPP I to **A**, followed by a 1,10‐cyclisation to **B**, a 1,3‐hydride shift to **C**, 1,14‐cyclisation to **D** and deprotonation (Scheme 4C), closely resembling the cyclisation mechanism from GGPP to benditerpe‐2,6,15‐triene (Scheme 4A). Notably, the shifted double bond in **45** disrupts the Cope system and consequently this compound is – in contrast to the natural product **41** – thermally stable. The stereochemical course of the 1,3‐hydride shift in the biosynthesis of **45** was investigated throught the incubation of (*R*)‐ and (*S*)‐(1‐^13^C,1‐^2^H)*iso*‐GGPP I^[28]^ with Bnd4, indicating retainment of the 1‐*pro*‐*R* hydrogen at C1 (Δδ=−0.51 ppm, ^1^ *J*_C,D_=18.8 Hz) and migration of the 1‐*pro*‐*S*, that resulted in an upfield shift of Δδ=−0.08 ppm (Figure S37). This upfield shift is larger than usually observed for deuterium located two positions away (Δδ ≈−0.01 ppm), but similar to the expected upfield shift for deuterium located at a directly neighbouring carbon (Δδ ≈ –0.10 ppm), questioning whether deuterium was located at C11 as a result of a direct 1,3‐hydride shift or at C10 through two sequential 1,2‐hydride shifts. However, two sequential hydride shifts would not only proceed through **B2’**, the enantiomer of **B2**, but also in the step from **C2’** to **D2’** the 1‐*pro*‐*R* hydrogen would have to migrate to set the correct stereochemistry at C10, which is not experimentally observed. The 1‐*pro*‐*R* and 1‐*pro*‐*S* hydrogens could swap their roles, if the absolute configuration of **45** and consequently also of **41** would be inverted, but stereoselective deuteration experiments with DMAPP and (*E*)‐ or (*Z*)‐(4‐^13^C,4‐^2^H)IPP,^[39]^ GGPPS and Bnd4, followed by conversion of the product with NBS into **42** (Figure S38) confirmed the published absolute configuration of **41**.^[35]^ These experiments make use of the introduction of artificial stereogenic centres at the deuterated carbons of known configuration, simplifying the problem of absolute configuration determination to one of solving the relative configuration of the naturally present stereogenic centres with respect to the artificially introduced anchors. The additional ^13^C‐labels at the deuterated carbons allow for a highly sensitive detection of deuterium incorporation through HSQC spectroscopy. These experiments require a detailed knowledge about the stereochemical course of the GGPP biosynthesis from the terpene monomers.^[40]^

**Scheme 5:**
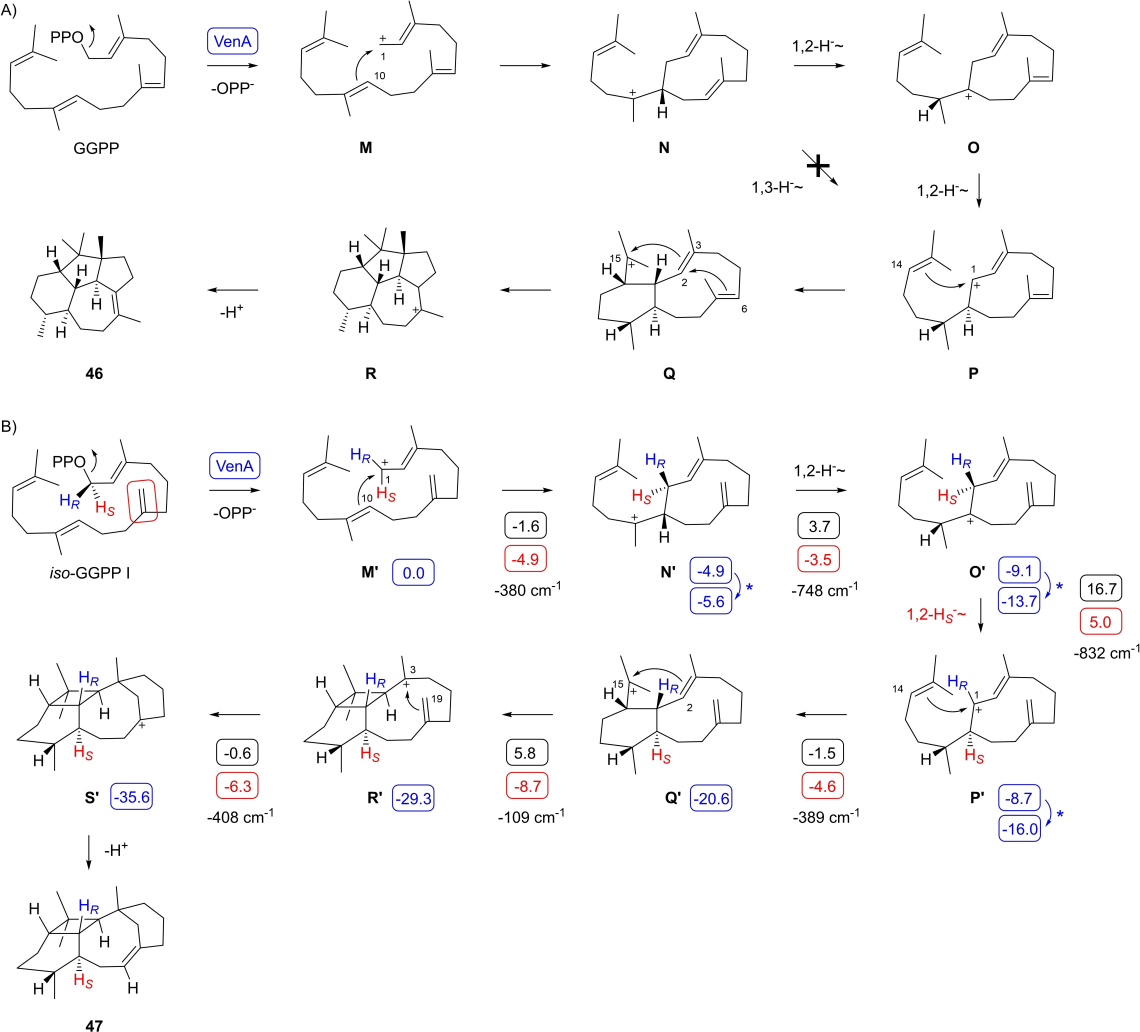
The diterpene synthase VenA from *S. exfoliatus*. A) Cyclisation mechanism from GGPP to **46**. Cyclisation mechanism from *iso*‐GGPP I to **47**. Blue boxes indicate computed relative energies to **M’** (set to 0.0 kcal/mol), black boxes indicate reaction barriers, and red boxes show Gibbs free energies (computed with the mPW1PW91/6‐311+G(d,p)//B97D3/6‐31G(d,p) method at 298 K). Imaginary frequencies of computationally localised transition state structures are given in cm^−1^ for each step.

The second investigated diterpene synthase is a VenA homolog from *Streptomyces exfoliatus* DSM 41693, exhibiting an amino sequence identity of 97 % to the previously characterised enzyme from *Streptomyces venezuelae* ATCC 15439.^[41]^ Incubation of the purified enzyme (Figure S1) with GGPP resulted in the formation of one major diterpene hydrocarbon (Figure S39) that was isolated and its structure identified as venezuelaene A (**46**).^[41]^ The proposed biosynthesis of **46** starts with substrate ionisation to **M** and 1,10‐cyclisation to **N** (Scheme 5A). Next, a 1,3‐hydride shift to **P** has been suggested,^[41]^ but this step has not been experimentally verified and may also be substituted by two sequential 1,2‐hydride shifts through **O**. A subsequent 1,14‐cyclisation to **P** followed by a 3,15‐ and 2,6‐cyclisation then lead to **R** as the direct precursor of **46** by deprotonation.

To distinguish between the possible 1,3‐ or the two sequential 1,2‐hydride shifts in the biosynthesis of **46** isotopic labelling experiments were performed. The incubation of (7‐^13^C,6‐^2^H)FPP and IPP with GGPPS and VenA resulted in an upfield shifted triplet for C11 (Δδ=−0.52 ppm, ^1^ *J*_C,D_=19.4 Hz), which supports the sequence of two 1,2‐hydride migrations and disfavours the single 1,3‐hydride transfer (Figure S40). Furthermore, the conversion of (3‐^13^C)GPP^[42]^ and (1,1‐^2^H_2_)IPP^[38]^ with GGPPS and VenA resulted in a slightly upfield shifted singlet for C11 of **46** (Δδ=−0.12 ppm), indicating deuterium in a neighbouring position and thus pointing into the same direction (Figure S41).

The conversion of *iso*‐GGPP I with VenA also yielded one major diterpene hydrocarbon (Figure S42) that was isolated and identified as venezuelaxenene (**47**) (Table S8, Figures S43–50). In this case the skeleton of **47** is very different to that of **46**, which is a result of the C7=C19 double bond in *iso*‐GGPP I that participates in the cyclisation reaction. After substrate ionisation to **M’** a 1,10‐cyclisation to **N’** and two sequential 1,2‐hydride shifts lead via **O’** to **P’** (Scheme 5B). A subsequent 1,14‐cyclisation to **Q’** and another 2,15‐cyclisation result in **R’** in which C19 is close enough to the cationic centre to initiate a 3,19‐cyclisation to **S’**. A terminal deprotonation results in **47**. While the initial steps towards **P’** are the same as those towards **P** in the biosynthesis of **46** (Scheme 5A), the late stage transformations deviate and yield the unusual skeleton of **47**.

For **47** the same sequence of two 1,2‐hydride shifts as experimentally verified for **46** can be assumed. To investigate the stereochemical course of this process, (*R*)‐ and (*S*)‐(1‐^13^C,1‐^2^H)*iso*‐GGPP I were incubated with VenA. The observed upfield shifted singlet peak for C1 with the substrate (*S*)‐(1‐^13^C,1‐^2^H)*iso*‐GGPP I (Δδ=–0.10 ppm) illustrated the hydride migration of the 1‐*pro*‐*S* hydrogen into the neighbouring position C10, while the upfield shifted triplet obtained from (*R*)‐(1‐^13^C,1‐^2^H)*iso*‐GGPP I (Δδ=–0.54 ppm, ^1^ *J*_C,D_=20.1 Hz) demonstrated retainment of the 1‐*pro*‐*R* hydrogen at C1 (Figure S51).

The cyclisation mechanims of *iso*‐GGPP I to **47** was also investigated computationally (Scheme 5B, Table S9, Figure S52). After a smooth cyclisation from **M’** to **N’** the DFT calculations in particular confirmed the sequence of two 1,2‐hydride shifts to **P’**, while a single 1,3‐hydride transfer could not be realised. The second 1,2‐hydride shift proceeds with migration of the 1‐*pro*‐*S* hydrogen and is with an activation barrier of 16.7 kcal/mol the rate limiting step. All subsequent cyclisation steps towards **S’** exhibit low activation barriers or are even barrierless, and the whole cascade is with ΔG=–35.6 kcal/mol strongly exergonic.

**Scheme 6:**
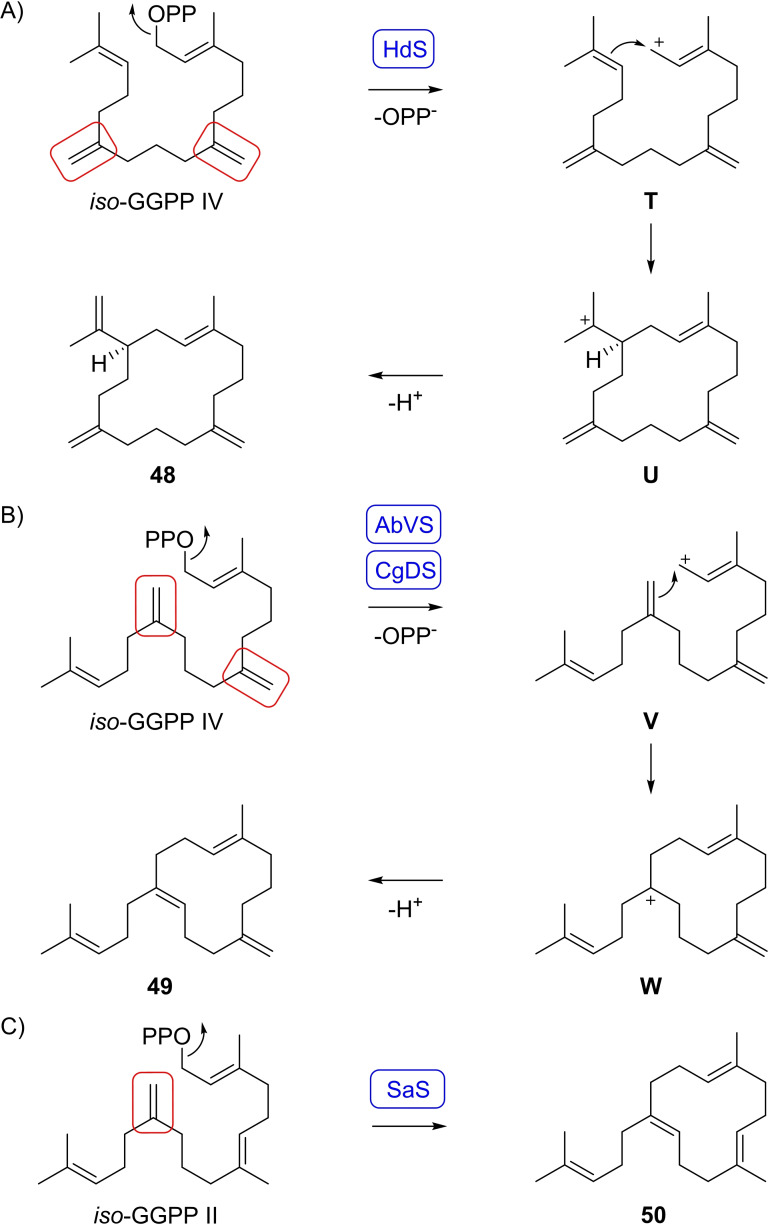
Cyclisation mechanisms from *iso*‐GGPP IV to A) **48** by HdS and to B) **49** by AbVS and CgDS, and C) of *iso*‐GGPP II to **50** by SaS.

The substrate analog *iso*‐GGPP IV was efficiently converted by three of the tested enzymes (Table S1). The 18‐hydroxydolabella‐3,7‐diene synthase from *Chitinophaga pinensis* (HdS)^[43]^ converted *iso*‐GGPP IV into one main product (Figure S53). Isolation and NMR‐based structure elucidation revealed the structure of a macrocyclic diterpene hydrocarbon (**48**) (Table S10, Figures S54–S61). Because of the structural similarity to cembrene A with two shifted double bonds this compound was named diisocembrene A (Scheme 6A). Its formation requires substrate ionisation to **T**, cyclisation to **U** and deprotonation. Also the variediene synthase from *Aspergillus brasiliensis* (AbVS) and the dolasta‐1(15),8‐diene synthase from *C. gloeosporioides* (CgDS)^[30]^ converted *iso*‐GGPP IV into one major diterpene hydrocarbon (Figure S62). Compound isolation and structure elucidation by NMR spectroscopy (Table S11, Figures S63–S70) revealed the structure of prenylisopseudogermacrene B (**49**). Its formation proceeds through ionisation of *iso*‐GGPP IV to **V**, followed by a 1,18‐cyclisation to **W** and deprotonation (Scheme 6B). Compound **49** is an isomer with a shifted double bond of prenylpseudogermacrene B (**50**) that was previously obtained from *iso*‐GGPP II^[28]^ using the spiroalbatene synthase from *A. albata* ^[27]^ (Scheme 6C).

The substrate analog *iso*‐GGPP VI, enzymatically prepared in situ from *iso*‐FPP III and IPP with GGPPS, was accepted by many different diterpene synthases, in some cases with conversion into one major product, but in other cases complex product mixtures were obtained (Scheme 7, Table S1, Figures S71–S73). In total, seven different products were isolated from enzymatic reactions in which the compounds were formed as major products. The structures of all seven isolated compounds were determined by NMR spectroscopy (Tables S12–S18, Figures S74–S129). Isopentenylpseudogermacrene A (**51**) was isolated from an incubation of *iso*‐FPP III and IPP with GGPPS and spinodiene synthase from *Saccharopolyspora spinosa* (SoS).^[44]^ The same compound is also formed by the phomopsene/allokutznere synthase from *A. albata* (PmS),^[39]^ the catenul‐14‐en‐6‐ol synthase from *Catenulispora acidiphila* (CaCS),^[17]^ and (in traces) by CgDS (Scheme 7, Figure S71).

**Scheme 7:**
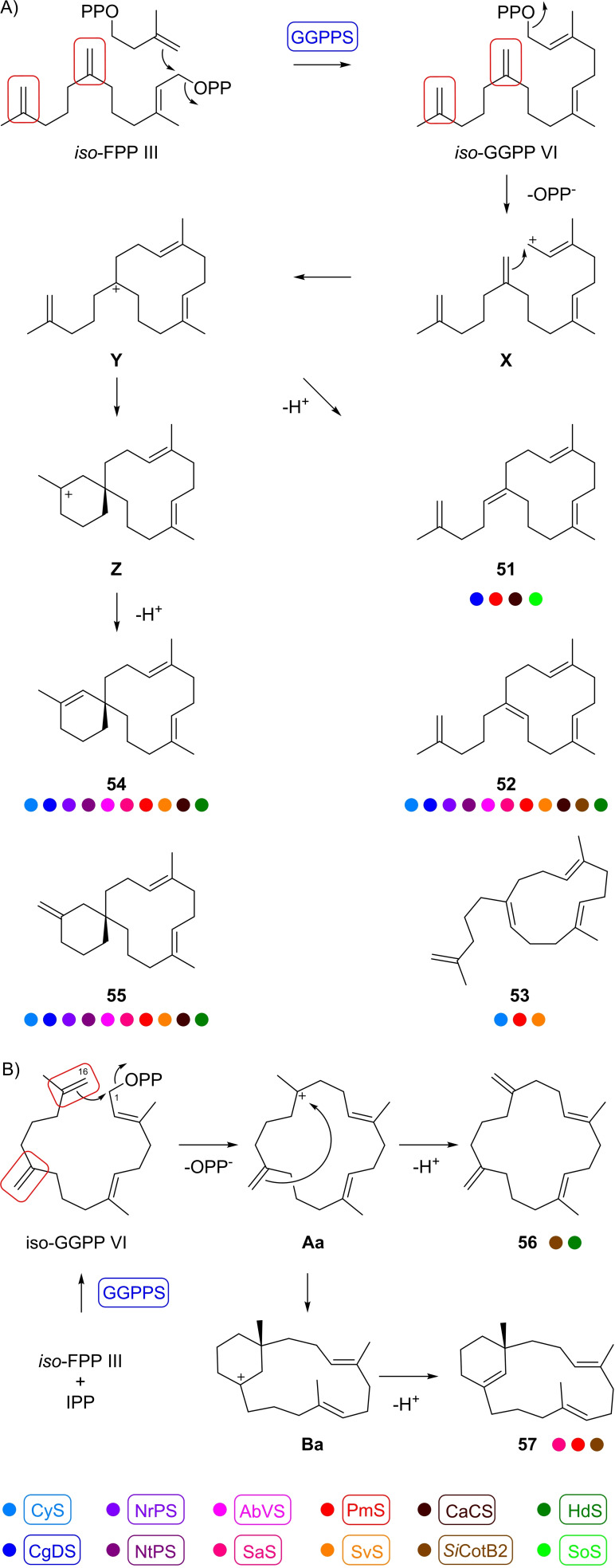
A) Cyclisation mechanism from *iso*‐GGPP VI to **51–55** by various diterpene synthases. B) Cyclisation mechanism from *iso*‐GGPP VI to **56** and **57** by *Si*CotB2. Coulored dots indicate compound production by enzymes according to the colour code at the bottom of the scheme.

This compound is formed from *iso*‐GGPP VI by substrate ionisation to **X**, 1,18‐cyclisation to **Y** and deprotonation. Its regioisomer isopentenylpseudogermacrene B (**52**) is produced by all investigated enzymes but SoS, albeit in different quantities, and arises from **Y** by alternative deprotonation. This compound was isolated from the cattleyene synthase from *Streptomyces cattleya* (CyS)^[45]^ that converts *iso*‐GGPP VI into **52** almost as a single product. Isopentenylpseudogermacrene C (**53**) also arises from **Y** and is a stereoisomer of **52**. This compound is only formed by CyS, PmS and the spiroviolene synthase from *Streptomyces violens*.^[34]^ SvS is the best producing enzyme of **53** and thus this enzyme was used for a preparative scale incubation for its isolation. Two more compounds coeluted in the GC/MS analysis, but were separable by column chromatography and identified as the regioisomers α‐spirocattleyaxenene (**54**), isolated as a minor product from CyS, and β‐spirocattleyaxenene (**55**) that was isolated from PmS in which larger amounts are produced. Their formation can be explained through a second cyclisation from **Y** to **Z** and deprotonation. Compounds **54** and **55** show similar mass spectra and coelute in the GC/MS analysis, so that it is impossible to decide based on GC/MS data which of these compounds is formed by which enzyme. Therefore, isotopic labelling experiments were performed, introducing a ^13^C label at C1 with the substrate combination of *iso*‐FPP III and (1‐^13^C)IPP, that were converted with GGPPS and the different diterpene synthases producing **54** and/or **55**. ^13^C‐NMR analysis of the product mixture allowed to distinguish the production of these compounds by each enzyme (Figure S130). Mixtures of **54** and **55** are formed by almost all investigated enzymes, albeit in different proportions, with the exceptions of SoS and cyclooctat‐9‐en‐7‐ol synthase from *S. iakyrus* (*Si*CotB2),^[46]^ a recently reported homolog of CotB2 from *Streptomyces melanosporofaciens* (*Sm*CotB2).^[47]^ Instead, *Si*CotB2 produced two other interesting compounds, namely isobucketwheelene (**56**) and iakyroxenene (**57**). Their biosynthesis can be explained by a 1,16‐cyclisation to **Aa** and deprotonation (to **56**), while another 15,18‐cyclisation from **Aa** to **Ba** and deprotonation result in **57**. Compound **56** is a double bond isomer of the previously reported bucketwheelene;^[31]^ both compounds were named because of their molecular shape that is reminiscent of a bucket wheel. The absolute configurations of **55** and **57** was determined through stereoselective deuteration experiments using *iso*‐FPP III and (*R*)‐ or (*S*)‐(1‐^13^C,1‐^2^H)IPP^[48]^ with GGPPS and PmS or *Si*CotB2, respectively (Figures S131 and S132).

## Conclusions

After our previous reports on the biosynthesis of diterpene analogs from different GGPP isomers with shifted double bonds, we now report the synthesis of four more GGPP isomers named *iso*‐GGPP IV–VII. While *iso*‐GGPP V and VII were not accepted by any of the tested diterpene synthases, the analogs *iso*‐GGPP IV and VI can serve as efficient substrates in several cases. This often leads to the formation of compounds with complex skeletons that are as a consequence of the changed reactivity in the substrate analogs different to naturally observed skeletons. Two newly investigated enzymes, Bnd4 from *S. iakyrus* and VenA from *S. exfoliates*, that are homologs of previously reported enzymes exhibiting the same function,[^35^, ^41^] were converted with *iso*‐GGPP I to benditerpe‐2,7(19),15‐triene (**45**) and venezuelaxenene (**47**), and their biosynthesis was investigated through isotopic labelling experiments, showing an early stage 1,3‐hydride shift for **45**, but two sequential 1,2‐hydride shifts for **47**, respectively. The same findings were made for the natural products of these enzymes obtained from GGPP, i. e. benditerpe‐2,6,15‐triene (**41**) and venezuelaene A (**46**). Due to severe line broadening in the NMR spectra of **41** interpretation of the results from the labelling experiments for this compound required NBS derivatization to obtain compounds with rigidified skeleton.

The observation that minor structural changes in terpenoid substrates can result in previously unknown skeletons using the enzymatic approach in this study is of high interest, because this method outcompetes total synthesis approaches for the rapid structural diversification of terpene skeletons. Future work on oxidative and other modifications of the obtained diterpene hydrocarbons will be of interest to gain access to potentially bioactive compounds.

## Experimental Section

**General methods**. Chemicals were purchased from Sigma Aldrich Chemie GmbH (Steinheim, Germany), Carbolution Chemicals GmbH (St. Ingbert, Germany), or Carl Roth (Karlsruhe, Germany) and used without purification. Solvents for column chromatography were purchased in p.a. grade and purified by distillation. Thin‐layer chromatography (TLC) was performed with 0.2 mm precoated plastic sheets Polygram Sil G/UV254 purchased from Machery‐Nagel (Düren, Germany). Column chromatography was performed using silica gel 60 purchased from Merck (Darmstadt, Germany).

**GC/MS**. GC/MS analyses were performed on a 5977 A GC/MSD system (Agilent, Santa Clara, CA, USA) with a 7890B GC and a 5977 A mass selective detector. The GC was equipped with a HP5‐MS fused silica capillary column (30 m, 0.25 mm i. d., 0.50 μm film). Specific GC settings were 1) inlet pressure: 77.1 kPa, He at 23.3 mL min^−1^, 2) injection volume: 1 μL, 3) temperature program: 5 min at 50 °C increasing at 10 °C min^−1^ to 320 °C, 4) 60 s valve time, and 5) carrier gas: He at 1.2 mL min^−1^. MS settings were 1) source: 230 °C, 2) transfer line: 250 °C, 3) quadrupole: 150 °C and 4) electron energy: 70 eV. Retention indices (*I*) were determined from retention times in comparison to the retention times of *n*‐alkanes (C_7_‐C_40_).

**HRMS**. High resolution mass spectra were recorded on an Orbitrap XL instrument (APCI; Thermo Fisher Scientific, Waltham, MA, USA) or using a 7890B/7200 series gas chromatography/accurate mass Q‐ToF detector system (Agilent). The GC was equipped with a HP5‐MS fused silica capillary column (30 m, 0.25 mm i. d., 0.50 μm film). GC settings were 1) injection volume: 1 μL, 2) temperature program: 5 min at 50 °C, increasing 10 °C min^−1^ to 320 °C, 3) split ratio: 5 : 1, 60 s valve time and 4) carrier gas flow: He at 1 mL min^−1^. MS settings were 1) inlet pressure: 83.2 kPa, He flow at 24.6 mL min^−1^, 2) transfer line temperature: 250 °C, 3) ionization energy: 70 eV.

**HPLC**. Analytical scale HPLC seperation was carried out using a EUROPA HPLC system (Knauer, Berlin, Germany), equipped with UV/Vis‐DAD detector (190–900 nm) and a KNAUER Eurospher II 100–3 C18 column (3.0 μm, 2.0 mm ×100 mm). The UV‐Vis absorption was monitored at 190–600 nm. Preparative scale HPLC purification was performed on an Azura series HPLC system (Knauer, Berlin, Germany) with a multi wavelength detector MWL 2.1 L (190 – 700 nm) using a Macherey‐Nagel Nucleodur 100–5 Gravity C18 column (5 μm, 10 x 250 mm).

**NMR spectroscopy**. NMR spectra were recorded on a Bruker (Billerica, MA, USA) Avance I (300 MHz), Avance I (400 MHz), Avance I (500 MHz), Avance III HD Prodigy (500 MHz) or an Avance III HD Cryo (700 MHz) NMR spectrometer. Spectra were measured in C_6_D_6_ and referenced against solvent signals (^1^H‐NMR, residual proton signal: *δ* =7.16 ppm; ^13^C‐NMR: *δ* =128.06 ppm).^[49]^

**IR spectroscopy**. IR spectra were recorded on a Bruker α infrared spectrometer with a diamond ATR probehead. Peak intensities are given as s (strong), m (medium), w (weak) and br (broad).

**Optical rotations**. Optical rotations were recorded on a Modular Compact Polarimeter MCP 100 (Anton Paar, Graz, Austria). The temperature setting was 25 °C, the wavelength of the light used was 589 nm (sodium D line), the path‐length was 10 cm, and the compound concentrations *c* are given in g 100 mL^−1^.

**General procedure for synthesis of allyl bromides**. To a cooled (0 °C) solution of alcohol (1.00 eq) in Et_2_O (3 mL mmol^−1^) PBr_3_ (0.40 eq) was added dropwise. The mixture was stirred for 1 h at 0 °C and was transferred directly to an ice/water mixture. The aqueous layer was extracted with Et_2_O three times and the organic layers were dried with MgSO_4_, concentrated under reduced pressure and the bromides were directly used for subsequent reactions without purification.

**General procedure for nucleophilic substitution with the dianion of isopentenol**. To a solution of *n*Buli (1.6 m in hexane, 2.00 eq) in hexane (0.75 mL mmol^−1^, −15 °C) was added TMEDA (2.00 eq) and the mixture was stirred for 0.5 h at –23 °C. The mixture was warmed to room temperature and isopentenol (**8**, 1.00 eq) was added dropwise. After 6 h stirring, HMPA (3 mL) and a solution of the corresponding allyl bromide or alkyl iodides (1.00 eq) in Et_2_O was added at −78 °C, and the mixture was allowed to warm to room temperature overnight. The reaction was quenched by the addition of 1 m HCl and extracted with Et_2_O three times. The combined organic layers were dried with MgSO_4_, evaporated under reduced pressure and subjected to column chromatography (petroleum ether/Et_2_O, 3 : 1) to yield the desired alcohols.

7‐Methyl‐3‐methyleneoct‐6‐en‐1‐ol (**9**).^[50]^ Prepared from prenyl bromide (**7**). Yield: 7.28 g (47 mmol, 68 %) TLC (pentane:Et_2_O=2 : 1): *R*_f_=0.35. GC (HP‐5): *I*=1219. ^1^H‐NMR (C_6_D_6_, 500 MHz): *δ* =5.15 (thept, *J*=6.9, *J*=1.4, 1H), 4.83 (m, 1H), 4.77 (m, 1H), 3.48 (m, 2H), 2.10 (m, 4H), 1.97 (m, 2H), 1.65 (d, *J*=1.2, 3H), 1.52 (br s, 3H) ppm. ^13^C‐NMR (C_6_D_6_, 125 MHz): *δ* =146.48 (C_q_), 131.54 (C_q_), 124.57 (CH), 111.45 (CH_2_), 60.78 (CH_2_), 39.74 (CH_2_), 36.36 (CH_2_), 26.80 (CH_2_), 25.82 (CH_3_), 17.74 (CH_3_) ppm.

11‐Methyl‐3,7‐dimethylenedodec‐10‐en‐1‐ol (**11**). Prepared from the iodide **10**. Yield: 586 mg (2.64 mmol, 53 %) TLC (pentane:Et_2_O=2 : 1): *R*_f_=0.30. GC (HP‐5): *I*=1690. ^1^H‐NMR (C_6_D_6_, 500 MHz): *δ* =5.23 (ddq, 1H, *J*=8.4, 5.5, 1.4 Hz), 4.88 (dhept, 1H, *J*=2.1, 0.8 Hz), 4.85 (m, 1H), 4.81 (m, 1H), 4.76 (m, 1H), 3.47 (td, 2H, *J*=6.6, 5.4 Hz), 2.19 (m, 2H), 2.07 (m, 4H), 1.98 (m, 2H), 1.90 (m, 2H), 1.67 (m, 3H), 1.56 (d, 3H, *J*=1.4 Hz), 1.53 (m, 2H) ppm. ^13^C‐NMR (C_6_D_6_, 125 MHz): *δ* =149.39 (C_q_), 146.56 (C_q_), 131.43 (C_q_), 124.77 (CH), 111.43 (CH_2_), 109.60 (CH_2_), 60.77 (CH_2_), 39.62 (CH_2_), 36.47 (CH_2_), 36.10 (CH_2_), 35.95 (CH_2_), 26.95 (CH_2_), 26.17 (CH_2_), 25.85 (CH_3_), 17.75 (CH_3_) ppm. IR (diamond ATR): ṽ=3345 (br), 3073 (m), 2966 (s), 2903 (m), 1738 (m), 1644 (m), 1440 (m), 1375 (m), 1216 (w), 1045 (m), 888 (s), 820 (w) cm^−1^. HR‐MS (Q‐TOF, 70 eV): calc. [C_15_H_26_O]^+^⋅ *m*/*z*=222.1979, found: *m*/*z*=222.1982.

(*E*)‐7,11‐Dimethyl‐3‐methylenedodeca‐6,11‐dien‐1‐ol (**19**). Prepared from the bromide corresponding to **18**. Yield: 1.04 g (4.68 mmol, 37 %) TLC (pentane:Et_2_O=1 : 1): *R*_f_=0.52. GC (HP‐5): *I*=1691. ^1^H‐NMR (C_6_D_6_, 500 MHz): *δ* =5.19 (ddq, 1H, *J*=8.4, 7.0, 1.3 Hz), 4.81 (m, 4H), 3.48 (td, 2H, *J*=6.6, 3.7 Hz), 2.12 (m, 4H), 1.98 (m, 6H), 1.66 (m, 3H), 1.54 (m, 3H) ppm. ^13^C‐NMR (C_6_D_6_, 125 MHz): *δ* =146.46 (C_q_), 145.82 (C_q_), 135.38 (C_q_), 124.53 (CH), 111.50 (CH_2_), 110.40 (CH_2_), 60.78 (CH_2_), 39.72 (CH_2_), 39.61 (CH_2_), 37.68 (CH_2_), 36.38 (CH_2_), 26.66 (CH_2_), 26.30 (CH_2_), 22.52 (CH_3_), 16.02 (CH_3_) ppm. IR (diamond ATR): ṽ=3345 (br), 3073 (m), 2966 (m), 2933 (s), 2861 (m), 1738 (m), 1646 (m), 1442 (m), 1373 (m), 1216 (w), 1044 (m), 886 (s), 821 (w) cm^−1^. HR‐MS (Q‐TOF, 70 eV): calc. [C_15_H_26_O]^+^⋅ *m*/*z*=222.1979, found: *m*/*z*=222.1977.

7‐Methyl‐3‐methyleneoct‐7‐en‐1‐ol (**26**). Prepared from isopentenyl iodide. Yield: 4.64 g (30.1 mmol, 60 %) TLC (pentane:Et_2_O=2 : 1): *R*_f_=0.26. GC (HP‐5): *I*=1239. ^1^H‐NMR (C_6_D_6_, 500 MHz): *δ* =4.80 (dt, 2H, *J*=2.1, 1.1 Hz), 4.77 (ddt, 2H, *J*=9.0, 2.0, 1.1 Hz), 3.47 (t, 2H, *J*=6.6 Hz), 2.07 (td, 2H, *J*=6.6, 1.4 Hz), 1.89 (m, 2H), 1.62 (m, 3H), 1.47 (m, 2H) ppm. ^13^C‐NMR (C_6_D_6_, 125 MHz): *δ* =146.54 (C_q_), 145.57 (C_q_), 111.40 (CH_2_), 110.50 (CH_2_), 60.78 (CH_2_), 39.61 (CH_2_), 37.65 (CH_2_), 35.82 (CH_2_), 25.95 (CH_2_), 22.42 (CH_3_) ppm. IR (diamond ATR): ṽ=3346 (br), 3073 (w), 2934 (s), 2884 (m), 1646 (m), 1443 m), 1373 (w), 1045 (s), 885 (s) cm^−1^. HR‐MS (Q‐TOF, 70 eV): calc. [C_10_H_18_O]^+^⋅ *m*/*z*=154.1353, found: *m*/*z*=154.1350.

11‐Methyl‐3,7‐dimethylenedodec‐11‐en‐1‐ol (**34**). Prepared from the iodide **27**. Yield: 3.49 g (15.7 mmol, 52 %) TLC (pentane:Et_2_O=1 : 1): *R*_f_=0.51. GC (HP‐5): *I*=1677. ^1^H‐NMR (C_6_D_6_, 500 MHz): *δ* =4.84 (m, 2H), 4.81 (m, 3H), 4.77 (m, 1H), 3.48 (t, 2H, *J*=5.8 Hz), 2.09 (t, 2H, *J*=6.6 Hz), 1.96 (m, 6H), 1.90 (m, 2H), 1.64 (d, 3H, *J*=1.2 Hz), 1.55 (m, 4H) ppm. ^13^C‐NMR (C_6_D_6_, 125 MHz): *δ* =149.43 (C_q_), 146.57 (C_q_), 145.65 (C_q_), 111.42 (CH_2_), 110.50 (CH_2_), 109.61 (CH_2_), 60.79 (CH_2_), 39.62 (CH_2_), 37.76 (CH_2_), 35.95 (CH_2_), 35.92 (CH_2_), 26.16 (CH_2_), 26.10 (CH_2_), 22.46 (CH_3_) ppm. IR (diamond ATR): ṽ=3335 (br), 3073 (m), 2934 (m), 1646 (m), 1442 (w), 1373 (w), 1044 (m), 885 (s) cm^−1^. HR‐MS (Q‐TOF, 70 eV): calc. [C_15_H_26_O]^+^⋅ *m*/*z*=222.1979, found: *m*/*z*=222.1980.

### Synthesis of *iso*‐GGPP IV

**Synthesis of 8‐iodo‐2‐methyl‐6‐methyleneoct‐2‐ene (10).^[20]^** A solution of imidazole (2.61 g, 38.4 mmol, 1.20 eq) and PPh_3_ (10.08 g, 38.4 mmol, 1.20 eq) in CH_2_Cl_2_ (90 mL) was mixed with I_2_ (9.77 g, 38.4 mmol, 1.20 eq) and the corresponding alcohol **9** (4.97 g, 32 mmol, 1.00 eq) subsequently. After stirring for 4 h at room temperature, the reaction mixture was quenched by the addition of sat. aqueous NH_4_Cl and then extracted with Et_2_O (150 mL) three times. The combined organic layers were dried with MgSO_4_ and concentrated under reduced pressure. The residue was taken up in petroleum ether, the precipitate was filtered off, and the petroleum ether was removed in vacuum. The product was purified by column chromatography (petroleum ether) to yield the iodide **10** (5.98 g, 23 mmol, 71 %) as a pink oil. 5.98 g (23 mmol, 71 %) TLC (pentane:Et_2_O=pentane): *R*_f_=0.50. GC (HP‐5): *I*=1401. ^1^H‐NMR (C_6_D_6_, 500 MHz): *δ* =5.09 (thept, *J*=7.0, *J*=1.4, 1H), 4.78 (m, 1H), 4.62 (m, 1H), 2.82 (t, *J*=7.4, 2H), 2.29 (t, *J*=7.4, 2H), 2.00 (m, 2H), 1.83 (m, 2H), 1.65 (d, *J*=1.2, 3H), 1.49 (br s, 3H) ppm. ^13^C‐NMR (C_6_D_6_, 125 MHz): *δ* =147.88 (C_q_), 131.63 (C_q_), 124.35 (CH), 111.34 (CH_2_), 40.66 (CH_2_), 35.59 (CH_2_), 26.58 (CH_2_), 25.81 (CH_3_), 17.73 (CH_3_), 3.28 (CH_2_) ppm.

**Synthesis of 12‐iodo‐2‐methyl‐6,10‐dimethylenedodec‐2‐ene (12)**. A solution of imidazole (0.88 g, 13.0 mmol, 1.20 eq) and PPh_3_ (3.41 g, 13.0 mmol, 1.20 eq) in CH_2_Cl_2_ (30 mL) was mixed with I_2_ (3.31 g, 13.0 mmol, 1.20 eq) and the corresponding alcohol **11** (2.40 g, 10.8 mmol, 1.00 eq) subsequently. After stirring for 4 h at room temperature, the reaction mixture was quenched by the addition of sat. aqueous NH_4_Cl and then extracted with Et_2_O (150 mL) three times. The combined organic layers were dried with MgSO_4_ and concentrated under reduced pressure. The residue was taken up in petroleum ether, the precipitate was filtered off, and the petroleum ether was removed in vacuum. The product was purified by column chromatography (petroleum ether) to yield the iodide **12** (2.20 g, 6.6 mmol, 61 %) as a pink oil. 2.20 g (6.6 mmol, 61 %) TLC (pentane): *R*_f_=0.54. GC (HP‐5): *I*=1880. ^1^H‐NMR (C_6_D_6_, 500 MHz): *δ* =5.22 (tp, 1H, *J*=7.0, 1.5 Hz), 4.86 (qt, 2H, *J*=1.5, 0.7 Hz), 4.82 (qt, 2H, *J*=1.5, 0.7 Hz), 4.77 (qt, 2H, *J*=1.4, 0.7 Hz), 4.62 (tp, 2H, *J*=1.4, 0.6 Hz), 2.82 (t, 3H, *J*=7.4 Hz), 2.28 (m, 4H), 2.18 (m, 2H), 2.05 (m, 2H), 1.92 (m, 2H), 1.76 (m, 2H), 1.67 (m, 3H), 1.56 (m, 3H), 1.43 (m, 2H) ppm. ^13^C‐NMR (C_6_D_6_, 125 MHz): *δ* =149.23 (C_q_), 147.91 (C_q_), 131.48 (C_q_), 124.73 (CH), 111.32 (CH_2_), 109.67 (CH_2_), 40.53 (CH_2_), 36.45 (CH_2_), 35.99 (CH_2_), 35.17 (CH_2_), 26.94 (CH_2_), 25.93 (CH_2_), 25.86 (CH_3_), 17.78 (CH_3_), 3.28 (CH_2_) ppm. IR (diamond ATR): ṽ=3406 (w), 3076 (m), 2966 (s), 2856 (m), 1737 (m), 1671 (m), 1441 (m), 1376 (w), 1232 (w), 1169 (m), 1105 (w), 1043 (w), 889 (s), 820 (w) cm^−1^. HR‐MS (Q‐TOF, 70 eV): calc. [C_15_H_25_I]^+^⋅ *m*/*z*=332.0996, found: *m*/*z*=332.0995.

**Synthesis of ethyl 2‐acetyl‐13‐methyl‐5,9‐dimethylenetetradec‐12‐enoate (13)**. To a cooled solution of ethyl acetoacetate (1.71 g, 13.2 mmol, 2.00 eq) in THF (30 mL) was added NaH (0.53 g, 13.2 mmol, 2.00 eq, 60 % in mineral oil) in small portions. The reaction mixture was allowed to warm to room temperature and stirred for 1 h. The corresponding iodide **12** (2.20 g, 6.6 mmol, 1.00 eq) was added dropwise and the reaction mixture was refluxed overnight, followed by cooling to room temperature. The cooled mixture was quenched by the addition of sat. aqueous NH_4_Cl solution and then extracted with Et_2_O (50 mL) three times. The combined organic layers were dried with MgSO_4_ and concentrated under reduced pressure. The residue was subjected to column chromatography (petroleum ether/EtoAc, 10 : 1) to yield **13** (1.64 g, 4.9 mmol, 75 %) as a colourless oil. TLC (pentane:Et_2_O=8 : 1): *R*_f_=0.70. GC (HP‐5): *I*=2219. ^1^H‐NMR (C_6_D_6_, 500 MHz): *δ* =5.23 (tdq, 1H, *J*=7.0, 2.7, 1.4 Hz), 4.87 (m, 2H), 4.81 (m, 2H), 3.89 (qd, 2H, *J*=7.2, 1.9 Hz), 3.28 (ddd, 1H, *J*=7.8, 4.7, 1.8 Hz), 2.19 (m, 2H), 2.02 (m, 12H), 1.87 (s, 3H), 1.67 (m, 3H), 1.56 (m, 3H), 0.88 (t, 3H, *J*=7.2 Hz) ppm. ^13^C‐NMR (C_6_D_6_, 125 MHz): *δ* =201.42 (C_q_), 169.69 (C_q_), 149.40 (C_q_), 148.50 (C_q_), 131.40 (C_q_), 124.80 (CH), 110.50 (CH_2_), 109.61 (CH_2_), 61.05 (CH_2_), 59.26 (CH), 36.48 (CH_2_), 36.10 (CH_2_), 35.68 (CH_2_), 33.97 (CH_2_), 28.55 (CH_3_), 26.95 (CH_2_), 26.15 (CH_2_), 25.85 (CH_3_), 17.76 (CH_3_), 14.04 (CH_3_) ppm. IR (diamond ATR): ṽ=3074 (w), 2977 (m), 2932 (m), 2858 (m), 1740 (s), 1716 (s), 1644 (m), 1444 (m), 1358 (m), 1241 (m), 1215 (m), 1177 (m), 1147 (w), 1096 (w), 1023 (w), 887 (m) cm^−1^. HR‐MS (Q‐TOF, 70 eV): calc. [C_21_H_34_O_3_]^+^⋅ *m*/*z*=334.2503, found: *m*/*z*=334.2500.

**Synthesis of 14‐methyl‐6,10‐dimethylenepentadec‐13‐en‐2‐one (14)**. To a solution of β‐keto ester **13** (2.70 g, 8.0 mol, 1.00 eq) in EtOH (40 mL) was added an aqueous solution of KOH (1.34 g dissolved in 8 mL of water, 24 mmol, 3.00 eq) and the reaction mixture was stirred under reflux for 3 h before cooling to room temperature. The reaction mixture was slowly acidified with 2 m HCl solution until CO_2_ developed. The resulting mixture was extracted with Et_2_O (100 mL) three times. The combined layers were dried with MgSO_4_ and concentrated under reduced pressure. The residue was subject to column chromatography (petroleum ether/EtOAc, 15 : 1) to yield the desired methyl ketone **14** (2.05 g, 7.8 mmol, 98 %) as a colourless oil. TLC (pentane:Et_2_O=8 : 1): *R*_f_=0.40. GC (HP‐5): *I*=1917. ^1^H‐NMR (C_6_D_6_, 500 MHz): *δ* =5.23 (tdp, 1H, *J*=7.0, 2.9, 1.4 Hz), 4.88 (ddt, 2H, *J*=4.3, 2.1, 1.3 Hz), 4.80 (m, 2H), 2.20 (m, 2H), 2.10 (m, 2H), 1.95 (m, 10H), 1.67 (m, 3H), 1.64 (m, 3H), 1.60 (m, 2H), 1.56 (m, 3H) ppm. ^13^C‐NMR (C_6_D_6_, 125 MHz): *δ* =205.99 (C_q_), 149.47 (C_q_), 149.13 (C_q_), 131.40 (C_q_), 124.80 (CH), 109.89 (CH_2_), 109.59 (CH_2_), 42.64 (CH_2_), 36.50 (CH_2_), 36.17 (CH_2_), 35.82 (CH_2_), 35.61 (CH_2_), 29.40 (CH_3_), 26.97 (CH_2_), 26.24 (CH_2_), 25.85 (CH_3_), 21.95 (CH_2_), 17.76 (CH_3_) ppm. IR (diamond ATR): ṽ=3072 (w), 2930 (s), 2858 (m), 1717 (s), 1644 (m), 1441 (m), 1364 (m), 1158 (w), 888 (s) cm^−1^. HR‐MS (Q‐TOF, 70 eV): calc. [C_18_H_30_O]^+^⋅ *m*/*z*=262.2292, found: *m*/*z*=262.2292.

**Synthesis of ethyl (*E*)‐3,15‐dimethyl‐7,11‐dimethylenehexadeca‐2,14‐dienoate (15)**. A solution of diisopropylamine (828 mg, 8.2 mmol, 1.05 eq) dissolved in dry THF (30 mL) was cooled to 0 °C. *n*Buli (5.1 mL, 8.2 mmol, 1.6 m in hexane, 1.05 eq) was added dropwise and the reaction mixture was stirred for 1 h at 0 °C. The reaction was cooled to –78 °C and triethyl phosphonaacetate (1.78 g, 7.8 mmol, 1.00 eq) was added. After stirring the reaction mixture for 1 h at –78 °C the methyl ketone **14** (2.05 g, 7.8 mmol, 1.00 eq) was added. The reaction mixture was allowed to warm to room temperature and stirred overnight. Water (15 mL) was added to quench the reaction. The aqueous phase was extracted with Et_2_O (80 mL, 3 times), and the combined layers were dried with MgSO_4_ and concentrated under reduced pressure. Purification by column chromatography (petroleum ether/EtOAc, 15 : 1) yielded pure (*E*)‐**15** and (*Z*)‐**15** as colourless oils.

(*E*)‐**15**. Yield: 1.12 g (3.4 mmol, 44 %). TLC (pentane:Et_2_O=10 : 1): *R*_f_=0.59. GC (HP‐5): *I*=2324. ^1^H‐NMR (C_6_D_6_, 500 MHz): *δ* =5.82 (h, 1H, *J*=1.3 Hz), 5.24 (tdp, *J*=7.0, 4.3, 1.4 Hz), 4.88 (m, 2H), 4.79 (m, 2H), 4.06 (q, 2H, *J*=7.2 Hz), 2.20 (m, 5H), 2.09 (m, 2H), 2.02 (m, 2H), 1.94 (m, 2H), 1.84 (m, 2H), 1.67 (m, 3H), 1.59 (m, 2H), 1.56 (m, 3H), 1.41 (m, 2H), 1.02 (t, 3H, *J*=7.1 Hz) ppm. ^13^C‐NMR (C_6_D_6_, 125 MHz): *δ* =166.47 (C_q_), 159.53 (C_q_), 149.43 (C_q_), 148.98 (C_q_), 131.44 (C_q_), 124.76 (CH), 116.38 (CH), 109.82 (CH_2_), 109.62 (CH_2_), 59.41 (CH_2_), 40.52 (CH_2_), 36.51 (CH_2_), 36.16 (CH_2_), 35.91 (CH_2_), 35.71 (CH_2_), 26.96 (CH_2_), 26.23 (CH_2_), 25.85 (CH_3_), 25.68 (CH_2_), 18.73 (CH_3_), 17.76 (CH_3_), 14.46 (CH_3_) ppm. IR (diamond ATR): ṽ=3074 (w), 3046 (w), 2978 (m), 2932 (w), 1715 (s), 1645 (s), 1442 (m), 1377 (w), 1321 (w), 1270 (s), 1220 (s), 1141 (s), 1096 (w), 1074 (w), 1040 (m), 886 (s), 819 (w) cm^−1^. HR‐MS (Q‐TOF, 70 eV): calc. [C_22_H_36_O_2_]^+^⋅ *m*/*z*=332.2712, found: *m*/*z*=332.2712.

(*Z*)‐**15**. Yield: 370 mg (1.1 mmol, 14 %). TLC (pentane:Et_2_O=10 : 1): *R*_f_=0.64. GC (HP‐5): *I*=2261. ^1^H‐NMR (C_6_D_6_, 500 MHz): *δ* =5.75 (dt, 1H, *J*=1.4, 0.7 Hz), 5.23 (ddq, 1H, *J*=8.4, 5.6, 1.5 Hz), 4.87 (m, 4H), 4.03 (q, 2H, *J*=7.2 Hz), 2.72 (m, 2H), 2.19 (m, 3H), 2.10 (m, 4H), 2.03 (m, 3H), 1.67 (m, 3H), 1.62 (m, 4H), 1.56 (m, 3H), 1.53 (d, 3H, *J*=1.4 Hz), 1.01 (t, 3H, *J*=7.1 Hz) ppm. ^13^C‐NMR (C_6_D_6_, 125 MHz): *δ* =166.02 (C_q_), 160.05 (C_q_), 149.52 (C_q_), 149.46 (C_q_), 131.33 (C_q_), 124.85 (CH), 116.93 (CH), 109.60 (CH_2_), 109.55 (CH_2_), 59.38 (CH_2_), 36.51 (CH_2_), 36.49 (CH_2_), 36.21 (CH_2_), 36.04 (CH_2_), 33.40 (CH_2_), 26.97 (CH_2_), 26.81 (CH_2_), 26.28 (CH_2_), 25.85 (CH_2_), 24.89 (CH_2_), 17.76 (CH_2_), 14.43 (CH_2_) ppm. IR (diamond ATR): ṽ=3074 (w), 3045 (w), 2978 (m), 2932 (w), 1714 (s), 1643 (s), 1442 (m), 1377 (w), 1322 (w), 1270 (s), 1220 (s), 1141 (s), 1096 (w), 1074 (w), 1040 (m), 886 (s), 821 (w) cm^−1^. HR‐MS (Q‐TOF, 70 eV): calc. [C_22_H_36_O_2_]^+^⋅ *m*/*z*=332.2712, found: *m*/*z*=332.2715.

**Synthesis of (*E*)‐3,15‐dimethyl‐7,11‐dimethylenehexadeca‐2,14‐dien‐1‐ol (16)**. To a cooled (0 °C) solution of the ester (*E*)‐**15** (1.0 g, 3.1 mmol, 1.00 eq) in THF (15 mL) was added DIBAL−H (7.4 mL, 7.4 mmol, 1.0 m in hexane, 2.40 eq) and the reaction mixture was stirred for 1 h at room temperature. The mixture was cooled to 0 °C and a saturated solution of Na−K‐tartrate (15 mL) was added. The resulting slurry was stirred for 2 h to dissolve the precipitate and the aqueous phase was extracted with Et_2_O (50 mL, 3 times). The organic layers were dried with MgSO_4_ and concentrated under reduced pressure. The residue was purified by column chromatography (petroleum ether/Et_2_O, 3 : 1) to yield the alcohol **16** (836 mg, 2.9 mmol, 94 %) as a colourless oil. TLC (pentane:Et_2_O=2 : 1): *R*_f_=0.35. GC (HP‐5): *I*=2193. ^1^H‐NMR (C_6_D_6_, 500 MHz): *δ* =5.39 (tp, 1H, *J*=6.7, 1.3 Hz), 5.23 (ddq, 1H, *J*=8.4, 5.5, 1.4 Hz), 4.87 (m, 4H), 3.97 (d, 2H, *J*=6.7 Hz), 2.20 (m, 2H), 2.09 (m, 2H), 1.98 (m, 8H), 1.67 (d, 3H, *J*=1.4 Hz), 1.62 (m, 2H), 1.56 (d, 3H, *J*=1.2 Hz), 1.52 (m, 2H), 1.46 (s, 3H) ppm. ^13^C‐NMR (C_6_D_6_, 125 MHz): *δ* =149.52 (C_q_), 149.49 (C_q_), 138.15 (C_q_), 131.42 (C_q_), 125.04 (CH), 124.79 (CH), 109.59 (2x CH_2_), 59.37 (CH_2_), 39.46 (CH_2_), 36.51 (CH_2_), 36.21 (CH_2_), 36.09 (CH_2_), 35.96 (CH_2_), 26.97 (CH_2_), 26.32 (CH_2_), 26.20 (CH_2_), 25.85 (CH_3_), 17.76 (CH_3_), 16.10 (CH_3_) ppm. IR (diamond ATR): ṽ=3316 (br), 3072 (m), 2966 (s), 2859 (m), 1644 (m), 1441 (m), 1371 (w), 1217 (w), 1102 (w), 1001 (m), 887 (s), 822 (w) cm^−1^. HR‐MS (Q‐TOF, 70 eV): calc. [C_20_H_34_O]^+^⋅ *m*/*z*=290.2604, found: *m*/*z*=290.2600.

**Synthesis of (*E*)‐3,15‐dimethyl‐7,11‐dimethylenehexadeca‐2,14‐dien‐1‐yl diphosphate (17)**. To a solution of tris(tetra‐n‐butylammonium)hydrogen diphosphate (1.64 g, 1.82 mmol, 1.30 eq) in acetonitrile (5 mL) a solution of the allyl bromide (made from 1.00 eq of alcohol **16** followed by general procedure) was added. The mixture was stirred at room temperature overnight. Acetonitrile was removed under reduced pressure. The residue was dissolved in aqueous NH_4_HCO_3_ solution (1.0 mL, 0.25 m) and loaded onto a DOWEX 50WX8 ion‐exchange column (NH_4_^+^ form, pH 7.0). The column was flushed slowly with 1.5 volumes of NH_4_HCO_3_ buffer (25 mm, 5 % iPrOH) and the eluate was lyophilized to yield the diphosphate **17** as a colourless hygroscopic powder (1.0 g, 2.0 mmol, 100 %). ^1^H‐NMR (D_2_O, 500 MHz): *δ* =5.42 (m, 1H), 5.10 (m, 1H), 4.72 (m, 4H), 4.48 (m, 2H), 2.04 (m, 12H), 1.70 (m, 3H), 1.65 (m, 4H), 1.59 (m, 3H), 1.38 (m, 5H) ppm. ^13^C‐NMR (D_2_O, 125 MHz): *δ* =149.36 (C_q_), 149.01 (C_q_), 140.93 (C_q_), 131.05 (C_q_), 124.27 (CH), 120.89 (d, CH, ^3^ *J*_C,P_=7.6 Hz), 109.02 (CH_2_), 108.89 (CH_2_), 62.47 (d, CH_2_, ^2^ *J*_C,P_=4.1 Hz), 39.31 (CH_2_), 36.10 (CH_2_), 35.82 (CH_2_), 35.78 (2x CH_2_), 26.48 (CH_2_), 25.90 (CH_2_), 25.78 (CH_2_), 25.49 (CH_3_), 17.45 (CH_3_), 15.90 (CH_3_) ppm. ^31^P‐NMR (D_2_O, 203 MHz): *δ* =–8.60 (d, ^2^ *J*_P,P_=16.7 Hz), −10.63 (d, ^2^ *J*_P,P_=18.1 Hz) ppm. HR‐MS (APCI): calc. for [C_20_H_35_O_7_P_2_]^−^ m/z=449.1863; found: *m*/*z*=449.1868.

### Synthesis of *iso*‐GGPP V

**Synthesis of (*E*)‐12‐iodo‐2,6‐dimethyl‐10‐methylenedodeca‐1,6‐diene (20)**. A solution of imidazole (1.27 g, 18.6 mmol, 1.20 eq) and PPh_3_ (4.89 g, 18.6 mmol, 1.20 eq) in CH_2_Cl_2_ (45 mL) was mixed with I_2_ (4.71 g, 18.6 mmol, 1.20 eq) and the corresponding alcohol **19** (3.45 g, 15.5 mmol, 1.00 eq) subsequently. After stirring for 4 h at room temperature, the reaction mixture was quenched by the addition of sat. aqueous NH_4_Cl and then extracted with Et_2_O (100 mL) three times. The combined organic layers were dried with MgSO_4_ and concentrated under reduced pressure. The residue was taken up in petroleum ether, the precipitate was filtered off, and the petroleum ether was removed under reduced pressure. The product was purified by column chromatography (petroleum ether) to yield the iodide **20** (3.1 g, 9.3 mmol, 60 %) as a pink oil. TLC (pentane): *R*_f_=0.60. GC (HP‐5): *I*=1884. ^1^H‐NMR (C_6_D_6_, 500 MHz): *δ* =5.13 (tq, 1H, *J*=6.9, 1.3 Hz), 4.83 (dt, 2H, *J*=2.1, 1.0 Hz), 4.79 (m, 1H), 4.63 (m, 1H), 2.83 (t, 2H, *J*=7.6 Hz), 2.30 (td, 2H, *J*=7.7, 1.4 Hz), 2.03 (m, 2H), 1.96 (m, 4H), 1.85 (m, 2H), 1.66 (t, 3H, *J*=1.3 Hz), 1.55 (m, 2H), 1.51 (d, 3H, *J*=1.4 Hz) ppm. ^13^C‐NMR (C_6_D_6_, 125 MHz): *δ* =147.88 (C_q_), 145.78 (C_q_), 135.49 (C_q_), 124.31 (CH), 111.39 (CH_2_), 110.43 (CH_2_), 40.64 (CH_2_), 39.60 (CH_2_), 37.69 (CH_2_), 35.62 (CH_2_), 26.47 (CH_2_), 26.30 (CH_2_), 22.53 (CH_3_), 16.02 (CH_3_), 3.02 (CH_2_) ppm. IR (diamond ATR): ṽ=3404 (w), 3075 (m), 2965 (s), 2856 (m), 1737 (m), 1670 (m), 1441 (m), 1376 (w), 1232 (w), 1169 (m), 1110 (w), 1043 (w), 886 (s), 820 (w) cm^−1^. HR‐MS (Q‐TOF, 70 eV): calc. [C_15_H_25_I]^+^⋅ *m*/*z*=332.0996, found: *m*/*z*=332.0991.

**Synthesis of ethyl (*E*)‐2‐acetyl‐9,13‐dimethyl‐5‐methylenetetradeca‐8,13‐dienoate (21)**. To a cooled solution of ethyl acetoacetate (2.43 g, 18.7 mmol, 2.00 eq) in THF (35 mL) was added NaH (0.45 g, 18.7 mmol, 2.00 eq, 60 % in mineral oil) in small portions. The reaction mixture was allowed to warm to room temperature and stirred for 1 h. The corresponding iodide **20** (3.1 g, 9.3 mmol, 1.00 eq) was added dropwise and the reaction mixture was stirred under reflux overnight, followed by cooling to room temperature. The cooled mixture was quenched by the addition of sat. aqueous NH_4_Cl solution and then extracted with Et_2_O (100 mL) three times. The combined organic layers were dried with MgSO_4_ and concentrated under reduced pressure. The residue was subjected to column chromatography (petroleum ether/EtoAc, 10 : 1) to yield **21** (2.30 g, 6.9 mmol, 74 %) as a colourless oil. TLC (pentane:Et_2_O=5 : 1): *R*_f_=0.45. GC (HP‐5): *I*=2223. ^1^H‐NMR (C_6_D_6_, 500 MHz): *δ* =5.21 (tq, 1H, *J*=7.0, 1.3 Hz), 4.83 (m, 4H), 3.89 (qd, 2H, *J*=7.1, 1.6 Hz), 3.28 (m, 1H), 2.16 (m, 2H), 2.02 (m, 12H), 1.87 (s, 3H), 1.66 (s, 3H), 1.56 (m, 3H), 0.88 (t, 3H, *J*=7.1 Hz) ppm. ^13^C‐NMR (C_6_D_6_, 125 MHz): *δ* =201.43 (C_q_), 169.69 (C_q_), 148.40 (C_q_), 145.83 (C_q_), 135.38 (C_q_), 124.54 (CH), 110.57 (CH_2_), 110.39 (CH_2_), 61.04 (CH_2_), 59.26 (CH), 39.63 (CH_2_), 37.70 (CH_2_), 36.12 (CH_2_), 34.06 (CH_2_), 28.55 (CH_3_), 26.64 (CH_2_), 26.55 (CH_2_), 26.31 (CH_2_), 22.52 (CH_3_), 16.03 (CH_3_), 14.03 (CH_3_) ppm. IR (diamond ATR): ṽ=3072 (w), 2968 (m), 2933 (s), 2859 (m), 1738 (m), 1646 (m), 1434 (m), 1373 (m), 1229 (m), 1216 (m), 1169 (w), 887 (s) cm^−1^. HR‐MS (Q‐TOF, 70 eV): [C_21_H_34_O_3_]^+^⋅ *m*/*z*=334.2503, found: *m*/*z*=334.2500.

**Synthesis of (*E*)‐10,14‐dimethyl‐6‐methylenepentadeca‐9,14‐dien‐2‐one (22)**. To a solution of β‐keto ester **21** (2.30 g, 6.9 mol, 1.00 eq) in EtOH (20 mL) was added an aqueous solution of KOH (1.16 g dissolved in 5 mL of water, 20.7 mmol, 3.00 eq) and the reaction mixture was stirred under reflux for 3 h before cooling to room temperature. The reaction mixture was slowly acidified with 2 m HCl solution until CO_2_ developed. The resulting mixture was extracted with Et_2_O (100 mL) three times. The combined organic layers were dried with MgSO_4_ and concentrated under reduced pressure. The residue was subjected to column chromatography (petroleum ether/EtOAc, 13 : 1) to yield the desired methyl ketone **22** (1.73 g, 6.6 mmol, 96 %) as a colourless oil. TLC (pentane:Et_2_O=8 : 1): *R*_f_=0.45. GC (HP‐5): *I*=1913. ^1^H‐NMR (C_6_D_6_, 500 MHz): *δ* =5.25 (ddq, 1H, *J*=8.4, 7.0, 1.4 Hz), 4.82 (m, 4H), 2.19 (m, 2H), 2.06 (m, 2H), 1.94 (m, 8H), 1.65 (m, 8H), 1.58 (m, 3H), 1.55 (m, 2H) ppm. ^13^C‐NMR (C_6_D_6_, 125 MHz): *δ* =205.98 (C_q_), 149.04 (C_q_), 145.83 (C_q_), 135.29 (C_q_), 124.68 (CH), 110.39 (CH_2_), 109.94 (CH_2_), 42.65 (CH_2_), 39.64 (CH_2_), 37.70 (CH_2_), 36.26 (CH_2_), 35.72 (CH_2_), 29.40 (CH_3_), 26.74 (CH_2_), 26.33 (CH_2_), 22.52 (CH_3_), 21.96 (CH_2_), 16.04 (CH_3_) ppm. IR (diamond ATR): ṽ=3073 (w), 2934 (s), 2856 (m), 1716 (s), 1644 (m), 1442 (m), 1364 (m), 1158 (w), 884 (s) cm^−1^. HR‐MS (Q‐TOF, 70 eV): calc. [C_18_H_30_O]^+^⋅ *m*/*z*=262.2292, found: *m*/*z*=262.2296.

**Synthesis of ethyl (2*E*,10*E*)‐3,11,15‐trimethyl‐7‐methylenehexadeca‐2,10,15‐trienoate (23)**. NaH (406 mg, 10.2 mmol, 2.00 eq, 60 % in mineral oil) suspended in dry THF (25 mL) was cooled to 0 °C. Triethyl phosphonoacetate (2.50 g, 11.1 mmol, 2.20 eq) was added. After stirring the reaction mixture for 1 h at 0 °C, the reaction mixture was cooled to –78 °C and the methyl ketone **22** (1.33 g, 5.3 mmol, 1.00 eq) was added. The reaction mixture was allowed to warm to room temperature and stirred overnight. Water (10 mL) was added to quench the reaction. The aqueous phase was extracted with Et_2_O (100 mL, 3 times), and the combined layers were dried with MgSO_4_ and concentrated under reduced pressure. Purification by column chromatography (petroleum ether/EtOAc, 15 : 1) yielded pure (*E*)‐**23** and (*Z*)‐**23** as colourless oils.

(*E*)‐**23**. Yield: 740 mg (2.3 mmol, 43 %). TLC (pentane:Et_2_O=8 : 1): *R*_f_=0.65. GC (HP‐5): *I*=2314. ^1^H‐NMR (C_6_D_6_, 500 MHz): *δ* =5.82 (q, 1H, *J*=1.3 Hz), 5.24 (qd, 1H, *J*=5.6, 1.4 Hz), 4.81 (m, 4H), 4.06 (q, 2H, *J*=7.1 Hz), 2.20 (d, 3H, *J*=1.2 Hz), 2.17 (m, 2H), 2.00 (m, 6H), 1.85 (m, 4H), 1.66 (t, 3H, *J*=1.2 Hz), 1.57 (m, 5H), 1.42 (m, 2H), 1.02 (t, 3H, *J*=7.1 Hz) ppm. ^13^C‐NMR (C_6_D_6_, 125 MHz): *δ* =166.28 (C_q_), 159.35 (C_q_), 148.71 (C_q_), 145.61 (C_q_), 135.12 (C_q_), 124.46 (CH), 116.18 (CH), 110.22 (CH_2_), 109.65 (CH_2_), 59.21 (CH_2_), 40.34 (CH_2_), 39.44 (CH_2_), 37.50 (CH_2_), 36.16 (CH_2_), 35.61 (CH_2_), 26.54 (CH_2_), 26.13 (CH_2_), 25.49 (CH_2_), 22.32 (CH_3_), 18.54 (CH_3_), 15.84 (CH_3_), 14.27 (CH_3_) ppm. IR (diamond ATR): ṽ=3074 (w), 2978 (m), 2933 (s), 2861 (m), 1716 (s), 1647 (s), 1443 (m), 1381 (m), 1348 (w), 1271 (w), 1220 (s), 1142 (s), 1096 (w), 1072 (m), 1039 (m), 885 (s) cm^−1^. HR‐MS (Q‐TOF, 70 eV): calc. [C_22_H_36_O_2_]^+^⋅ *m*/*z*=332.2711, found: *m*/*z*=332.2714.

(*Z*)‐**23**. Yield: 151 mg (0.45 mmol, 8 %). TLC (pentane:Et_2_O=8 : 1): *R*_f_=0.70. GC (HP‐5): *I*=2257. ^1^H‐NMR (C_6_D_6_, 500 MHz): *δ* =5.75 (m, 1H), 5.26 (m, 1H), 4.83 (m, 4H), 4.03 (q, 1H, *J*=7.2 Hz), 2.73 (m, 2H), 2.22 (m, 2H), 2.12 (m, 4H), 1.98 (m, 4H), 1.65 (s, 3H), 1.58 (s, 3H), 1.55 (m, 2H), 1.53 (m, 3H), 1.01 (t, 3H, *J*=7.1 Hz) ppm. ^13^C‐NMR (C_6_D_6_, 125 MHz): *δ* =166.02 (C_q_), 159.99 (C_q_), 149.40 (C_q_), 145.86 (C_q_), 135.15 (C_q_), 124.87 (CH), 116.96 (CH), 110.36 (CH_2_), 109.63 (CH_2_), 59.37 (CH_2_), 39.66 (CH_2_), 37.72 (CH_2_), 36.59 (CH_2_), 36.49 (CH_2_), 33.42 (CH_2_), 26.85 (CH_2_), 26.80 (CH_2_), 26.36 (CH_2_), 24.87 (CH_3_), 22.51 (CH_3_), 16.03 (CH_3_), 14.43 (CH_3_) ppm. IR (diamond ATR): ṽ=3076 (w), 2978 (m), 2932 (s), 2860 (m), 1714 (s), 1644 (s), 1444 (m), 1381 (m), 1348 (m), 1271 (w), 1221 (s), 1142 (s), 1096 (w), 1072 (m), 1039 (m), 886 (s) cm^−1^. HR‐MS (Q‐TOF, 70 eV): calc. [C_22_H_36_O_2_]^+^⋅ *m*/*z*=332.2711, found: *m*/*z*=332.2719.

**Synthesis of (2*E*,10*E*)‐3,11,15‐trimethyl‐7‐methylenehexadeca‐2,10,15‐trien‐1‐ol (24)**. To a cooled (0 °C) solution of the ester (*E*)‐**23** (740 mg, 2.2 mmol, 1.00 eq) in THF (10 mL) was added DIBAL−H (5.3 mL, 5.3 mmol, 1.0 m in hexane, 2.40 eq) and the reaction mixture was stirred for 1 h at room temperature. The mixture was cooled to 0 °C and a saturated solution of Na−K‐tartrate (10 mL) was added. The resulting slurry was stirred for 2 h to dissolve the precipitate and the aqueous phase was extracted with Et_2_O (50 mL, 3 times). The organic layers were dried with MgSO_4_ and concentrated under reduced pressure. The residue was purified by column chromatography (petroleum ether/Et_2_O, 3 : 1) to yield the alcohol **24** (530 mg, 1.8 mmol, 82 %) as a colourless oil. TLC (pentane:Et_2_O=2 : 1): *R*_f_=0.30. ^1^H‐NMR (C_6_D_6_, 500 MHz): *δ* =5.38 (tq, 1H, *J*=6.7, 1.4 Hz), 5.27 (tq, 1H, *J*=7.0, 1.3 Hz), 4.88 (m, 2H), 4.82 (m, 2H), 3.97 (dq, 2H, *J*=6.7, 0.8 Hz), 2.22 (m, 2H), 2.10 (m, 2H), 1.99 (m, 6H), 1.91 (m, 2H), 1.66 (m, 3H), 1.58 (m, 3H), 1.54 (m, 4H), 1.45 (m, 3H) ppm. ^13^C‐NMR (C_6_D_6_, 125 MHz): *δ* =149.43 (C_q_), 145.83 (C_q_), 138.17 (C_q_), 135.26 (C_q_), 125.01 (CH), 124.74 (CH), 110.40 (CH_2_), 109.61 (CH_2_), 59.37 (CH_2_), 39.65 (CH_2_), 39.47 (CH_2_), 37.69 (CH_2_), 36.52 (CH_2_), 36.07 (CH_2_), 26.81 (CH_2_), 26.32 (CH_2_), 26.21 (CH_2_), 22.52 (CH_3_), 16.10 (CH_3_), 16.04 (CH_3_) ppm. IR (diamond ATR): ṽ=3357 (br), 3073 (w), 2965 (m), 2933 (s), 2860 (m), 1727 (w), 1667 (w), 1646 (m), 1441 (m), 1375 (w), 1302 (w), 1097 (w), 1000 (m), 886 (s) cm^−1^. HR‐MS (Q‐TOF, 70 eV): calc. [C_20_H_34_O]^+^⋅ *m*/*z*=290.2605, found: *m*/*z*=290.2604.

**Synthesis of (2*E*,10*E*)‐3,11,15‐trimethyl‐7‐methylenehexadeca‐2,10,15‐trien‐1‐yl diphosphate (25)**. To a solution of tris(tetra‐n‐butylammonium)hydrogen diphosphate (1.67 g, 1.86 mmol, 1.20 eq) in acetonitrile (6 mL) a solution of the allyl bromide (made from 1.00 eq of alcohol **24** followed by the above mentioned general procedure) was added and the mixture was stirred at room temperature overnight. Acetonitrile was removed under reduced pressure. The residue was dissolved in aqueous NH_4_HCO_3_ solution (1.0 mL, 0.25 m) and loaded onto a DOWEX 50WX8 ion‐exchange column (NH_4_^+^ form, pH 7.0). The column was flushed slowly with 1.5 volumes of NH_4_HCO_3_ buffer (25 mm, 5 % iPrOH) and the eluate was lyophilized to yield the diphosphate **25** as a colourless hygroscopic powder (1.1 g, 2.0 mmol, 100 %). ^1^H‐NMR (C_6_D_6_, 500 MHz): *δ* =5.36 (q, 1H, *J*=7.5 Hz), 5.06 (m, 1H), 4.64 (m, 4H), 4.41 (m, 2H), 1.96 (m, 12H), 1.64 (m, 3H), 1.61 (s, 3H), 1.52 (m, 4H), 1.34 (m, 3H) ppm. ^13^C‐NMR (C_6_D_6_, 125 MHz): *δ* =149.55 (C_q_), 145.42 (C_q_), 140.44 (C_q_), 134.73 (C_q_), 124.56 (CH), 121.45 (CH, ^3^ *J*_P,C_=7.5 Hz), 110.05 (CH_2_), 108.98 (CH_2_), 62.41 (CH_2_, ^2^ *J*_P,C_=3.8 Hz), 39.39 (CH_2_), 39.26 (CH_2_), 37.30 (CH_2_), 36.30 (CH_2_), 35.89 (CH_2_), 26.39 (CH_2_), 26.01 (CH_2_), 25.90 (CH_2_), 22.24 (CH_3_), 15.96 (CH_3_), 15.76 (CH_3_) ppm. ^31^P‐NMR (D_2_O, 203 MHz): *δ* =–8.49 (d, ^2^ *J*_P,P_=19.1 Hz), –10.78 (d, ^2^ *J*_P,P_=19.8 Hz) ppm. HR‐MS (APCI): calc. for [C_20_H_35_O_7_P_2_]^−^ m/z=449.1863; found: *m*/*z*=449.1860.

### Synthesis of *iso*‐FPP III

**Synthesis of 8‐iodo‐2‐methyl‐6‐methyleneoct‐1‐ene (27)**. A solution of imidazole (5.47 g, 80.4 mmol, 1.20 eq) and PPh_3_ (21.1 g, 80.4 mmol, 1.20 eq) in CH_2_Cl_2_ (150 mL) was mixed with I_2_ (20.3 g, 80.4 mmol, 1.20 eq) and the corresponding alcohol **26** (10.39 g, 67.0 mmol, 1.00 eq) subsequently. After stirring for 2 h at room temperature, the reaction mixture was quenched by the addition of sat. aqueous NH_4_Cl and then extracted with Et_2_O (200 mL) three times. The combined organic layers were dried with MgSO_4_ and concentrated under reduced pressure. The residue was taken up in petroleum ether, the precipitate was filtered off, and the petroleum ether was removed under reduced pressure. The product was purified by column chromatography (petroleum ether) to yield the iodide **27** (17.2 g, 65.2 mmol, 97 %) as a pink oil. TLC (pentane): *R*_f_=0.52. GC (HP‐5): *I*=1422. ^1^H‐NMR (C_6_D_6_, 500 MHz): *δ* =4.77 (m, 2H), 4.68 (m, 2H), 2.82 (t, 2H, *J*=7.6 Hz), 2.26 (td, 2H, *J*=7.7, 1.4 Hz), 1.85 (td, 2H, *J*=7.6, 1.4 Hz), 1.73 (m, 2H), 1.60 (m, 3H), 1.37 (m, 2H) ppm. ^13^C‐NMR (C_6_D_6_, 125 MHz): *δ* =147.89 (C_q_), 145.41 (C_q_), 111.31 (CH_2_), 110.58 (CH_2_), 40.52 (CH_2_), 37.54 (CH_2_), 35.05 (CH_2_), 25.72 (CH_2_), 22.41 (CH_3_), 3.28 (CH_2_) ppm. IR (diamond ATR): ṽ=3073 (w), 2966 (m), 2934 (s), 2862 (m), 1647 (m), 1443 (m), 1373 (w), 1350 (w), 1232 (w), 1169 (m), 887 (s) cm^−1^. HR‐MS (Q‐TOF, 70 eV): calc. [C_10_H_17_I]^+^⋅ *m*/*z*=264.0371, found: *m*/*z*=264.0365.

**Synthesis of ethyl 2‐acetyl‐9‐methyl‐5‐methylenedec‐9‐enoate (28)**. To a cooled solution of ethyl acetoacetate (5.21 g, 40.0 mmol, 2.00 eq) in THF (40 mL) was added NaH (1.60 g, 40.0 mmol, 2.00 eq, 60 % in mineral oil) in small portions. The reaction mixture was allowed to reaction room temperature and stirred for 1 h. The corresponding iodide **27** (5.28 g, 20.0 mmol, 1.00 eq) was added dropwise and the reaction mixture was refluxed overnight, followed by cooling to room temperature. The cooled mixture was quenched by the addition of sat. aqueous NH_4_Cl solution and then extracted with Et_2_O (150 mL) three times. The combined organic layers were dried with MgSO_4_ and concentrated under reduced pressure. The residue was subjected to column chromatography (petroleum ether/EtoAc, 10 : 1) to yield **28** (3.10 g, 11.7 mmol, 60 %) as a colourless oil. TLC (pentane:Et_2_O=8 : 1): *R*_f_=0.39. GC (HP‐5): *I*=1798. ^1^H‐NMR (C_6_D_6_, 500 MHz): *δ* =4.80 (m, 4H), 3.89 (qd, 2H, *J*=7.1, 1.9 Hz), 3.28 (m, 1H), 2.03 (m, 4H), 1.92 (dd, 4H, *J*=9.1, 5.6 Hz), 1.87 (s, 3H), 1.63 (s, 3H), 1.52 (m, 2H) 0.88 (t, 3H, *J*=7.1 Hz) ppm. ^13^C‐NMR (C_6_D_6_, 125 MHz): *δ* =201.42 (C_q_), 169.69 (C_q_), 148.47 (C_q_), 145.57 (C_q_), 110.52 (CH_2_), 110.48 (CH_2_), 61.05 (CH_2_), 59.25 (CH), 37.66 (CH_2_), 35.56 (CH_2_), 33.94 (CH_2_), 28.55 (CH_3_), 26.52 (CH_2_), 25.93 (CH_2_), 22.44 (CH_3_), 14.03 (CH_3_) ppm. IR (diamond ATR): ṽ=3073 (w), 2970 (m), 2937 (m), 2877 (w), 1741 (s), 1716 (s), 1646 (w), 1446 (m), 1367 (m), 1242 (m), 1215 (m), 1148 (m), 1095 (w), 1041 (w), 888 (m) cm^−1^. HR‐MS (Q‐TOF, 70 eV): calc. [C_16_H_26_O_3_]^+^⋅ *m*/*z*=266.1877, found: *m*/*z*=266.1880.

**Synthesis of 10‐methyl‐6‐methyleneundec‐10‐en‐2‐one (29)**. To a solution of β‐keto ester **28** (3.10 g, 11.7 mol, 1.00 eq) in EtOH (40 mL) was added an aqueous solution of KOH (1.96 g dissolved in 10 mL of water, 35.0 mmol, 3.00 eq) and the reaction mixture was stirred under reflux for 3 h before cooling to room temperature. The reaction mixture was slowly acidified with 2 m HCl solution until CO_2_ developed. The resulting mixture was extracted with Et_2_O (150 mL) three times. The combined layers were dried with MgSO_4_ and concentrated under reduced pressure. The residue was subjected to column chromatography (petroleum ether/EtOAc, 13 : 1) to yield the desired methyl ketone **29** (2.74 g, 11.7 mmol, 100 %, solvent containing) as a colourless oil. TLC (pentane:Et_2_O=8 : 1): *R*_f_=0.30. GC (HP‐5): *I*=1435. ^1^H‐NMR (C_6_D_6_, 500 MHz): *δ* =4.81 (m, 2H), 4.78 (m, 2H), 1.92 (m, 10H), 1.64 (m, 6H), 1.55 (m, 2H) ppm. ^13^C‐NMR (C_6_D_6_, 125 MHz): *δ* =206.00 (C_q_), 149.11 (C_q_), 145.65 (C_q_), 110.50 (CH_2_), 109.87 (CH_2_), 42.64 (CH_2_), 37.74 (CH_2_), 35.69 (CH_2_), 35.60 (CH_2_) 29.40 (CH_3_), 26.02 (CH_2_), 22.46 (CH_3_), 21.95 (CH_2_) ppm. IR (diamond ATR): ṽ=3073 (w), 2935 (m), 1716 (s), 1646 (m), 1413 (m), 1365 (m), 1225 (w), 1158 (w), 865 (s) cm^−1^. HR‐MS (Q‐TOF, 70 eV): calc. [C_13_H_22_O]^+^⋅ *m*/*z*=194.1666, found: *m*/*z*=194.1665.

**Synthesis of ethyl (*E*)‐3,11‐dimethyl‐7‐methylenedodeca‐2,11‐dienoate (30)**. A solution of diisopropylamine (1.23 g, 12.2 mmol, 1.05 eq) dissolved in dry THF (40 mL) was cooled to 0 °C. *n*Buli (7.6 mL, 12.2 mmol, 1.6 m in hexane, 1.05 eq) was added dropwise and the reaction mixture was stirred for 1 h at 0 °C. The reaction was cooled to –78 °C and triethyl phosphonoacetate (2.61 g, 11.7 mmol, 1.00 eq) was added. After stirring the reaction mixture for 1 h at −78 °C the methyl ketone **29** (2.74 g, 11.7 mmol, 1.00 eq) was added. The reaction mixture was allowed to warm to room temperature and stirred overnight. Water (25 mL) was added to quench the reaction. The aqueous phase was extracted with Et_2_O (150 mL, 3 times), and the combined layers were dried with MgSO_4_ and concentrated under reduced pressure. Purification by column chromatography (petroleum ether/EtOAc, 15 : 1) yielded pure (*E*)‐**30** and (*Z*)‐**30** as colourless oils.

(*E*)‐**30**. Yield: 1.75 g (6.6 mmol, 57 %) TLC (pentane:Et_2_O=8 : 1): *R*_f_=0.68. GC (HP‐5): *I*=1850. ^1^H‐NMR (C_6_D_6_, 500 MHz): *δ* =5.82 (h, 1H, *J*=1.3 Hz), 4.79 (m, 4H), 4.06 (q, 2H, *J*=7.2 Hz), 2.20 (d, 3H, *J*=1.4 Hz), 1.89 (m, 8H), 1.64 (t, 3H, *J*=1.2 Hz), 1.53 (m, 2H), 1.40 (m, 2H), 1.02 (t, 3H, *J*=7.2 Hz) ppm. ^13^C‐NMR (C_6_D_6_, 125 MHz): *δ* =166.46 (C_q_), 159.53 (C_q_), 148.95 (C_q_), 145.60 (C_q_), 116.38 (CH), 110.53 (CH_2_), 109.80 (CH_2_), 59.42 (CH_2_) 40.50 (CH_2_), 37.72 (CH_2_), 35.78 (CH_2_), 35.67 (CH_2_), 26.02 (CH_2_), 25.66 (CH_2_), 22.45 (CH_3_), 18.73 (CH_3_), 14.46 (CH_3_) ppm. IR (diamond ATR): ṽ=3073 (w), 2979 (m), 2937 (m), 2879 (w), 1716 (s), 1648 (m), 1444 (w), 1367 (w), 1322 (w), 1272 (s), 1221 (s), 1040 (w), 886 (m) cm^−1^. HR‐MS (Q‐TOF, 70 eV): calc. [C_17_H_28_O_2_]^+^⋅ *m*/*z*=264.2084, found: *m*/*z*=264.2090.

(*Z*)‐**30**. Yield: 370 mg (1.6 mmol, 14 %) TLC (pentane:Et_2_O=8 : 1): *R*_f_=0.74. GC (HP‐5): *I*=1791. ^1^H‐NMR (C_6_D_6_, 500 MHz): *δ* =5.75 (dhept, 1H, *J*=1.4, 0.7 Hz), 4.83 (m, 4H), 4.02 (q, *J*=7.1 Hz), 2.73 (m, 2H), 2.10 (m, 2H), 1.99 (m, 2H), 1.64 (m, 3H), 1.58 (m, 2H), 1.53 (d, 3H, *J*=1.4 Hz), 1.00 (t, 3H, *J*=7.1 Hz) ppm. ^13^C‐NMR (C_6_D_6_, 125 MHz): *δ* =166.03 (C_q_), 160.06 (C_q_), 149.45 (C_q_), 145.71 (C_q_), 116.93 (CH), 110.45 (CH_2_), 109.59 (CH_2_), 59.38 (CH_2_), 37.79 (CH_2_), 36.47 (CH_2_), 35.92 (CH_2_), 33.39 (CH_2_), 26.81 (CH_2_), 24.88 (CH_3_), 22.46 (CH_3_), 14.42 (CH_3_) ppm. IR (diamond ATR): ṽ=3073 (w), 2977 (m), 2935 (s), 2865 (m), 1715 (s), 1647 (s), 1444 (m), 1375 (m), 1270 (s), 1227 (s), 1153 (s), 1077 (w), 1057 (w), 1036 (w), 886 (s), 858 (m) cm^−1^. HR‐MS (Q‐TOF, 70 eV): calc. [C_17_H_28_O_2_]^+^⋅ *m*/*z*=264.2084, found: *m*/*z*=264.2080.

**Synthesis of (*E*)‐3,11‐dimethyl‐7‐methylenedodeca‐2,11‐dien‐1‐ol (31)**. To a cooled (0 °C) solution of the ester (*E*)‐**30** (1.50 g, 5.7 mmol, 1.00 eq) in THF (25 mL) was added DIBAL−H (15.9 mL, 15.9 mmol, 1.0 m in hexane, 2.40 eq) and the reaction mixture was stirred for 1 h at room temperature. The mixture was cooled to 0 °C and a saturated solution of Na−K‐tartrate (15 mL) was added. The resulting slurry was stirred for 2 h to dissolve the precipitate and the aqueous phase was extracted with Et_2_O (100 mL, 3 times). The organic layers were dried with MgSO_4_ and concentrated under reduced pressure. The residue was purified by column chromatography (petroleum ether/Et_2_O, 3 : 1) to yield the alcohol **31** (943 mg, 4.2 mmol, 75 %) as a colourless oil. TLC (pentane:Et_2_O=2 : 1): *R*_f_=0.25. GC (HP‐5): *I*=1715. ^1^H‐NMR (C_6_D_6_, 500 MHz): *δ* =5.39 (tq, 1H, *J*=6.7, 1.4 Hz), 4.83 (m, 4H), 3.98 (d, 2H, *J*=6.7 Hz), 1.94 (m, 8H), 1.64 (s, 3H), 1.54 (m, 4H), 1.45 (m, 3H) ppm. ^13^C‐NMR (C_6_D_6_, 125 MHz): *δ* =149.50 (C_q_), 145.66 (C_q_), 138.15 (C_q_), 125.02 (CH), 110.50 (CH_2_), 109.57 (CH_2_), 59.37 (CH_2_), 39.46 (CH_2_), 37.77 (CH_2_), 35.95 (CH_2_), 26.19 (CH_2_), 26.10 (CH_2_), 22.46 (CH_3_), 16.10 (CH_3_) ppm. IR (diamond ATR): ṽ=3323 (br), 3073 (w), 2969 (m), 2934 (s), 2863 (m), 1738 (m), 1668 (w), 1646 (m), 1442 (m), 1374 (m), 1230 (w), 1099 (s), 1000 (s), 885 (s) cm^−1^. HR‐MS (Q‐TOF, 70 eV): calc. [C_15_H_26_O]^+^⋅ *m*/*z*=222.1979, found: *m*/*z*=222.1970.

**Synthesis of (*E*)‐3,11‐dimethyl‐7‐methylenedodeca‐2,11‐dien‐1‐yl diphosphate (32)**. To a solution of tris(tetra‐n‐butylammonium)hydrogen diphosphate (4.50 g, 5.0 mmol, 1.20 eq) in acetonitrile (20 mL) a solution of the allyl bromide (made from 1.00 eq of alcohol **31** following the above mentioned general procedure) was added and the mixture was stirred at room temperature overnight. Acetonitrile was removed under reduced pressure. The residue was dissolved in aqueous NH_4_HCO_3_ solution (1.0 mL, 0.25 m) and loaded onto a DOWEX 50WX8 ion‐exchange column (NH_4_^+^ form, pH 7.0). The column was flushed slowly with 1.5 volumes of NH_4_HCO_3_ buffer (25 mm, 5 % iPrOH) and the eluate was lyophilized to yield the diphosphate **32** as a colourless hygroscopic powder (1.75 g, 4.0 mmol, 95 %). ^1^H‐NMR (C_6_D_6_, 500 MHz): *δ* =5.46 (t, 1H, *J*=7.2 Hz), 4.79 (m, 2H, overlapped with the signal of D_2_O), 4.74 (m, 2H), 4.47 (t, 2H, *J*=6.6 Hz), 2.04 (m, 8H), 1.71 (s, 6H), 1.57 (m, 4H) ppm. ^13^C‐NMR (C_6_D_6_, 125 MHz): *δ* =151.40 (C_q_), 147.63 (C_q_), 143.00 (C_q_), 119.86 (d, CH, ^3^ *J*_C,P_=8.7 Hz), 109.56 (CH_2_), 108.69 (CH_2_), 62.63 (d, CH_2_, ^2^ *J*_C,P_=5.2 Hz), 38.76 (CH_2_), 36.98 (CH_2_), 35.17 (CH_2_), 35.09 (CH_2_), 25.39 (CH_2_), 25.27 (CH_2_), 21.74 (CH_3_), 15.65 (CH_3_) ppm. ^31^P‐NMR (D_2_O, 203 MHz): *δ* =−7.41 (d, ^2^ *J*_P,P_=21.5 Hz), −10.30 (d, ^2^ *J*_P,P_=21.0 Hz) ppm. HR‐MS (APCI): calc. for [C_15_H_27_O_7_P_2_]^−^ *m*/*z*=381.1237; found: *m*/*z*=381.1238.

### Synthesis of *iso*‐GGPP VII

**Synthesis of 12‐iodo‐2‐methyl‐6,10‐dimethylenedodec‐1‐ene (35)**. A solution of imidazole (1.29 g, 18.9 mmol, 1.20 eq) and PPh_3_ (4.96 g, 18.9 mmol, 1.20 eq) in CH_2_Cl_2_ (45 mL) was mixed with I_2_ (4.78 g, 18.9 mmol, 1.20 eq) and the corresponding alcohol **34** (3.49 g, 15.7 mmol, 1.00 eq) subsequently. After stirring for 2 h at room temperature, the reaction mixture was quenched by the addition of sat. aqueous NH_4_Cl and then extracted with Et_2_O (150 mL) three times. The combined organic layers were dried with MgSO_4_ and concentrated under reduced pressure. The residue was taken up in petroleum ether, the precipitate was filtered off, and the petroleum ether was removed under reduced pressure. The product was purified by column chromatography (petroleum ether) to yield the iodide **35** (3.75 g, 11.2 mmol, 71 %) as a pink oil. TLC (pentane): *R*_f_=0.70. GC (HP‐5): *I*=1871. ^1^H‐NMR (C_6_D_6_, 500 MHz): *δ* =4.82 (m, 4H), 4.77 (m, 1H), 4.62 (m, 1H), 2.83 (t, 2H, *J*=7.6 Hz), 2.28 (td, 2H, *J*=7.6, 1.4 Hz), 1.93 (m, 6H), 1.76 (m, 2H), 1.65 (br s, 3H), 1.56 (m, 2H), 1.42 (m, 2H) ppm. ^13^C‐NMR (C_6_D_6_, 125 MHz): *δ* =149.27 (C_q_), 147.90 (C_q_), 145.61 (C_q_), 111.34 (CH_2_), 110.55 (CH_2_), 109.68 (CH_2_), 40.53 (CH_2_), 37.75 (CH_2_), 35.90 (CH_2_), 35.85 (CH_2_), 35.18 (CH_2_), 26.08 (CH_2_), 25.93 (CH_2_), 22.48 (CH_3_), 3.26 (CH_2_) ppm. IR (diamond ATR): ṽ=3070 (w), 2966 (m), 2933 (s), 2855 (m), 1743 (m), 1636 (m), 1454 (m), 1373 (m), 1216 (w), 1140 (w), 880 (s) cm^−1^. HR‐MS (Q‐TOF, 70 eV): calc. [C_15_H_25_I]^+^⋅ *m*/*z*=332.0996, found: *m*/*z*=332.0992.

**Synthesis of ethyl 2‐acetyl‐13‐methyl‐5,9‐dimethylenetetradec‐13‐enoate (36)**. To a cooled solution of ethyl acetoacetate (2.92 g, 22.4 mmol, 2.00 eq) in THF (40 mL) was added NaH (537 mg, 22.4 mmol, 2.00 eq, 60 % in mineral oil) in small portions. The reaction mixture was allowed to warm to room temperature and stirred for 1 h. The corresponding iodide **35** (3.75 g, 11.2 mmol, 1.00 eq) was added dropwise and the reaction mixture was stirred under reflux overnight, followed by cooling to room temperature. The cooled mixture was quenched by the addition of sat. aqueous NH_4_Cl solution and then extracted with Et_2_O (150 mL) three times. The combined organic layers were dried with MgSO_4_ and concentrated under reduced pressure. The residue was subjected to column chromatography (petroleum ether/EtoAc, 10 : 1) to yield **36** (3.55 g, 10.6 mmol, 95 %) as a colourless oil. TLC (pentane:Et_2_O=5 : 1): *R*_f_=0.47. GC (HP‐5): *I*=2209. ^1^H‐NMR (C_6_D_6_, 500 MHz): *δ* =4.82 (m, 6H), 3.89 (qd, 2H, *J*=7.1, 1.8 Hz), 3.28 (ddd, 1H, *J*=7.8, 4.6, 1.9 Hz), 1.99 (m, 12H), 1.88 (s, 3H), 1.65 (m, 3H), 1.57 (m, 4H), 0.88 (t, 3H, *J*=7.2 Hz) ppm. ^13^C‐NMR (C_6_D_6_, 125 MHz): *δ* =201.42 (C_q_), 169.68 (C_q_), 149.44 (C_q_), 148.50 (C_q_), 145.67 (C_q_), 110.50 (CH_2_), 110.49 (CH_2_), 109.62 (CH_2_), 61.06 (CH_2_), 59.25 (CH), 37.77 (CH_2_), 35.97 (CH_2_), 35.93 (CH_2_), 35.68 (CH_2_), 33.97 (CH_2_), 28.56 (CH_3_), 26.54 (CH_2_), 26.14 (CH_2_), 26.11 (CH_2_), 22.47 (CH_3_), 14.04 (CH_3_) ppm. IR (diamond ATR): ṽ=3068 (w), 2967 (m), 2933 (s), 2859 (m), 1746 (m), 1630 (m), 1444 (m), 1372 (m), 1229 (m), 1169 (w), 880 (s) cm^−1^. HR‐MS (Q‐TOF, 70 eV): [C_21_H_34_O_3_]^+^⋅ *m*/*z*=334.2503, found: *m*/*z*=334.2499.

**Synthesis of 14‐methyl‐6,10‐dimethylenepentadec‐14‐en‐2‐one (37)**. To a solution of β‐keto ester **36** (3.55 g, 10.6 mol, 1.00 eq) in EtOH (40 mL) was added an aqueous solution of KOH (1.78 g dissolved in 10 mL of water, 35.0 mmol, 3.00 eq) and the reaction mixture was stirred under reflux for 3 h before cooling to room temperature. The reaction mixture was slowly acidified with 2 m HCl solution until CO_2_ developed. The resulting mixture was extracted with Et_2_O (150 mL) three times. The combined organic layers were dried with MgSO_4_ and concentrated under reduced pressure. The residue was subjected to column chromatography (petroleum ether/EtOAc, 13 : 1) to yield the desired methyl ketone **37** (2.31 g, 8.8 mmol, 83 %) as a colourless oil. TLC (pentane:Et_2_O=8 : 1): *R*_f_=0.40. GC (HP‐5): *I*=1894. ^1^H‐NMR (C_6_D_6_, 500 MHz): *δ* =4.82 (m, 6H), 1.95 (m, 12H), 1.65 (m, 8H), 1.58 (m, 4H) ppm. ^13^C‐NMR (C_6_D_6_, 125 MHz): *δ* =206.00 (C_q_), 149.51 (C_q_), 149.13 (C_q_), 145.67 (C_q_), 110.49 (CH_2_), 109.90 (CH_2_), 109.60 (CH_2_), 42.64 (CH_2_), 37.77 (CH_2_), 36.04 (CH_2_), 35.95 (CH_2_), 35.81 (CH_2_), 35.62 (CH_2_), 29.41 (CH_3_), 26.23 (CH_2_), 26.12 (CH_2_), 22.47 (CH_3_), 21.96 (CH_2_) ppm. IR (diamond ATR): ṽ=3073 (w), 2934 (s), 2865 (m), 1716 (s), 1644 (m), 1442 (m), 1364 (m), 1158 (w), 884 (s) cm^−1^. HR‐MS (Q‐TOF, 70 eV): calc. [C_18_H_30_O]^+^⋅ *m*/*z*=262.2292, found: *m*/*z*=262.2290.

**Synthesis of ethyl (*E*)‐3,15‐dimethyl‐7,11‐dimethylenehexadeca‐2,15‐dienoate (38)**. NaH (704 mg, 17.6 mmol, 2.00 eq, 60 % in mineral oil) suspended in dry THF (30 mL) was cooled to 0 °C. Triethyl phosphonoacetate (4.34 g, 19.4 mmol, 2.20 eq) was added. After stirring the reaction mixture for 1 h at 0 °C, the reaction mixture was cooled to −78 °C and the methyl ketone **37** (2.31 g, 8.8 mmol, 1.00 eq) was added. The reaction mixture was allowed to warm to room temperature and stirred overnight. Water (15 mL) was added to quench the reaction. The aqueous phase was extracted with Et_2_O (100 mL, 3 times), and the combined organic layers were dried with MgSO_4_ and concentrated under reduced pressure. Purification by column chromatography (petroleum ether/EtOAc, 15 : 1) yielded pure (*E*)‐**38** and (*Z*)‐**38** as colourless oils.

(*E*)‐**38**. Yield: 1.97 g (5.9 mmol, 67 %) TLC (pentane:Et_2_O=8 : 1): *R*_f_=0.65. GC (HP‐5): *I*=2305. ^1^H‐NMR (C_6_D_6_, 500 MHz): *δ* =5.82 (q, 1H, *J*=1.3 Hz), 4.81 (m, 6H), 4.06 (q, 2H, *J*=7.1 Hz), 2.20 (d, 3H, *J*=1.4 Hz), 1.97 (m, 8H), 1.84 (m, 4H), 1.65 (t, 3H, *J*=1.2 Hz), 1.57 (m, 4H), 1.42 (m, 2H), 1.02 (t, 3H, *J*=7.1 Hz) ppm. ^13^C‐NMR (C_6_D_6_, 125 MHz): *δ* =166.46 (C_q_), 159.51 (C_q_), 149.47 (C_q_), 148.97 (C_q_), 145.63 (C_q_), 116.39 (CH), 110.52 (CH_2_), 109.83 (CH_2_), 109.64 (CH_2_), 59.41 (CH_2_), 40.52 (CH_2_), 37.77 (CH_2_), 36.02 (CH_2_), 35.95 (CH_2_), 35.91 (CH_2_), 35.71 (CH_2_), 26.23 (CH_2_), 26.11 (CH_2_), 25.68 (CH_2_), 22.46 (CH_3_), 18.73 (CH_3_), 14.46 (CH_3_) ppm. IR (diamond ATR): ṽ=3073 (w), 2979 (m), 2935 (s), 2872 (w), 1716 (s), 1647 (s), 1443 (w), 1368 (w), 1272 (s), 1221 (s), 1143 (s), 1040 (w), 886 (s), 858 (w) cm^−1^. HR‐MS (Q‐TOF, 70 eV): calc. [C_22_H_36_O_2_]^+^⋅ *m*/*z*=332.2710, found: *m*/*z*=332.2700.

(*Z*)‐**38**. Yield: 558 mg (1.7 mmol, 19 %) TLC (pentane:Et_2_O=8 : 1): *R*_f_=0.73. GC (HP‐5): *I*=2292. ^1^H‐NMR (C_6_D_6_, 500 MHz): *δ* =5.75 (q, 1H, *J*=1.0 Hz), 4.84 (m, 6H), 4.03 (q, 2H, *J*=7.2 Hz), 2.73 (m, 2H), 2.11 (m, 2H), 2.00 (m, 8H), 1.61 (m, 9H), 1.53 (d, 3H, *J*=1.4 Hz), 1.01 (t, 3H, *J*=7.1 Hz) ppm. ^13^C‐NMR (C_6_D_6_, 125 MHz): *δ* =166.03 (C_q_), 160.05 (C_q_), 149.56 (C_q_), 149.46 (C_q_), 145.69 (C_q_), 116.93 (CH), 110.47 (CH_2_), 109.61 (CH_2_), 109.56 (CH_2_), 59.39 (CH_2_), 37.78 (CH_2_), 36.49 (CH_2_), 36.07 (CH_2_), 36.04 (CH_2_), 35.97 (CH_2_), 33.40 (CH_2_), 26.82 (CH_2_), 26.27 (CH_2_), 26.12 (CH_2_), 24.89 (CH_3_), 22.47 (CH_3_), 14.43 (CH_3_) ppm. IR (diamond ATR): ṽ=3073 (w), 2978 (m), 2935 (s), 2862 (m), 1716 (s), 1647 (s), 1444 (m), 1375 (m), 1227 (m), 1154 (s), 1070 (w), 1037 (w), 886 (s), 858 (w) cm^−1^. HR‐MS (Q‐TOF, 70 eV): calc. [C_22_H_36_O_2_]^+^⋅ *m*/*z*=332.2710, found: *m*/*z*=332.2715.

**Synthesis of (*E*)‐3,15‐dimethyl‐7,11‐dimethylenehexadeca‐2,15‐dien‐1‐ol (39)**. To a cooled (0 °C) solution of the ester (*E*)‐**38** (1.97 g, 5.9 mmol, 1.00 eq) in THF (10 mL) was added DIBAL−H (11.9 mL, 11.9 mmol, 1.0 m in hexane, 2.00 eq) and the reaction mixture was stirred for 1 h at room temperature. The mixture was cooled to 0 °C and a saturated solution of Na−K‐tartrate (15 mL) was added. The resulting slurry was stirred for 2 h to dissolve the precipitate and the aqueous phase was extracted with Et_2_O (100 mL, 3 times). The organic layers were dried with MgSO_4_ and concentrated under reduced pressure. The residue was purified by column chromatography (petroleum ether/Et_2_O, 3 : 1) to yield the alcohol **39** (1.24 g, 4.3 mmol, 73 %) as a colourless oil. TLC (pentane:Et_2_O=2 : 1): *R*_f_=0.30. GC (HP‐5): *I*=2176. ^1^H‐NMR (C_6_D_6_, 500 MHz): *δ* =5.40 (ddq, 1H, *J*=7.9, 6.7, 1.4 Hz), 4.86 (m, 4H), 4.80 (m, 2H), 3.99 (d, 2H, *J*=6.7 Hz), 1.96 (m, 12 H), 1.64 (s, 3H), 1.57 (m, 6H), 1.47 (d, 3H, *J*=1.4 Hz) ppm. ^13^C‐NMR (C_6_D_6_, 125 MHz): *δ* =149.53 (C_q_), 149.52 (C_q_), 145.66 (C_q_), 138.14 (C_q_), 125.04 (CH), 110.50 (CH_2_), 109.60 (2x CH_2_), 59.36 (CH_2_), 39.48 (CH_2_), 37.77 (CH_2_), 36.08 (CH_2_), 36.07 (CH_2_), 35.98 (CH_2_), 35.96 (CH_2_), 26.31 (CH_2_), 26.21 (CH_2_), 26.11 (CH_2_), 22.47 (CH_3_), 16.12 (CH_3_) ppm. IR (diamond ATR): ṽ=3358 (br), 3073 (w), 2965 (m), 2933 (s), 2860 (m), 1727 (w), 1667 (w), 1656 (m), 1444 (m), 1375 (w), 1302 (w), 1021 (m), 886 (s) cm^−1^. HR‐MS (Q‐TOF, 70 eV): calc. [C_20_H_34_O]^+^⋅ *m*/*z*=290.2605, found: *m*/*z*=290.2600.

**Synthesis of (*E*)‐3,15‐dimethyl‐7,11‐dimethylenehexadeca‐2,15‐dien‐1‐yl diphosphate (40)**. To a solution of tris(tetra‐n‐butylammonium)hydrogen diphosphate (4.53 g, 5.0 mmol, 1.20 eq) in acetonitrile (12 mL) a solution of the allyl bromide (made from 1.00 eq of alcohol **39** following the above mentioned general procedure) was added and the mixture was stirred at room temperature overnight. Acetonitrile was removed under reduced pressure. The residue was dissolved in aqueous NH_4_HCO_3_ solution (1.0 mL, 0.25 m) and loaded onto a DOWEX 50WX8 ion‐exchange column (NH_4_^+^ form, pH 7.0). The column was flushed slowly with 1.5 volumes of NH_4_HCO_3_ buffer (25 mm, 5 % iPrOH) and the eluate was lyophilized to yield the diphosphate **40** as a colourless hygroscopic powder (3.1 g, 6.2 mmol, 100 %). ^1^H‐NMR (C_6_D_6_, 500 MHz): *δ* =5.41 (t, 1H, *J*=6.7 Hz), 4.70 (m, 6H), 4.47 (m, 2H), 1.98 (m, 12H), 1.68 (s, 3H), 1.55 (m, 6H), 1.38 (m, 3H) ppm. ^13^C‐NMR (C_6_D_6_, 125 MHz): *δ* =149.12 (C_q_), 148.83 (C_q_), 144.87 (C_q_), 140.47 (C_q_), 120.90 (d, CH, ^3^ *J*_C,P_=9.2 Hz), 110.05 (CH_2_), 109.03 (CH_2_), 108.81 (CH_2_), 62.46 (d, ^2^ *J*_C,P_=3.8 Hz), 39.28 (CH_2_), 37.30 (CH_2_), 35.78 (CH_2_), 35.99 (CH_2_), 35.74 (CH_2_), 35.53 (CH_2_),25.85 (CH_2_), 25.71 (CH_2_), 25.56 (CH_2_), 22.13 (CH_3_), 15.87 (CH_3_) ppm. ^31^P‐NMR (D_2_O, 203 MHz): *δ* =−9.75 (d, ^2^ *J*_P,P_=19.8 Hz), −10.94 (d, ^2^ *J*_P,P_=19.1 Hz) ppm. HR‐MS (APCI): calc. for [C_20_H_35_O_7_P_2_]^−^ m/z=449.1863; found: *m*/*z*=449.1869.

**Strains and culture conditions**. *Streptomyces iakyrus* DSM 40482 and *Streptomyces exfoliatus* DSM 41693 were obtained from the Leibniz Institute DSMZ – German Collection of Microorganisms and Cell Cultures GmbH. Both strains were cultivated in 65 medium (4.0 g glucose, 4.0 g yeast extract, 10.0 g malt extract dissolved in 1 L of distilled water, pH 7.2) at 28 °C. For agar plates CaCO_3_ (2.0 g L^−1^) and agar (20.0 g L^−1^) were added to the medium. *Saccharomyces cerevisiae* FY834 was grown on SM‐URA agar (425 mg yeast nitrogen base, 1.25 g ammonium sulfate, 5 g glucose, 192.5 mg nutritional supplement minus uracil (Carl Roth GmbH, Karlsruhe, Germany), 5 g agar, 250 mL water).

**Buffers**. The buffers used for protein purification and incubation experiments were: binding buffer (20 mm Na_2_HPO_4_, 500 mm NaCl, 20 mm imidazole, 1 mm MgCl_2_, pH=7.4), washing buffer (20 mm Na_2_HPO_4_, 500 mm NaCl, 100 mm imidazole, 1 mm MgCl_2_, pH=7.4), elution buffer (20 mm Na_2_HPO_4_, 500 mm NaCl, 500 mm imidazole, 1 mm MgCl_2_, pH=7.4), incubation buffer (50 mm Tris/HCl, 10 mm MgCl_2_, 10 % glycerol, 20 mm β‐cyclodextrin, pH=8.2) and substrate buffer (25 mm aq. NH_4_HCO_3_).

**Gene cloning**. The target genes coding for Bnd4 (accession number WP_033312626) from *S. iakyrus* DSM 40482 and VenA (WP_024756998) from *S. exfoliatus* DSM 41693 were amplified from gDNA by PCR using Q5 High‐fidelity DNA polymerase (New England Biolabs, Ipswich, MA, USA) and the primer pairs (homology arms are in bold and underlined; Bnd4‐Fw: **GGCAGCCATATGGCTAGCATGACTGGTGGA** GTGATCA‐CCGACGCCGATCT; Bnd4‐Rv: **TCTCAGTGGTGGTGGTGGTGGTGCT‐CGAGTG** TCAAACCAGCGGTCGGGTGG; VenA−Fw: **GGCAGCCATAT‐GGCTAGCATGACTGGTGGA** ATGACGACCATCCCGAAGCC; VenA−Rv: **TCTCAGTGGTGGTGGTGGTGGTGCTCGAGTG** TCACGCGGGCA‐CCTCCGTGC). Yeast homologous recombination of the PCR products with the linearised (BamHI and HindIII digestion) pYE‐Express shuttle vector^[51]^ was carried out through the standard protocol using LiOAc, polyethylene glycol and salmon sperm DNA.^[52]^ After transformation of *Saccharomyces cerevisiae* FY834 cultures were grown on SM‐URA agar at 28 °C for 3 days. The recombinant plasmids were isolated from grown yeast colonies using the Zymoprep Yeast Plasmid Miniprep II kit (Zymo Research, Irvine, CA, USA) and subsequently used for transformation of *Escherichia coli* BL21 (DE3) electrocompetent cells. Cells were plated on LB agar plates amended with kanamycin sulfate (50 μg mL^−1^) followed by incubation at 37 °C overnight. Single colonies were selected and used to inoculate LB medium (6 mL) liquid cultures with kanamycin sulfate (6 μL; 50 mg mL^−1^). After 24 h growth plasmid DNA was isolated and checked for correct insertion of the desired gene by PCR amplification of the inserted DNA sequence using the T7 primer pair and by sequencing. The obtained plasmids were named pYE‐Bnd4 and pYE‐VenA.

**Gene expression and protein purification**. Small scale cultures of *Escherichia coli* BL21 (DE3) transformed with the plasmid pYE‐Bnd4 or pYE‐VenA were grown in LB medium (10 mL) containing kanamycin sulfate (50 mg L^−1^) overnight with shaking at 37 °C. A large scale expression culture in LB medium (1 L) containing kanamycin sulfate was inoculated with the grown overnight culture (2 %). Culturing was continued with shaking at 37 °C until an OD_600_=0.4–0.6 was reached. After cooling the culture to 18 °C, enzyme expression was induced by the addition of IPTG solution (100 mm, 1 ‰). The cultures were shaken at 18 °C for 18 h. Cells were harvested via centrifugation (1,500 g, 40 min, 4 °C), resuspended in binding buffer (30 mL, 4 °C) and lysed by ultrasonication (10x 1 min) on ice. The cell debris was removed by centrifugation (14,600 g, 10 min, 4 °C) and the supernatant was loaded on a Ni^2+^‐NTA superflow affinity chromatography column (Qiagen, Venlo, Netherlands) equilibrated with binding buffer. The column was washed with binding buffer (2 column volumes, CV, 4 °C) and washing buffer (2 CV, 4 °C). The desired protein was eluted with elution buffer (1 CV, 4 °C). Protein purity was checked by SDS‐PAGE analysis (Figure S1) and protein concentrations were determined through Bradford assay.^[53]^

**Preparative scale incubation of substrate analogs with recombinant diterpene synthases and compound isolation**. For preparative scale enzymatic conversions the enzyme‐substrate combinations as detailed in Table S1 were used (for expression and purification of the enzymes cf. the references given in Table S1).

**Compounds from** ***iso*** **‐GGPP I**. A solution of the trisammonium salt of *iso*‐GGPP I (60 mg, 120 μmol) in substrate buffer (20 mL) was added to incubation buffer (130 mL) containing Bnd4 or VenA (0.3 mg L^−1^), followed by incubation for 16 h at 30 °C. The reaction mixtures were extracted with n‐hexane, the extracts were dried with MgSO_4_ and the solvent was evaporated, followed by compound isolation through column chromatography on silica gel to yield the pure compounds **45** and **47**.

**Compounds from** ***iso*** **‐GGPP IV**. A solution of the trisammonium salt of *iso*‐GGPP IV (60 mg, 120 μmol for HdS; 80 mg, 160 μmol for AbVS) in substrate buffer (10 mL) was added to incubation buffer (105 mL for HdS; 80 mL for AbVS) containing HdS (0.52 mg L^−1^, containing 60 mg of protein in total) or AbVS (0.35 mg L^−1^, 40 mg), followed by incubation for 16 h at 30 °C. The reaction mixtures were extracted with n‐hexane, the extracts were dried with MgSO_4_ and the solvent was evaporated, followed by compound isolation through column chromatography on silica gel to give compounds **48** and **49**.

**Compounds from** ***iso*** **‐GGPP VI**. A solution of the trisammonium salts of *iso*‐FPP III (70 mg, 162 μmol) and IPP (60 mg, 203 μmol) (as precursors of *iso*‐GGPP VI using GGPP synthase, GGPPS) in substrate buffer (10 mL) was added to incubation buffer (65 mL) containing SoS (1.06 mg L^−1^, 80 mg) and GGPPS (0.86 mg L^−1^, 60 mg) followed by incubation for 16 h at 30 °C. The reaction mixture was extracted with n‐hexane, the extract was dried with MgSO_4_ and the solvent was evaporated, followed by compound isolation through column chromatography on silica gel to give compound **51**.

A solution of the trisammonium salts of *iso*‐FPP III (70 mg, 162 μmol) and IPP (70 mg, 296 μmol) in substrate buffer (10 mL) was added to incubation buffer (70 mL) containing CyS (1.56 mg L^−1^, 125 mg) and GGPPS (0.94 mg L^−1^, 80 mg) followed by incubation for 16 h at 30 °C. The reaction mixture was extracted with n‐hexane, the extract was dried with MgSO_4_ and the solvent was evaporated, followed by compound isolation through column chromatography on silica gel to give compounds **52** and **54**.

A solution of the trisammonium salts of *iso*‐FPP III (90 mg, 208 μmol) and IPP (100 mg, 338 μmol) in substrate buffer (20 mL) was added to incubation buffer (150 mL) containing SvS (0.59 mg L^−1^, 100 mg) and GGPPS (0.47 mg L^−1^, 80 mg) followed by incubation for 16 h at 30 °C. The reaction mixture was extracted with n‐hexane, the extract was dried with MgSO_4_ and the solvent was evaporated, followed by compound isolation through column chromatography on silica gel to give compound **53** and a mixture of **54** and **55**. Compounds **54** and **55** were further separated on AgNO_3_ coated silica gel (for its preparation 50 g of silica gel were suspended in a solution of AgNO_3_ (10.0 g) in methanol (150 mL) overnight, followed by rigorous evaporation of the solvent).

A solution of the trisammonium salts mixture of *iso*‐FPP III (100 mg, 231 μmol) and IPP (100 mg, 338 μmol) in substrate buffer (30 mL) was added to incubation buffer (245 mL) containing *Si*CotB2 (0.54 mg L^−1^, 150 mg) and GGPPS (0.36 mg L^−1^, 100 mg) followed by incubation for 16 h at 30 °C. The reaction mixture was extracted with n‐hexane, the extract was dried with MgSO_4_ and the solvent was evaporated, followed by compound isolation through column chromatography on silica gel. A mixture of **56** and **57** was obtained that was further separated on AgNO_3_ coated silica gel to obtain pure **57**. The purity of **56** was further improved by HPLC.

**Benditerpe‐2,7(19),15‐triene (45)**. Yield: 4.3 mg (15.8 μmol, 13 %). TLC (pentane): *R*_f_=0.60. GC (HP‐5MS): *I*=1928. MS (EI, 70 eV): *m*/*z* (%)=39 (1), 41 (4), 55 (5), 67 (6), 79 (9), 91 (11), 105 (9), 107 (7), 119 (6), 133 (6), 147 (4), 161 (4), 173 (3), 187 (5), 201 (4), 216 (2), 229 (2), 257 (9), 272 (1). IR (diamond ATR): ṽ=3071 (w), 2924 (s), 2867 (s), 1639 (w), 1452 (m), 1375 (w), 884 (s), 846 (w), 805 (w) cm^−1^. HR‐MS (Q‐TOF, 70 eV): calc. [C_20_H_32_]^+^⋅ *m*/*z*=272.2499; found: *m*/*z*=272.2495. Optical rotation: [α]_D_^25^=+24.8 (*c* 0.4, acetone). NMR data are given in Table S7.

**Venezuelaxenene (47)**. Yield: 2.0 mg (7.4 μmol, 6 %). TLC (pentane): *R*_f_=0.84. GC (HP‐5MS): *I*=2001. MS (EI, 70 eV): *m*/*z* (%)=41 (1), 55 (2), 67 (2), 79 (3), 91 (4), 105 (4), 107 (3), 119 (3), 133 (3), 147 (3), 161 (2), 173 (2), 187 (3), 201 (2), 229 (11), 257 (2), 272 (3). IR (diamond ATR): ṽ=2924 (s), 2866 (m), 1731 (w), 1672 (w), 1463 (m), 1373 (m), 1312 (m), 1260 (w), 1228 (m), 1081 (m), 1025 (m), 893 (w), 800 (m) cm^−1^. HR‐MS (Q‐TOF, 70 eV): calc. [C_20_H_32_]^+^⋅ *m*/*z*=272.2499; found: *m*/*z*=272.2503. Optical rotation: [α]_D_^25^=+18.5 (*c* 0.2, CH_2_Cl_2_). NMR data are given in Table S8.

**Diisocembrene A (48)**. Yield: 0.63 mg (2.3 μmol, 1.9 %). TLC (pentane): *R*_f_=0.42. GC (HP‐5MS): *I*=1986. MS (EI, 70 eV): *m*/*z* (%)=39 (2), 41 (6), 43 (1), 67 (8), 79 (9), 81 (10), 93 (8), 107 (6), 121 (5), 133 (4), 159 (1), 175 (1), 189 (1), 229 (1), 257 (1), 272 (1). IR (diamond ATR): ṽ=3070 (w), 2928 (s), 2854 (m), 2180 (w), 1734 (w), 1643 (m), 1439 (m), 1374 (w), 1260 (w), 1093 (w), 1022 (w), 887 (s), 802 (w) cm^−1^. HR‐MS (Q‐TOF, 70 eV): calc. [C_20_H_32_]^+^⋅ *m*/*z*=272.2499; found: *m*/*z*=272.2502. Optical rotation: [α]_D_^25^=+30.0 (*c* 0.06, acetone). NMR data are given in Table S10.

**Prenylisopseudogermacrene B (49)**. Yield: 0.40 mg (1.5 μmol, 0.9 %). TLC (pentane): *R*_f_=0.54. GC (HP‐5MS): *I*=2058. MS (EI, 70 eV): *m*/*z* (%)=41 (18), 53 (5), 67 (9), 69 (14), 79 (7), 93 (22), 107 (5), 109 (2), 121 (3), 135 (4), 147 (2), 161 (2), 175 (1), 187 (1), 203 (1), 229 (1), 257 (1), 272 (1). IR (diamond ATR): ṽ=2958 (br), 2932 (br), 2852 (m), 2315 (s), 1644 (w), 1375 (s), 1259 (w), 1200 (w), 1083 (w), 1021 (w), 884 (w), 789 (m) cm^−1^. HR‐MS (Q‐TOF, 70 eV): calc. [C_20_H_32_]^+^⋅ *m*/*z*=272.2499; found: *m*/*z*=272.2495. NMR data are given in Table S11.

**Isopentenylpseudogermacrene A (51)**. Yield: 4.0 mg (14.7 μmol, 9.1 %). TLC (AgNO_3_ activated TLC plate, pentane:Et_2_O=8 : 1): *R*_f_=0.32. GC (HP‐5MS): *I*=2042. MS (EI, 70 eV): *m*/*z* (%)=39 (3), 41 (13), 55 (7), 67 (10), 69 (8), 79 (12), 93 (11), 107 (9), 109 (3), 121 (4), 135 (2), 147 (3), 161 (3), 175 (2), 190 (2), 203 (4), 229 (1), 257 (1), 272 (1). IR (diamond ATR): ṽ=3368 (w), 2922 (s), 2852 (m), 2268 (w), 1650 (w), 1633 (w), 1443 (w), 1382 (w), 1259 (w), 886 (w), 831 (w) cm^−1^. HR‐MS (Q‐TOF, 70 eV): calc. [C_20_H_32_]^+^⋅ *m*/*z*=272.2499; found: *m*/*z*=272.2501. NMR data are given in Table S12.

**Isopentenylpseudogermacrene B (52)**. Yield: 2.0 mg (7.4 μmol, 4.6 %). TLC (pentane): *R*_f_=0.38. GC (HP‐5MS): *I*=2013. MS (EI, 70 eV): *m*/*z* (%)=39 (3), 41 (10), 55 (7), 68 (16), 69 (3), 79 (10), 93 (11), 107 (8), 109 (2), 121 (9), 133 (4), 147 (3), 161 (3), 175 (1), 189 (3), 201 (2), 216 (2), 229 (1), 257 (2), 272 (1). IR (diamond ATR): ṽ=3352 (w), 2924 (s), 2851 (m), 2144 (w), 1991 (w), 1739 (w), 1657 (w), 1632 (w), 1443 (w), 1381 (w), 1259 (w), 1215 (w), 1097 (w), 1081 (w), 1027 (w), 814 (w) cm^−1^. HR‐MS (Q‐TOF, 70 eV): calc. [C_20_H_32_]^+^⋅ *m*/*z*=272.2499; found: *m*/*z*=272.2497. NMR data are given in Table S13.

**Isopentenylpseudogermacrene C (53)**. Yield: 1.3 mg (4.8 μmol, 2.3 %). TLC (pentane): *R*_f_=0.48. GC (HP‐5MS): *I*=2036. MS (EI, 70 eV): *m*/*z* (%)=39 (6), 41 (13), 55 (10), 67 (19), 79 (11), 93 (8), 107 (5), 121 (5), 133 (4), 147 (2), 161 (2), 173 (1), 189 (7), 201 (2), 216 (1), 257 (3), 272 (1). IR (diamond ATR): ṽ=3074 (w), 2917 (s), 2852 (m), 1744 (w), 1649 (w), 1445 (m), 1375 (w), 1259 (w), 1099 (w), 885 (w), 829 (w) cm^−1^. HR‐MS (Q‐TOF, 70 eV): calc. [C_20_H_32_]^+^⋅ *m*/*z*=272.2499; found: *m*/*z*=272.2508. NMR data are given in Table S14.

**α‐Spirocattleyaxenene (54)**. Yield: 0.4 mg (1.5 μmol, 0.9 %). TLC (pentane): *R*_f_=0.48. GC (HP‐5MS): *I*=2118. MS (EI, 70 eV): *m*/*z* (%)=39 (3), 41 (9), 53 (5), 55 (9), 67 (12), 79 (10), 81 (11), 93 (9), 105 (11), 107 (5), 121 (5), 135 (2), 147 (2), 161 (1), 189 (1), 201 (1), 272 (4). IR (diamond ATR): ṽ=2923 (s), 2850 (m), 1739 (w), 1442 (m), 1377 (w), 1258 (m), 1216 (w), 1090 (m), 1014 (m), 924 (w), 885 (w), 794 (s), 702 (w) cm^−1^. HR‐MS (Q‐TOF, 70 eV): calc. [C_20_H_32_]^+^⋅ *m*/*z*=272.2499; found: *m*/*z*=272.2503. Optical rotation: [α]_D_^25^=−20.0 (*c* 0.04, acetone, obtained with CyS); [α]_D_^25^=+7.5 (*c* 0.04, acetone, obtained with SvS). NMR data are given in Table S15.

**β‐Spirocattleyaxenene (55)**. Yield: 0.4 mg (1.5 μmol, 0.7 %). TLC (AgNO_3_ activated TLC plate, pentane:Et_2_O=8 : 1): *R*_f_=0.52. GC (HP‐5MS): *I*=2113. MS (EI, 70 eV): *m*/*z* (%)=39 (2), 41 (7), 53 (4), 55 (6), 67 (9), 79 (11), 81 (10), 93 (8), 105 (5), 107 (6), 121 (8), 135 (3), 147 (3), 161 (2), 189 (2), 201 (3), 216 (1), 229 (1), 243 (1), 257 (1), 272 (4). IR (diamond ATR): ṽ=3361 (w), 2969 (m), 2924 (s), 2851 (w), 1738 (s), 1656 (w), 1438 (w), 1365 (m), 1228 (m), 1216 (m), 1206 (m), 885 (w), 527 (w) cm^−1^. HR‐MS (Q‐TOF, 70 eV): calc. [C_20_H_32_]^+^⋅ *m*/*z*=272.2499; found: *m*/*z*=272.2494. Optical rotation: [α]_D_^25^=+5.0 (*c* 0.06, acetone, obtained with CyS); [α]_D_^25^=−2.5 (*c* 0.04, acetone, obtained with SvS). NMR data are given in Table S16.

**Isobucketwheelene (56)**. Yield: 3.0 mg (11.0 μmol, 4.8 %). TLC (AgNO_3_ activated TLC plate, pentane:Et_2_O=15 : 1): *R*_f_=0.28. GC (HP‐5MS): *I*=2062. MS (EI, 70 eV): *m*/*z* (%)=39 (3), 41 (9), 53 (6), 55 (7), 67 (10), 79 (12), 81 (13), 93 (9), 105 (4), 107 (6), 121 (6), 133 (5), 147 (2), 161 (2), 175 (1), 189 (1), 201 (1), 257 (1), 272 (1). IR (diamond ATR): ṽ=3071 (w), 2978 (m), 2931 (s), 2849 (m), 1731 (w), 1643 (m), 1439 (m), 1381 (w), 1229 (w), 1217 (w), 1099 (w), 887 (s), 831 (w) cm^−1^. HR‐MS (Q‐TOF, 70 eV): calc. [C_20_H_32_]^+^⋅ *m*/*z*=272.2499; found: *m*/*z*=272.2505. NMR data are given in Table S17.

**Iakyroxenene (57)**. Yield: 1.8 mg (6.6 μmol, 3.3 %). TLC (AgNO_3_ activated TLC plate, pentane:Et_2_O=15 : 1): *R*_f_=0.28. GC (HP‐5MS): *I*=2092. MS (EI, 70 eV): *m*/*z* (%)=39 (2), 41 (6), 53 (4), 55 (5), 67 (9), 79 (10), 81 (7), 91 (7), 93 (9), 108 (8), 121 (14), 135 (3), 147 (2), 161 (2), 175 (3), 189 (3), 201 (1), 215 (1), 229 (1), 243 (1), 257 (2), 272 (2). IR (diamond ATR): ṽ=2925 (s), 2845 (m), 1737 (w), 1441 (w), 1381 (w), 1368 (w), 1229 (w), 1216 (w), 852 (w) cm^−1^. HR‐MS (Q‐TOF, 70 eV): calc. [C_20_H_32_]^+^⋅ *m*/*z*=272.2499; found: *m*/*z*=272.2503. Optical rotation: [α]_D_^25^=+98.0 (*c* 0.3, acetone). NMR data are given in Table S18.

**Conversion of 41 with NBS**. To a solution of **41** (11.3 mg, 41.6 μmol, 1.0 eq) in CH_2_Cl_2_ was added *N*‐bromosuccinimide (7.4 mg, 41.6 μmol, 1.0 eq) at −78 °C under argon atmosphere. After stirring at −78 °C for 20 min, the reaction mixture warmed to room temperature over 1 h and stirring was continued for another 1 h. The reaction was quenched by the addition of sat. NaHCO_3_ soultion and extracted with n‐pentane (3×20 mL). The combined organic layers were dried with MgSO_4_ and concentrated under reduced pressure. The residues was subjected to column chromatography (n‐pentane) to yield **42** (0.7 mg, 2.00 μmol, 5 %), **43** (0.6 mg, 1.71 μmol, 4 %) and **44** (0.3 mg, 0.85 μmol, 2 %).

Bromide **42**. *R*_f_=0.31 (n‐pentane). GC (HP‐5MS): *I*=2473. IR (diamond ATR): ṽ=2924 (s), 2853 (m), 1754 (w), 1655 (w), 1634 (w), 1463 (w), 1385 (w), 1170 (w), 888 (w), 795 (w) cm^−1^. Optical rotation: [α]_D_^25^=−42.5 (*c* 0.03, CH_2_Cl_2_). HRMS (APCI): [M−Br]^+^ calc. for [C_20_H_31_]^+^ *m*/*z*=271.2421, found *m*/*z*=271.2418. NMR data are given in Table S2.

Bromide **43**. TLC (n‐pentane): *R*_f_=0.50. GC (HP‐5MS): *I*=2460. IR (diamond ATR): ṽ=2922 (s), 2853 (s), 1677 (w), 1465 (m), 1441 (m), 1383 (m), 1260 (m), 1090 (m), 1018 (m), 867 (w), 795 (m), 701 (w) cm^−1^. Optical rotation: [α]_D_^25^=+43.3 (*c* 0.06, CH_2_Cl_2_). HRMS (APCI): [M−Br]^+^ calc. for [C_20_H_31_]^+^ *m*/*z*=271.2421, found *m*/*z*=271.2415. NMR data are given in Table S3.

Bromide **44**. *R*_f_=0.42 (n‐pentane). GC (HP‐5MS): *I*=2285. IR (diamond ATR): ṽ=2923 (s), 2853 (m), 2172 (w), 2023 (w), 1720 (w), 1634 (w), 1454 (w), 1259 (w), 1077 (w), 1024 (w), 907 (w), 799 (w) cm^−1^. Optical rotation: [α]_D_^25^=+56.7 (*c* 0.03, CH_2_Cl_2_). HRMS (APCI): [M−Br]^+^ calc. for [C_20_H_31_]^+^ *m*/*z*=271.2421, found *m*/*z*=271.2426. NMR data are given in Table S4.

## Conflict of Interests

The authors declare no conflict of interest.

1

## Supporting information

As a service to our authors and readers, this journal provides supporting information supplied by the authors. Such materials are peer reviewed and may be re‐organized for online delivery, but are not copy‐edited or typeset. Technical support issues arising from supporting information (other than missing files) should be addressed to the authors.

Supporting Information

## Data Availability

The data that support the findings of this study are available in the supplementary material of this article.
