# Supplementary material for: Enzyme‐Catalysed Formation of Hydrocarbon Scaffolds from Geranylgeranyl Diphosphate Analogs with Shifted Double Bonds
Source: Chemistry. 2025 Mar 21;31(23):e202500712. doi: 10.1002/chem.202500712 (PMC12015392; doi:10.1002/chem.202500712)
Supplement: Supplementary file 1 — Supporting Information [file CHEM-31-e202500712-s001.pdf]

Chemistry–A European Journal

Supporting Information

Enzyme-Catalysed Formation of Hydrocarbon Scaffolds from Geranylgeranyl Diphosphate Analogs with Shifted Double Bonds

Heng Li, Bernd Goldfuss, and Jeroen S. Dickschat*

Table of Contents

SDS-PAGE analysis of purified recombinant Bnd4 and VenA	2
Production of diterpenoids by different tested enzyme-substrate combinations	3
GC/MS analysis of the products obtained from GGPP with Bnd4	4
NMR data and structure elucidation of **42**	5
NMR data and structure elucidation of **43**	13
NMR data and structure elucidation of **44**	21
Computational methods	28
Results of DFT calculations for bromination of **41** with NBS	29
Incubation experiments with labelled substrates	31
The 1,3-hydride shift from **B** to **C** in the biosynthesis of **41**	32
GC/MS analysis of the products obtained from *iso*-GGPP I with Bnd4	33
NMR data and structure elucidation of **45**	34
The 1,3-hydride shift from **B2** to **C2** in the biosynthesis of **45**	42
The absolute configuration of **41**	43
GC/MS analysis of the products obtained from GGPP with VenA	44
Two sequential 1,2-hydride shift from **N** to **P** in biosynthesis of **46**	45
GC/MS analysis of the products obtained from *iso*-GGPP I with VenA	47
NMR data and structure elucidation of **47**	48
Stereochemical course for the 1,2-hydride shift in the biosynthesis of **47** by VenA	56
Results of DFT calculations for the cyclisation of *iso*-GGPP I to **47**	57
GC/MS analysis of the products obtained from *iso*-GGPP IV with HdS	59
NMR data and structure elucidation of **48**	60
GC/MS analysis of the products obtained from *iso*-GGPP IV with AbVS	68
NMR data and structure elucidation of **49**	69
GC/MS analysis of the products obtained from *iso*-GGPP VI with diterpene synthases	77
NMR data and structure elucidation of **51**	80
NMR data and structure elucidation of **52**	88
NMR data and structure elucidation of **53**	96
NMR data and structure elucidation of **54**	104
NMR data and structure elucidation of **55**	112
NMR data and structure elucidation of **56**	120
NMR data and structure elucidation of **57**	128
The production of **54** and **55** by diverse diterpene synthases	136
The absolute configuration of **55**	137
The absolute configuration of **57**	138
Cartesian coordinates of computed structures (bromination products)	139
Cartesian coordinates of computed structures (variexenene biosynthesis)	156
References	176

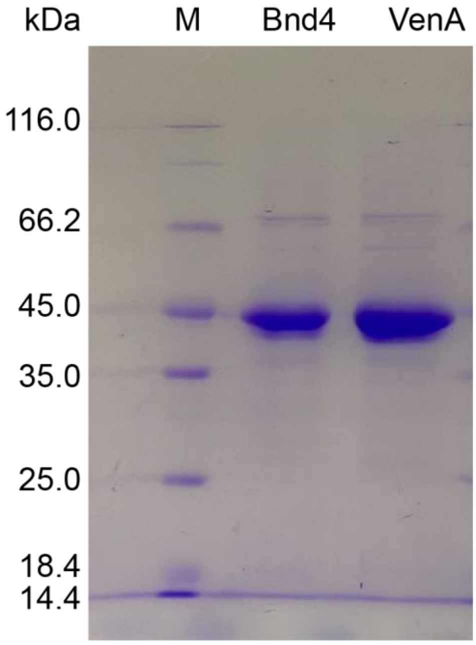

Figure S1. SDS-PAGE analysis of purified recombinant Bnd4 and VenA. M = protein marker.

Table S1. Production of diterpenoids by different tested enzyme-substrate combinations.[a]

enzyme	*iso*-GGPP I	*iso*-GGPP IV	*iso*-GGPP V	*iso*-GGPP VI	*iso*-GGPP VII
AbVS[1]		**49***		**52**, **54**, **55**	
Bnd4[2]	**45***				
CaCS[3]				**51**, **52**, **54**, **55**	
CgDS[4]		**49**		**51**, **52**, **54**, **55**	
CyS[5]				**52***, **53**, **54***, **55**	
HdS[6]		**48***		**52**, **54**, **55**, **56**	
NrPS[5]				**52**, **54**, **55**	
NtPS[5]				**52**, **54**, **55**	
PmS[7]				**51**, **52**, **53**, **54**, **55***, **57**	
SaS[8]				**52**, **54**, **55**, **57**	
*Si*CotB2[9]				**52**, **56***, **57***	
SoS[10]				**51***	
SvS[11]				**52**, **53***, **54***, **55***	
VenA[12]	**47***				

[a] Asterisks indicate enzyme-substrate combinations that were used for compound isolations.

A)

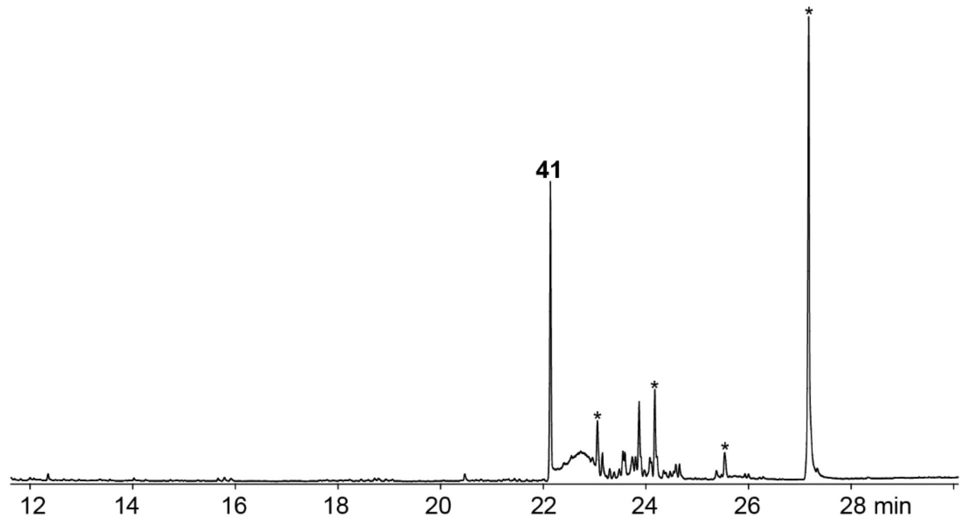

B)

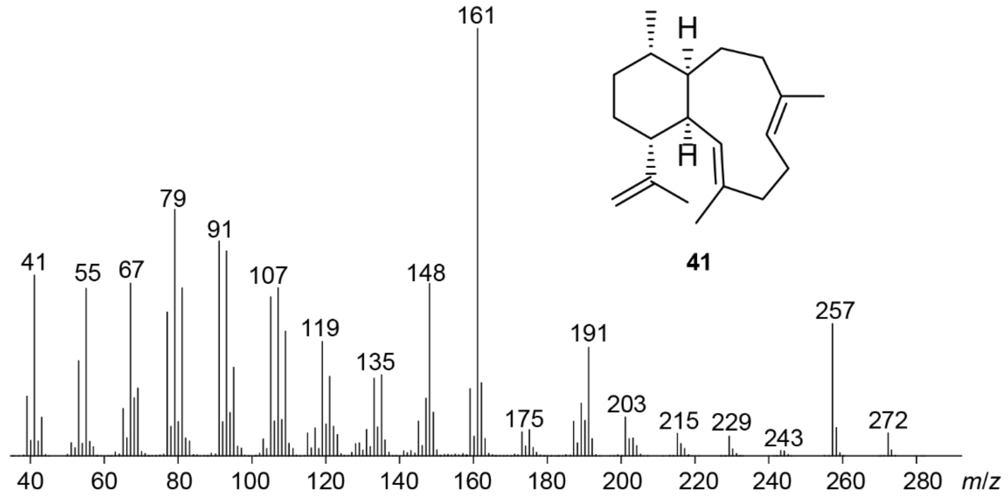

Figure S2. Enzymatic conversion of GGPP with Bnd4. A) Total ion chromatogram of an extract from the incubation of GGPP with Bnd4 from *S. iakyrus*. B) EI mass spectrum of **41**. Asterisks indicate contaminants and spontaneous lysis and hydrolysis products of GGPP.

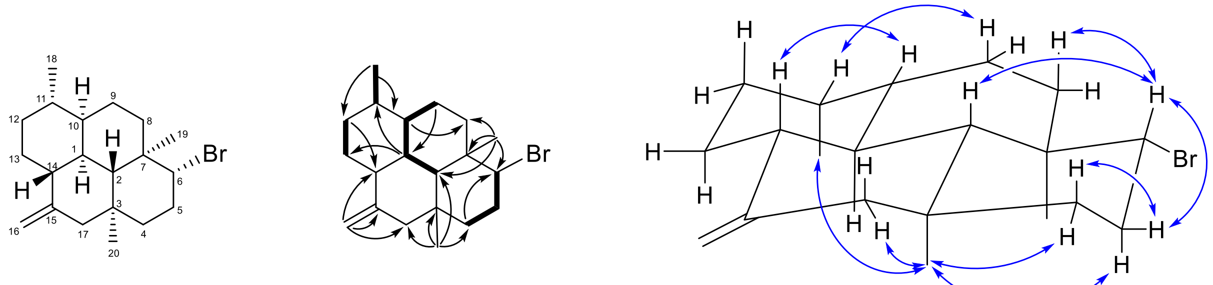

42

Figure S3. Structure elucidation of **42**. Bold: ^{1}H,^{1}H-COSY, single headed arrows: key HMBC, and blue double headed arrows: NOESY correlations.

Table S2. NMR data of **42** in C_6D_6 recorded at 298 K.

C[a]	type	^{13}C[b]	^{1}H[b]
1	CH	36.09	1.47 (m)
2	CH	51.81	0.91 (m)
3	C_q	35.66	–
4	CH_2	43.06	1.15 (m)
			0.89 (m)
5	CH_2	31.50	2.08 (m)
			1.89 (qd, J = 3.6, 13.2)
6	CH-Br	71.65	3.64 (dd, J = 4.0, 12.5)
7	C_q	38.38	–
8	CH_2	42.17	1.51 (m)
			1.46 (m)
9	CH_2	23.28	1.60 (m)
			1.49 (m)
10	CH	38.90	1.23 (m)
11	CH	31.93	1.31 (m)
12	CH_2	29.41	1.50 (m)
			1.17 (m)
13	CH_2	23.97	1.24 (m, 2H)
14	CH	42.09	1.66 (m)
15	C_q	150.37	–
16	CH_2	55.07	1.76 (d, J = 13.0)
			1.66 (m)
17	CH_2	106.91	4.72 (q, J = 1.8)
			4.70 (q, J = 1.8)
18	CH_3	20.96	0.85 (d, J = 6.7)
19	CH_3	21.68	1.08 (s)
20	CH_3	20.28	0.74 (s)

[a] Carbon numbering as shown in Figure S3. [b] Chemical shifts δ in ppm, multiplicity: s = singlet, d = doublet, m = multiplet, q = quartlet, coupling constants J are given in Hertz.

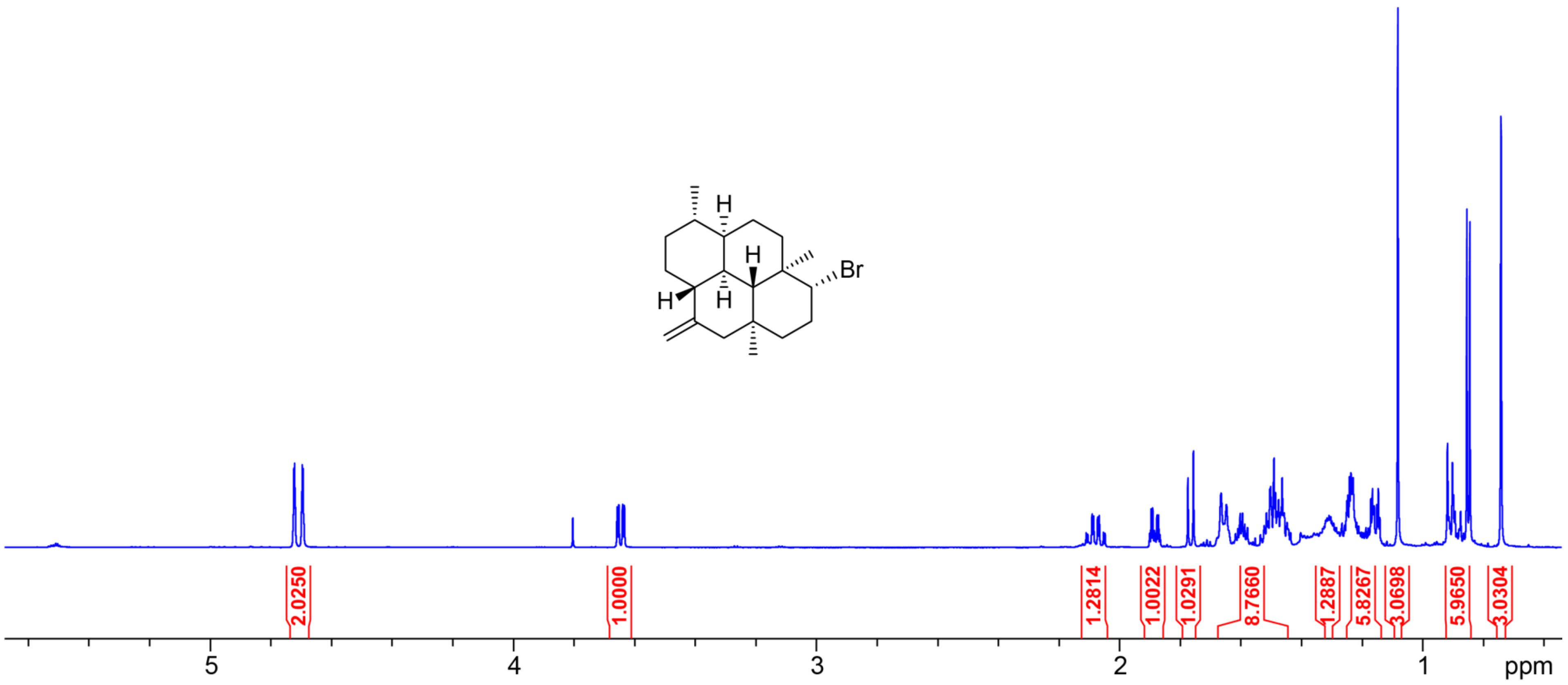

Figure S4. ^{1}H-NMR spectrum of **42** (700 MHz, C_6D_6).

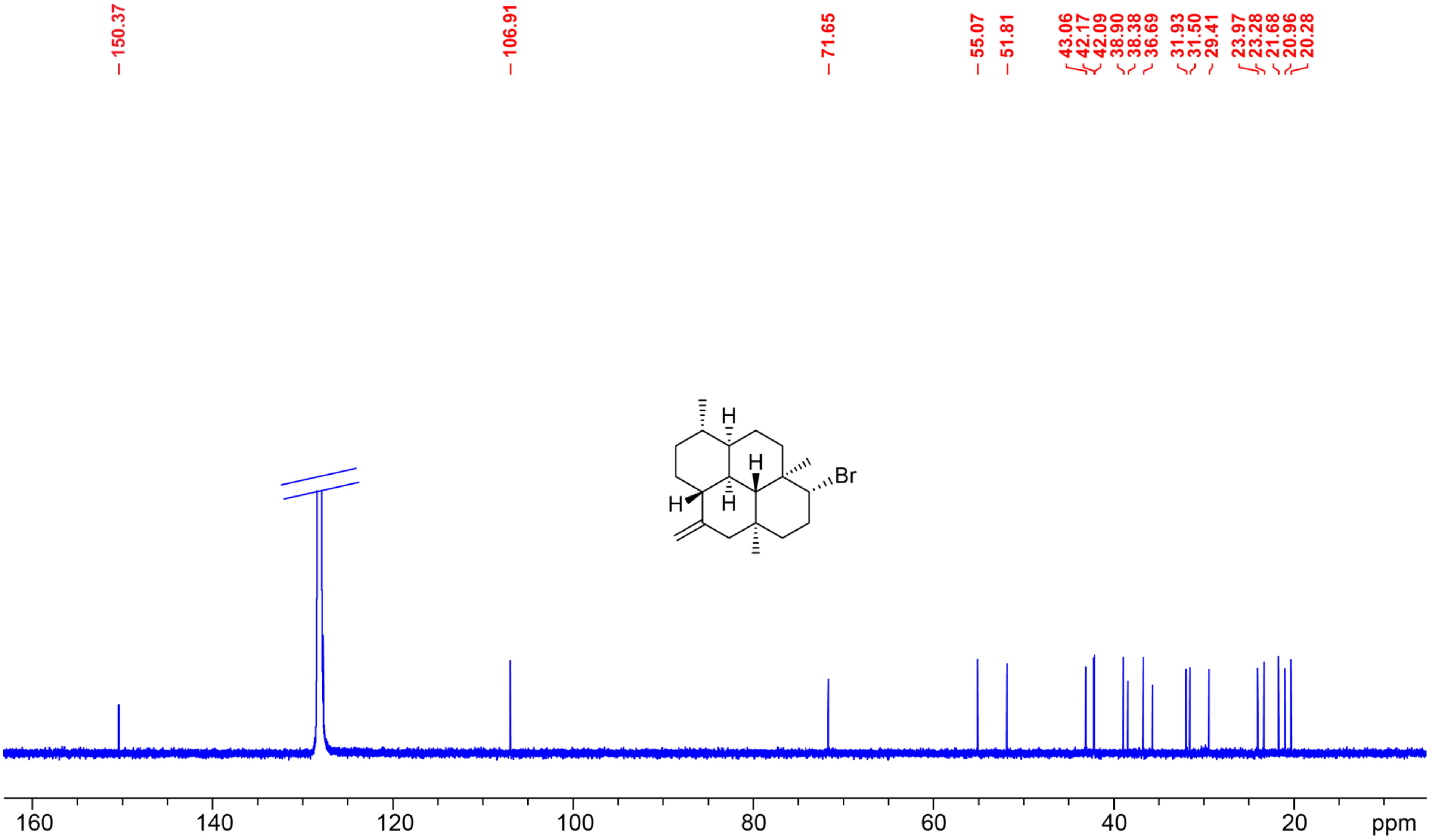

Figure S5. ^{13}C-NMR spectrum of **42** (176 MHz, C_6D_6).

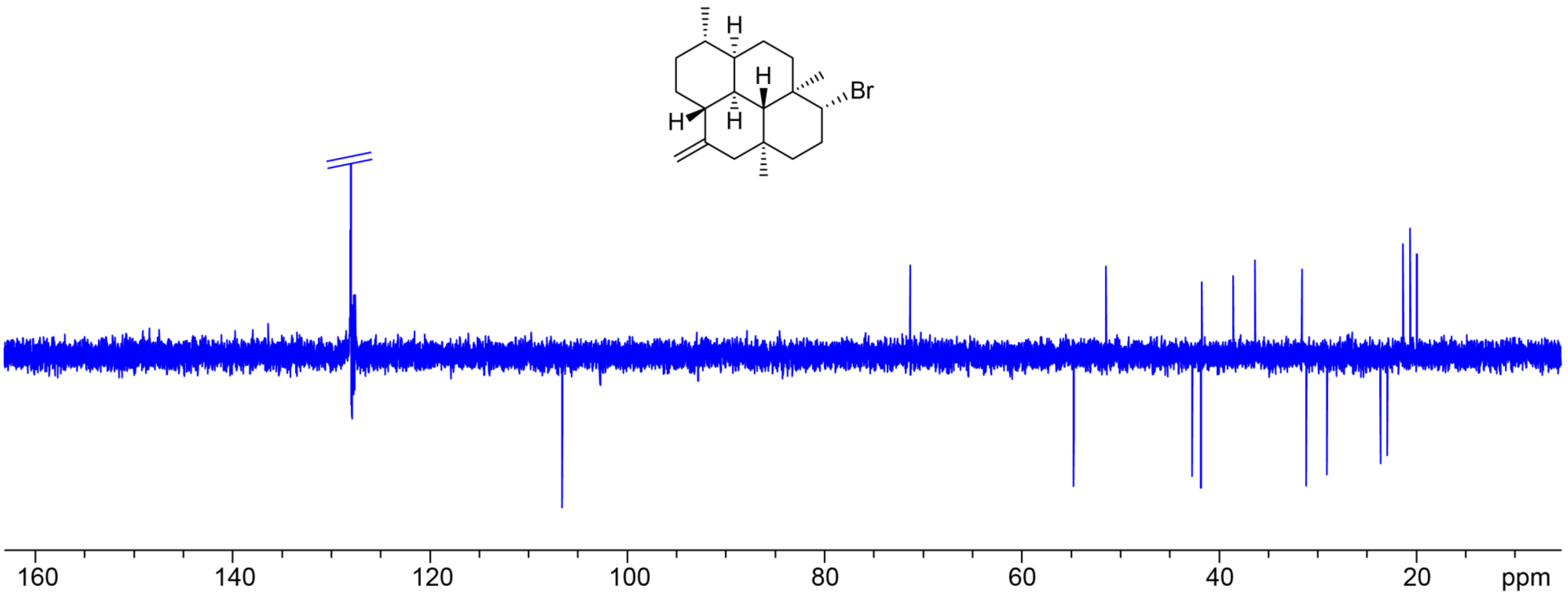

Figure S6. ^{13}C-DEPT135 spectrum of **42** (176 MHz, C_6D_6).

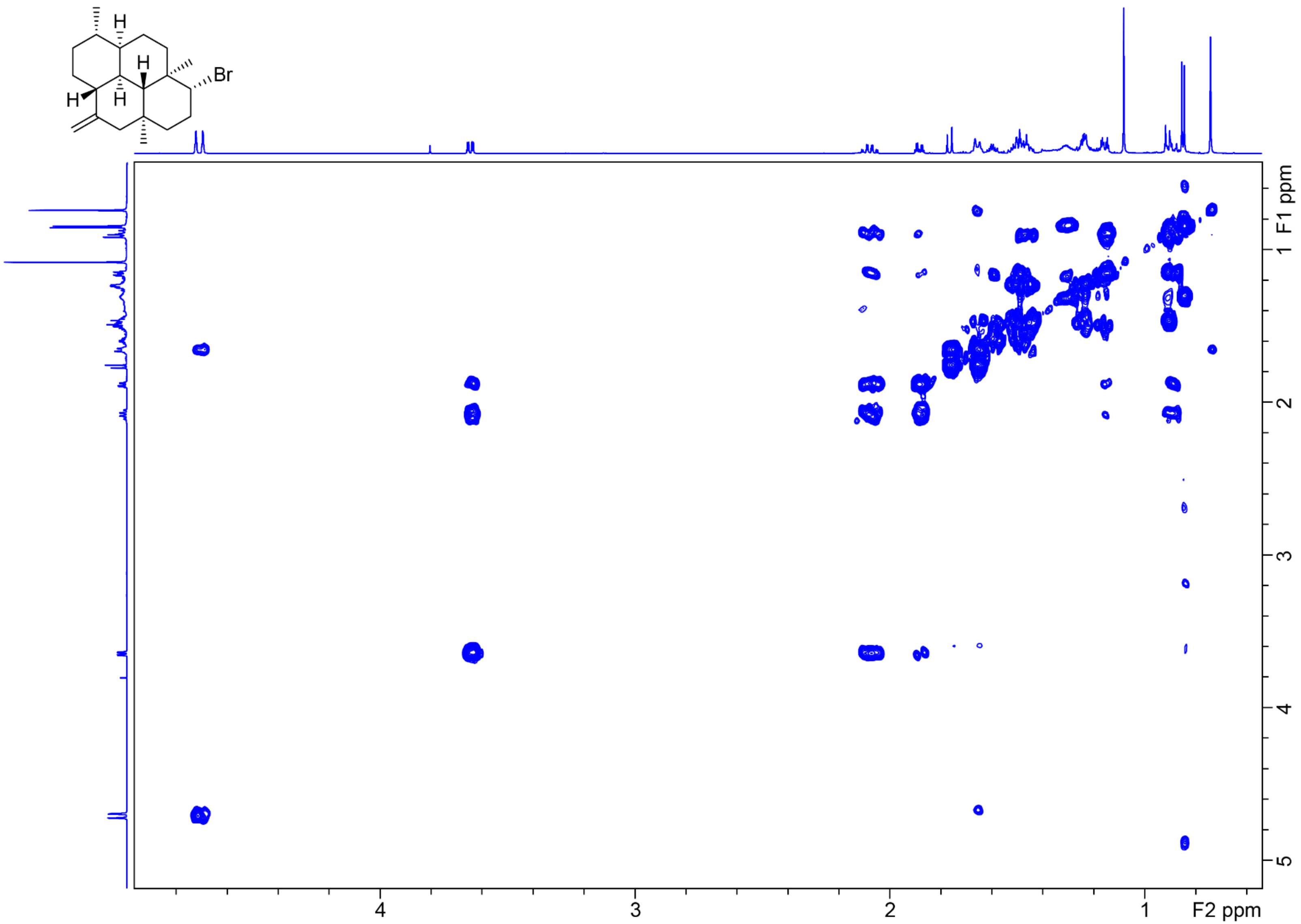

Figure S7. ^{1}H-^{1}H-COSY spectrum (C_6D_6) of **42**.

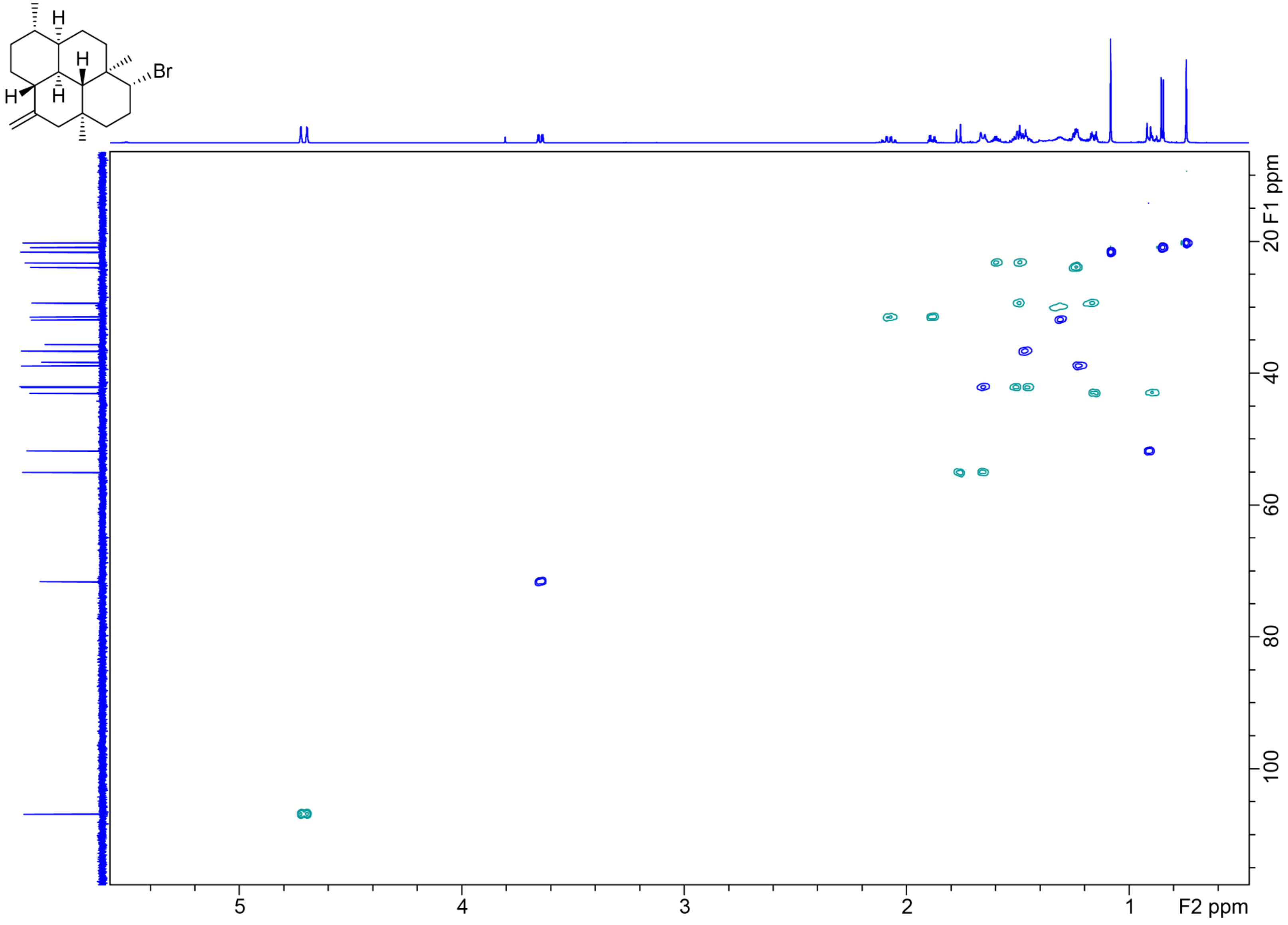

Figure S8. HSQC spectrum (C_6D_6) of **42**.

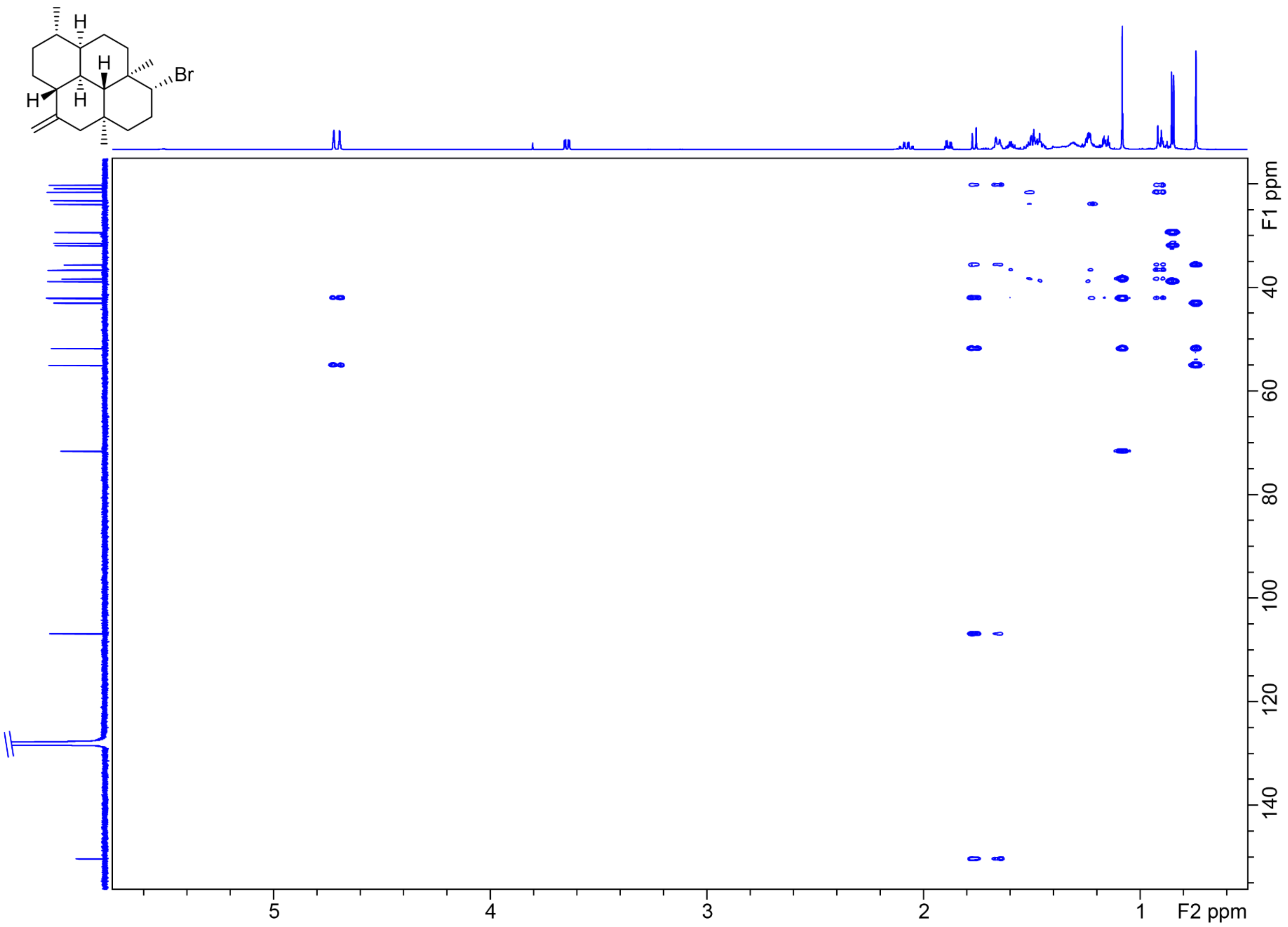

Figure S9. HMBC spectrum (C_6D_6) of **42**.

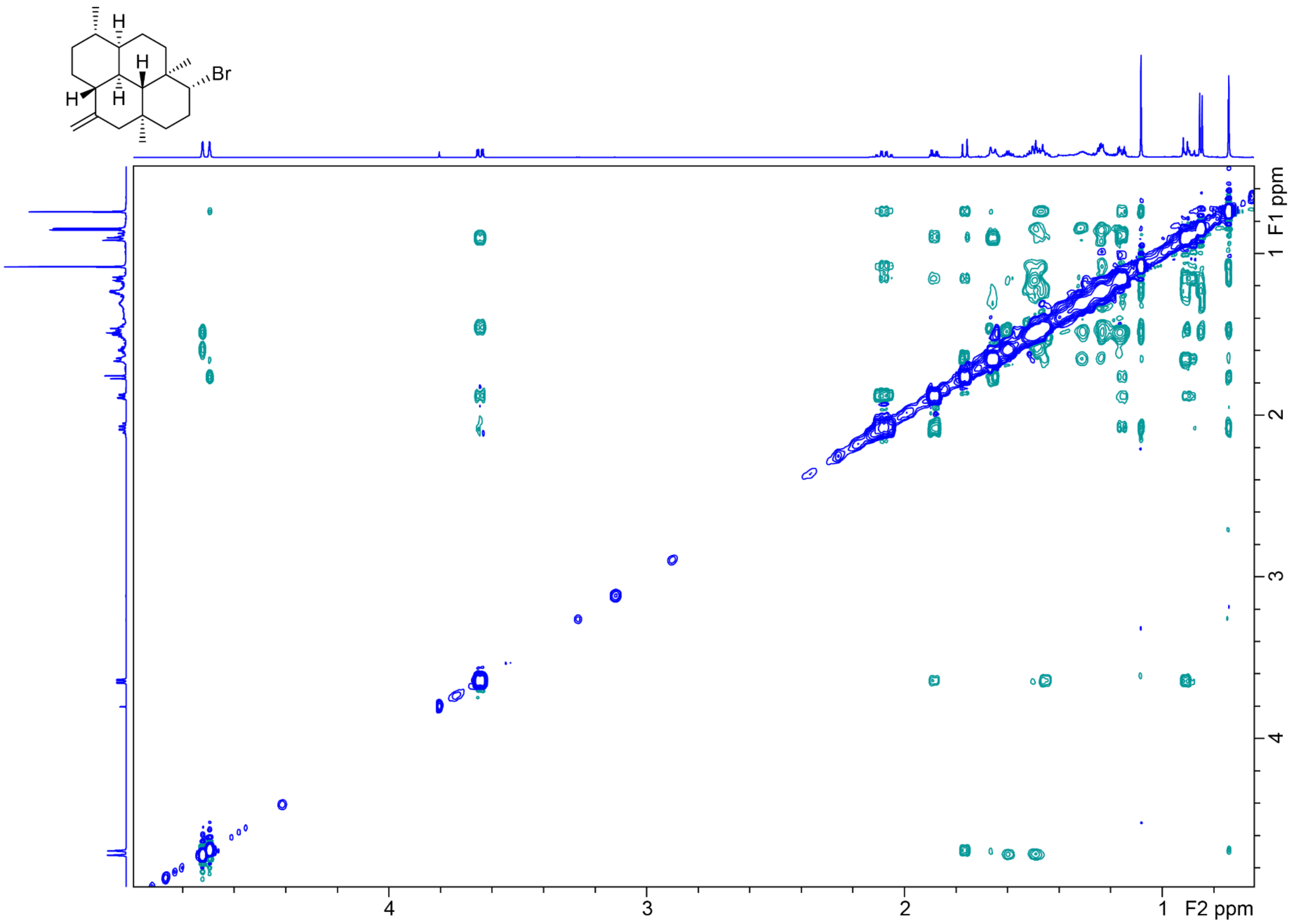

Figure S10. NOESY spectrum (C_6D_6) of **42**.

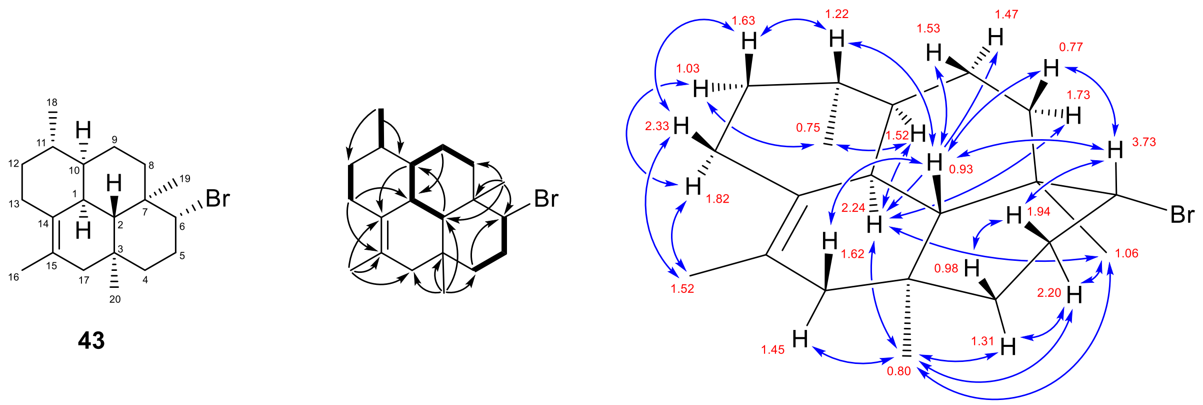

43

Figure S11. Structure elucidation of **43**. Bold: ^{1}H,^{1}H-COSY, single headed arrows: key HMBC, and blue double headed arrows: NOESY correlations.

Table S3. NMR data of **43** in C_6D_6 recorded at 298 K.

C[a]	type	^{13}C[b]	^{1}H[b]
1	CH	36.93	2.24 (m)
2	CH	47.84	0.93 (d, *J* = 11.6)
3	C_q	32.32	–
4	CH_2	43.92	1.31 (dt, *J* = 13.5, 3.5)
			0.98 (td, *J* = 3.8, 14.4)
5	CH_2	31.30	2.20 (dd, *J* = 13.2, 3.6)
			1.95 (dq, *J* = 13.9, 3.4)
6	CH-Br	69.45	3.73 (dd, *J* = 3.9, 12.2)
7	C_q	39.17	–
8	CH_2	38.25	1.73 (dt, *J* = 12.8, 4.0)
			0.77 (bd d, *J* = 3.8)
9	CH_2	21.26	1.53 (m)
			1.47 (m)
10	CH	37.98	1.52 (m)
11	CH	26.71	1.22 (m)
12	CH_2	34.03	1.63 (m)
			1.03 (m)
13	CH_2	24.05	2.33 (dd, *J* = 8.2, 13.3)
			1.82 (m)
14	C_q	122.52	–
15	C_q	130.85	–
16	CH_3	18.89	1.52 (br s)
17	CH_2	52.29	1.62 (m)
			1.46 (m)
18	CH_3	19.23	0.75 (d, *J* = 6.6)
19	CH_3	16.94	1.06 (s)
20	CH_3	20.57	0.80 (s)

[a] Carbon numbering as shown in Figure S11. [b] Chemical shifts δ in ppm, multiplicity: s = singlet, d = doublet, m = multiplet, t = triplet, q = quartet, br = broad, coupling constants *J* are given in Hertz.

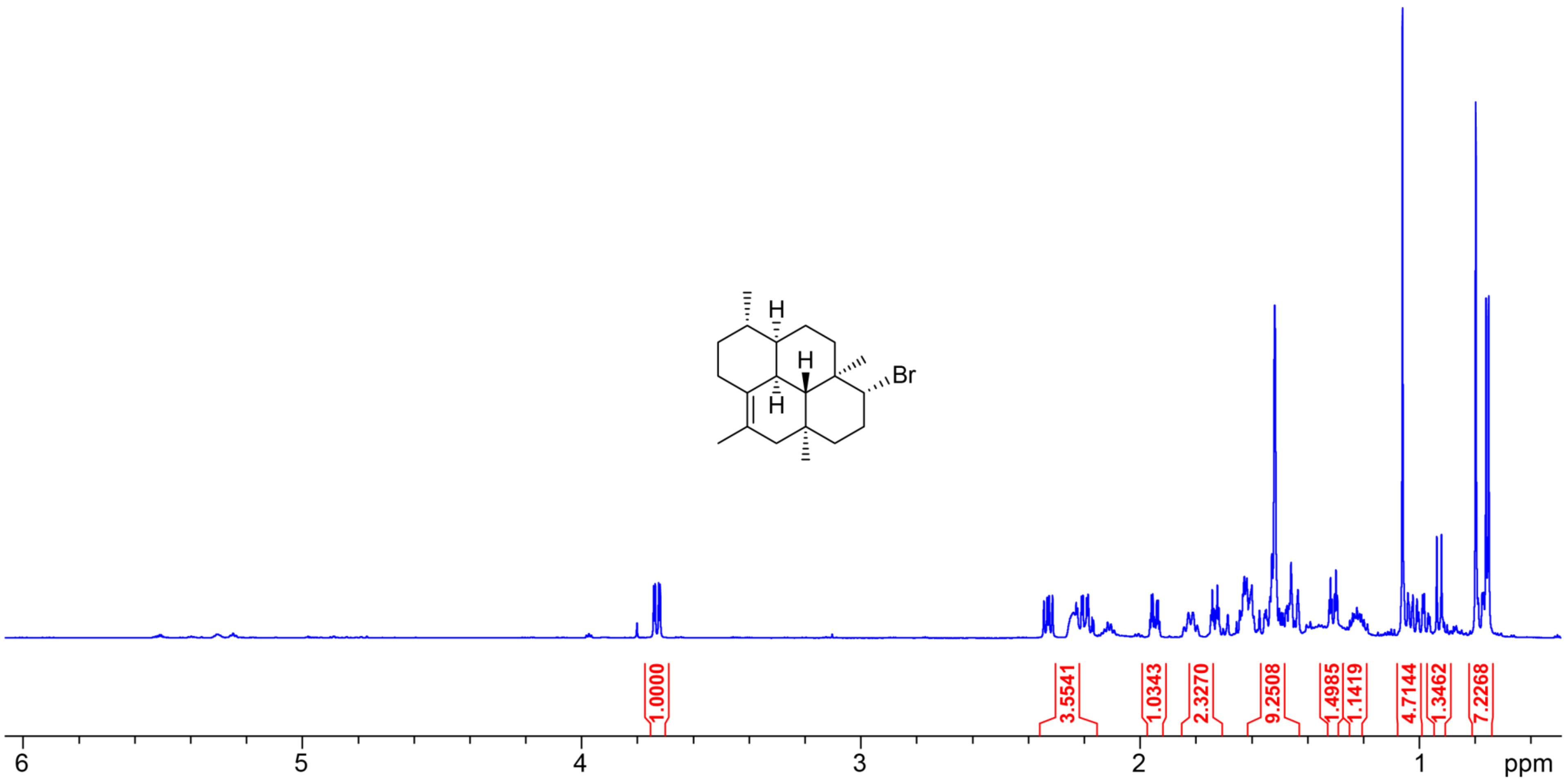

Figure S12. ^{1}H-NMR spectrum of **43** (700 MHz, C_6D_6).

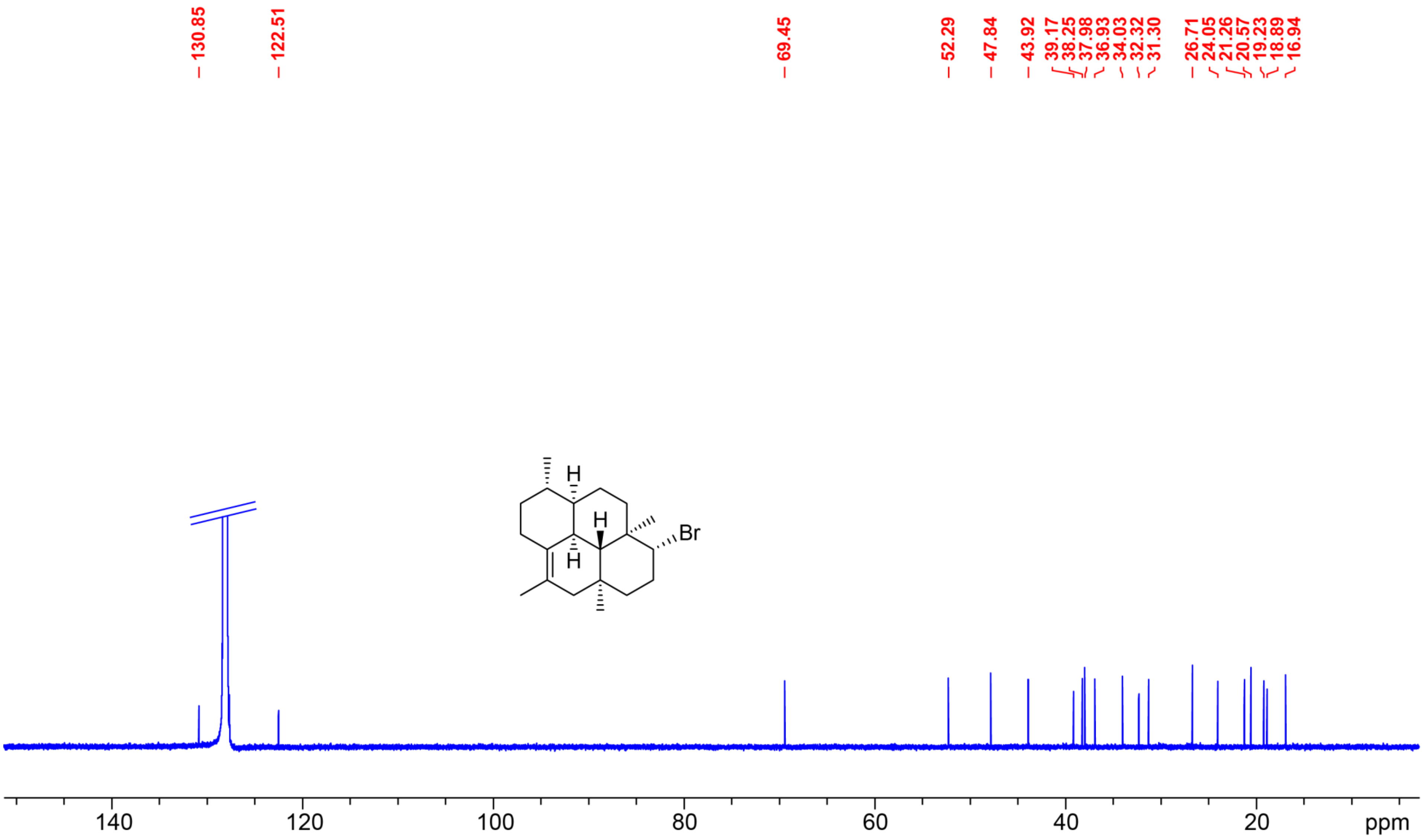

Figure S13. ^{13}C-NMR spectrum of **43** (176 MHz, C_6D_6).

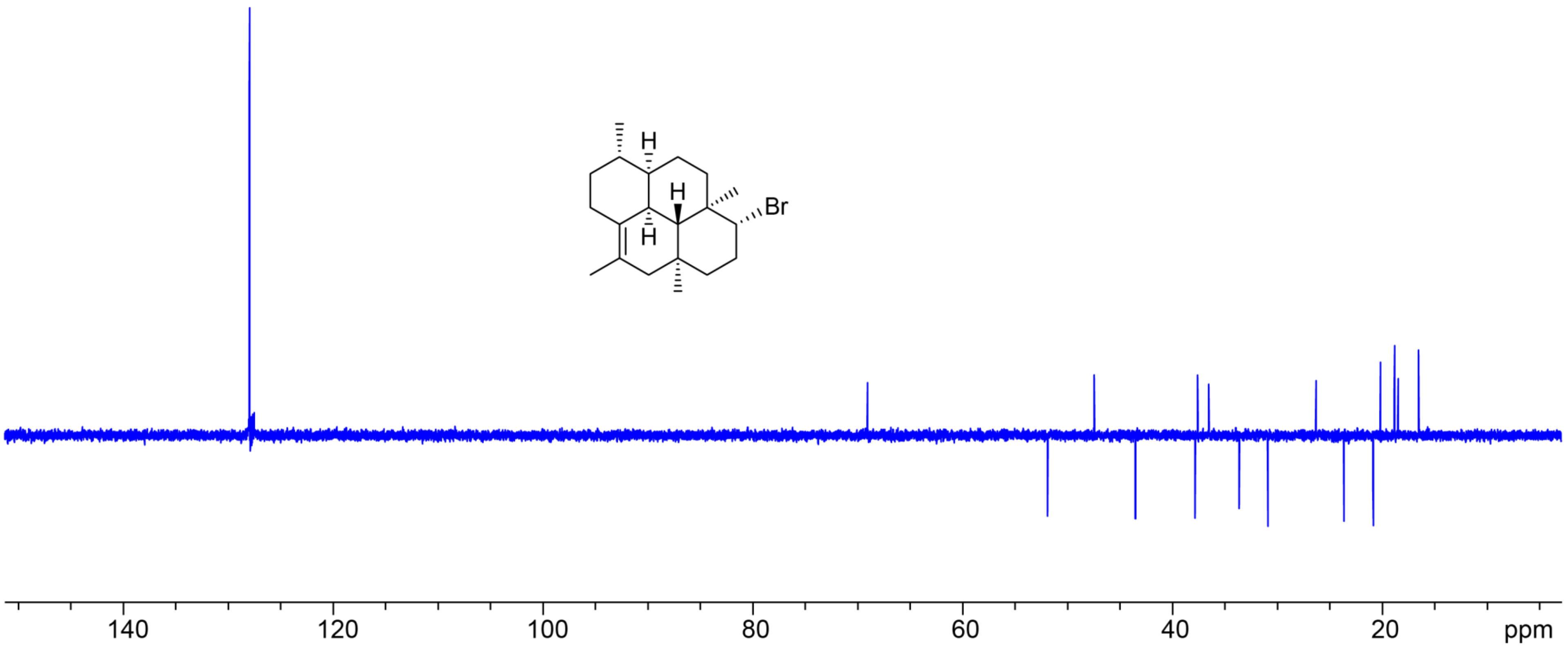

Figure S14. ^{13}C-DEPT135 spectrum of **43** (176 MHz, C_6D_6).

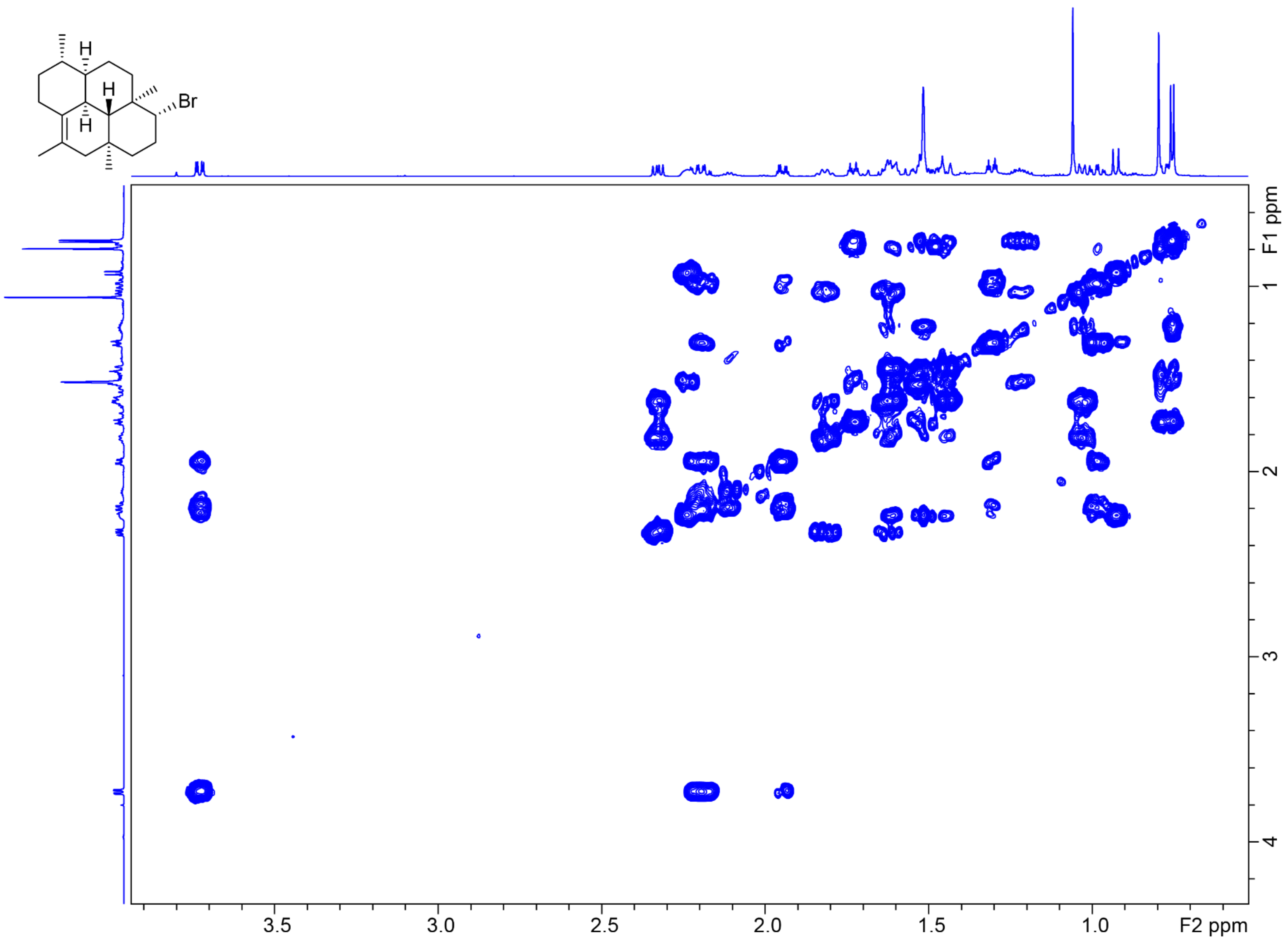

Figure S15. ¹H-¹H-COSY spectrum (C_6D_6).of **43**.

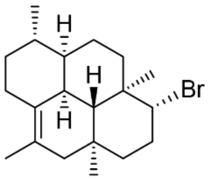

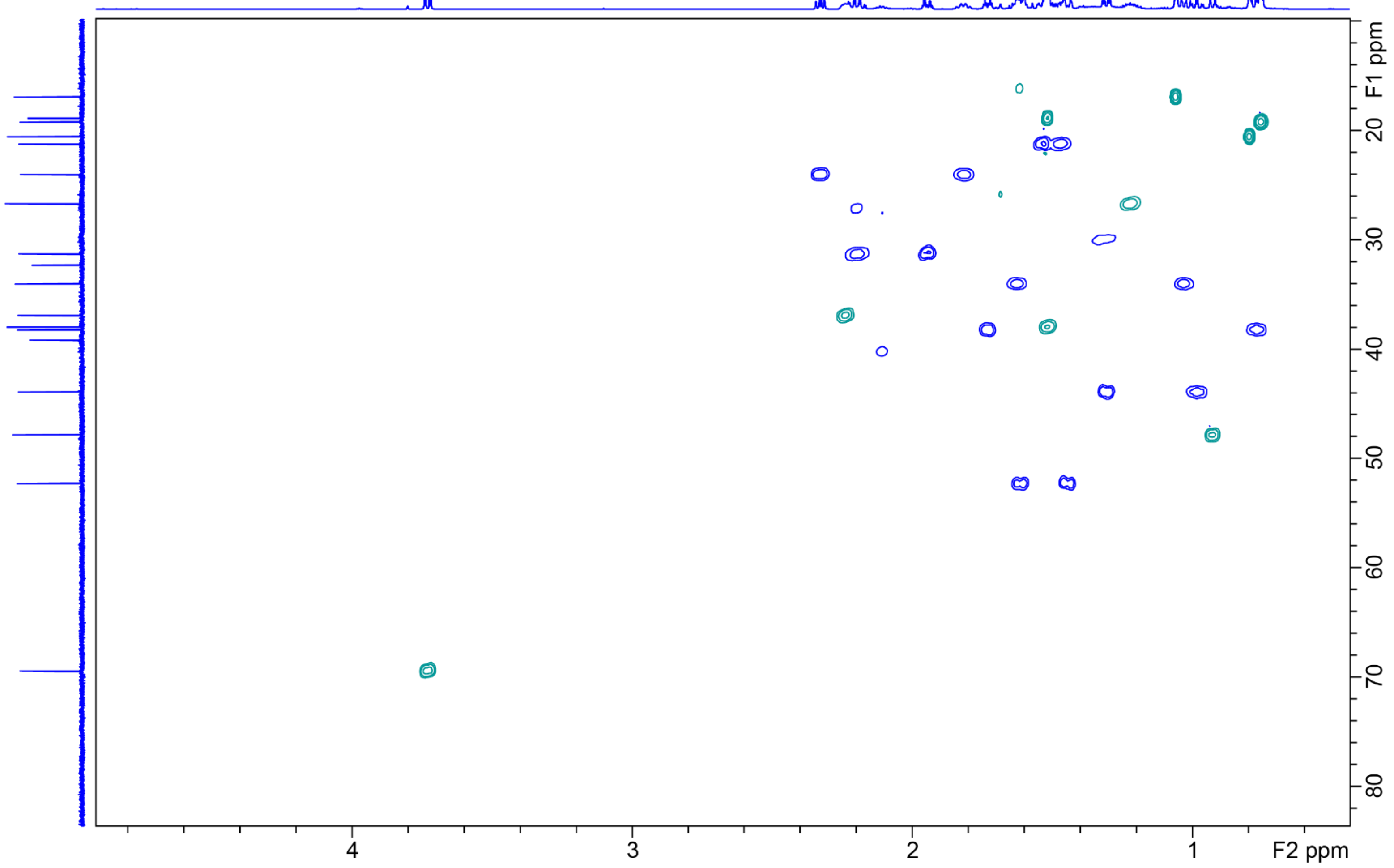

Figure S16. HSQC spectrum (C_6D_6) of **43**.

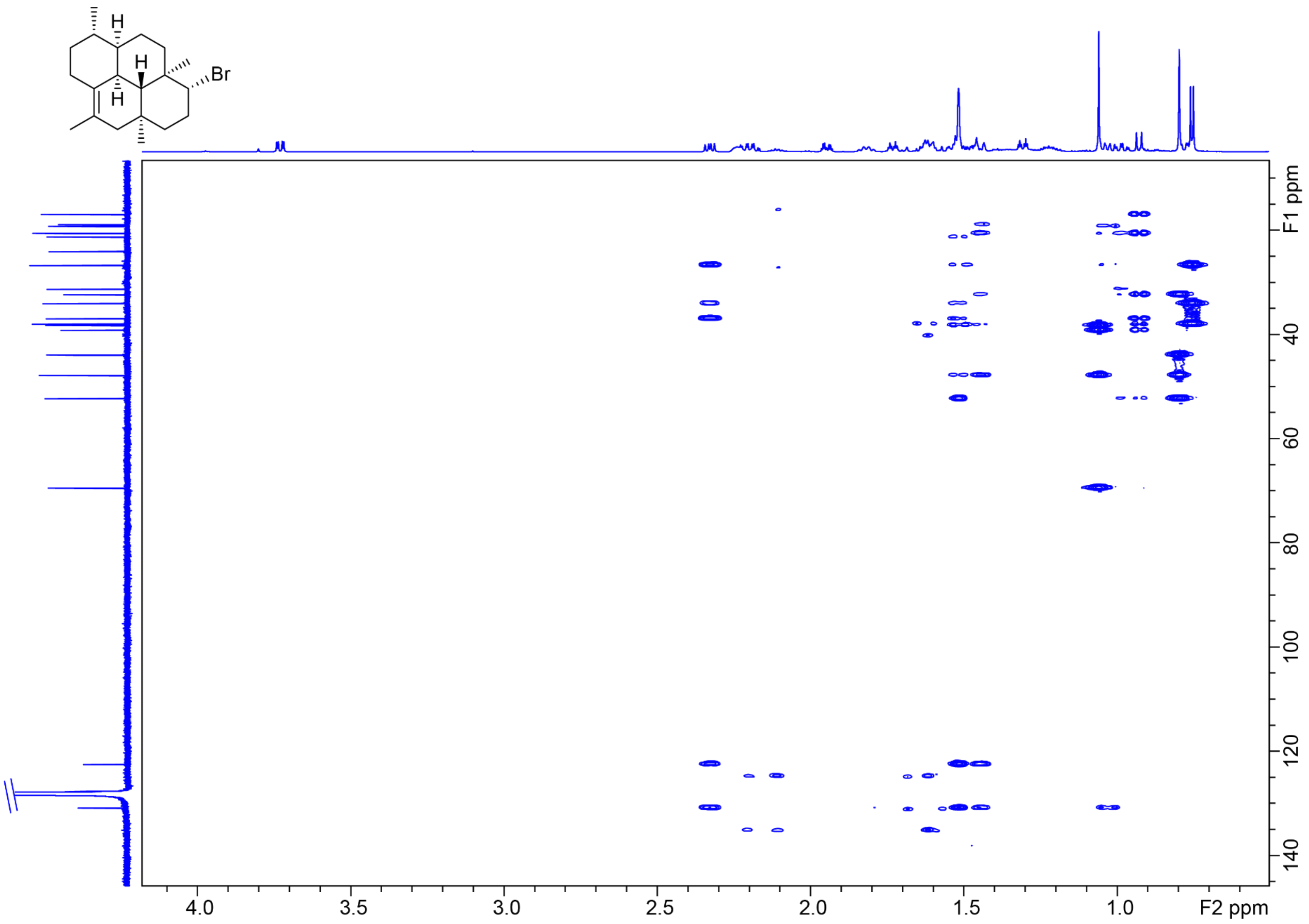

Figure S17. HMBC spectrum (C_6D_6) of **43**.

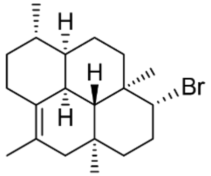

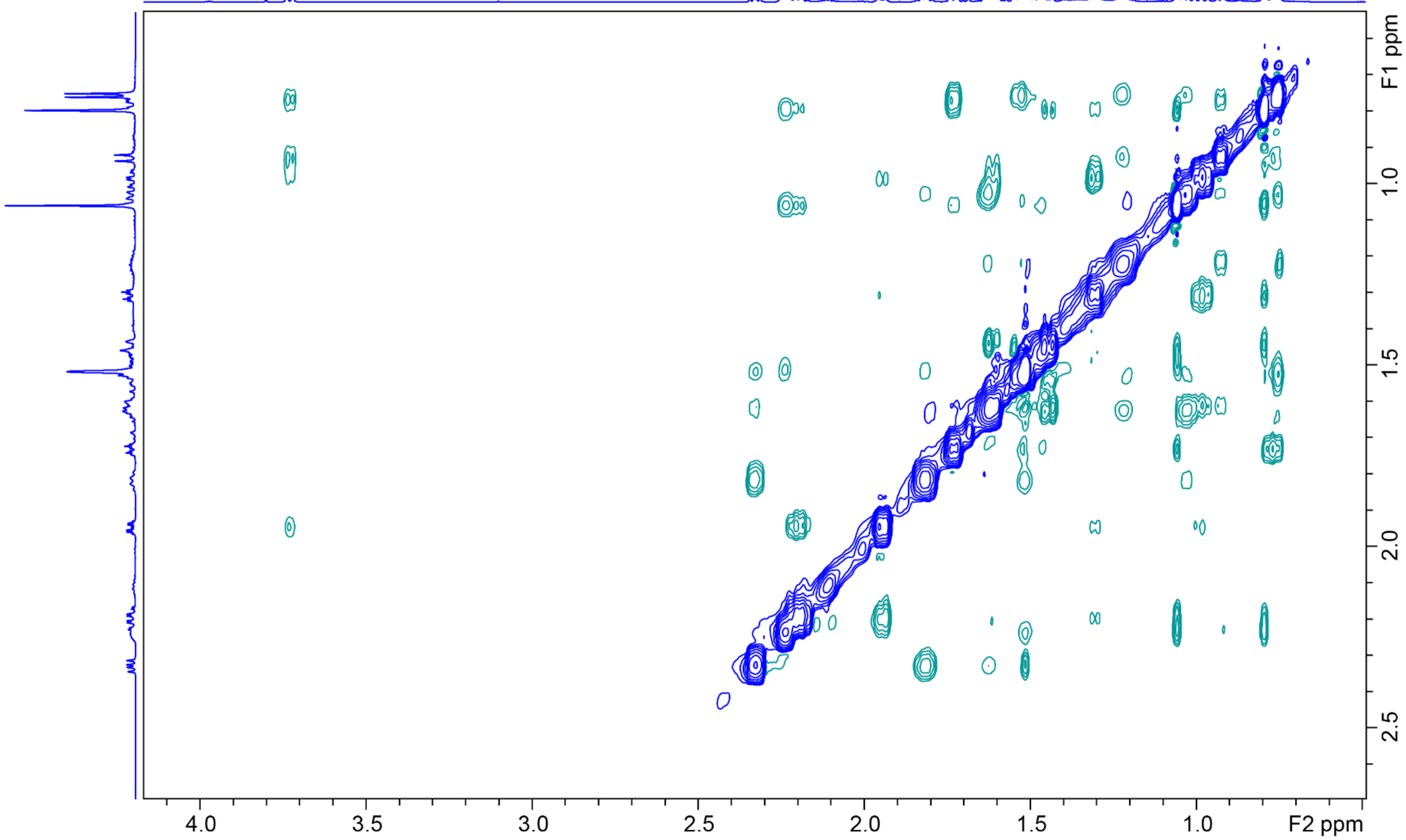

Figure S18. NOESY spectrum (C_6D_6) of **43**.

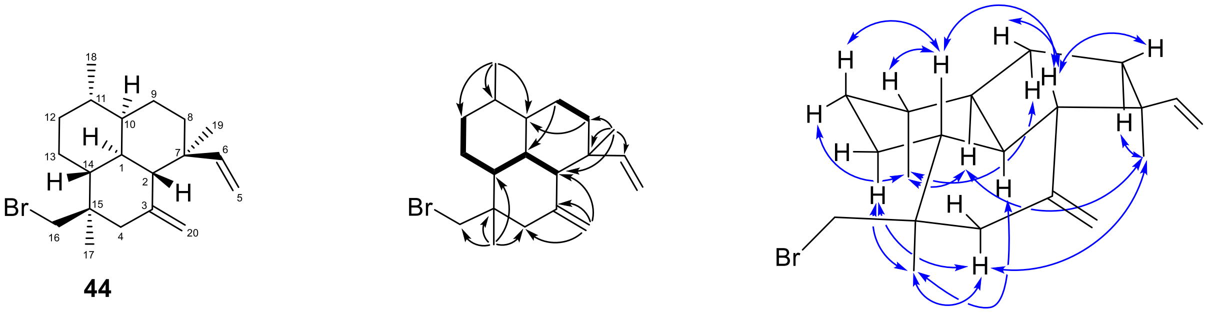

Figure S19. Structure elucidation of **44**. Bold: ^{1}H,^{1}H-COSY, single headed arrows: key HMBC, and blue double headed arrows: NOESY correlations.

Table S4. NMR data of **44** in C_6D_6 recorded at 298 K.

C[a]	type	^{13}C[b]	^{1}H[b]
1	CH	33.43	1.51 (m)
2	CH	47.28	1.88 (br d, *J* = 12.6)
3	C_q	145.45	–
4	CH_2	43.06	2.32 (d, *J* = 13.5)
			1.74 (d, *J* = 13.7)
5	CH_2	109.72	4.97 (m)
			4.95 (m)
6	CH	147.75	5.91 (dd, *J* = 10.9, 16.8)
7	C_q	39.89	–
8	CH_2	35.30	1.48 (m)
			1.11 (m)
9	CH_2	23.05	1.34 (m)
			1.00 (m)
10	CH	38.45	1.12 (m)
11	CH	27.94	1.11 (m)
12	CH_2	31.24	1.43 (m)
			0.95 (m)
13	CH_2	21.01	1.09 (m)
			0.94 (d, *J* = 3.6)
14	CH	42.05	0.61 (dt, *J* = 2.6, 11.6)
15	C_q	38.29	–
16	CH_2 (-Br)	44.72	3.20 (d, *J* = 5.9)
			3.08 (dd, *J* = 9.7, 1.6)
17	CH_3	20.93	0.82 (s)
18	CH_3	19.45	0.72 (d, *J* = 6.0)
19	CH_3	20.12	0.91 (s)
20	CH_2	114.21	4.93 (m)
			4.84 (m)

[a] Carbon numbering as shown in Figure S19. [b] Chemical shifts δ in ppm, multiplicity: s = singlet, d = doublet, m = multiplet, br = broad, t = triplet, coupling constants *J* are given in Hertz.

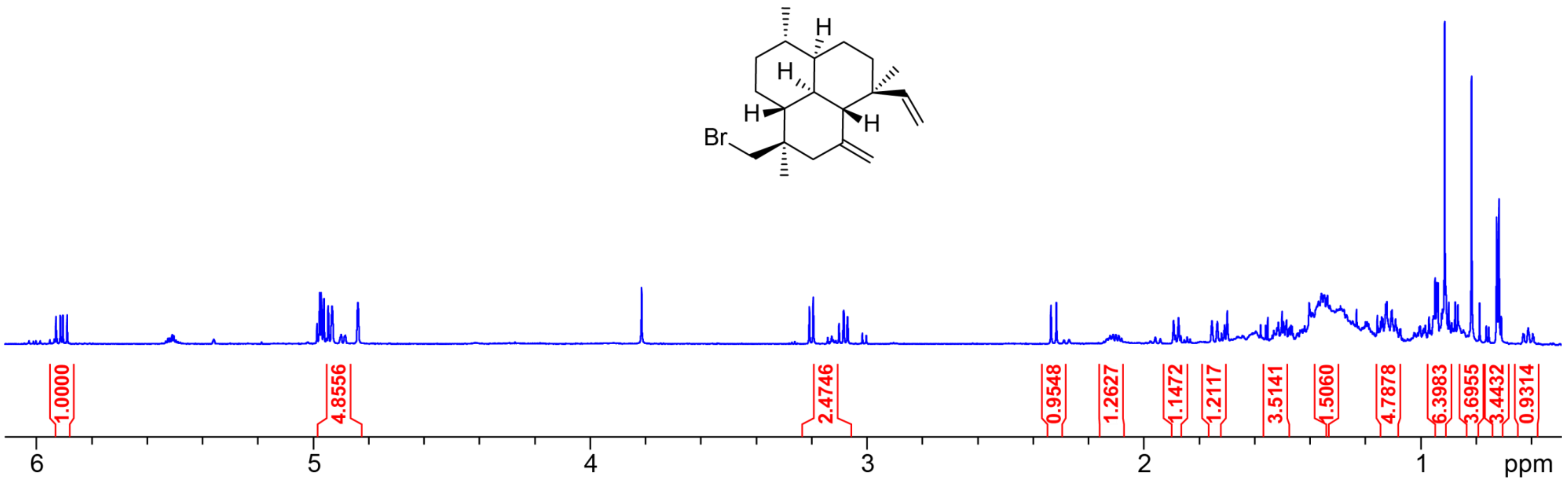

Figure S20. ^{1}H-NMR spectrum of **44** (700 MHz, C_6D_6).

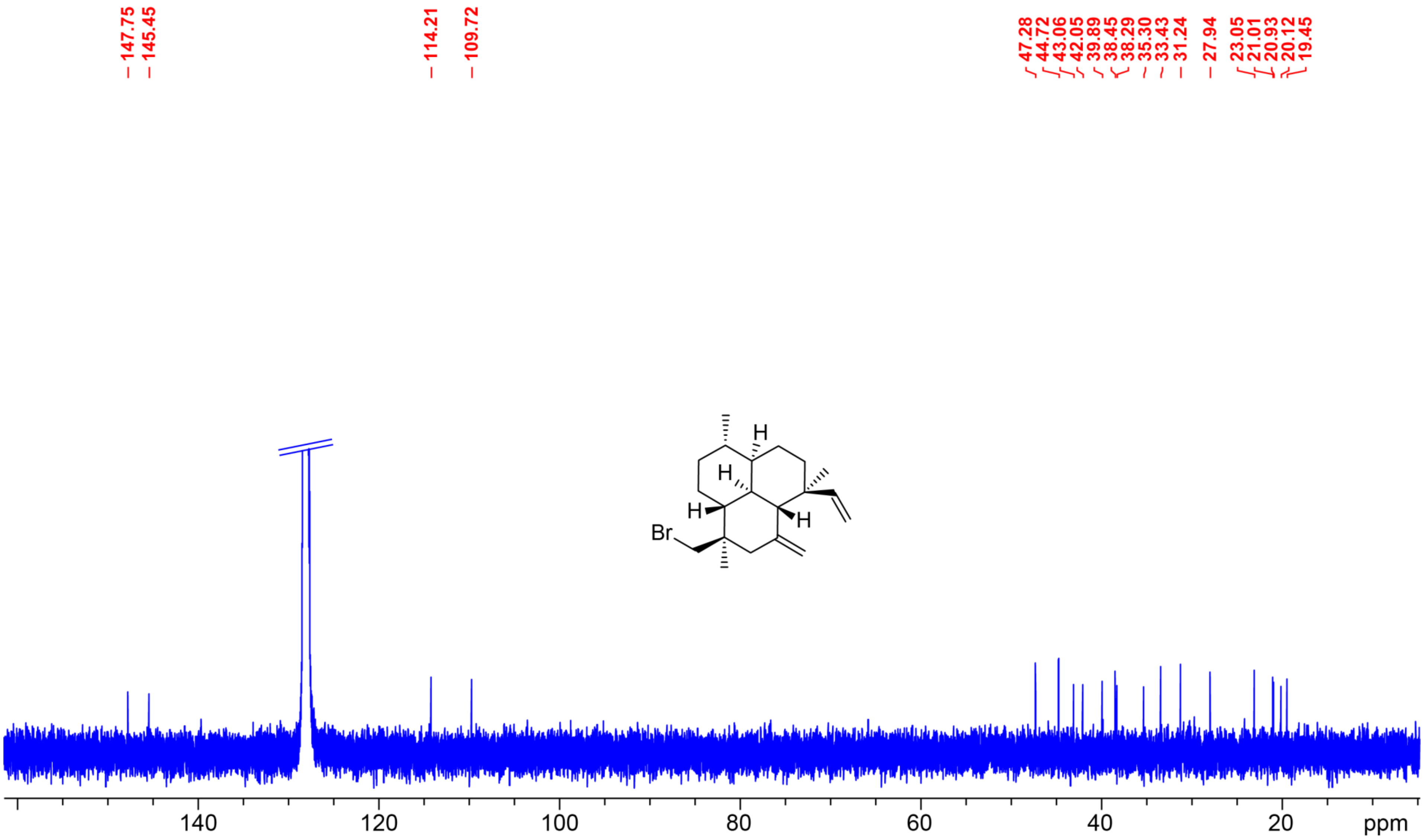

Figure S21. ^{13}C-NMR spectrum of **44** (700 MHz, C_6D_6).

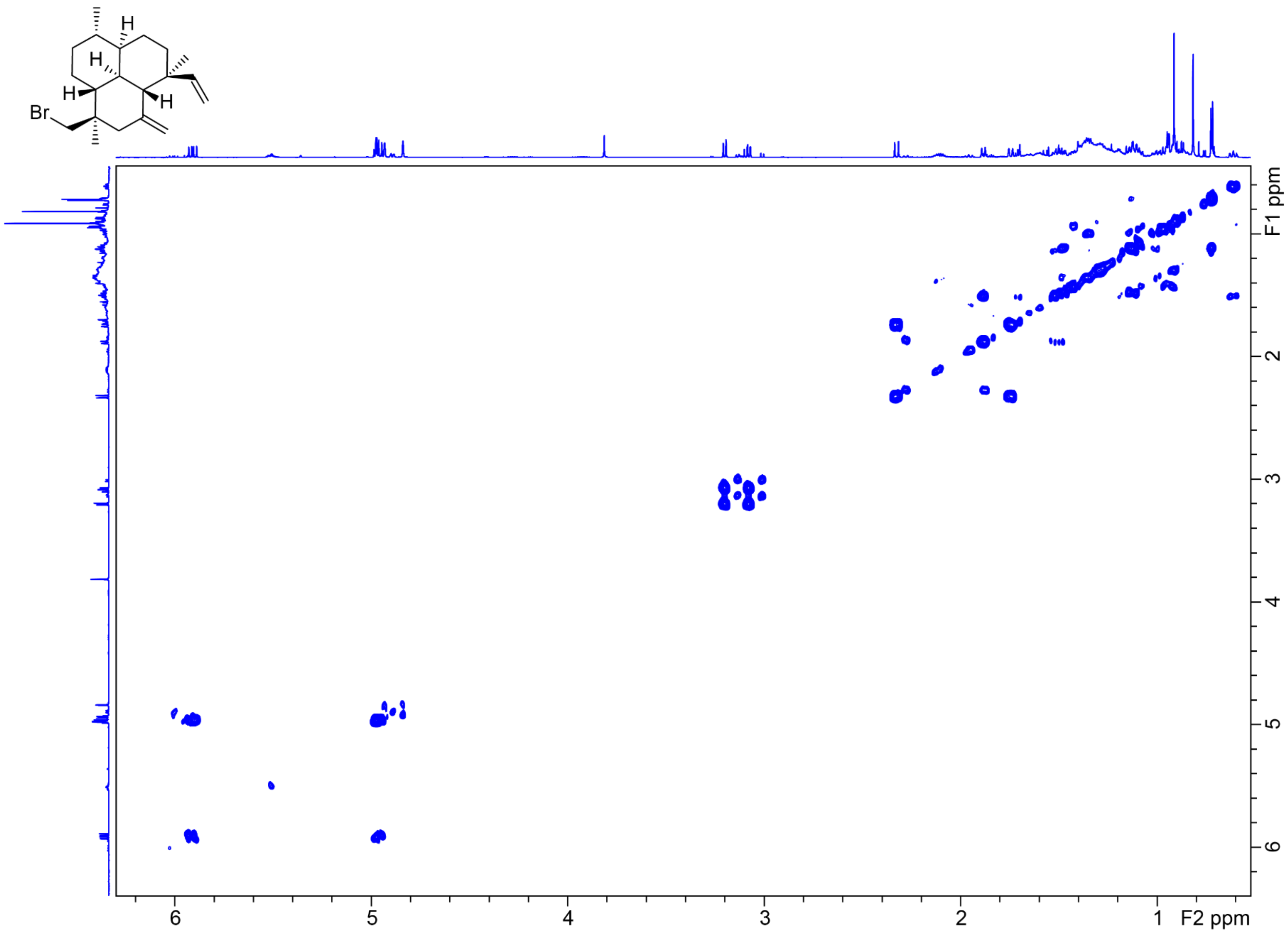

Figure S22. ^{1}H-^{1}H-COSY spectrum (C_6D_6) of **44**.

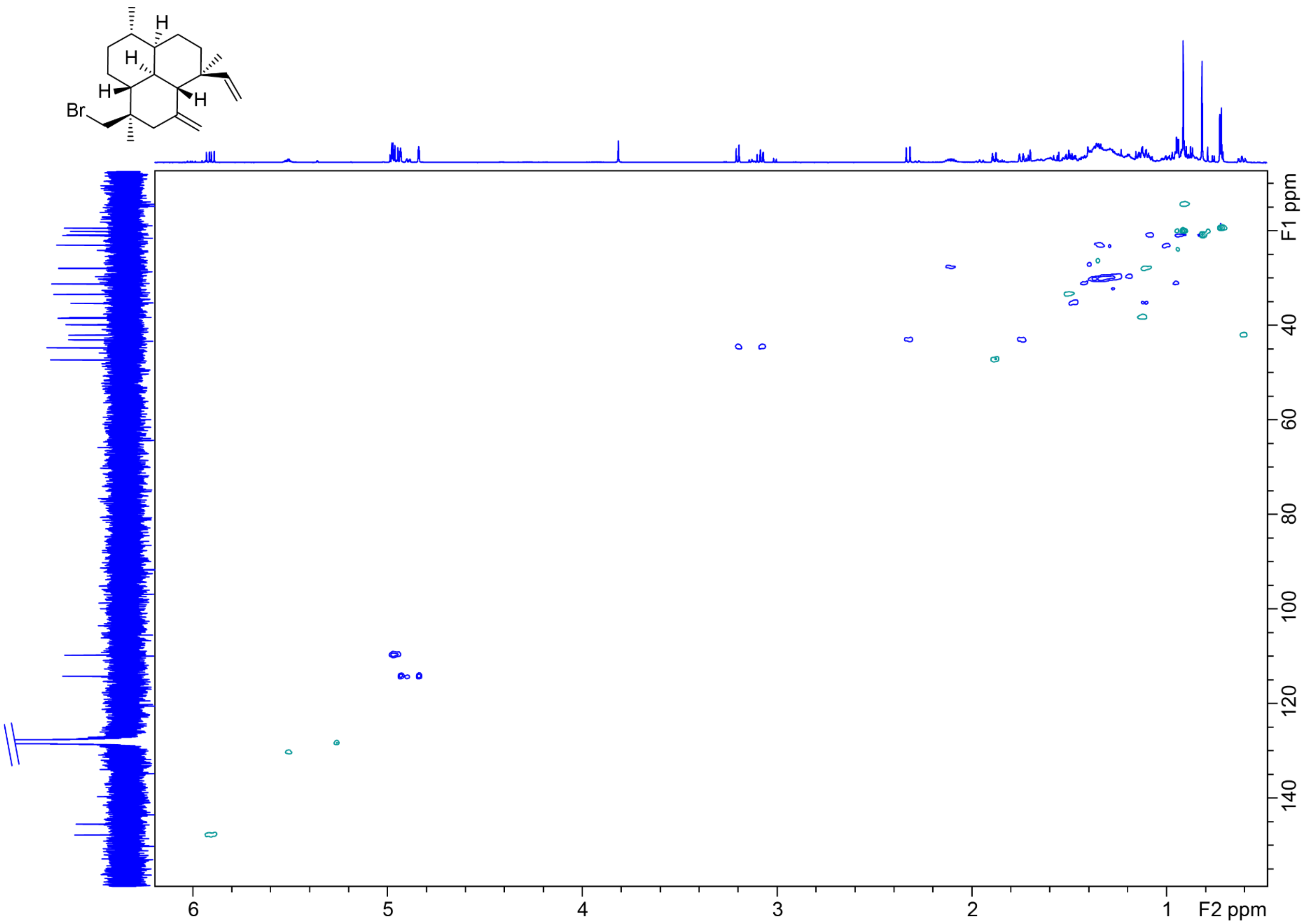

Figure S23. HSQC spectrum (C_6D_6) of **44**.

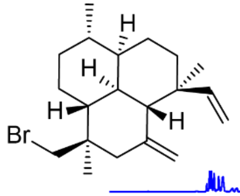

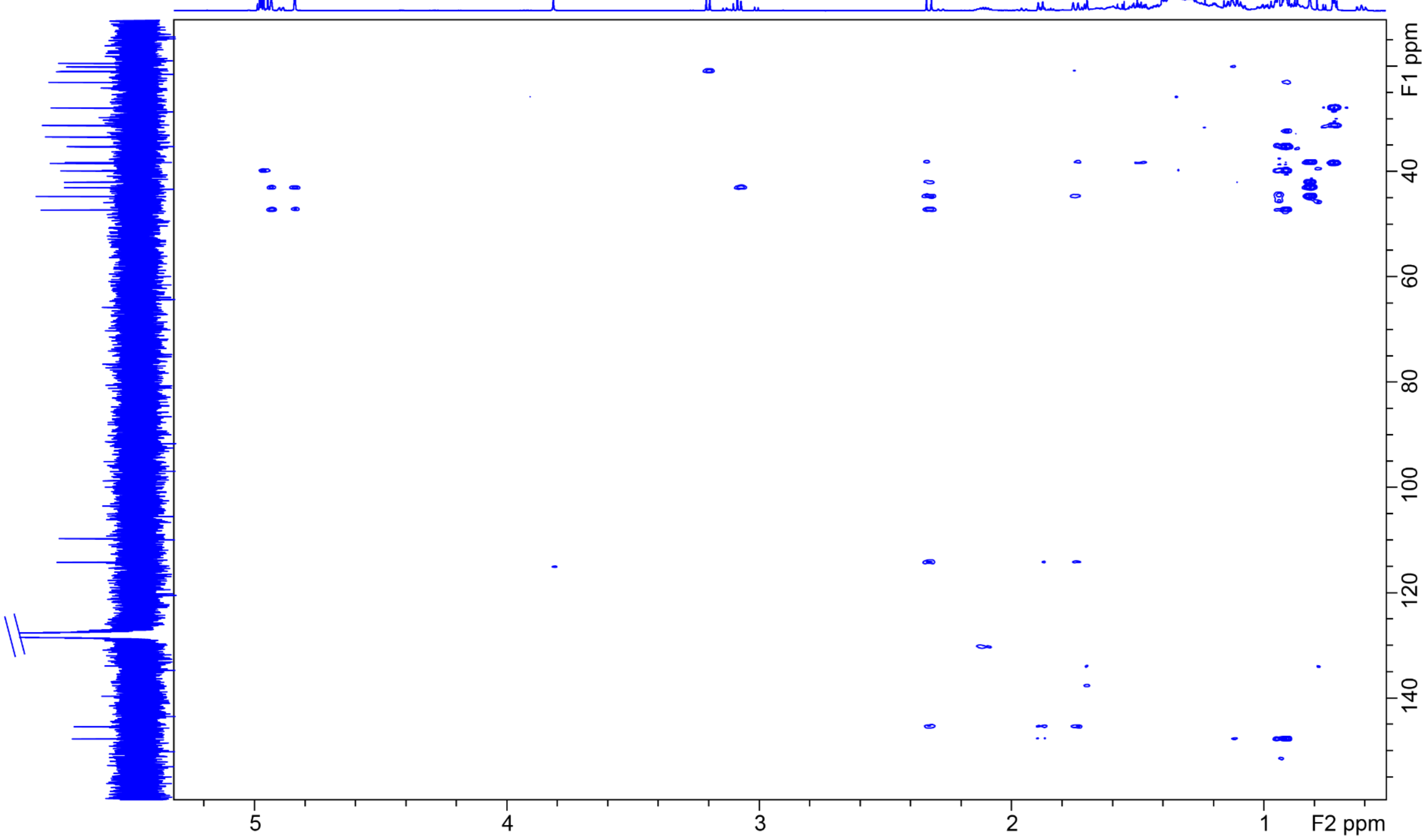

Figure S24. HMBC spectrum (C_6D_6) of **44**.

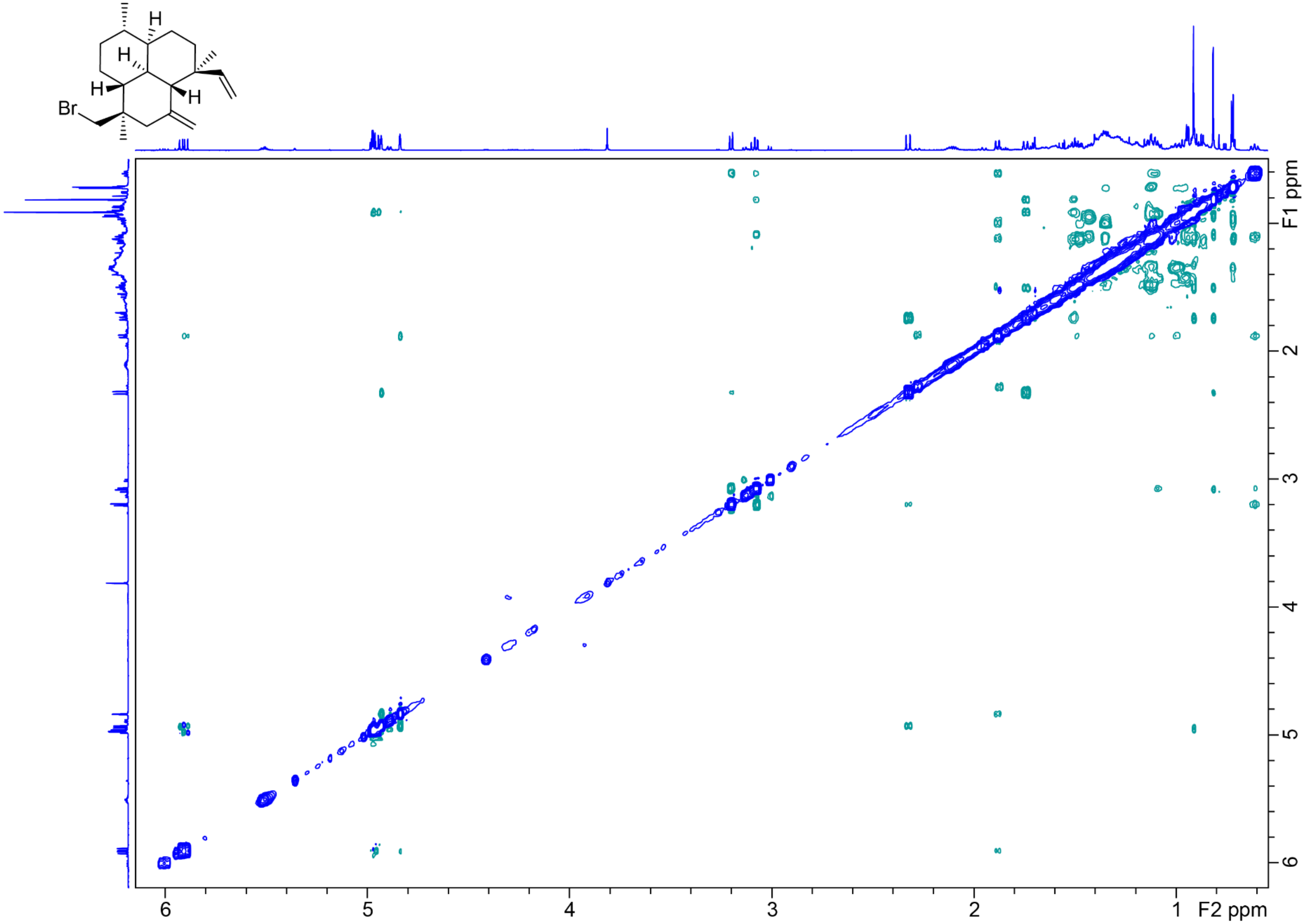

Figure S25. NOESY spectrum (C_6D_6) of **44**.

Computational methods

All computed structures were geometry optimized without restrictions and were characterized as minima or as transition state structures by frequency analyses using the B97D3/6-31g(d,p) method with the density fitting approximation for s- and p-functions, including Grimme's empirical D3-dispersion correction[13] in Gaussian16.[14] Frequency computations also provided Gibbs corrections, which include Grimme's quasi-RRHO approach with a frequency cut-off value of 100.0 wave numbers using GoodVibes.[15,16] For single point energies, the mPW1PW91 functional was applied with the 6-311+G(d,p) basis set without density fitting and the ultra-fine integration grid, as this method was shown to be very reliable for examining carbocation cyclization and rearrangement reactions.[17-21] Conformational analyses were performed with xTB-GFN2 in the CREST 2.12 program (github.com/crest-lab), developed by the Grimme group.[22-26]

Table S5. Results of DFT calculations for bromination of **41** with NBS (Scheme 4 of main text).

Structure	Gibbs energy (298.15K) in Hartree	energy relative to E or G in kcal/mol	reaction barrier in kcal/mol	Gibbs free energy in kcal/mol
**E**	–3355.442271	0.00		
**E-F-TS**	–3355.444553	–1.43	–1.43	
**F**	–3355.451037	–5.50		–5.50
**F**	–3355.451037	–5.50		
**F-G-TS**	–3355.451471	–5.77	–0.27	
**G**	–3355.486467	–27.73		–22.23
**H**	–3355.412059	0.00		
**H-J-TS**	–3355.404370	4.82	4.82	
**J**	–3355.443379	–19.65		–19.65
**J**	–3355.443378	–19.65		
**J-K-TS**	–3355.442001	–18.79	0.86	
**K**	–3355.440871	–18.08		1.57
**K**	–3355.440853	–18.07		
**K-L-TS**	–3355.392006	12.58	30.65	
**L**	–3355.438893	–16.84		1.23

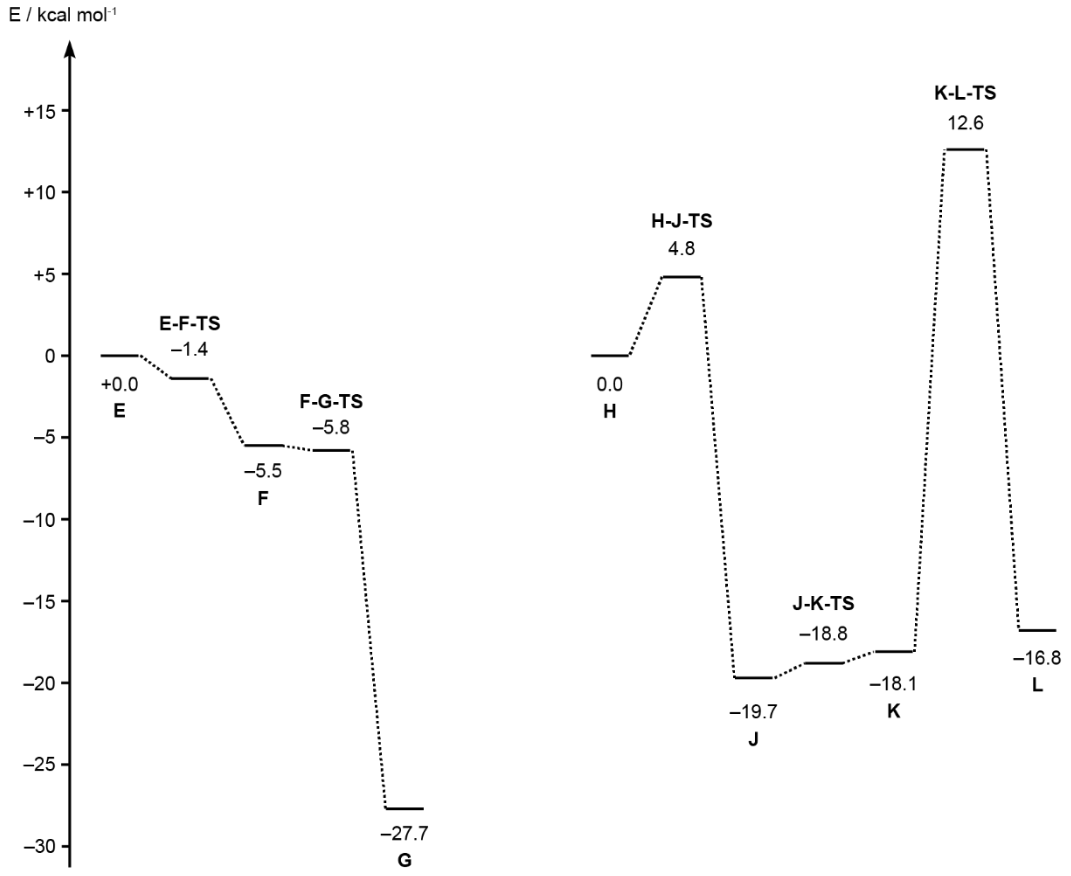

Figure S26. Energy profile for the bromination of **41** with NBS (Scheme 4 of main text, Table S5).

Incubation experiments with labelled substrates

Isotopic labelling experiments were performed with the substrates listed in Table S6 (ca. 1 mg of each substrate in 1 mL 25 mM NH_4HCO_3; 5 mg of each substrate were used for entries 4 and 5), incubation buffer (5 mL) and preparations of purified enzymes (each 1 mL). After incubation at 28 °C overnight, the products were extracted with C_6D_6 (600 µL + 300 µL) or *n*-hexane (500 µL), the extracts were dried with $MgSO_4$ and analysed by NMR and/or GC/MS.
For entries 4 and 5, the crude extracts from the enzymatic reaction in n-hexane were concentrated and the residues were dissolved in CH_2Cl_2 (1 mL), followed by the addition of NBS (0.5 mg) at –78 °C. The reaction mixtures were stirred at this temperature for 20 min, then warmed to room temperature within 1 h and stirring was continued for another 1 h. The reactions were quenched with sat. NH_4Cl, extracted with n-pentane, and the extracts were dried over $MgSO_4$. The solvent was evaporated and the samples were dissolved in C_6D_6 (0.5 mL), followed by NMR analysis.

Table S6. Labelling experiments with different DTSs.

entry	substrates	enzymes	results in
1	(7-^{13}C,6-^{2}H)FPP + IPP[27]	GGPPS,[11] Bnd4	Figure S27
2	(*R*)-(1-^{13}C,1-^{2}H)*iso*-GGPP I[28]	Bnd4	Figure S37
3	(*S*)-(1-^{13}C,1-^{2}H)*iso*-GGPP I[28]	Bnd4	Figure S37
4	DMAPP[27] + (*E*)-(4-^{13}C,4-^{2}H)IPP[7]	GGPPS,[11] Bnd4, then NBS	Figure S38
5	DMAPP[27] + (*Z*)-(4-^{13}C,4-^{2}H)IPP[7]	GGPPS,[11] Bnd4, then NBS	Figure S38
6	(7-^{13}C,6-^{2}H)FPP + IPP[27]	GGPPS,[11] VenA	Figure S40
7	(3-^{13}C)GPP[29] + (1,1-$^{2}H_2$)IPP[30]	GGPPS,[11] VenA	Figure S41
8	(*R*)-(1-^{13}C,1-^{2}H)*iso*-GGPP I[28]	VenA	Figure S51
9	(*S*)-(1-^{13}C,1-^{2}H)*iso*-GGPP I[28]	VenA	Figure S51
10	*iso*-FPP III + (1-^{13}C)IPP[11]	GGPPS,[11] CyS[5]	Figure S130
11	*iso*-FPP III + (1-^{13}C)IPP[11]	GGPPS,[11] NrPS[5]	Figure S130
12	*iso*-FPP III + (1-^{13}C)IPP[11]	GGPPS,[11] NtPS[5]	Figure S130
13	*iso*-FPP III + (1-^{13}C)IPP[11]	GGPPS,[11] PmS[7]	Figure S130
14	*iso*-FPP III + (1-^{13}C)IPP[11]	GGPPS,[11] AbVS[1]	Figure S130
15	*iso*-FPP III + (1-^{13}C)IPP[11]	GGPPS,[11] CaCS[3]	Figure S130
16	*iso*-FPP III + (1-^{13}C)IPP[11]	GGPPS,[11] SaS[8]	Figure S130
17	*iso*-FPP III + (1-^{13}C)IPP[11]	GGPPS,[11] SvS[11]	Figure S130
18	*iso*-FPP III + (1-^{13}C)IPP[11]	GGPPS,[11] *Si*CotB2[9]	Figure S130
19	*iso*-FPP III + (1-^{13}C)IPP[11]	GGPPS,[11] HdS[6]	Figure S130
20	*iso*-FPP III + (1-^{13}C)IPP[11]	GGPPS,[11] CgDS[4]	Figure S130
21	*iso*-FPP III + (*R*)-(1-^{13}C,1-^{2}H)IPP[31]	GGPPS,[11] PmS[7]	Figure S131
22	*iso*-FPP III + (*S*)-(1-^{13}C,1-^{2}H)IPP[31]	GGPPS,[11] PmS[7]	Figure S131
23	*iso*-FPP III + (*R*)-(1-^{13}C,1-^{2}H)IPP[31]	GGPPS,[11] *Si*CotB2[9]	Figure S132
24	*iso*-FPP III + (*S*)-(1-^{13}C,1-^{2}H)IPP[31]	GGPPS,[11] *Si*CotB2[9]	Figure S132

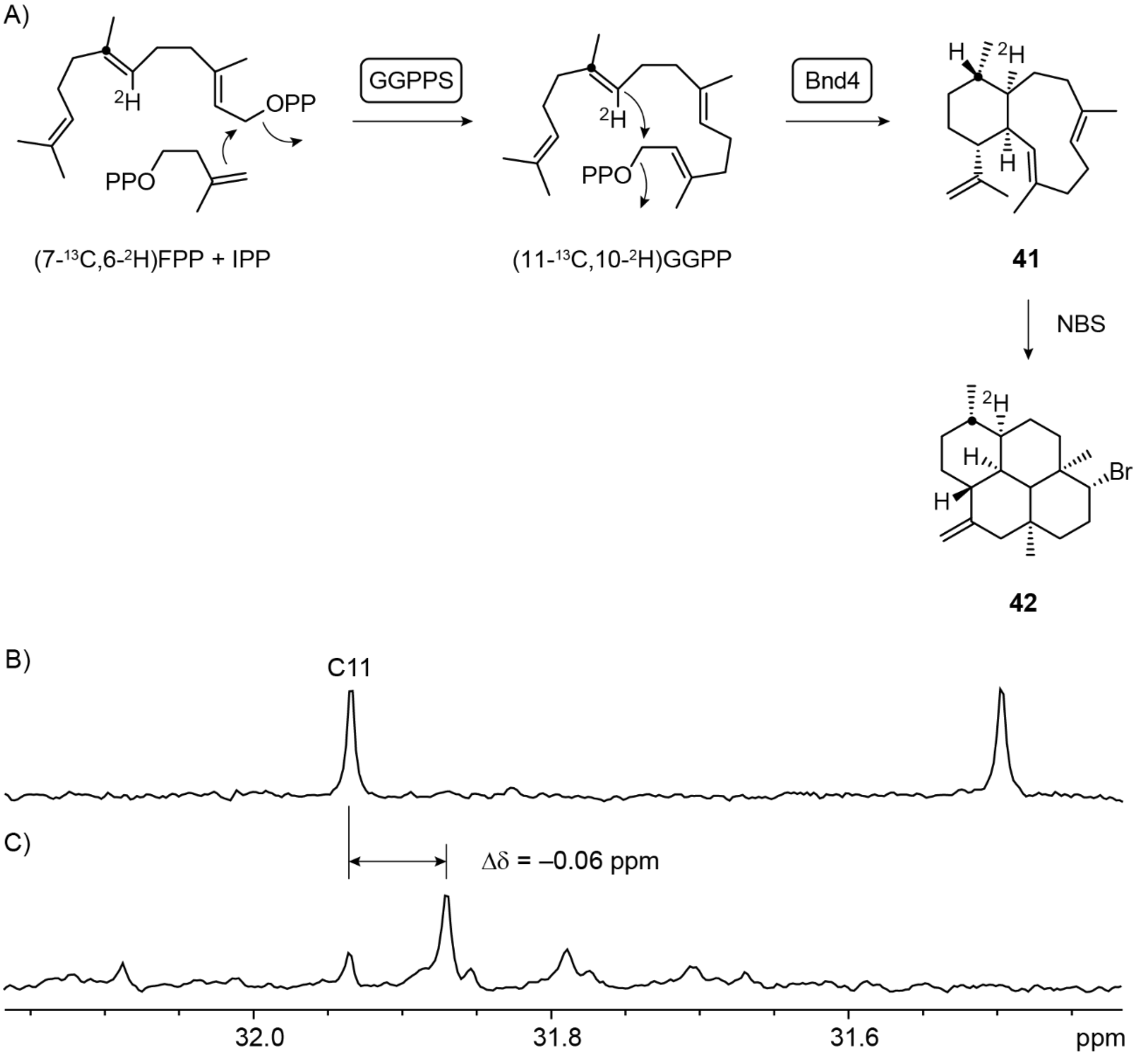

Figure S27. The 1,3-hydride shift from **B** to **C** in the biosynthesis of **41** (Scheme 4A of main text). A) The enzymatic conversion of (7-^{13}C,6-^{2}H)FPP plus IPP to (11-^{13}C,10-^{2}H)-**41** with GGPPS and Bnd4, followed by the treatment with NBS to yield (11-^{13}C,10-^{2}H)-**42**. B) Partial ^{13}C spectra of unlablelled **42** and C) partial ^{13}C spectra of (11-^{13}C,10-^{2}H)-**42**, showing the region for C11. The minor upfield shift ($\Delta\delta$ = –0.06 ppm) indicates deuterium located in a neighbouring position (C10) and thus supports the 1,3-hydride shift.

A)

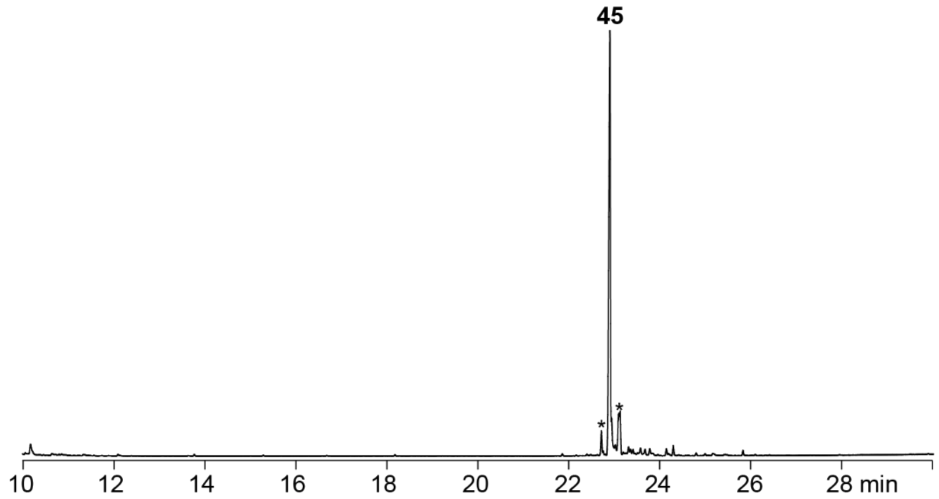

B)

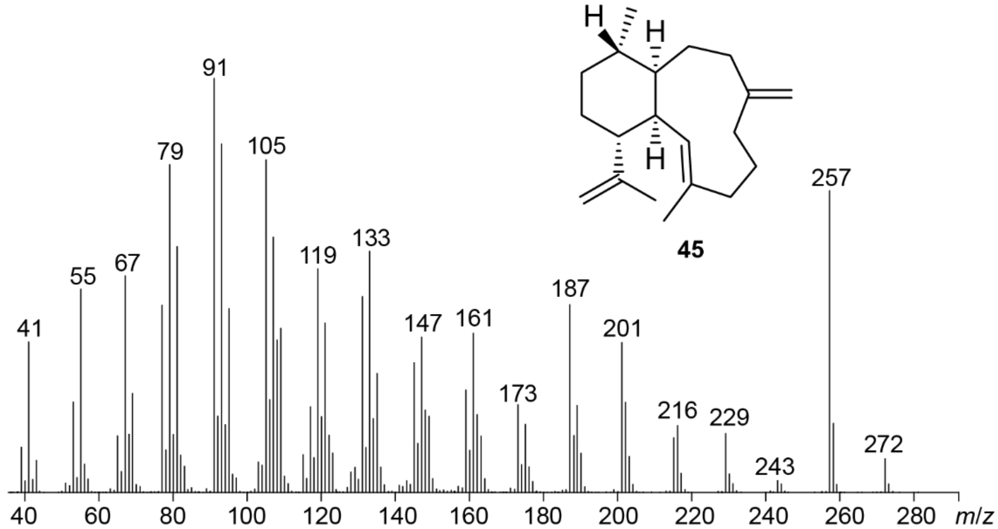

Figure S28. Enzymatic conversion of *iso*-GGPP I with Bnd4. A) Total ion chromatogram of the crude extract of the enzymatic reaction, B) EI mass spectrum of **45**. Asterisks indicate spontaneous lysis and hydrolysis products of *iso*-GGPP I.

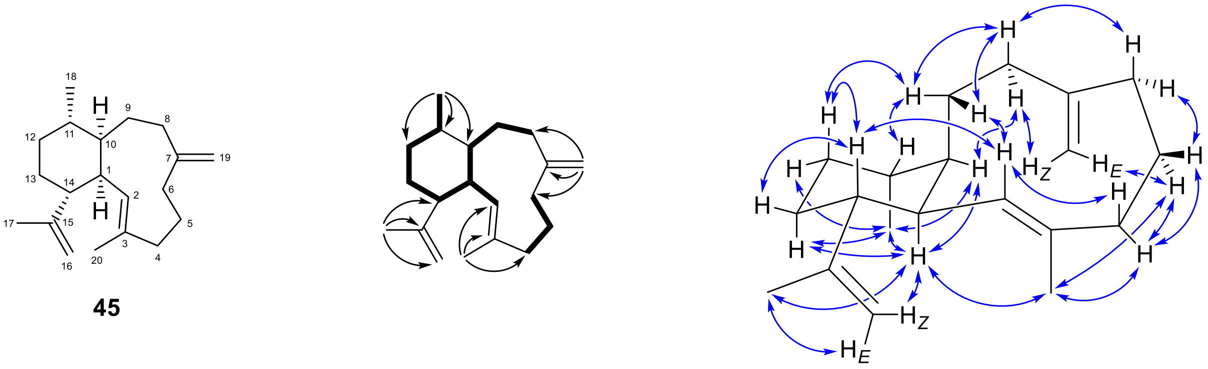

45

Figure S29. Structure elucidation of benditerpe-2,7(19),15-triene (**45**). Bold: ^{1}H,^{1}H-COSY, single headed arrows: key HMBC, and blue double headed arrows: NOESY correlations.

Table S7. NMR data of benditerpe-2,7(19),15-triene (**45**) in C_6D_6 recorded at 298 K.

C[a]	type	^{13}C[b]	^{1}H[b]
1	CH	35.13	2.52 (ddd, *J* = 12.0, 8.1, 4.6)
2	CH	131.69	5.07 (d, *J* = 8.1)
3	C_q	131.92	–
4	CH_2	41.36	1.98 (m, H_α)
			1.83 (m, H_β)
5	CH_2	23.45	1.73 (m, H_α)
			1.50 (m, H_β)
6	CH_2	31.00	2.00 (m, H_α)
			1.62 (m, H_β)
7	C_q	148.80	–
8	CH_2	39.04	2.09 (m, H_β)
			1.97 (m, H_α)
9	CH_2	27.41	1.81 (m, H_β)
			1.08 (m, H_α)
10	CH	40.80	1.62 (m)
11	CH	33.81	1.61 (m)
12	CH_2	27.50	1.69 (m, H_β)
			1.33 (br d, *J* = 13.1, H_α)
13	CH_2	26.57	1.55 (m, H_α)
			1.44 (dddd, *J* = 13.2, 3.3, 3.3, 3.3, H_β)
14	CH	47.37	2.01 (m)
15	C_q	149.28	–
16	CH_2	111.03	4.88 (br s, H_Z)
			4.83 (br s, H_E)
17	CH_3	19.12	1.65 (s)
18	CH_3	19.13	1.10 (d, *J* = 7.1)
19	CH_2	111.12	4.85 (br s, H_E)
			4.80 (br s, H_Z)
20	CH_3	16.65	1.54 (br s)

[a] Carbon numbering as shown in Figure S29. [b] Chemical shifts δ in ppm, multiplicity: s = singlet, d = doublet, m = multiplet, br = broad, coupling constants *J* are given in Hertz.

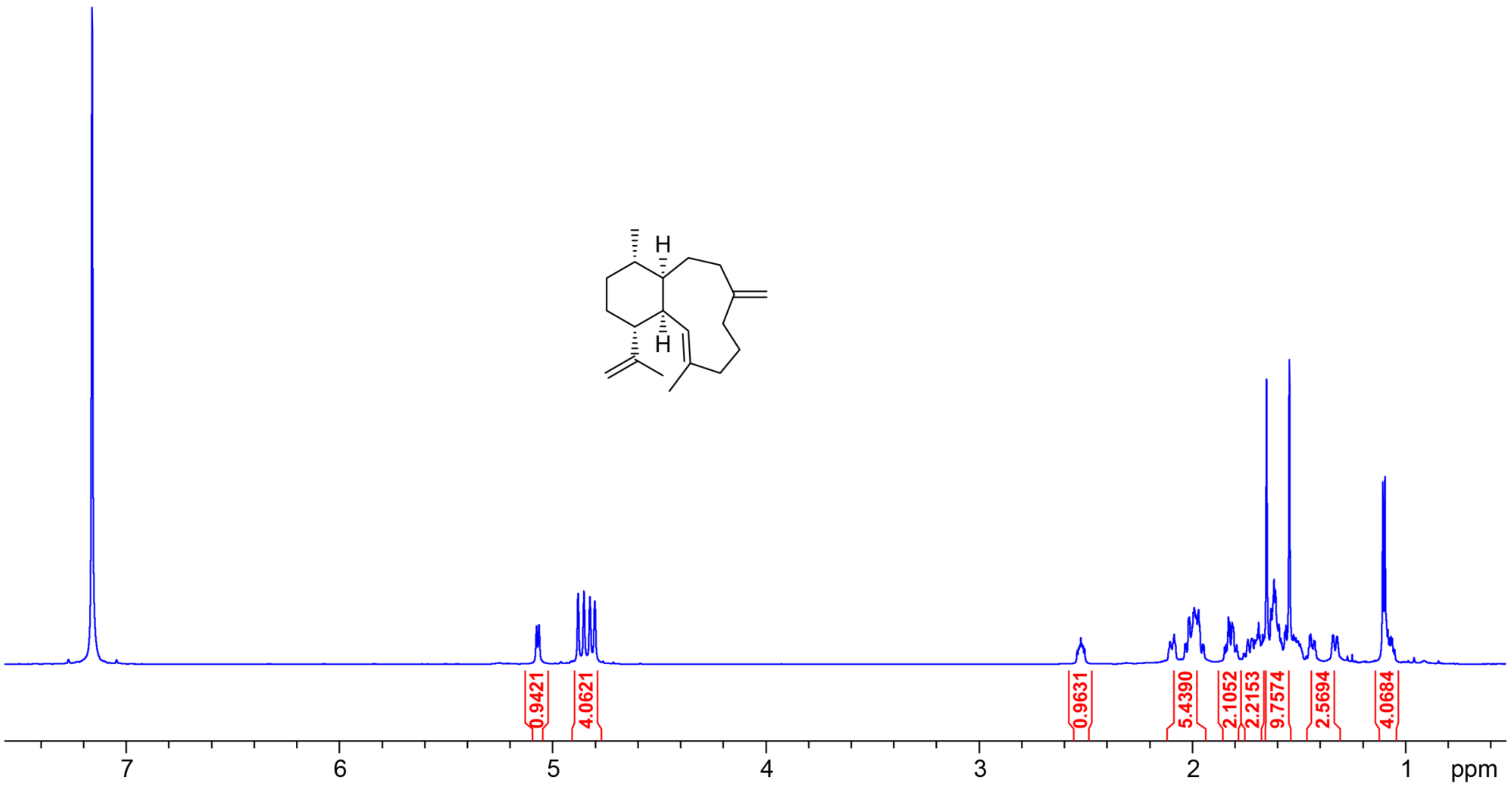

Figure S30. ^{1}H-NMR spectrum of **45** (700 MHz, C_6D_6).

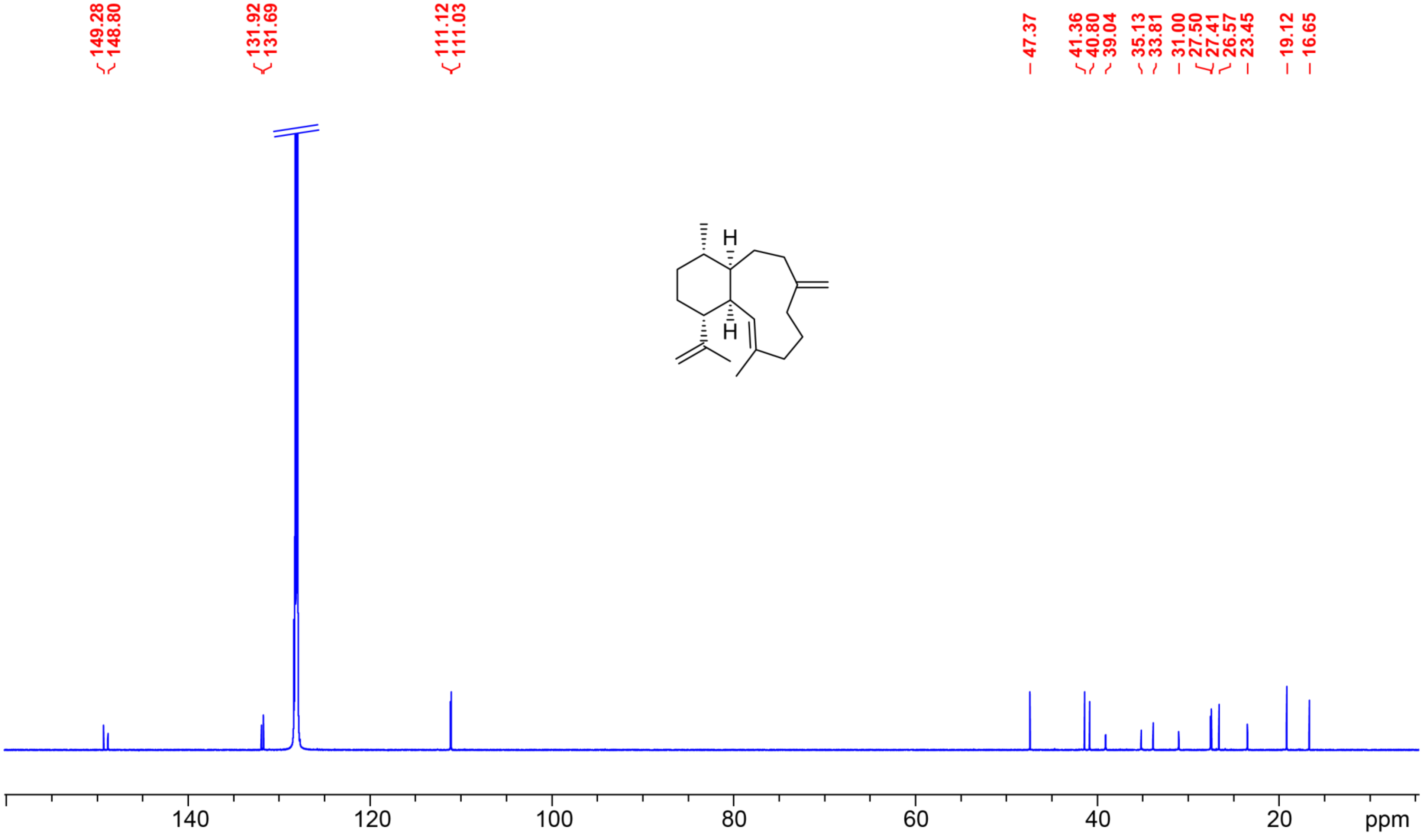

Figure S31. ^{13}C-NMR spectrum of **45** (176 MHz, C_6D_6).

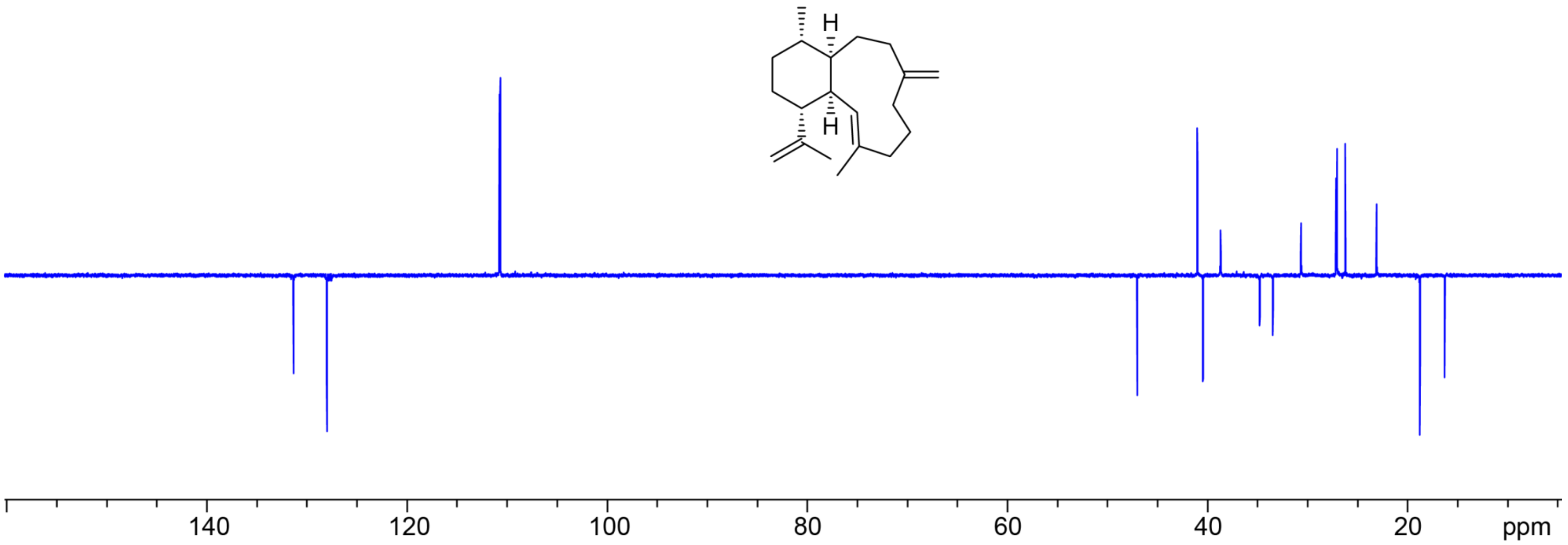

Figure S32. ^{13}C-DEPT135 spectrum of **45** (176 MHz, C_6D_6).

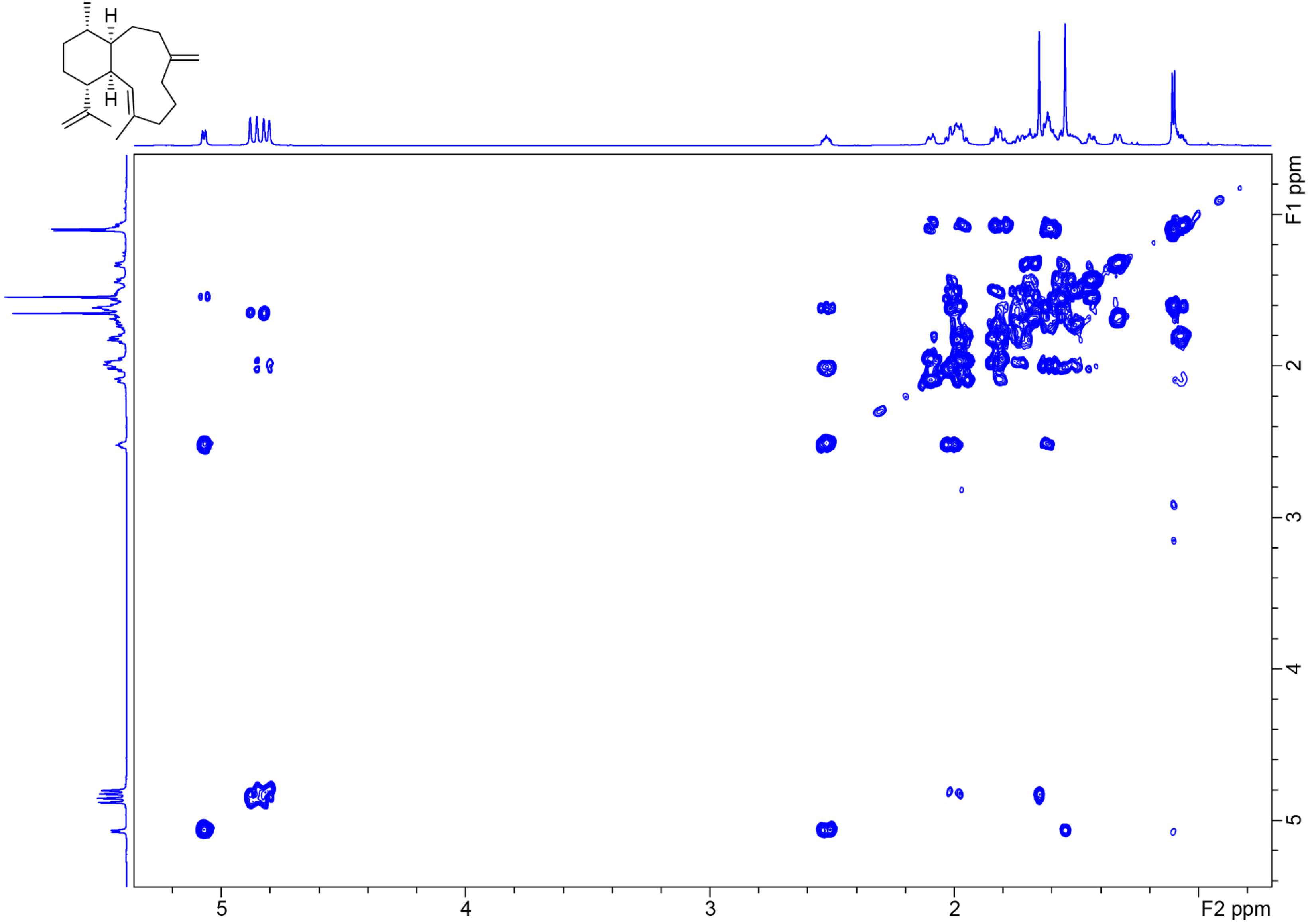

Figure S33. ^{1}H-^{1}H-COSY spectrum (C_6D_6) of **45**.

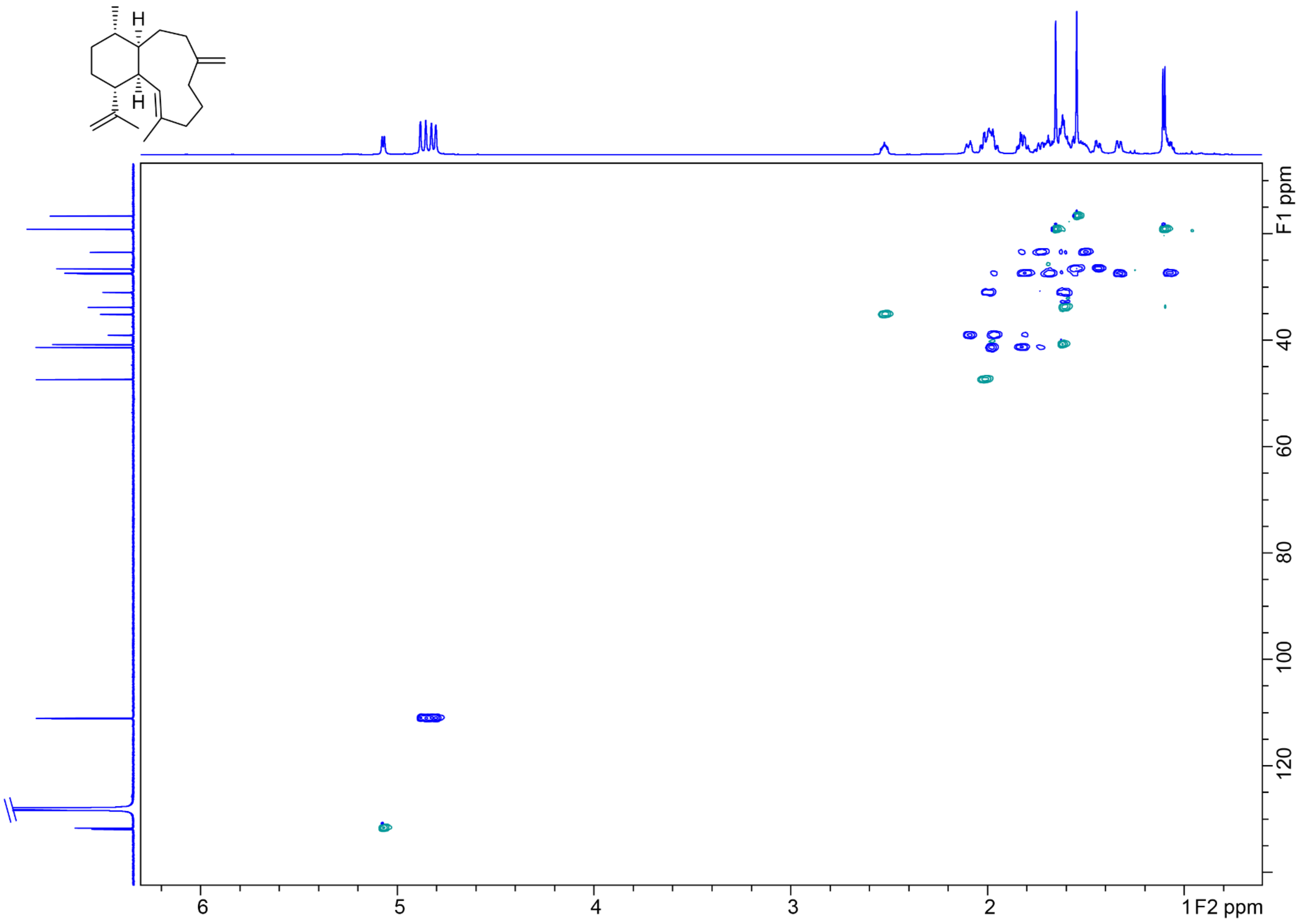

Figure S34. HSQC spectrum (C_6D_6) of **45**.

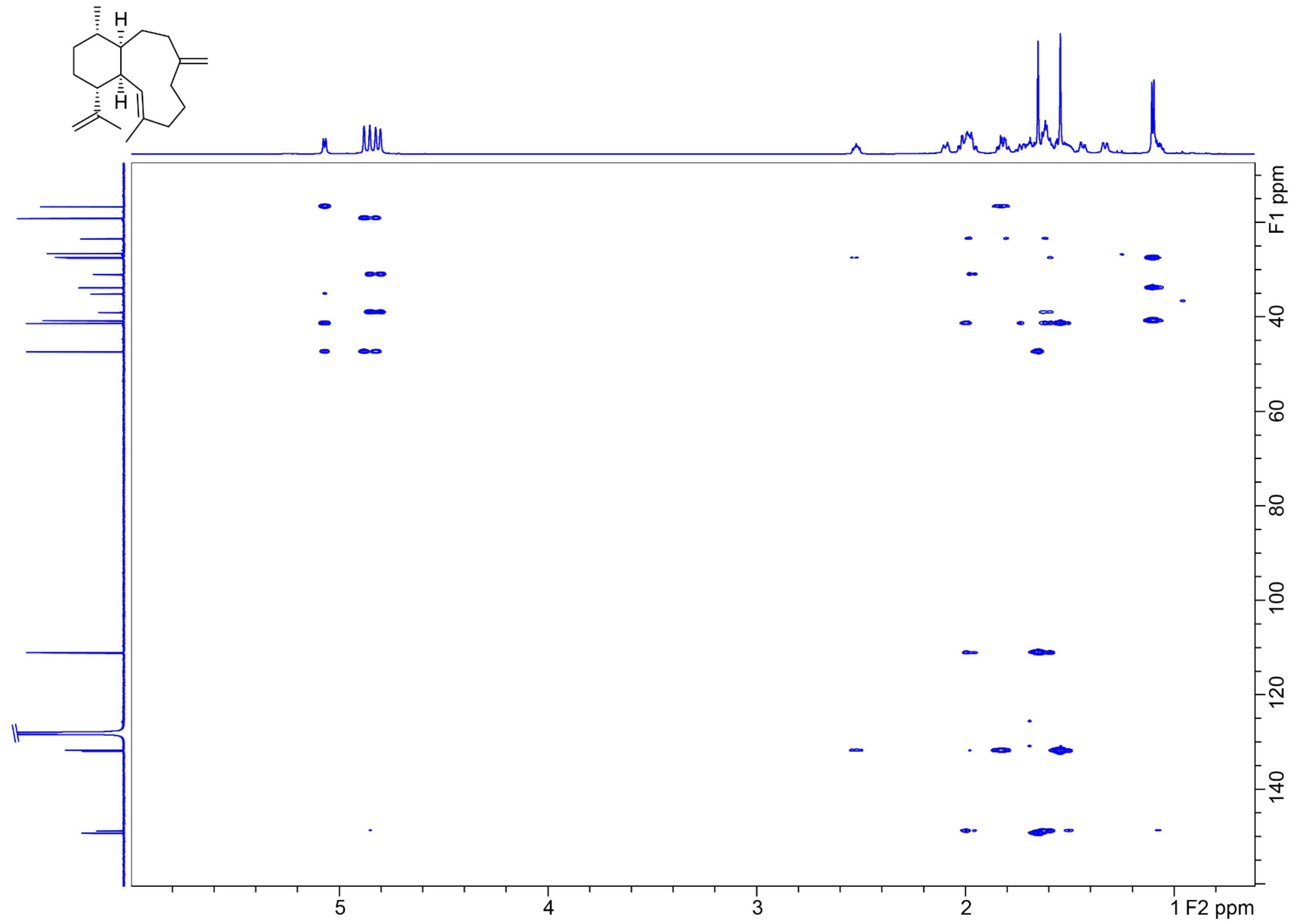

Figure S35. HMBC spectrum (C_6D_6) of **45**.

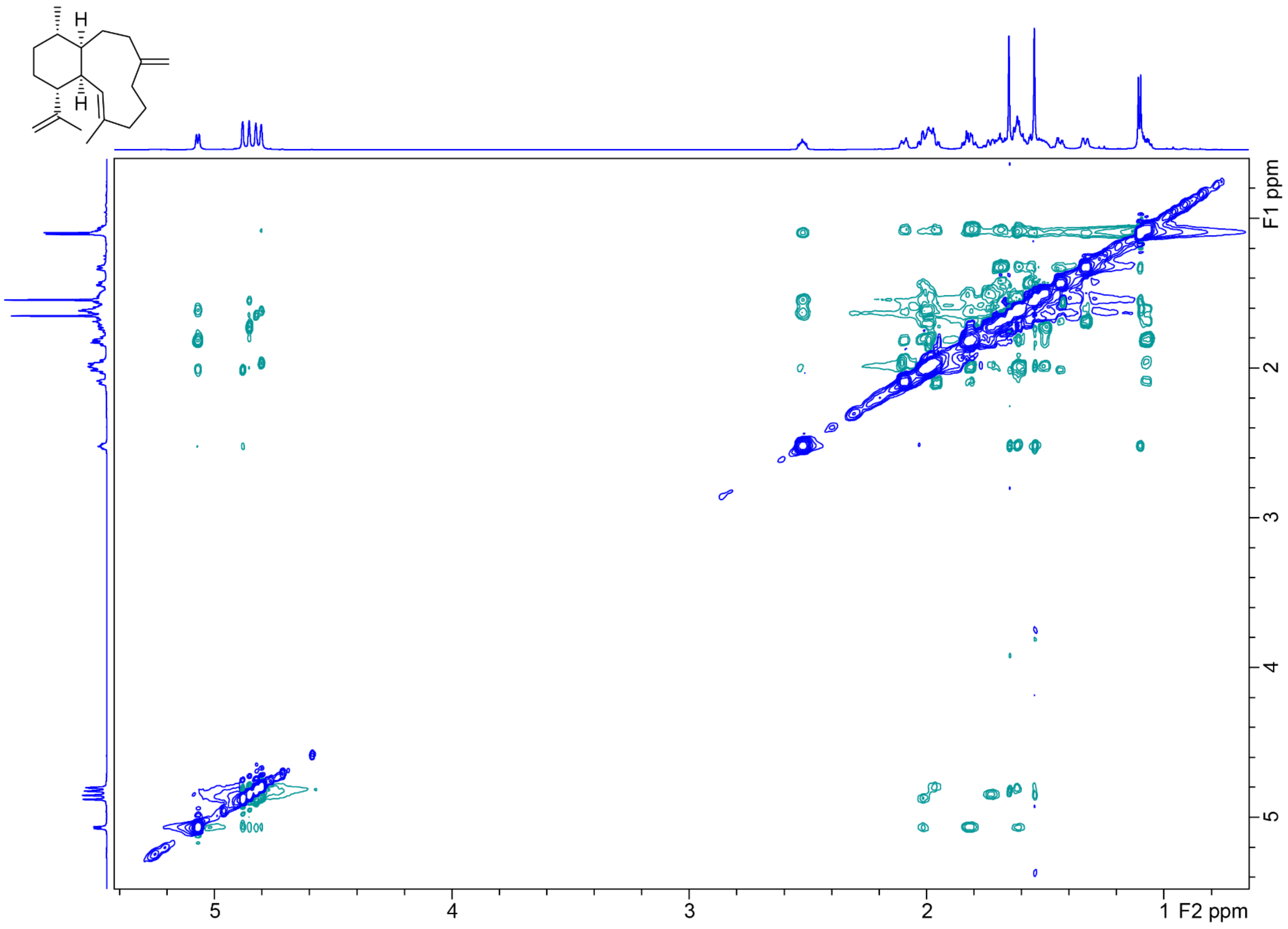

Figure S36. NOESY spectrum (C_6D_6) of **45**.

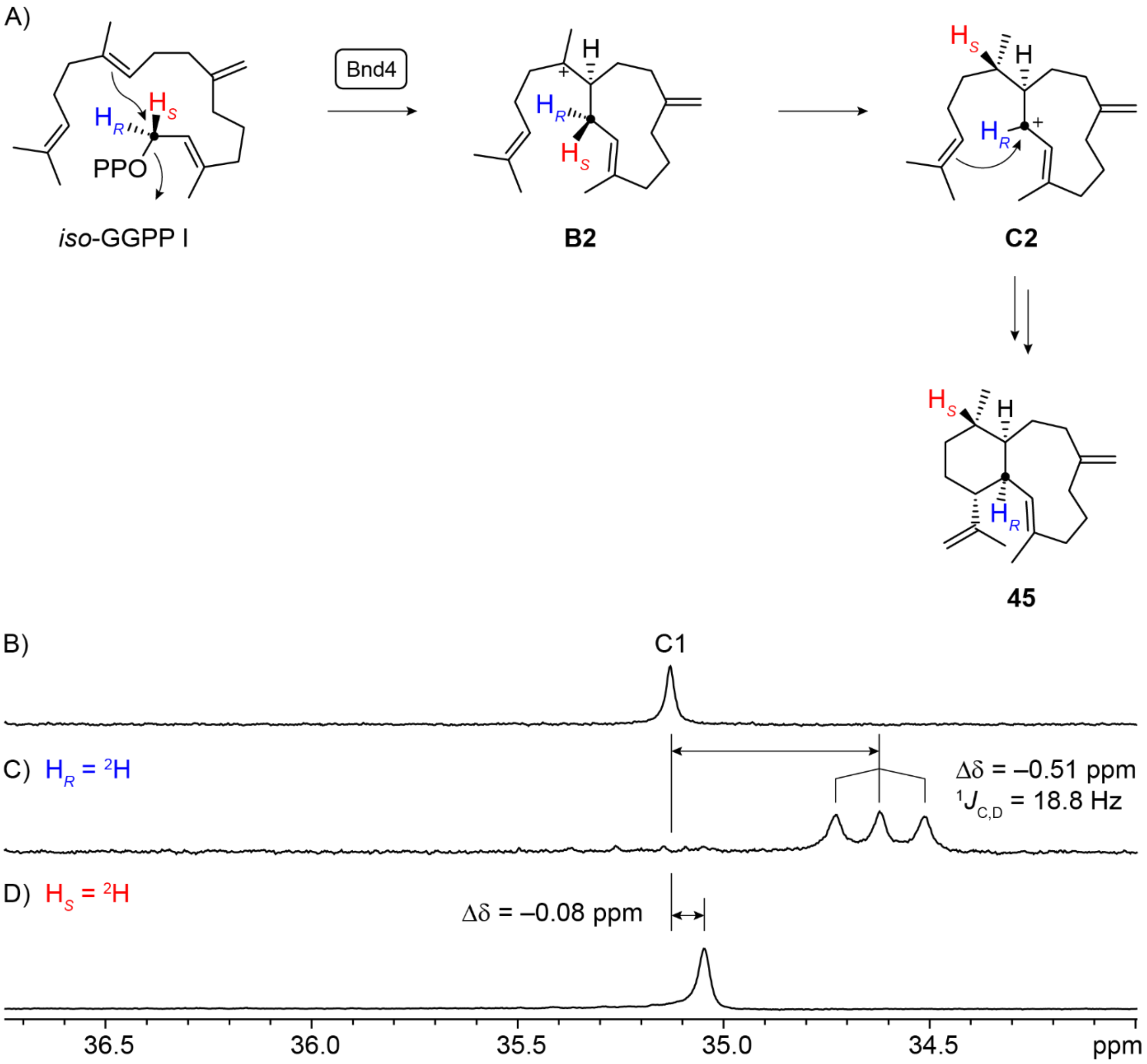

Figure S37. The 1,3-hydride shift from **B2** to **C2** in the biosynthesis of **45**. A) Conversion of (*R*)- (blue hydrogen substituted by ^{2}H) or (*S*)-(1-^{13}C,1-^{2}H)*iso*-GGPP I (red hydrogen substituted by ^{2}H) with Bnd4 into labelled **45**. Partial ^{13}C-NMR spectra of B) unlabelled-**45**, C) labelled **45** obtained from (*R*)-(1-^{13}C,1-^{2}H)*iso*-GGPP I, and D) labelled **45** obtained from (*S*)-(1-^{13}C,1-^{2}H)*iso*-GGPP I. The upfield shited triplet for C1 in D) ($\Delta\delta$ = –0.51 ppm, $^{1}J_{C,D}$ = 18.8 Hz) shows retainment of the 1-*pro-R* hydrogen at C1, while the upfield shifted singlet for C1 in E) ($\Delta\delta$ = -0.08 ppm) confirms migration of the 1-*pro-S* hydrogen.

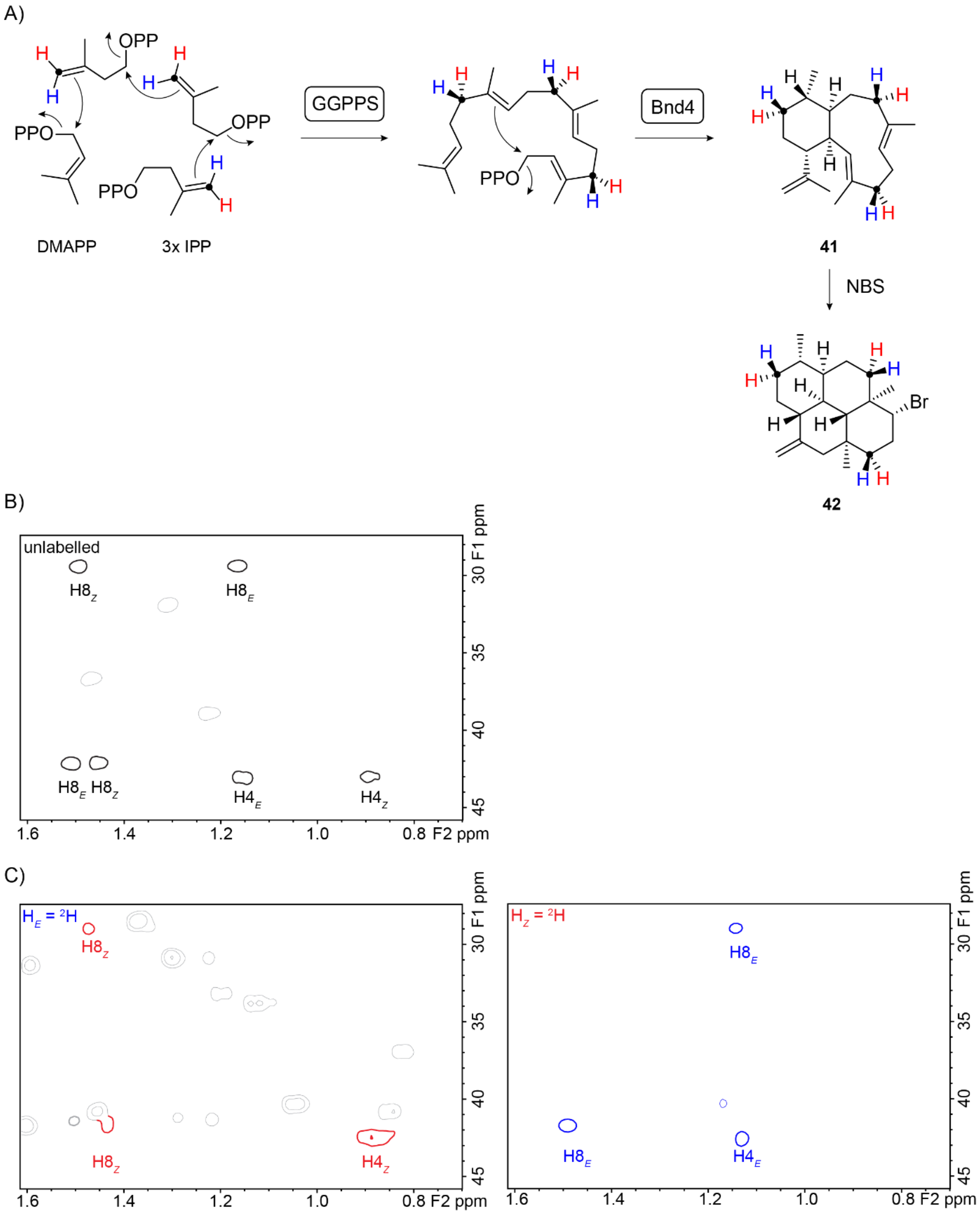

Figure S38. The absolute configuration of **41**. A) Conversion of DMAPP plus (*E*)-(4-^{13}C,4-^{2}H)IPP (red hydrogen substituted by ^{2}H) and (*Z*)-(4-^{13}C,4-^{2}H)IPP (blue hydrogen substituted by ^{2}H) with GGPPS and Bnd4 into labelled **41**, followed by the treatment with NBS to obtain labelled **42**. Partial HSQC spectra of B) unlabelled **42** showing the crosspeaks for the hydrogens at C4, C8 and C12, and C) of labelled **42** obtained from (*E*)- or (*Z*)-(4-^{13}C,4-^{2}H)IPP. In each labelling experiment one of the two crosspeaks is vanished, while the second crosspeak shows slight upfiled shifts in both dimensions (^{1}H and ^{13}C chemical shifts). From the known absolute configuration at the deuterated carbons C4, C8 and C12 the absolute configuration of **42** and consequently of **41** can be inferred. Black dots represent ^{13}C-labelled carbons.

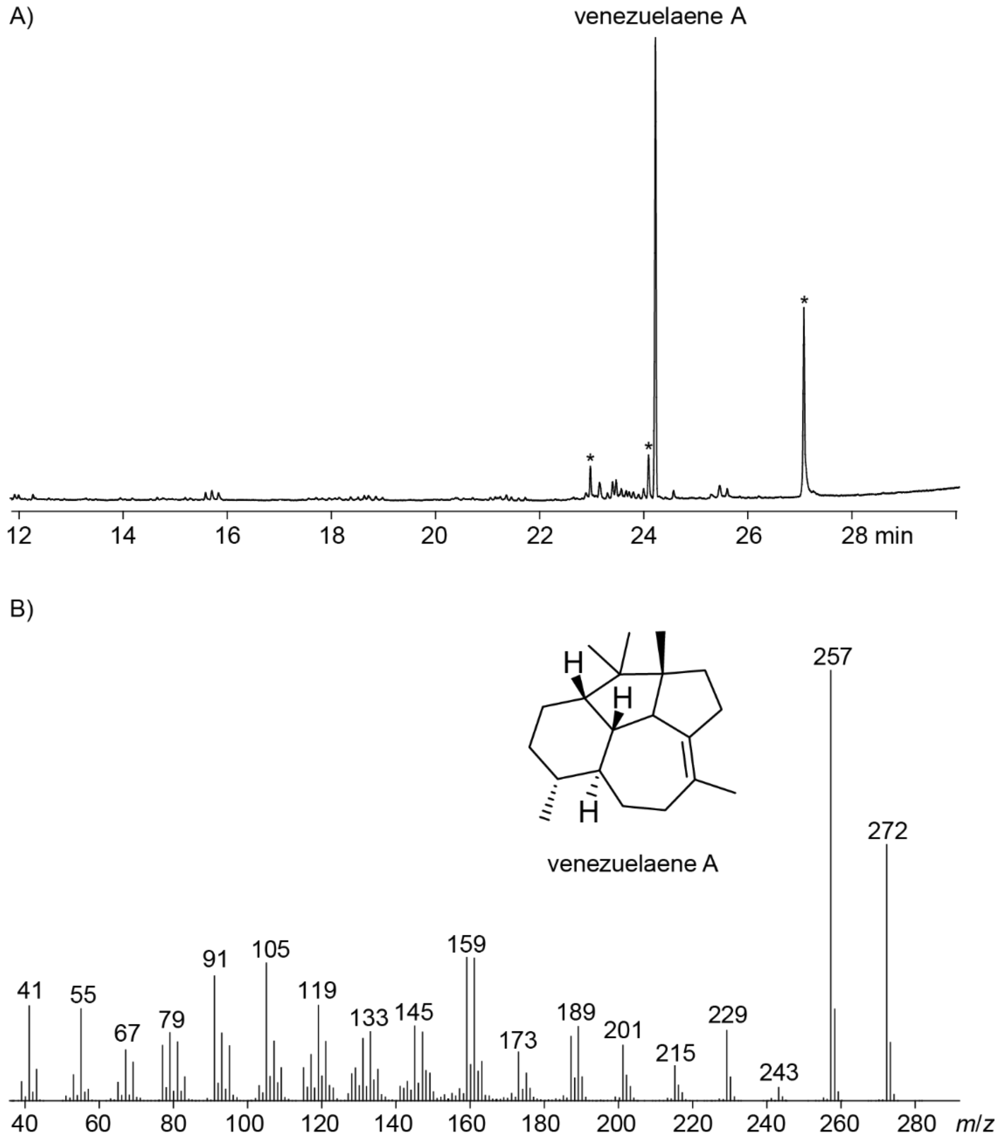

Figure S39. Enzymatic conversion of GGPP with VenA. A) Total ion chromatogram of an extract of the enzyme incubation, B) EI mass spectrum of venezuelaene A.

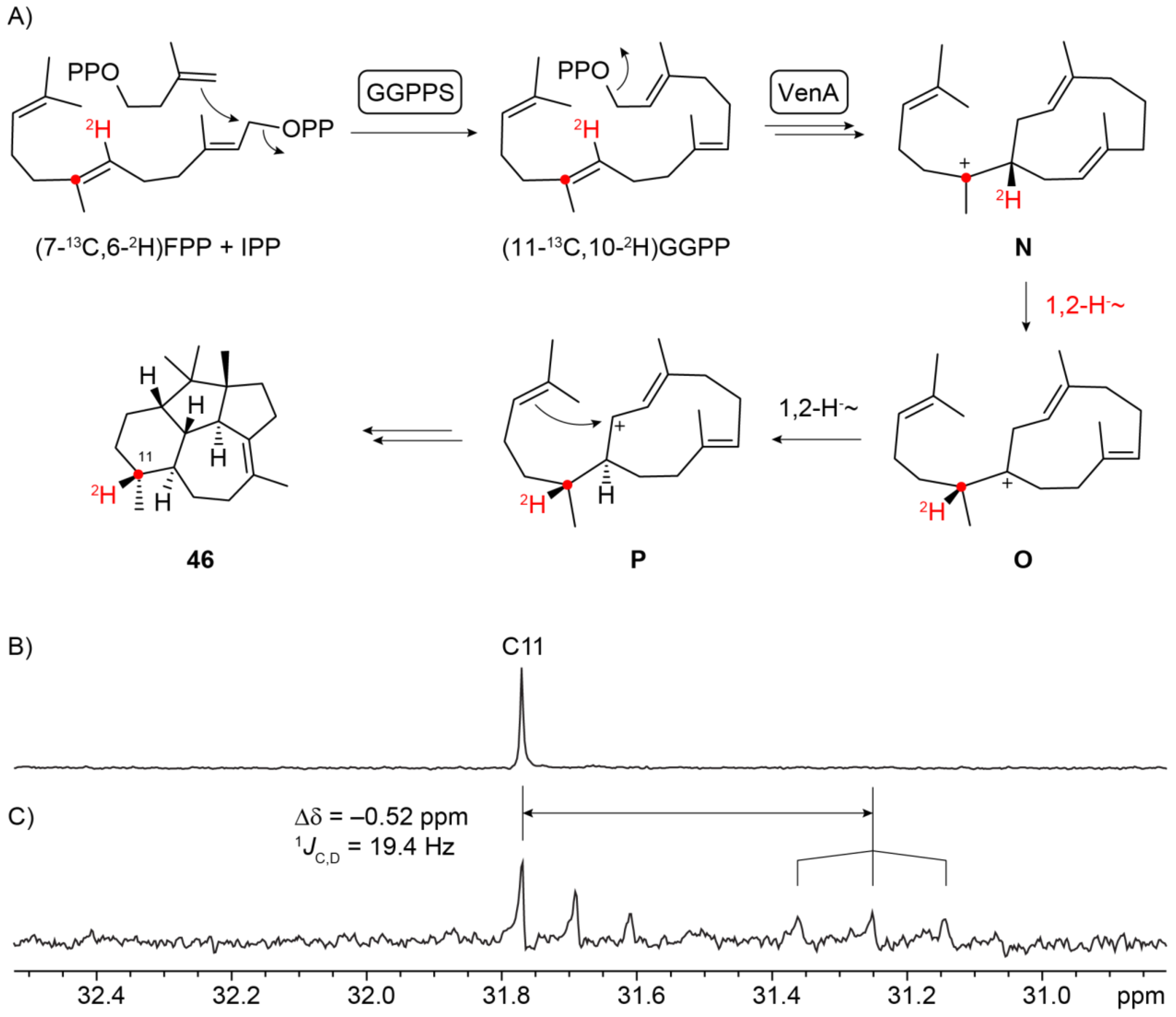

Figure S40. Two sequential 1,2-hydride shift from **N** to **P** in biosynthesis of **46** (Scheme 5A of main text). A) The conversion of (7-^{13}C,6-^{2}H)FPP plus IPP with GGPPS and VenA to **46** via two sequential 1,2-hydride shifts. Partial ^{13}C-NMR spectra showing the region for C11 of B) unlabelled **46** and C) labelled **46** obtained from (7-^{13}C,6-^{2}H)FPP. The upfield shifted triplet for C11 ($\Delta\delta$ = –0.52 ppm, $^{1}J_{C,D}$ = 19.4 Hz) indicates that deuterium migrates from C10 to C11, supporting the 1,2-hydride shift from **O** to **P**. Red dots represent ^{13}C-labelled carbons.

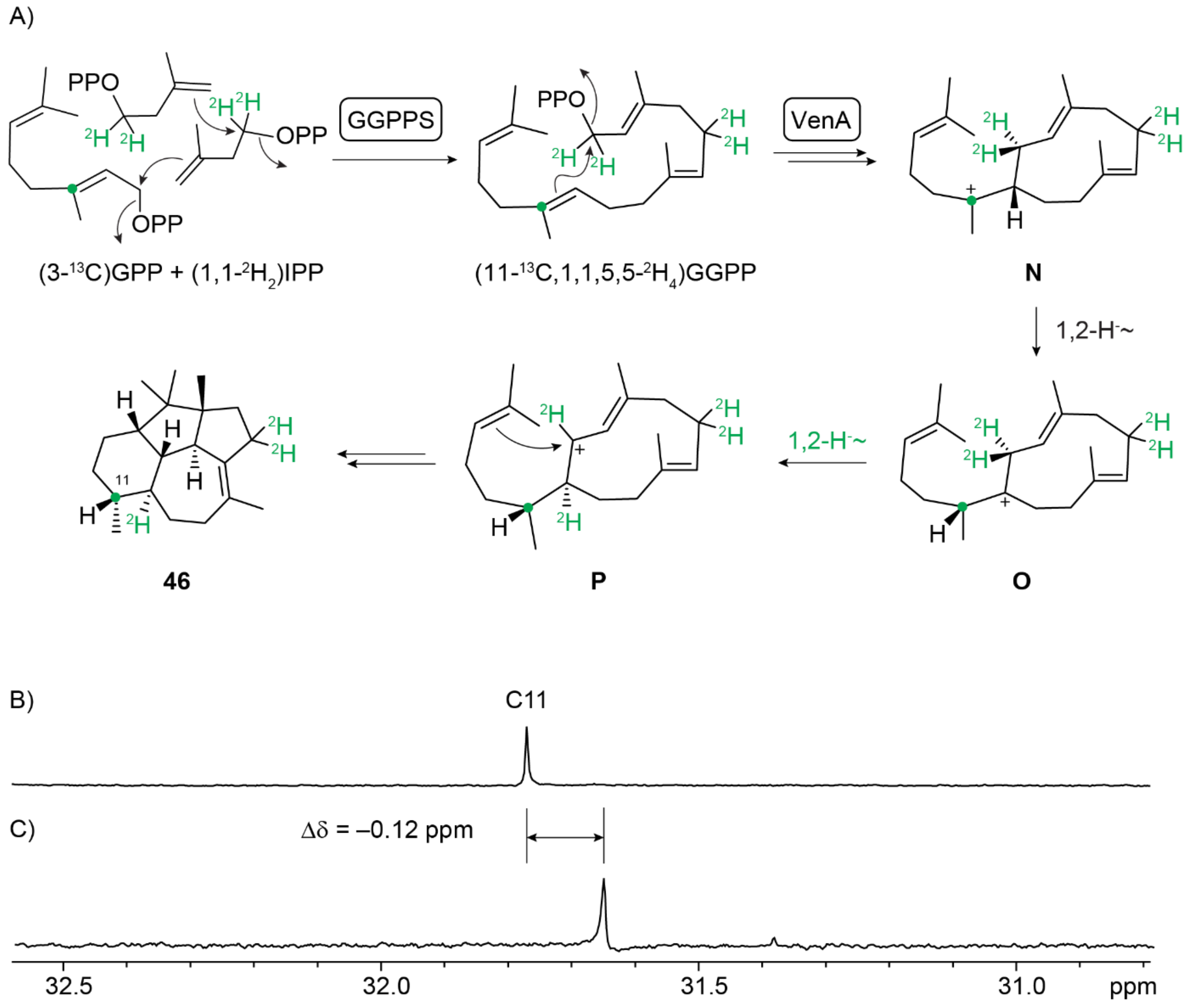

Figure S41. Two sequential 1,2-hydride shift from **N** to **P** in biosynthesis of **46** (Scheme 5A of main text). A) The conversion of (3-^{13}C)GPP plus (1,1-$^{2}H_2$)IPP with GGPPS and VenA into labelled **46**. Partial ^{13}C spectra showing the region for C11 of B) unlabelled **46** and C) labelled **46**. The slight upfield shifted singlet indicates location of deuterium in a neigbouring position (C10). Green dots represent ^{13}C-labelled carbons.

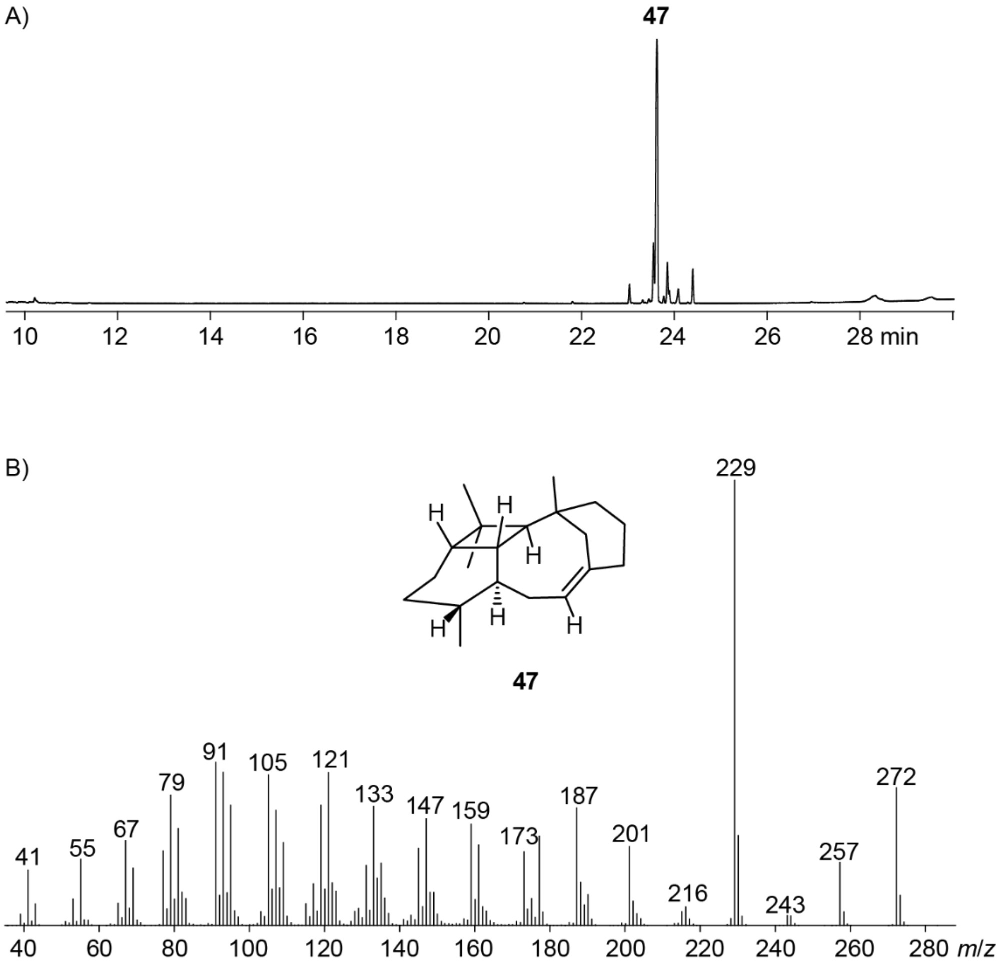

Figure S42. Enzymatic conversion of *iso*-GGPP I with VenA. A) Total ion chromatogram of extract, B) EI mass spectrum of **47**.

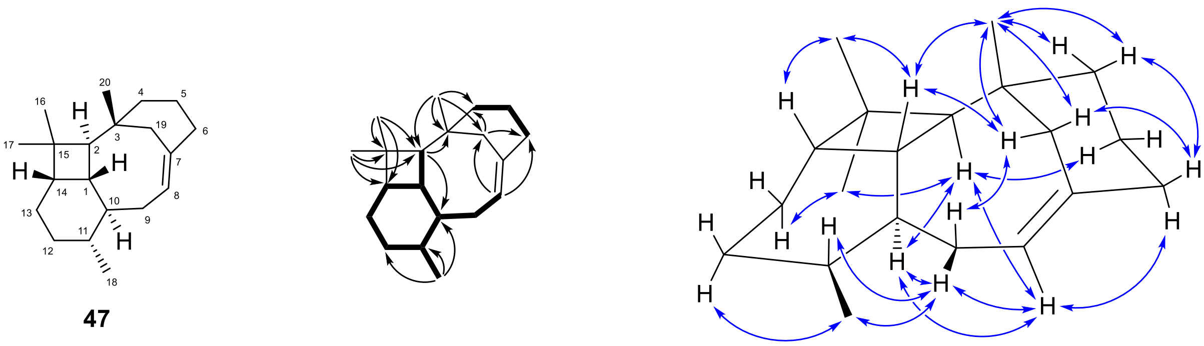

Figure S43. Structure elucidation of venezuelaxenene (**47**). Bold: ^{1}H,^{1}H-COSY, single headed arrows: key HMBC, and blue double headed arrows: NOESY correlations.

Table S8. NMR data of venezuelaxenene (**47**) in C_6D_6 recorded at 298 K.

C[a]	type	^{13}C[b]	^{1}H[b]
1	CH	37.67	1.69 (ddd, J = 10.5, 10.5, 7.7)
2	CH	51.86	2.45 (d, J = 7.5)
3	C_q	40.68	–
4	CH_2	37.71	1.43 (m, H_α)<br>1.22 (m, H_β)
5	CH_2	25.85	1.77 (m, H_α)<br>1.61 (m, H_β)
6	CH_2	36.39	2.12 (m, H_α)<br>1.92 (ddd, J = 12.5, 12.5, 5.5, H_β)
7	C_q	139.59	–
8	CH	121.08	5.35 (dd, J = 7.8, 7.8)
9	CH_2	30.94	2.09 (m, H_α)<br>1.78 (m, H_β)
10	CH	47.83	1.14 (m)
11	CH	31.62	1.17 (m)
12	CH_2	31.02	1.51 (m, H_β)<br>1.26 (m, H_α)
13	CH_2	17.92	1.54 (m, H_α)<br>1.17 (m, H_β)
14	CH	43.70	1.76 (m)
15	C_q	38.90	–
16	CH_3	29.22	1.11 (s)
17	CH_3	29.91	1.05 (s)
18	CH_3	22.00	0.94 (d, J = 6.3)
19	CH_2	43.70	2.57 (ddd, J = 11.8, 2.0, 2.0, H_X)<br>1.25 (m, H_Y)
20	CH_3	27.29	1.07 (s)

[a] Carbon numbering as shown in Figure S43. [b] Chemical shifts δ in ppm, multiplicity: s = singlet, d = doublet, m = multiplet, coupling constants J are given in Hertz.

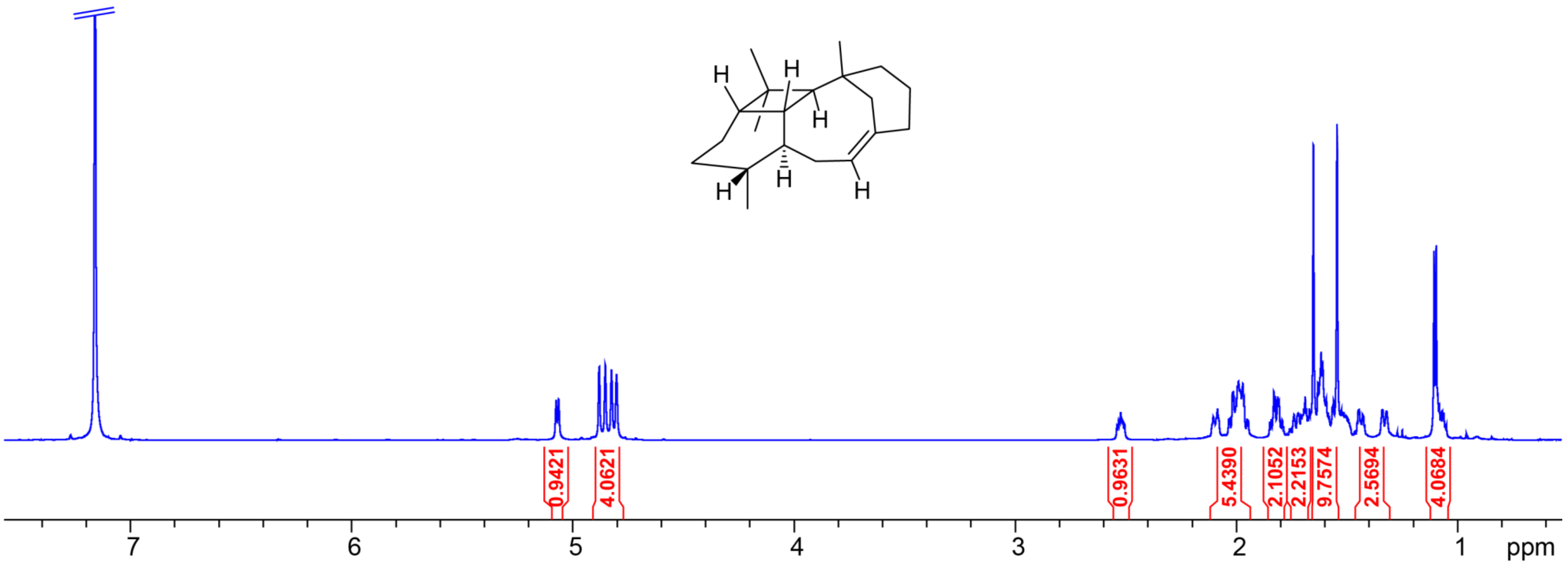

Figure S44. ^{1}H-NMR spectrum of **47** (700 MHz, C_6D_6).

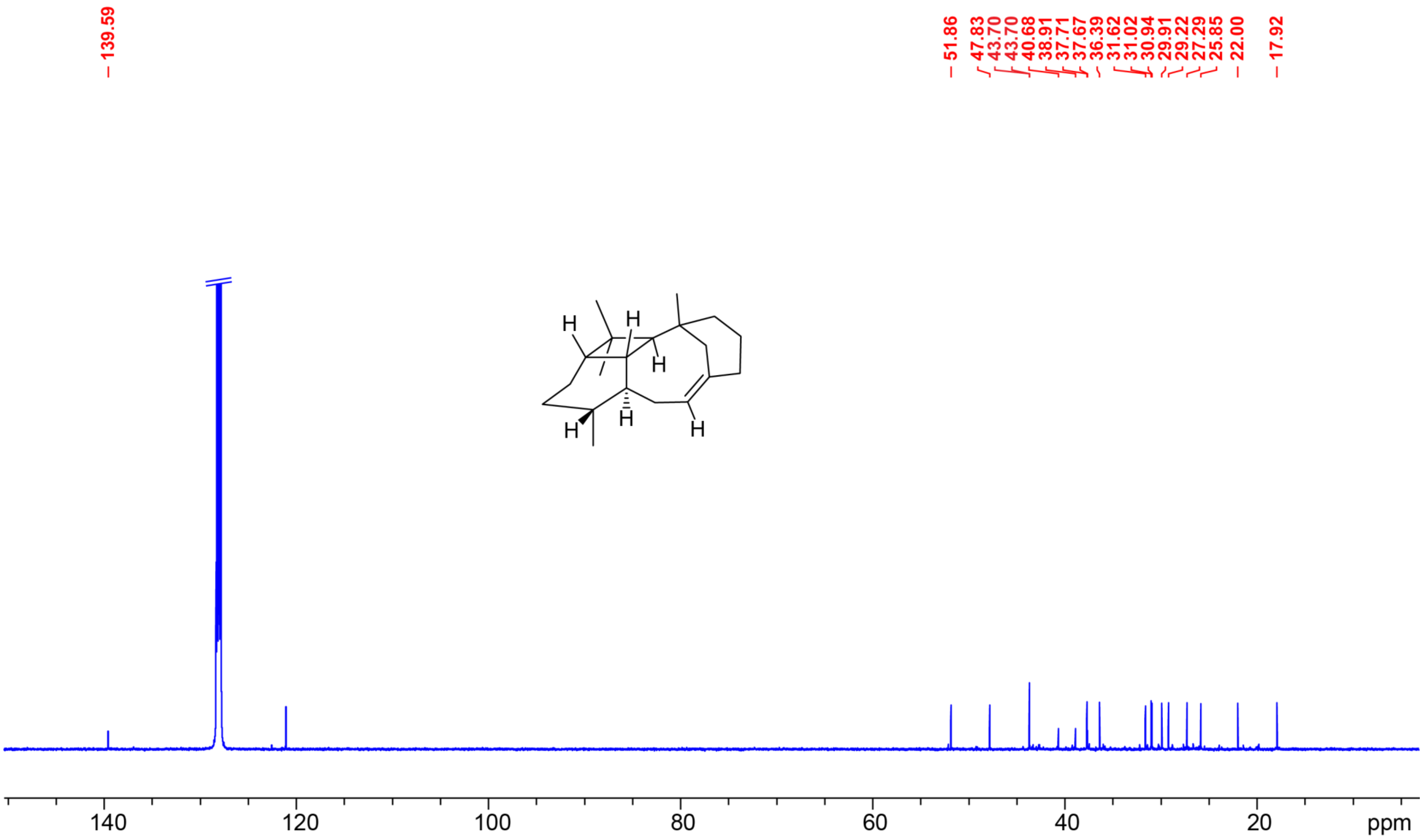

Figure S45. ^{13}C-NMR spectrum of **47** (176 MHz, C_6D_6).

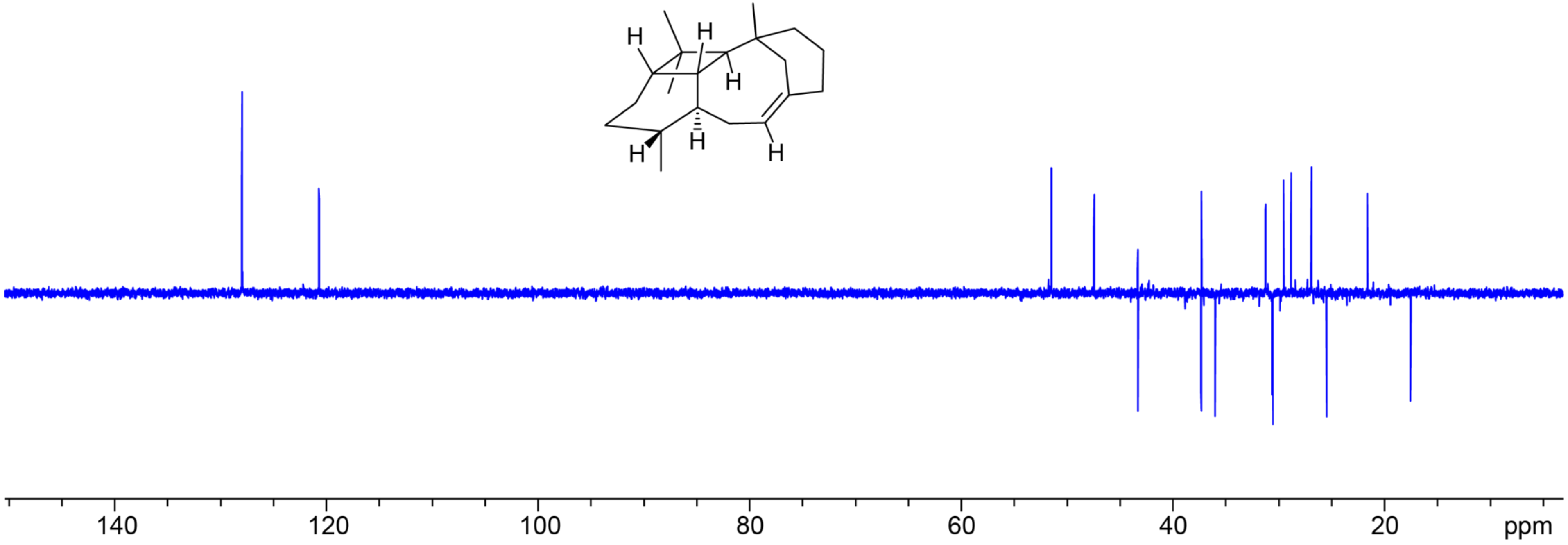

Figure S46. ^{13}C-DEPT135 spectrum of **47** (176 MHz, C_6D_6).

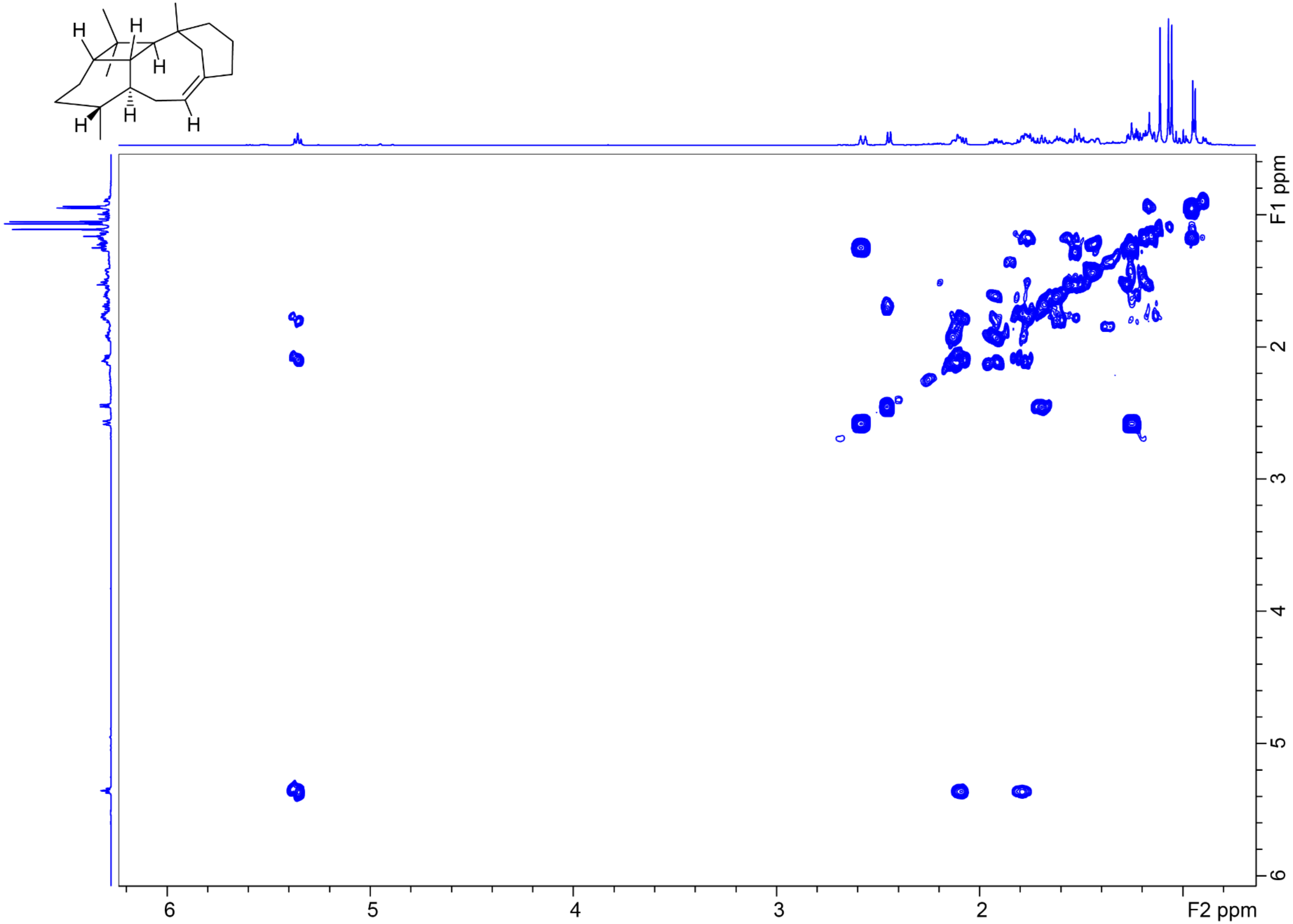

Figure S47. ^{1}H-^{1}H-COSY spectrum (C_6D_6) of **47**.

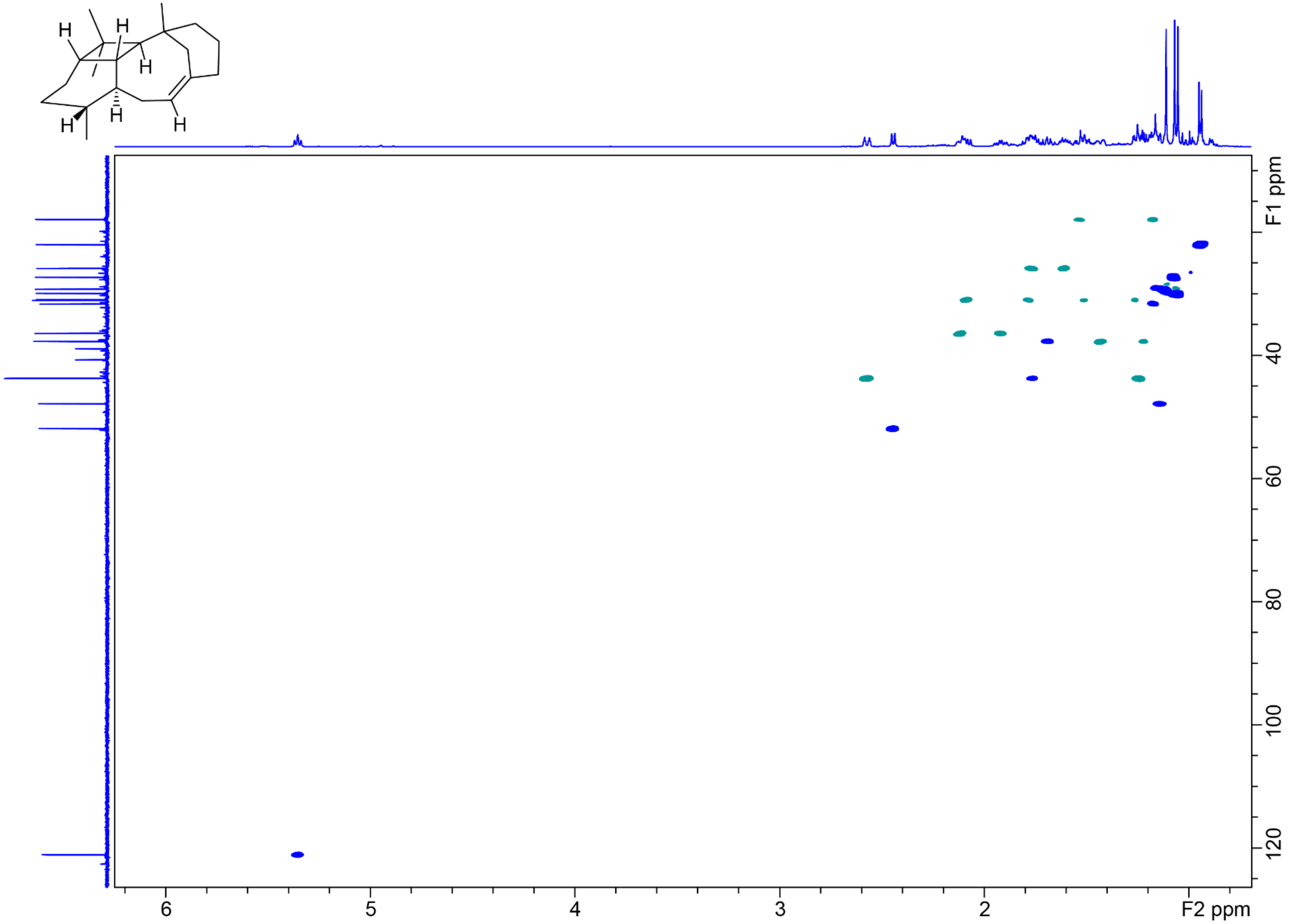

Figure S48. HSQC spectrum (C_6D_6) of **47**.

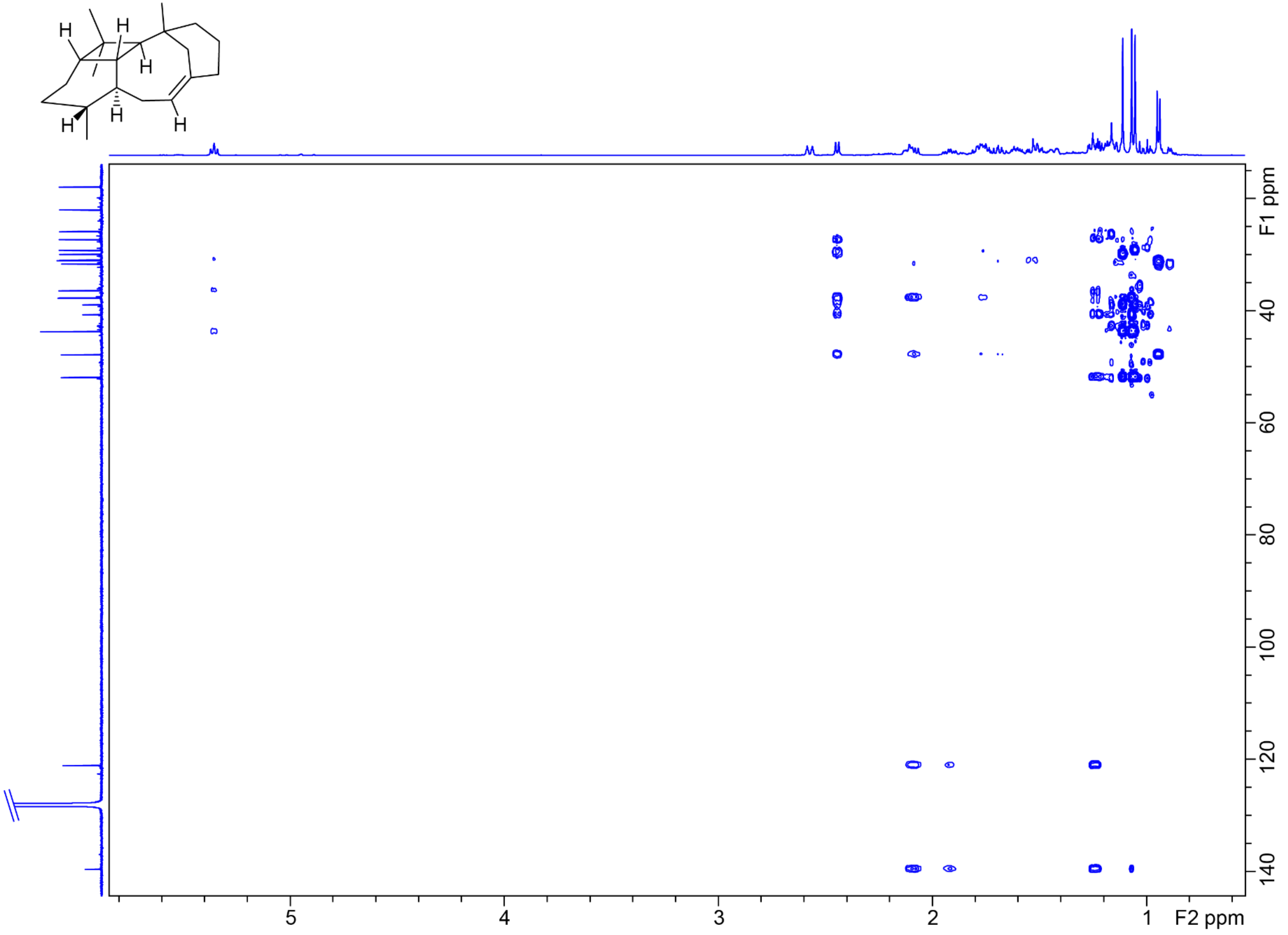

Figure S49. HMBC spectrum (C_6D_6) of **47**.

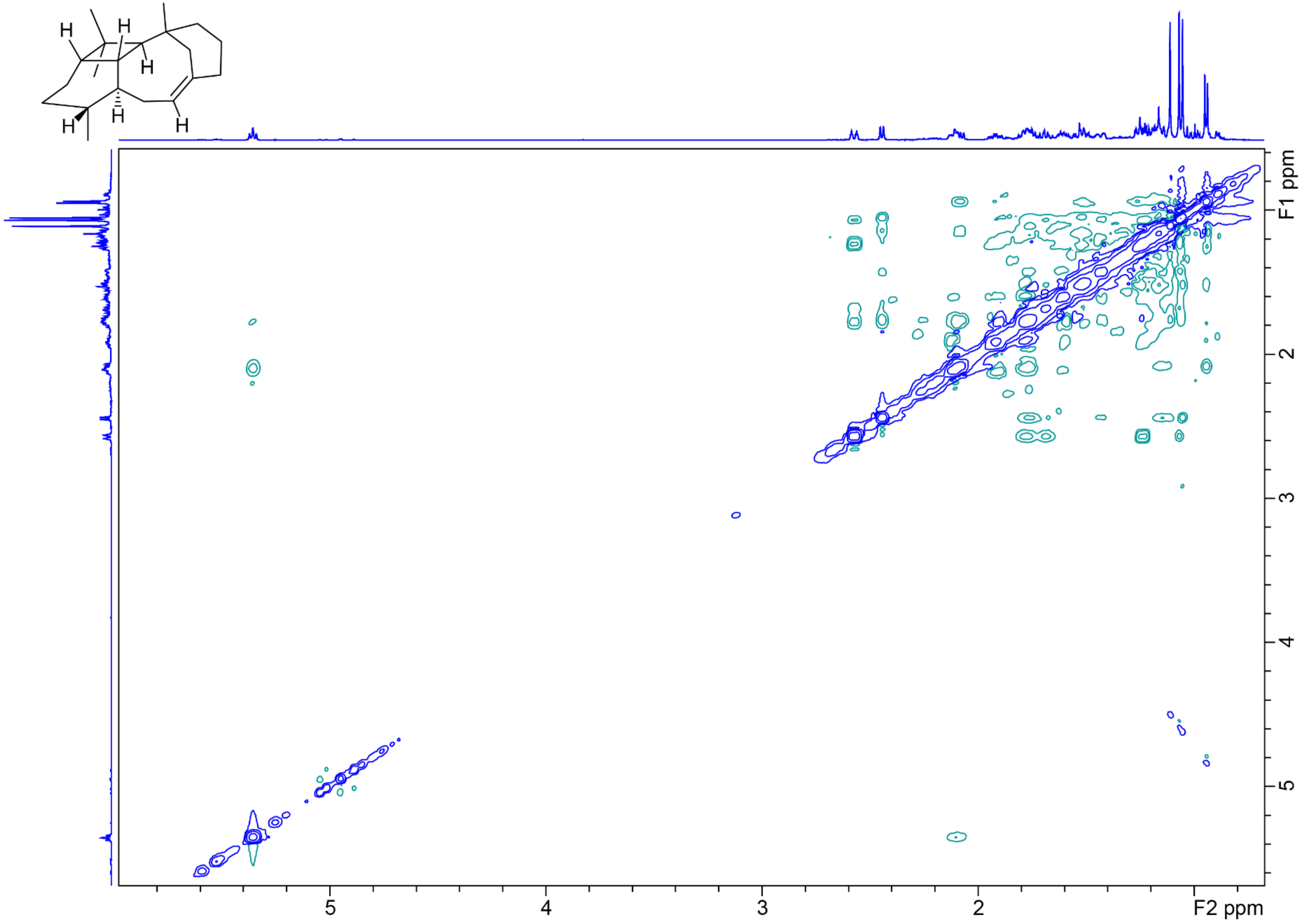

Figure S50. NOESY spectrum (C_6D_6) of **47**.

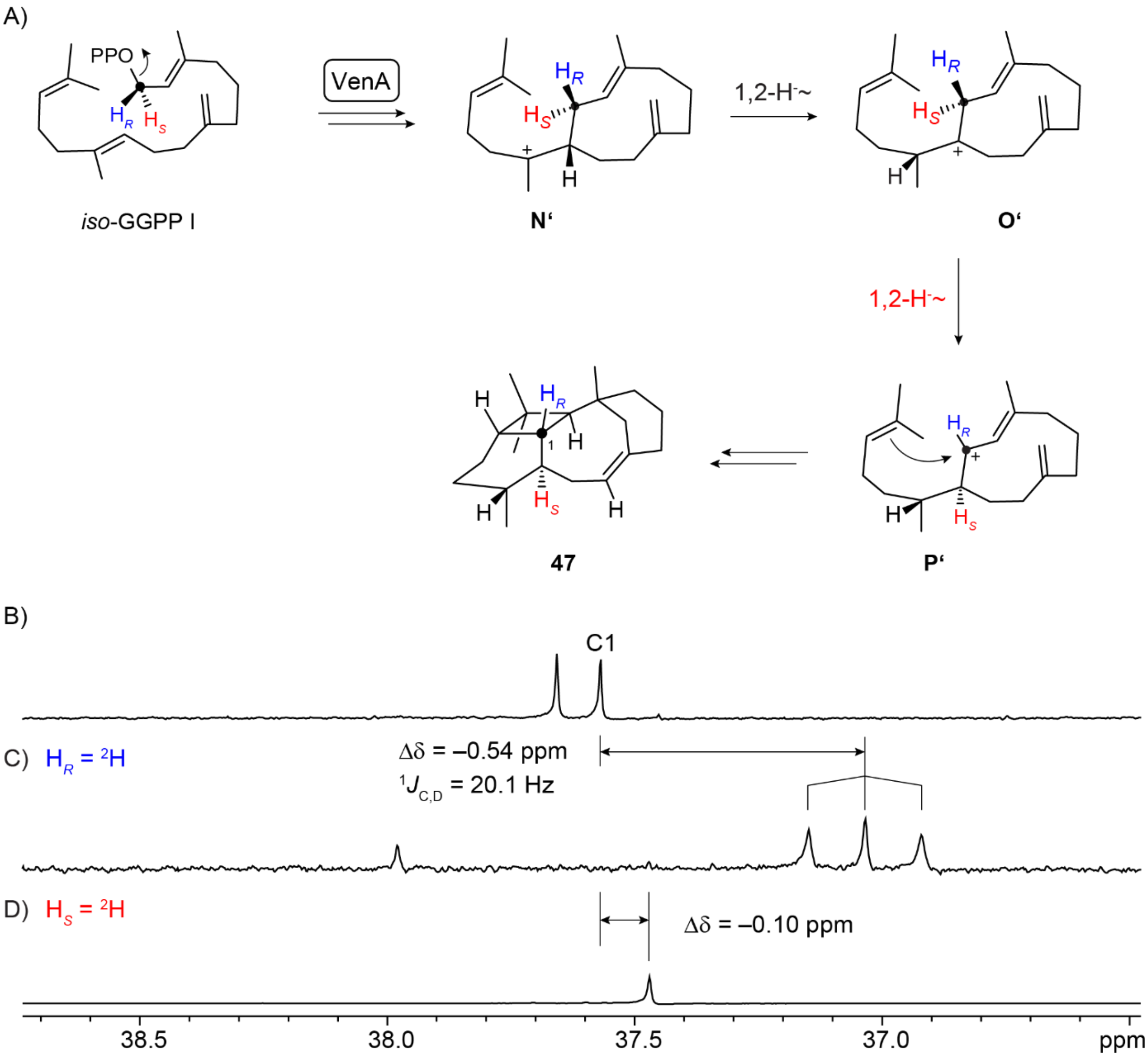

Figure S51. Stereochemical course for the 1,2-hydride shift from **O'** to **P'** in the biosynthesis of **47** by VenA. A) The conversion of (*R*)- (blue hydrogen substituted by ^{2}H) and (*S*)-(1-^{13}C,1-^{2}H)*iso*-GGPP I (red hydrogen substituted by ^{2}H) with Ven A into **47**. Partial ^{13}C spectra showing the region for C1 of B) unlabelled **47**, C) labelled **47** obtained from (*R*)-(1-^{13}C,1-^{2}H)*iso*-GGPP I, and D) labelled **47** obtained from (*S*)-(1-^{13}C,1-^{2}H)*iso*-GGPP I. The upfield shifted triplet for C1 in C) indicates retainment of the 1-*pro-R* hydrogen at C1, while the slightly upfield shifted singlet in D) indicates migration of the 1-*pro-S* hydrogen into a neighbouring position (C10). Black dots represent ^{13}C-labelled carbons.

Table S9. Results of DFT calculations for the cyclisation of *iso*-GGPP I to **47** (Scheme 5B of main text).

Structure	Gibbs energy (298.15K) in Hartree	energy relative to E or G in kcal/mol	reaction barrier in kcal/mol	Gibbs free energy in kcal/mol
**M’**	–781.673512	0.00		
**M’-N’-TS**	–781.676064	–1.60	–1.60	
**N’**	–781.681337	–4.91		–4.91
**N’**	–781.682447	–5.61		
**N’-O’-TS**	–781.676536	–1.90	3.71	
**O’**	–781.687942	–9.05		–3.45
**O’**	–781.695302	–13.67		
**O’-P’-TS**	–781.668771	2.98	16.65	
**P’**	–781.687363	–8.69		4.98
**P’**	–781.699057	–16.03		
**P’-Q’-TS**	–781.701393	–17.50	–1.47	
**Q’**	–781.706428	–20.66		–4.63
**Q’**	–781.706428	–20.66		
**Q’-R’-TS**	–781.697257	–14.90	5.75	
**R’**	–781.720212	–29.30		–8.65
**R’**	–781.720199	–29.30		
**R’-S’-TS**	–781.721144	–29.89	–0.59	
**S’**	–781.730189	–35.57		–6.27

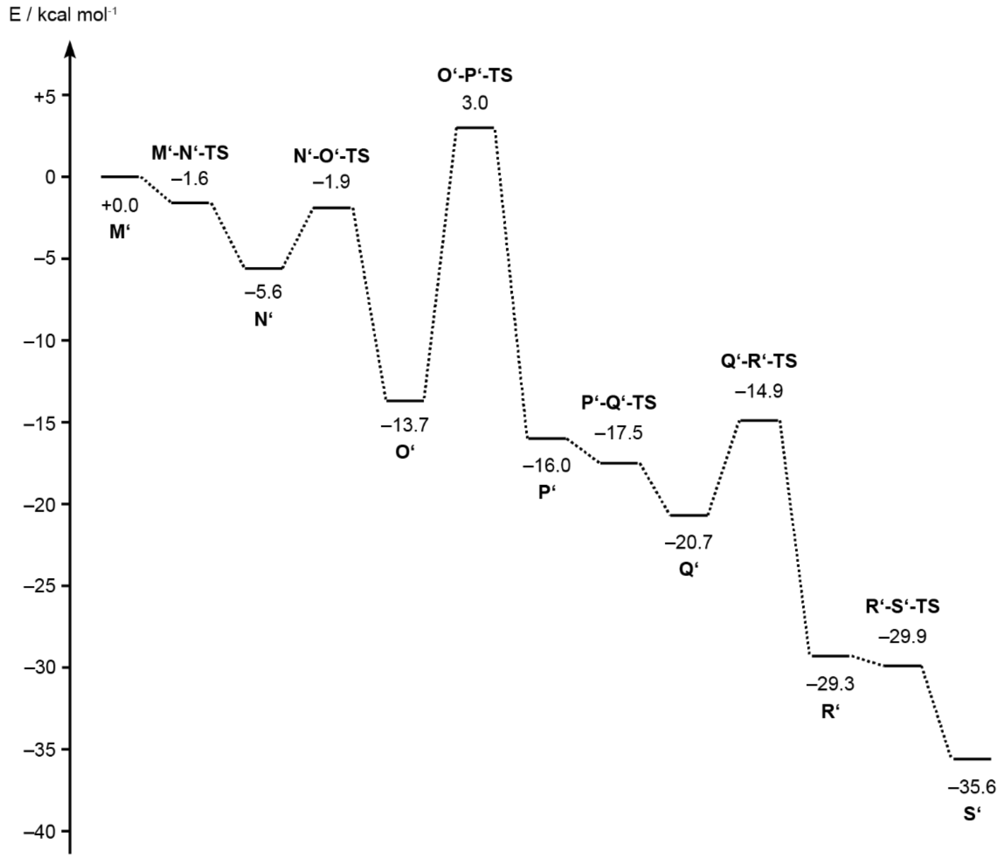

Figure S52. Energy profile for the cyclisation of *iso*-GGPP I to **47** (Scheme 5B of main text, Table S9). For intermediates for which conformers of different energy were found in succeeding steps, always the conformer of lowest energy is shown.

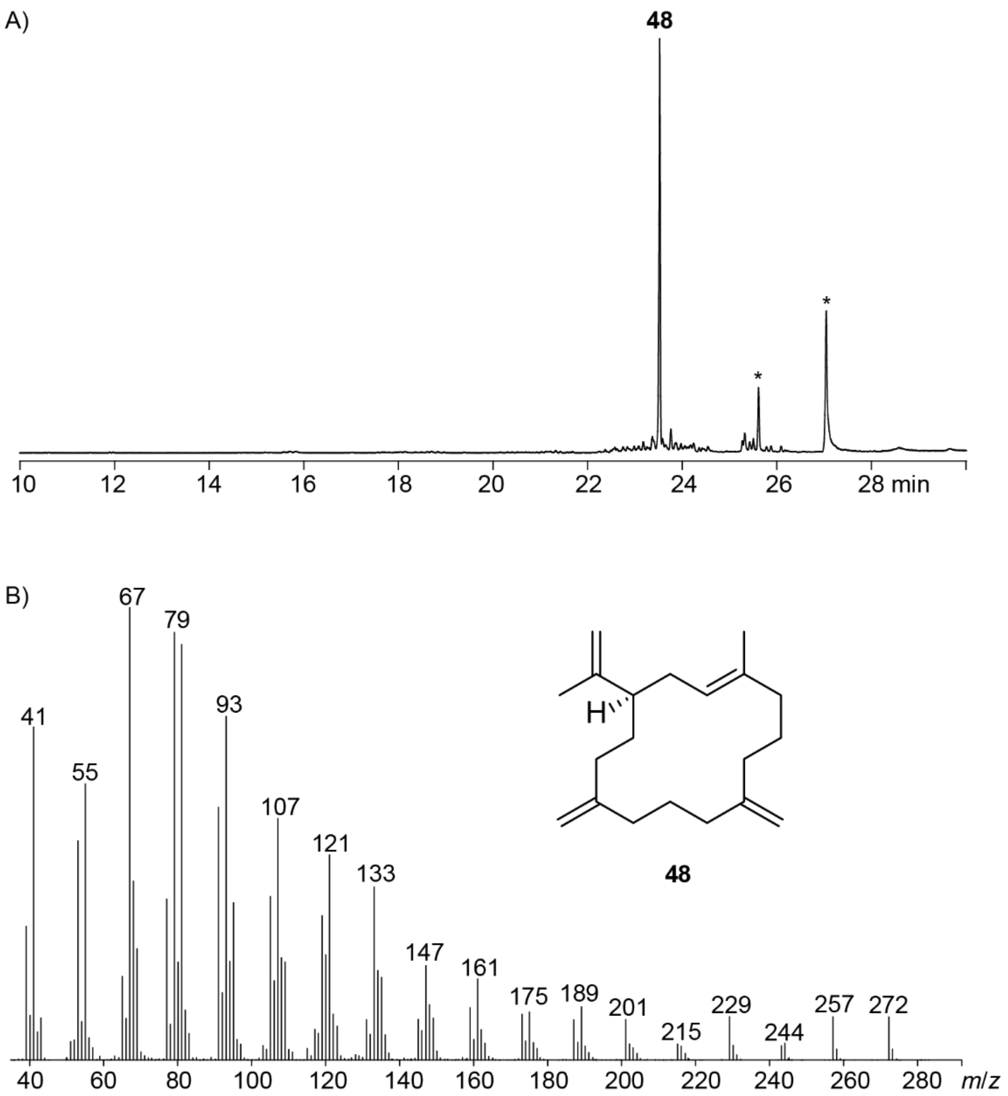

Figure S53. Enzymatic conversion of *iso*-GGPP IV with HdS. A) Total ion chromatogram of an extract of the enzyme incubation, B) EI mass spectrum of **48**. Asterisks indicate spontaneous lysis and hydrolysis products of *iso*-GGPP IV and contaminants such as plasticisers.

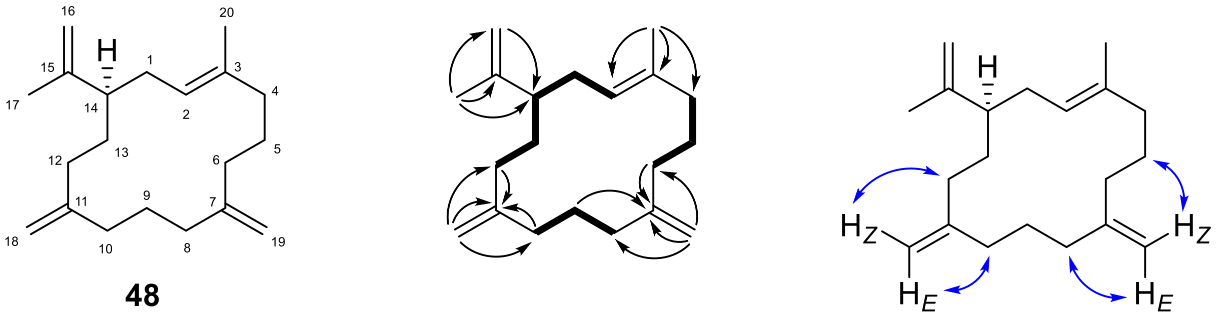

48

Figure S54. Structure elucidation of diisocembrene A (**48**). Bold: ^{1}H,^{1}H-COSY, single headed arrows: key HMBC, and blue double headed arrows: NOESY correlations.

Table S10. NMR data of diisocembrene A (**48**) in C_6D_6 recorded at 298 K.

C[a]	type	^{13}C[b]	^{1}H[b]
1	CH_2	33.54	2.09 (m)
			2.03 (m)
2	CH	125.90	5.07 (ddq, J = 9.5, 5.3, 1.2)
3	C_q	135.36	–
4	CH_2	38.82	2.05 (m)
			2.01 (m)
5	CH_2	23.34	1.55 (m, 2H)
6	CH_2	30.80	1.96 (m)
			1.81 (m)
7	C_q	148.67	–
8	CH_2	37.49	2.13 (m)
			2.10 (m)
9	CH_2	25.46	1.48 (m)
			1.45 (m)
10	CH_2	34.36	1.97 (m)
			1.92 (m)
11	C_q	149.74	–
12	CH_2	34.12	2.15 (m)
			2.00 (m)
13	CH_2	28.03	1.66 (dddd, J = 13.8, 9.4, 7.6, 3.1)
			1.19 (dddd, J = 13.8, 10.6, 9.0, 5.0)
14	CH	47.16	1.98 (m)
15	C_q	148.32	–
16	CH_2	111.00	4.85 (m, 2H)
17	CH_3	19.49	1.61 (m)
18	CH_2	111.22	4.83 (m, H_Z)
			4.82 (m, H_E)
19	CH_2	109.72	4.86 (m, H_E)
			4.85 (m, H_Z)
20	CH_3	15.91	1.47 (br s)

[a] Carbon numbering as shown in Figure S54. [b] Chemical shifts δ in ppm, multiplicity: s = singlet, d = doublet, q = quartet, m = multiplet, br = broad, coupling constants J are given in Hertz.

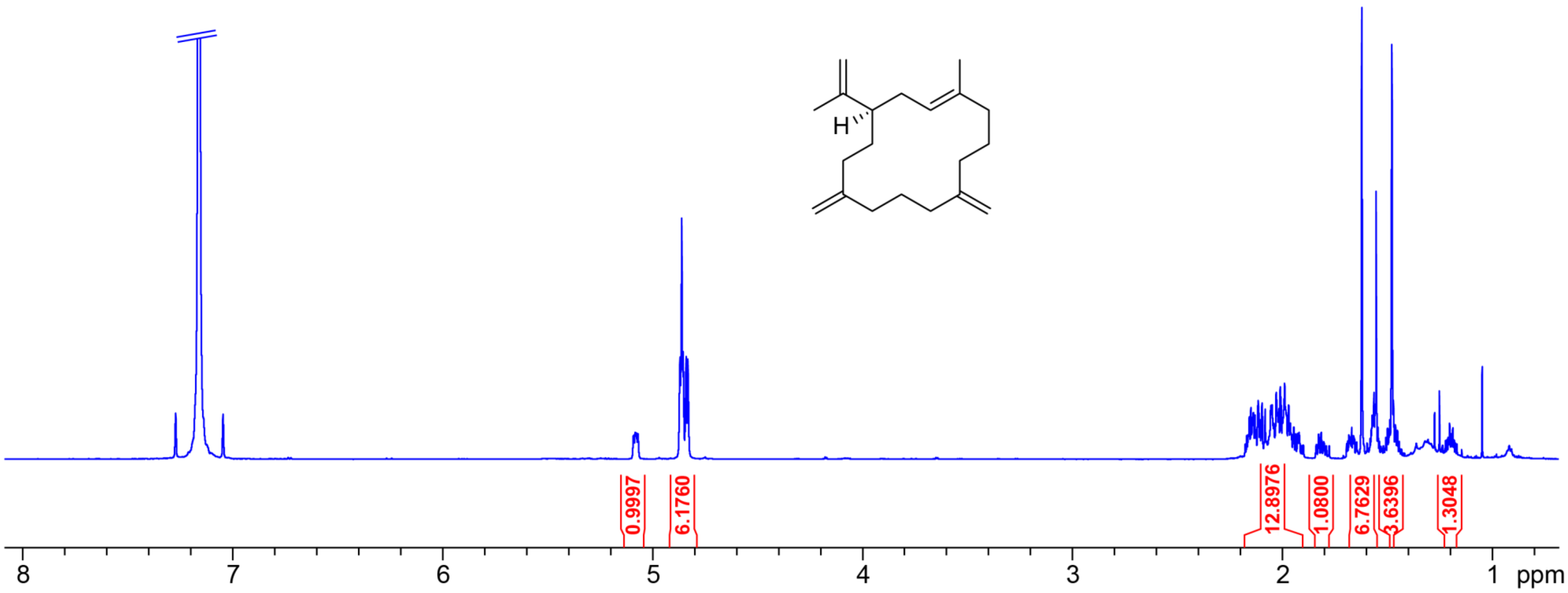

Figure S55. ^{1}H-NMR spectrum of **48** (700 MHz, C_6D_6).

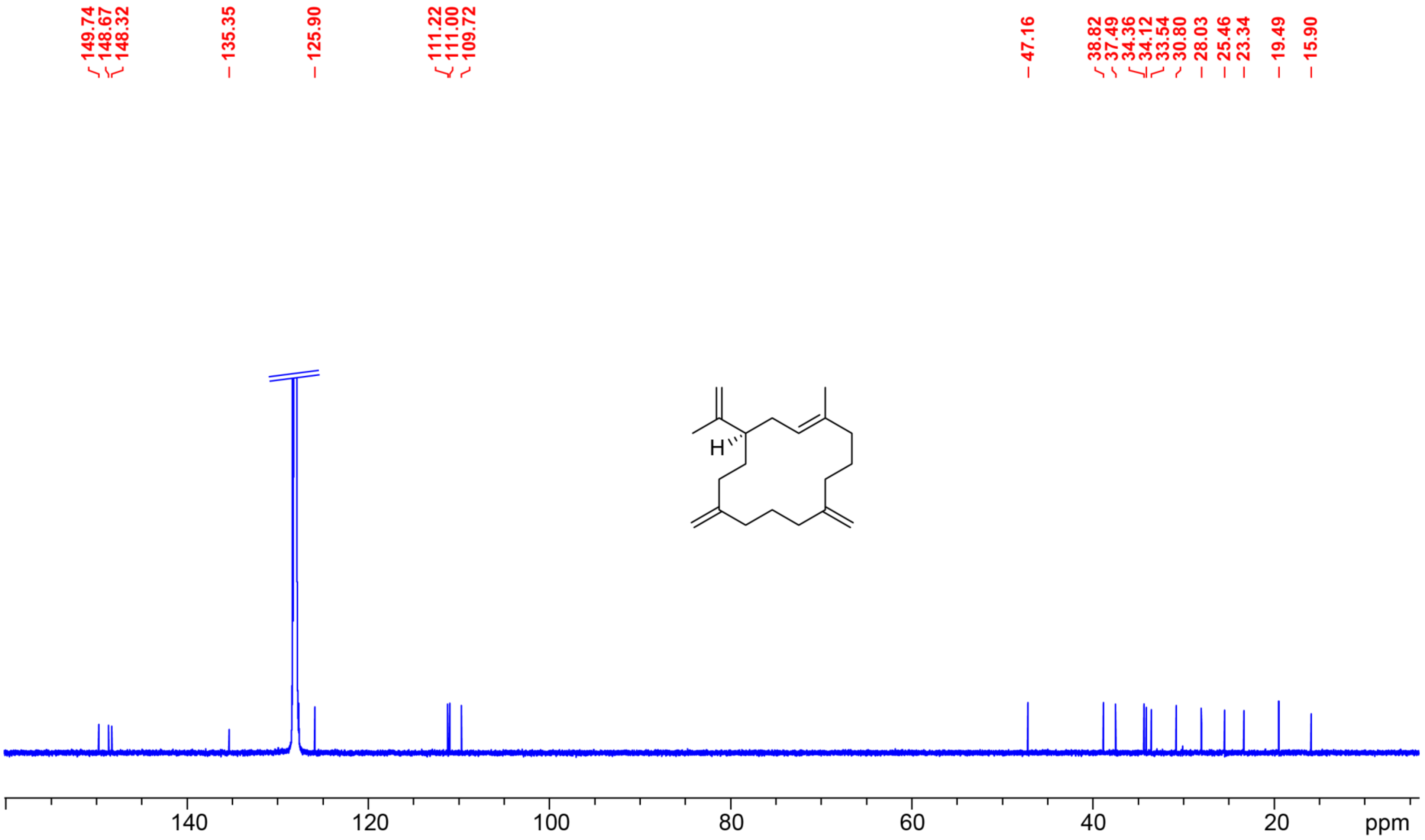

Figure S56. ^{13}C-NMR spectrum of **48** (176 MHz, C_6D_6).

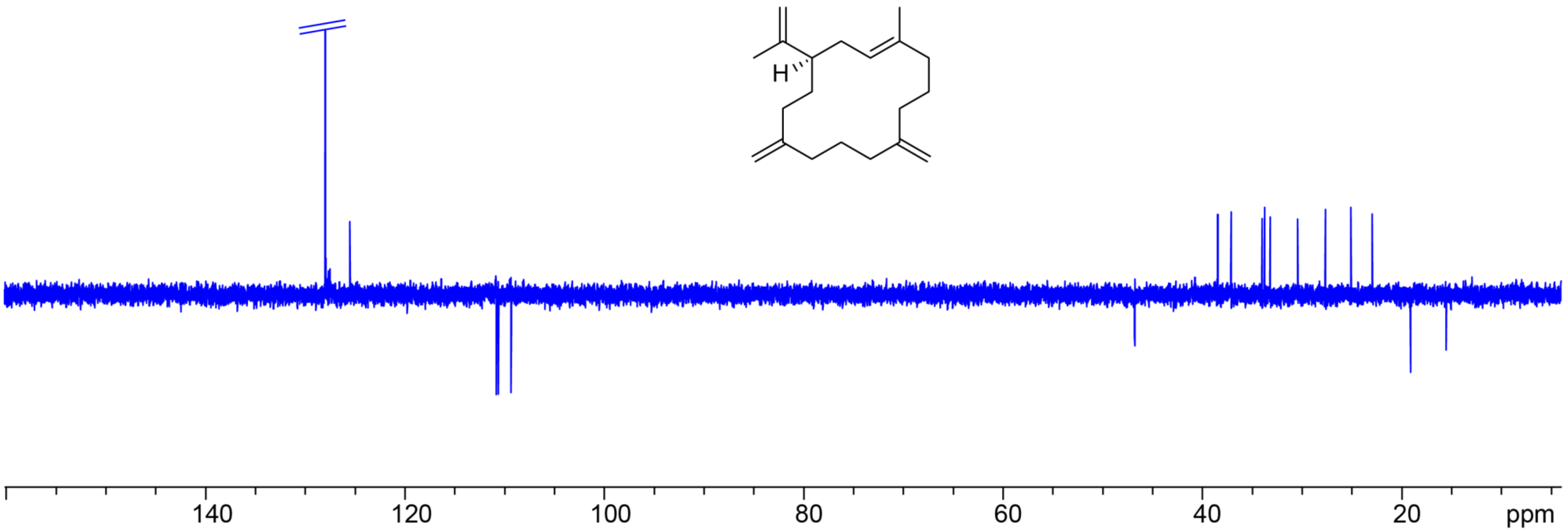

Figure S57. ^{13}C-DEPT135 spectrum of **48** (176 MHz, C_6D_6).

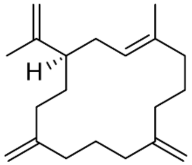

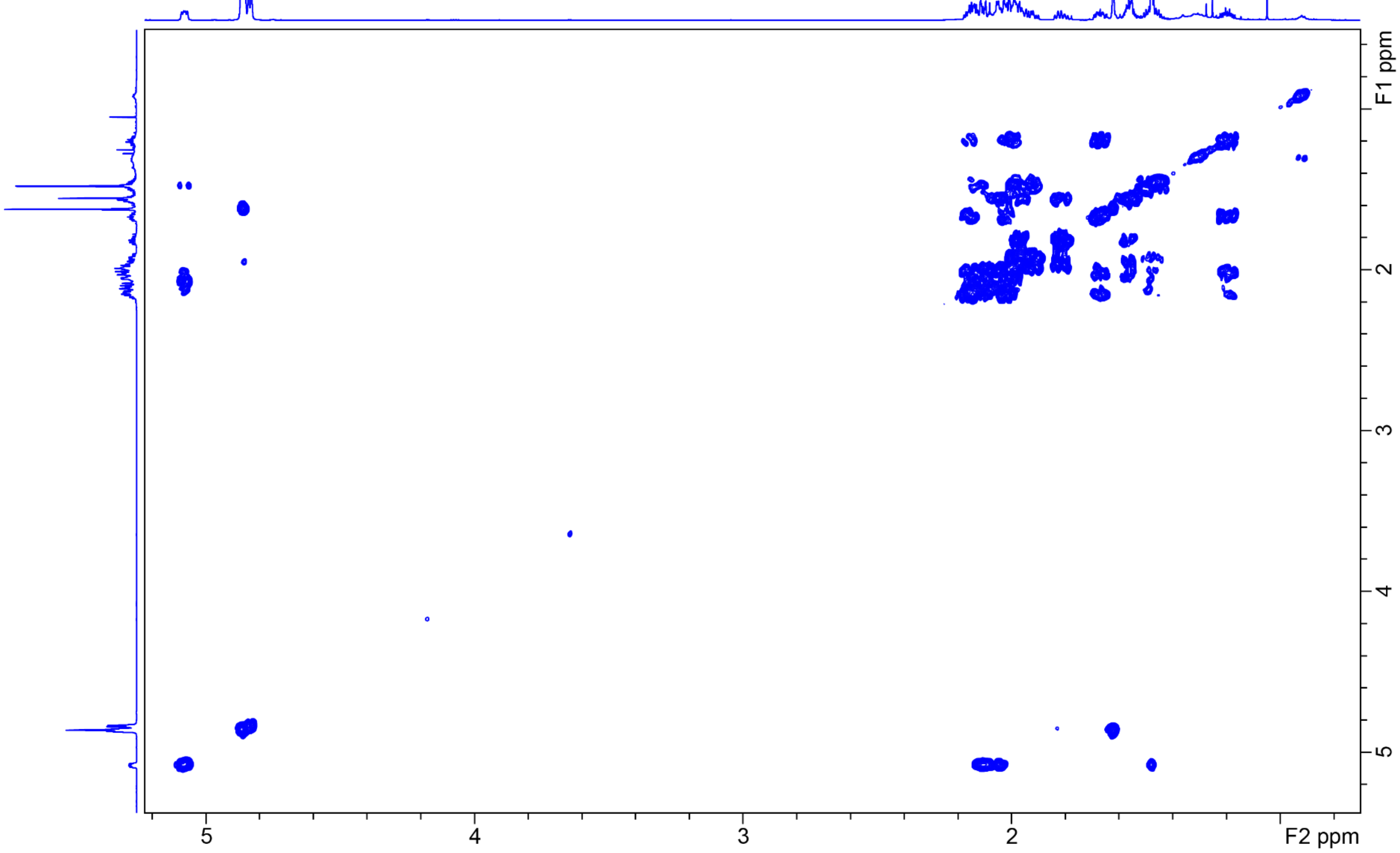

Figure S58. ^{1}H-^{1}H-COSY spectrum (C_6D_6) of **48**.

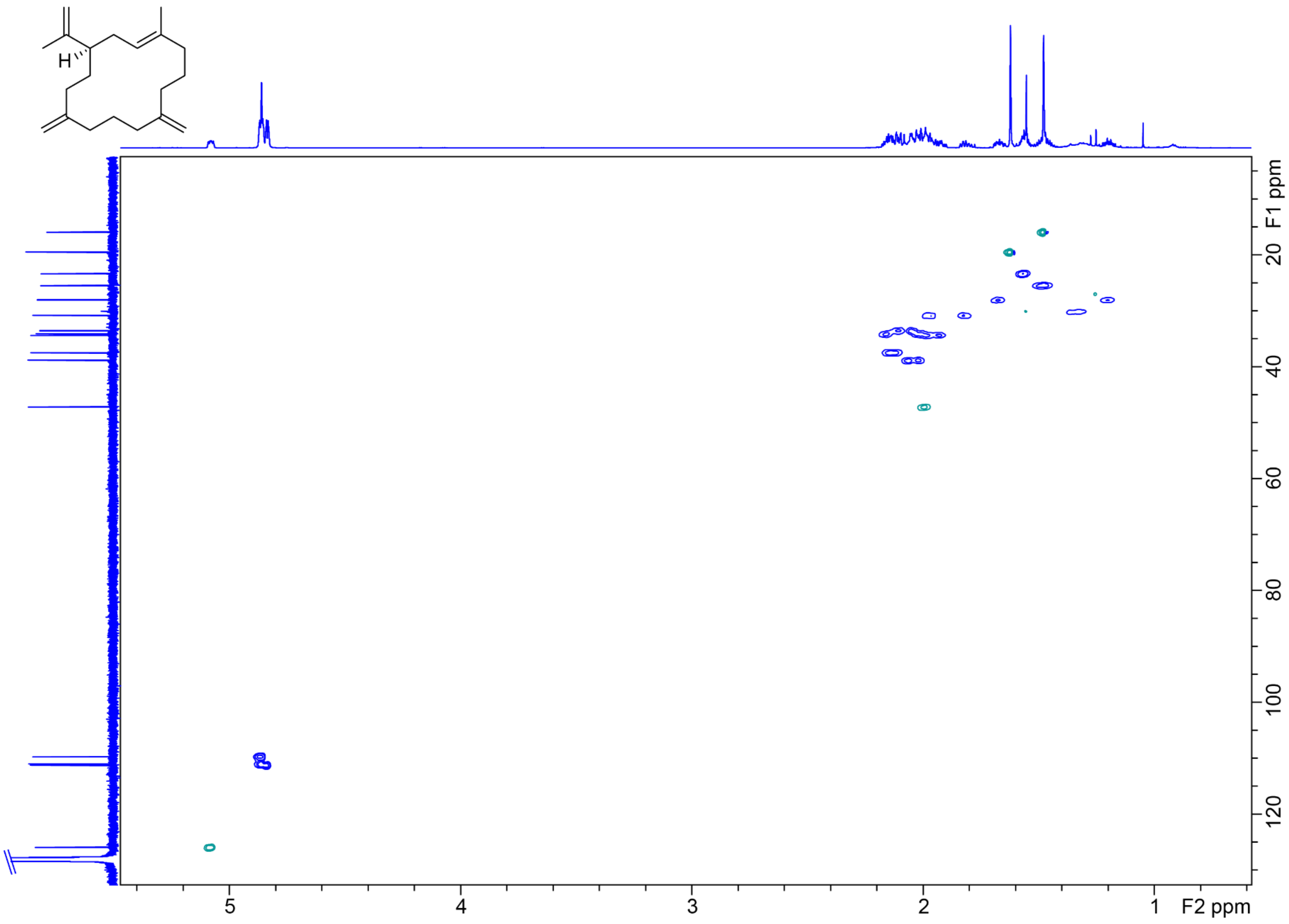

Figure S59. HSQC spectrum (C_6D_6) of **48**.

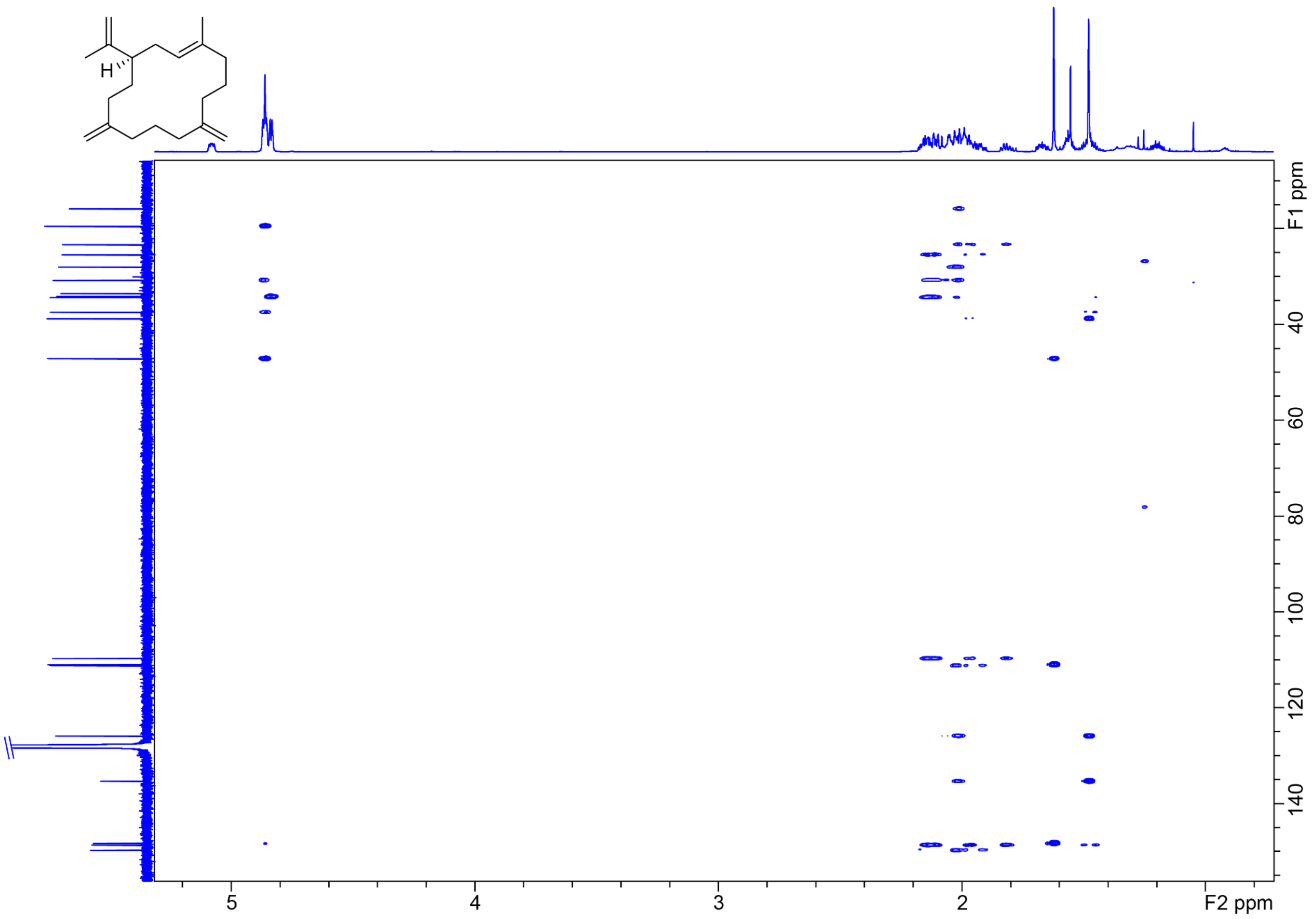

Figure S60. HMBC spectrum (C_6D_6) of **48**.

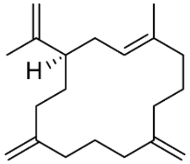

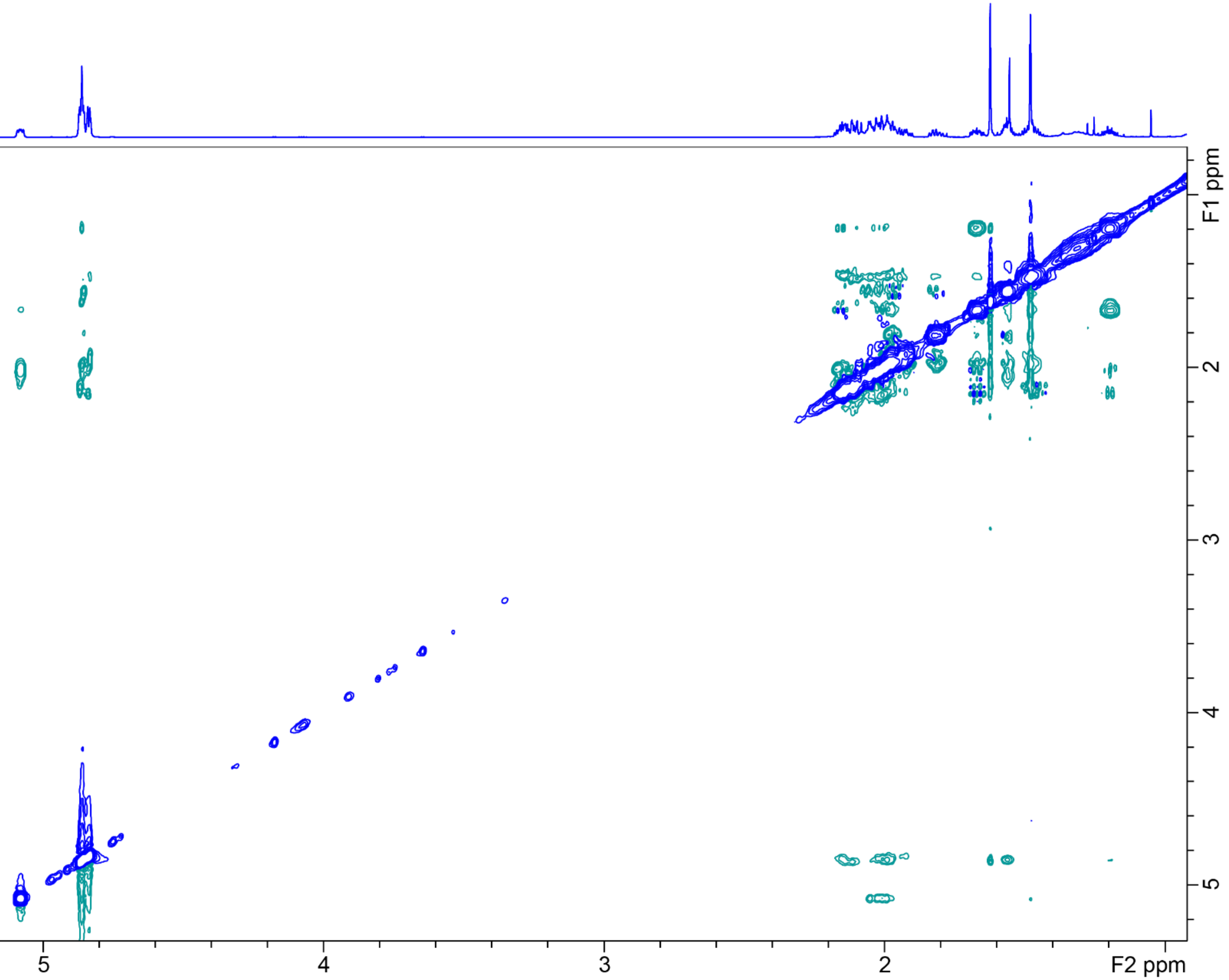

Figure S61. NOESY spectrum (C_6D_6) of **48**.

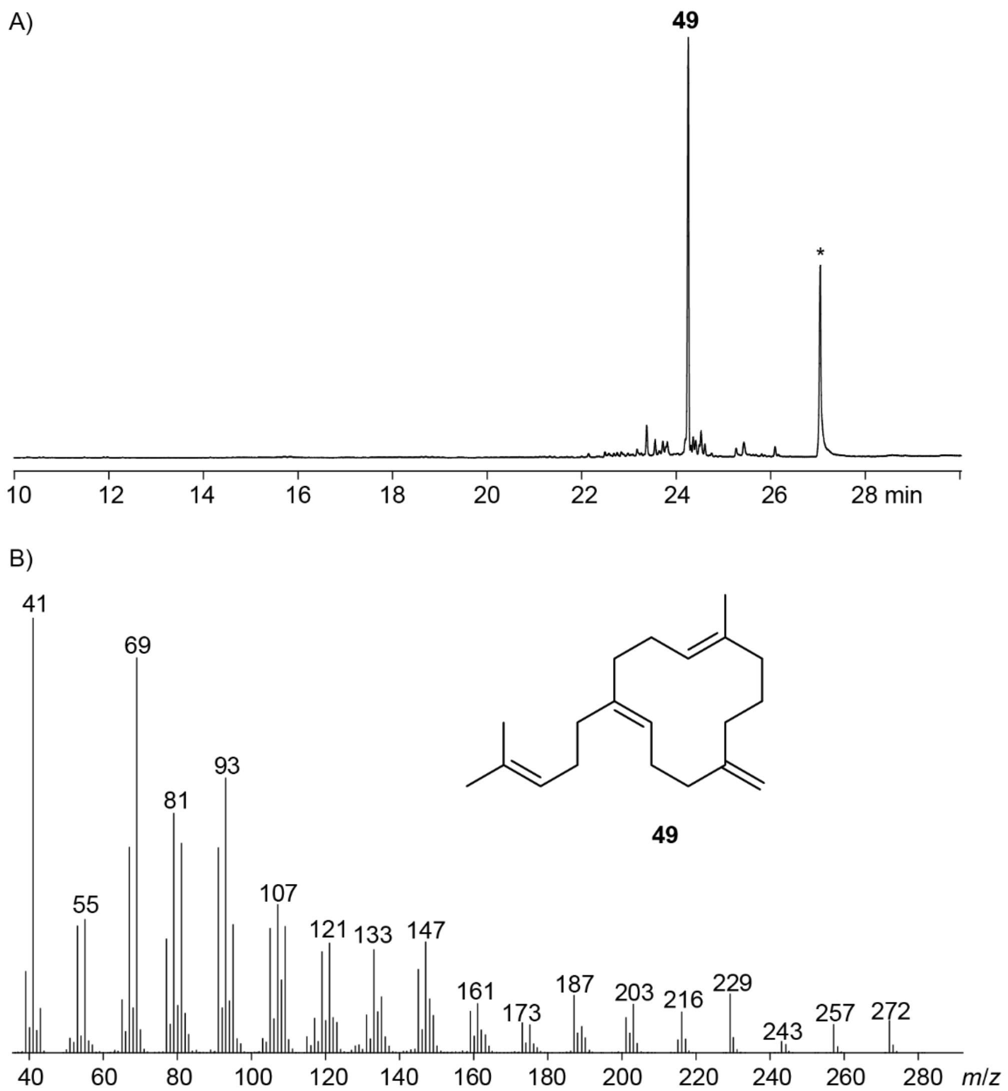

Figure S62. Enzymatic conversion of *iso*-GGPP IV with AbVS. A) Total ion chromatogram of an extract from an enzyme incubation, B) EI mass spectrum of **49**. Asterisks indicate spontaneous lysis and hydrolysis products of *iso*-GGPP IV and contaminants such as plasticisers.

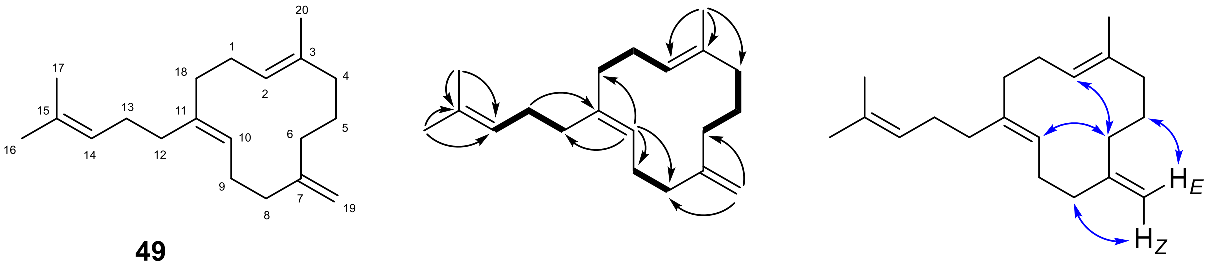

Figure S63. Structure elucidation of prenylisopseudogermacrene B (**49**). Bold: $^{1}H,^{1}H$-COSY, single headed arrows: key HMBC, and blue double headed arrows: NOESY correlations.

Table S11. NMR data of prenylisopseudogermacrene B (**49**) in C_6D_6 recorded at 298 K.

C[a]	type	^{13}C[b]	^{1}H[b]
1	CH_2	25.64	2.13 (m, 2H)
2	CH	126.36	5.16 (t, *J* = 7.9)
3	C_q	132.37	–
4	CH_2	36.91	2.12 (m, 2H)
5	CH_2	21.77	1.61 (m, 2H)
6	CH_2	29.73	1.93 (t, *J* = 5.7, 2H)
7	C_q	149.01	–
8	CH_2	38.94	2.11 (m, 2H)
9	CH_2	27.80	2.16 (m, 2H)
10	CH	128.04	5.20 (t, *J* = 7.9)
11	C_q	136.87	–
12	CH_2	36.23	2.09 (m, 2H)
13	CH_2	27.48	2.20 (m, 2H)
14	CH	125.20	5.26 (tqq, *J* = 6.9, 1.2, 1.2)
15	C_q	131.13	–
16	CH_3	25.88	1.68 (br s)
17	CH_3	17.79	1.57 (br s)
18	CH_2	29.74	2.09 (m, 2H)
19	CH_2	109.00	4.88 (br s, H_Z)
			4.75 (td, *J* = 1.9, 1.9, H_E)
20	CH_3	14.83	1.47 (d, *J* = 1.1)

[a] Carbon numbering as shown in Figure S63. [b] Chemical shifts δ in ppm, multiplicity: s = singlet, d = doublet, t = triplet, q = quartet, m = multiplet, br = broad, coupling constants *J* are given in Hertz.

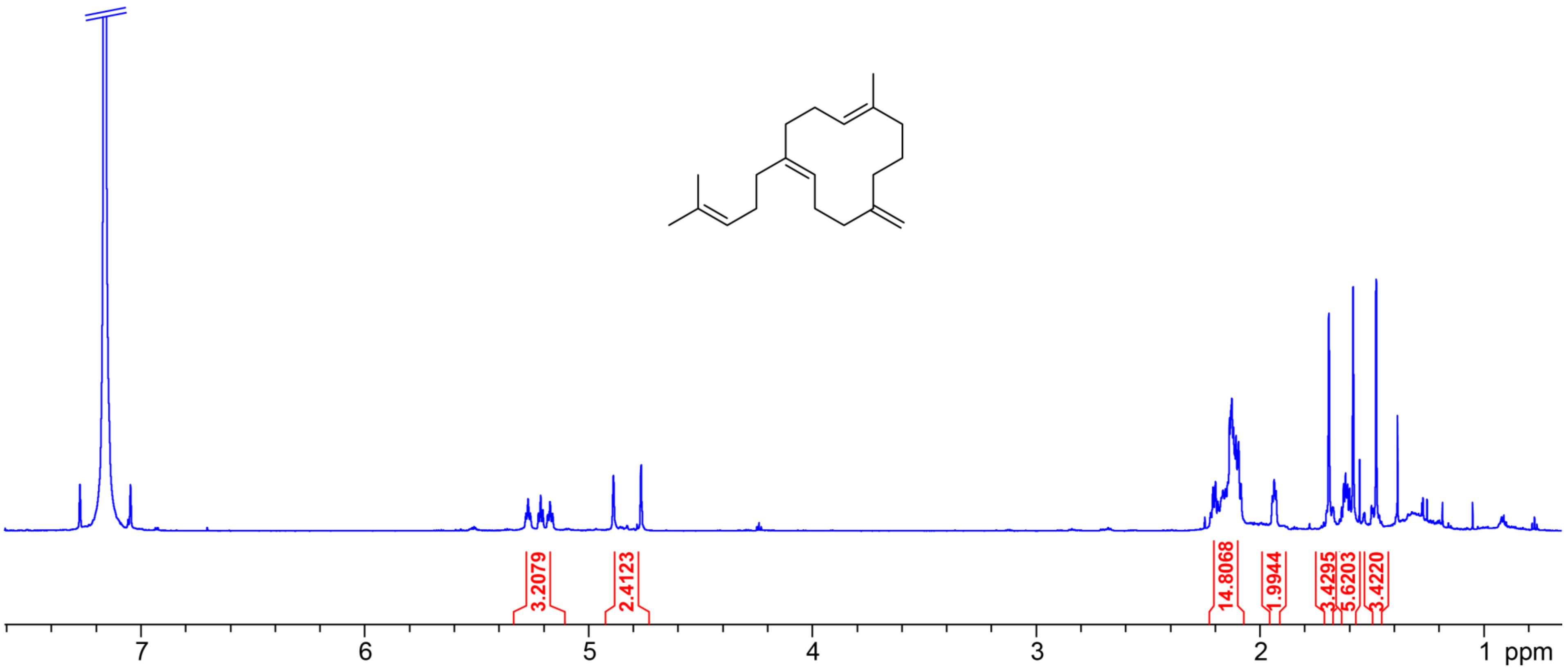

Figure S64. ^{1}H-NMR spectrum of **49** (700 MHz, C_6D_6).

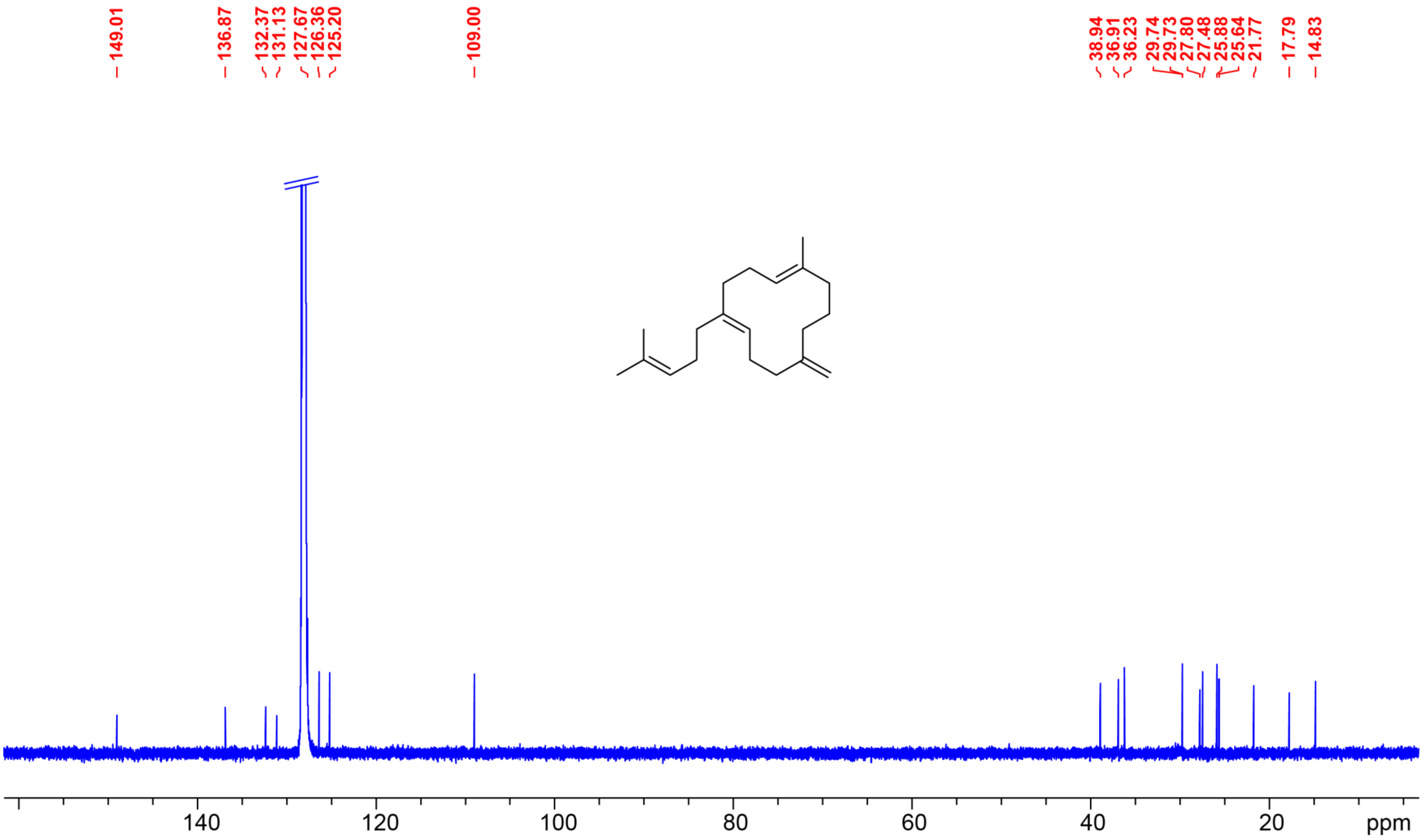

Figure S65. ^{13}C-NMR spectrum of **49** (176 MHz, C_6D_6).

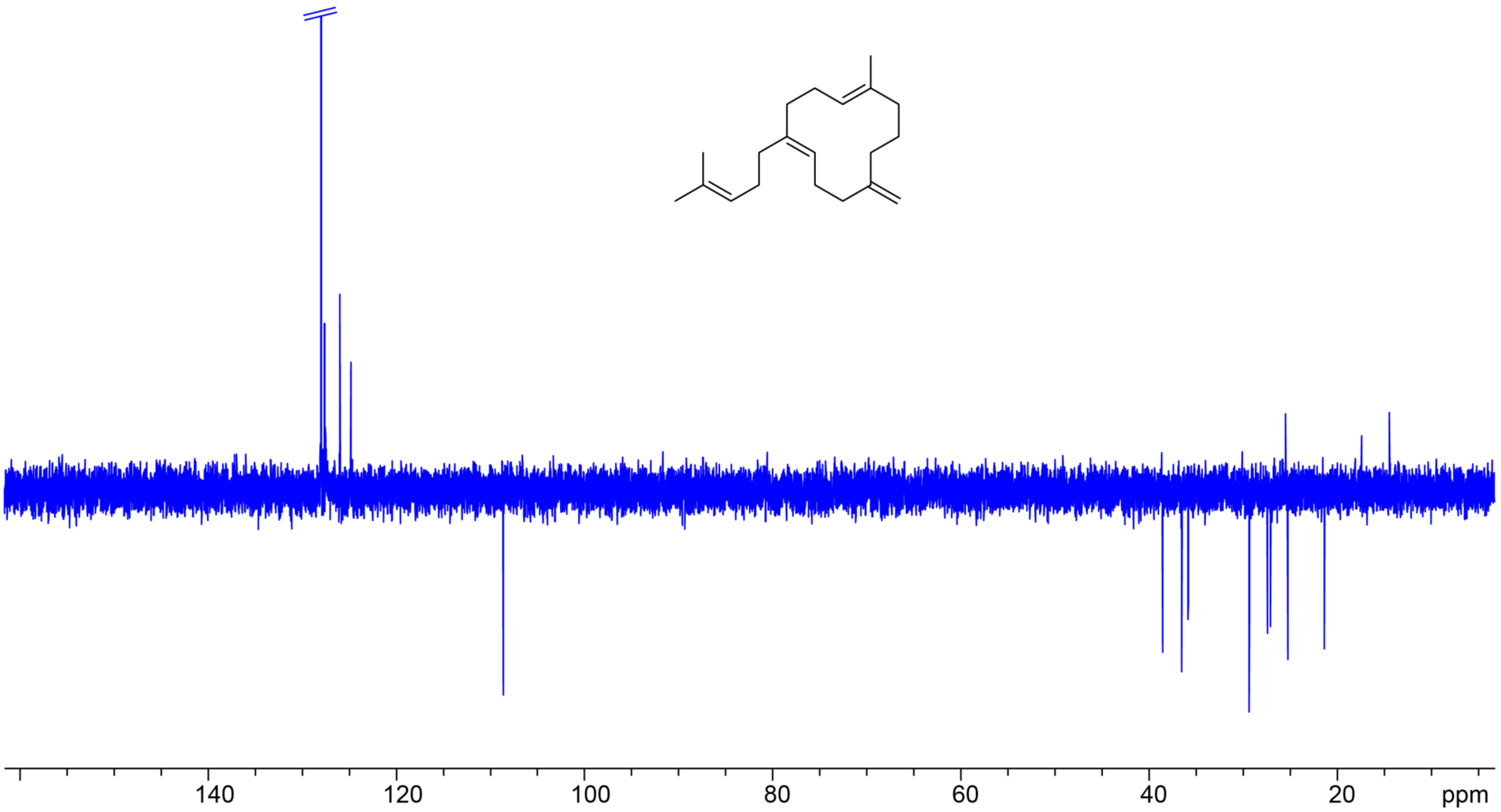

Figure S66. ^{13}C-DEPT135 spectrum of **49** (176 MHz, C_6D_6).

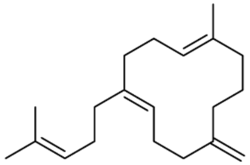

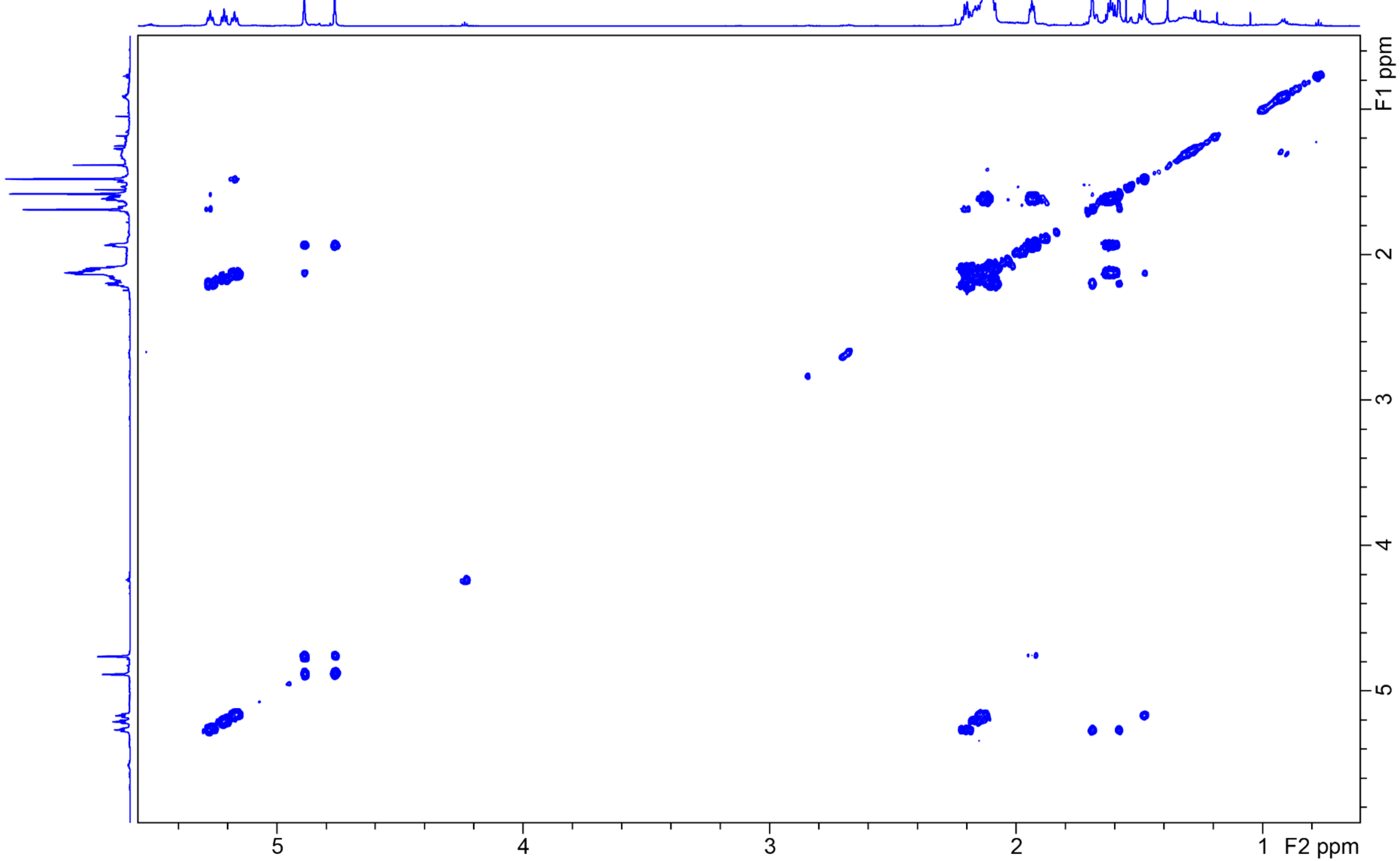

Figure S67. ^{1}H-^{1}H-COSY spectrum (C_6D_6) of **49**.

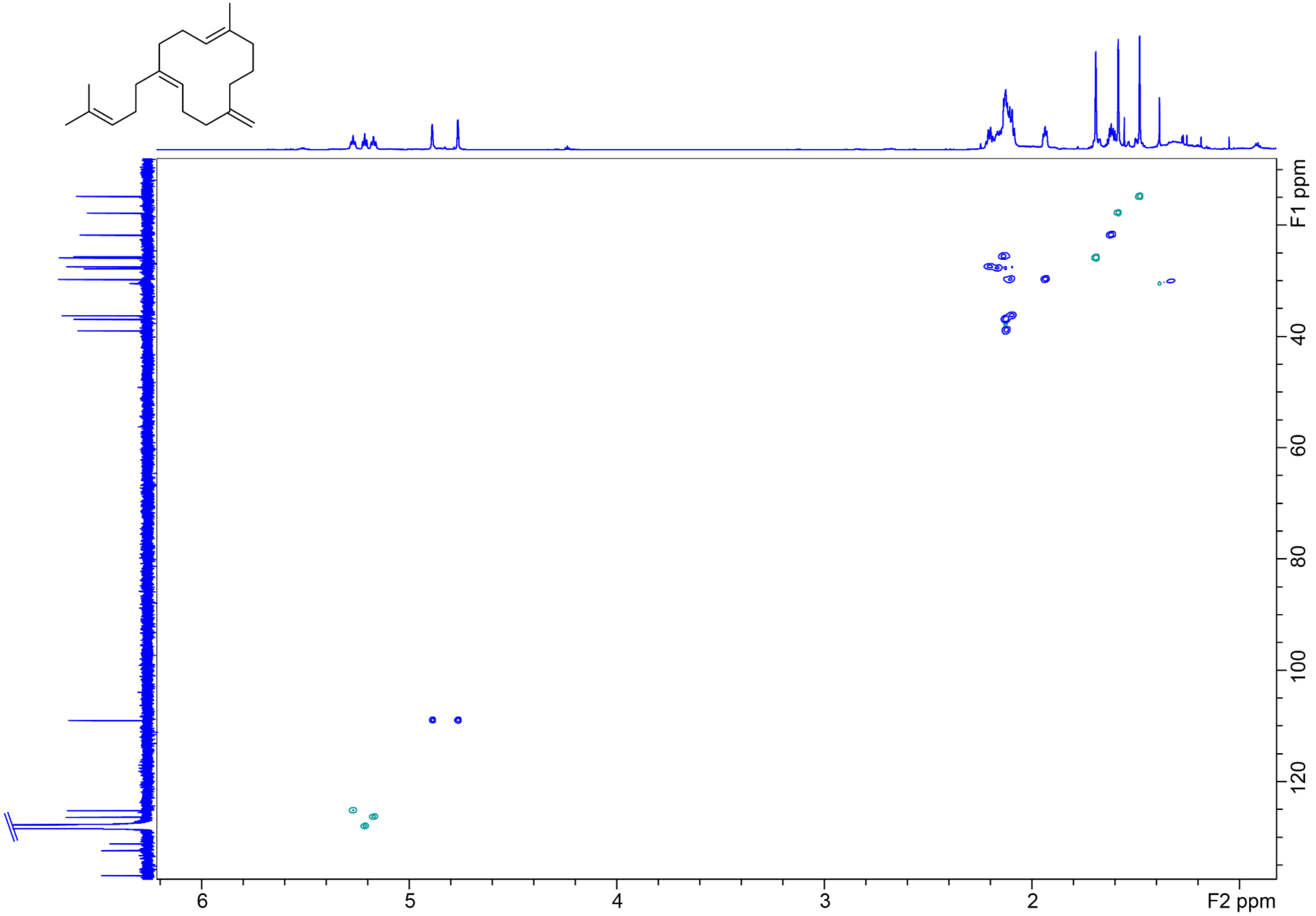

Figure S68. HSQC spectrum (C_6D_6) of **49**.

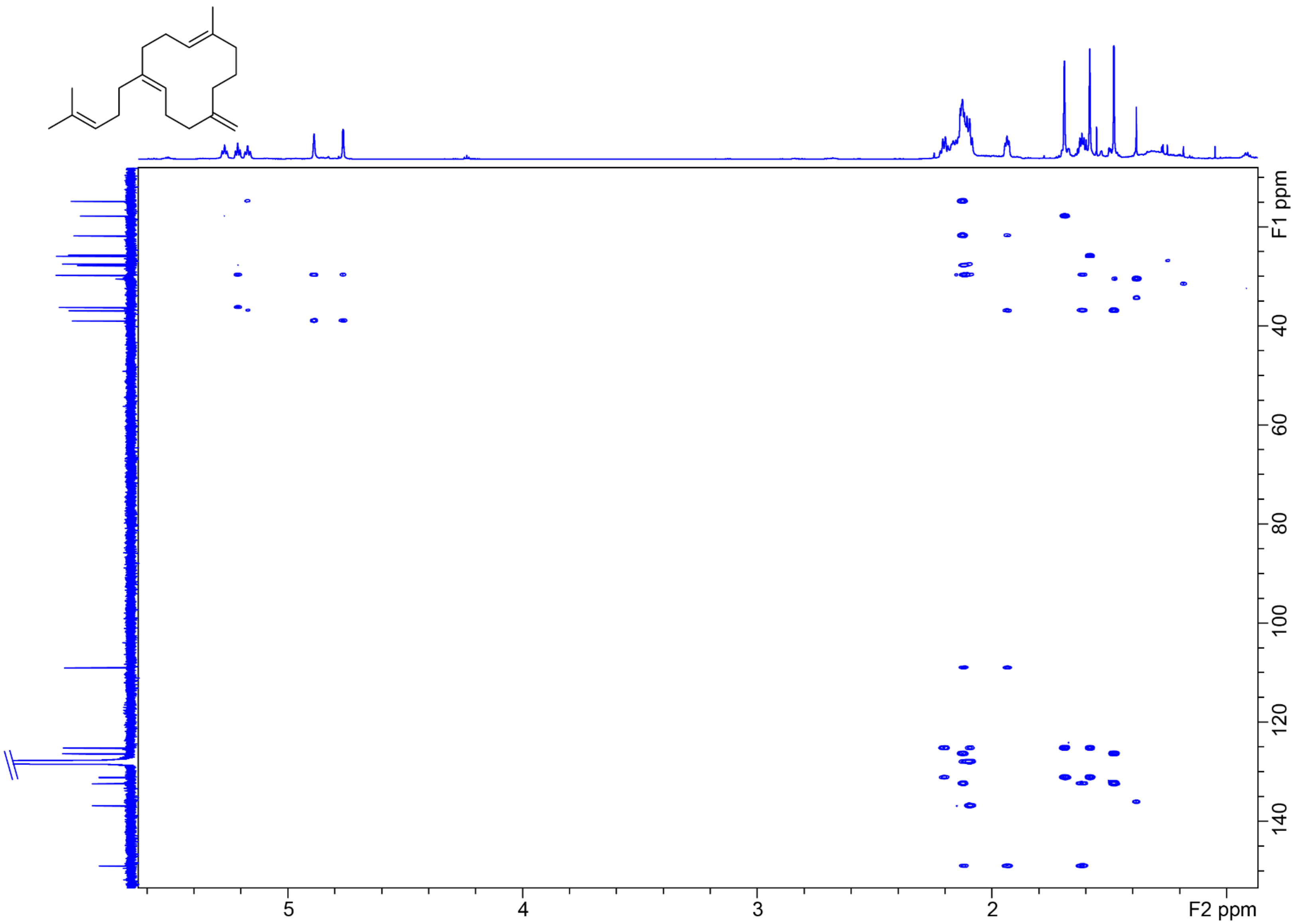

Figure S69. HMBC spectrum (C_6D_6) of **49**.

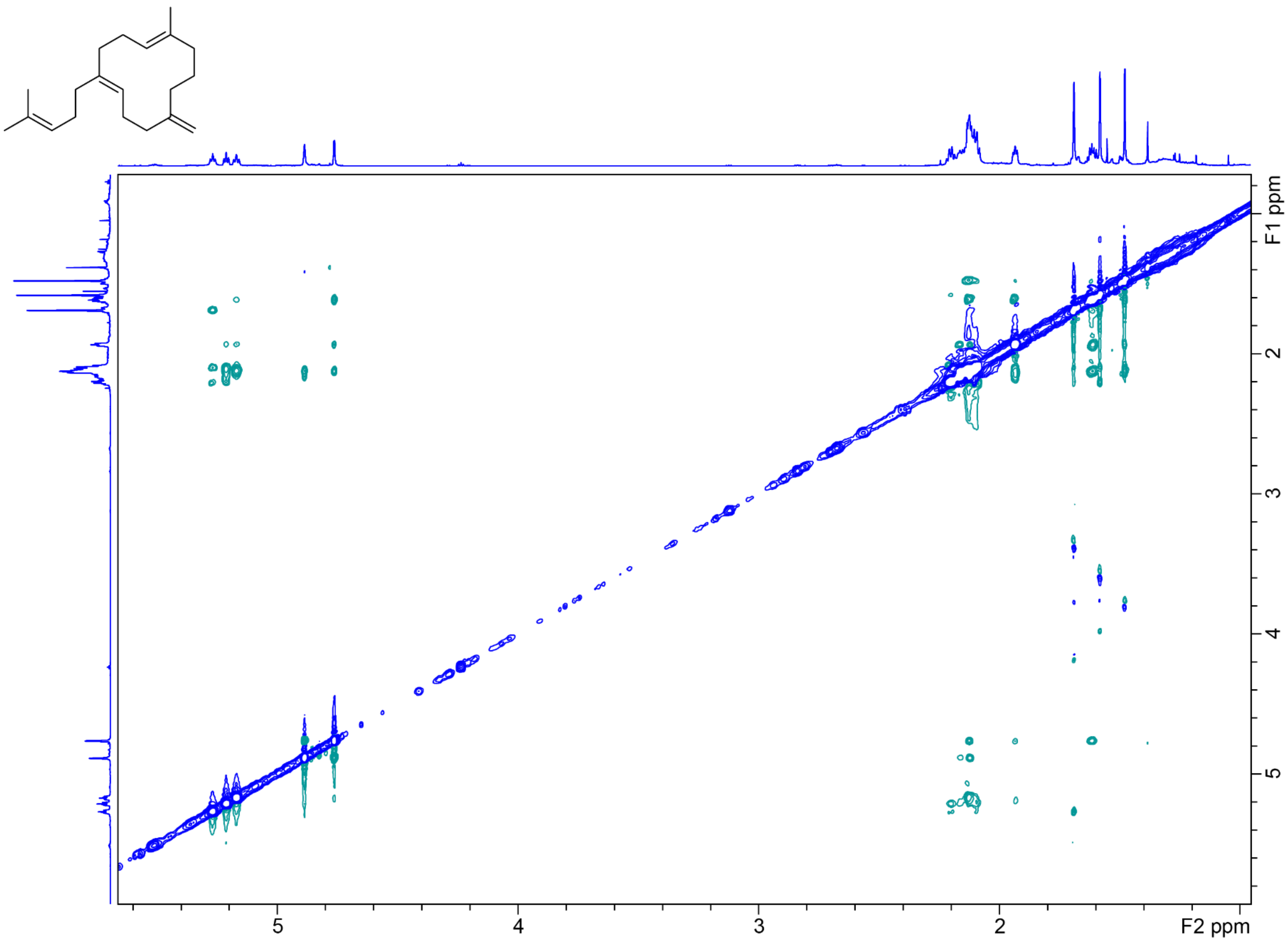

Figure S70. NOESY spectrum (C_6D_6) of **49**.

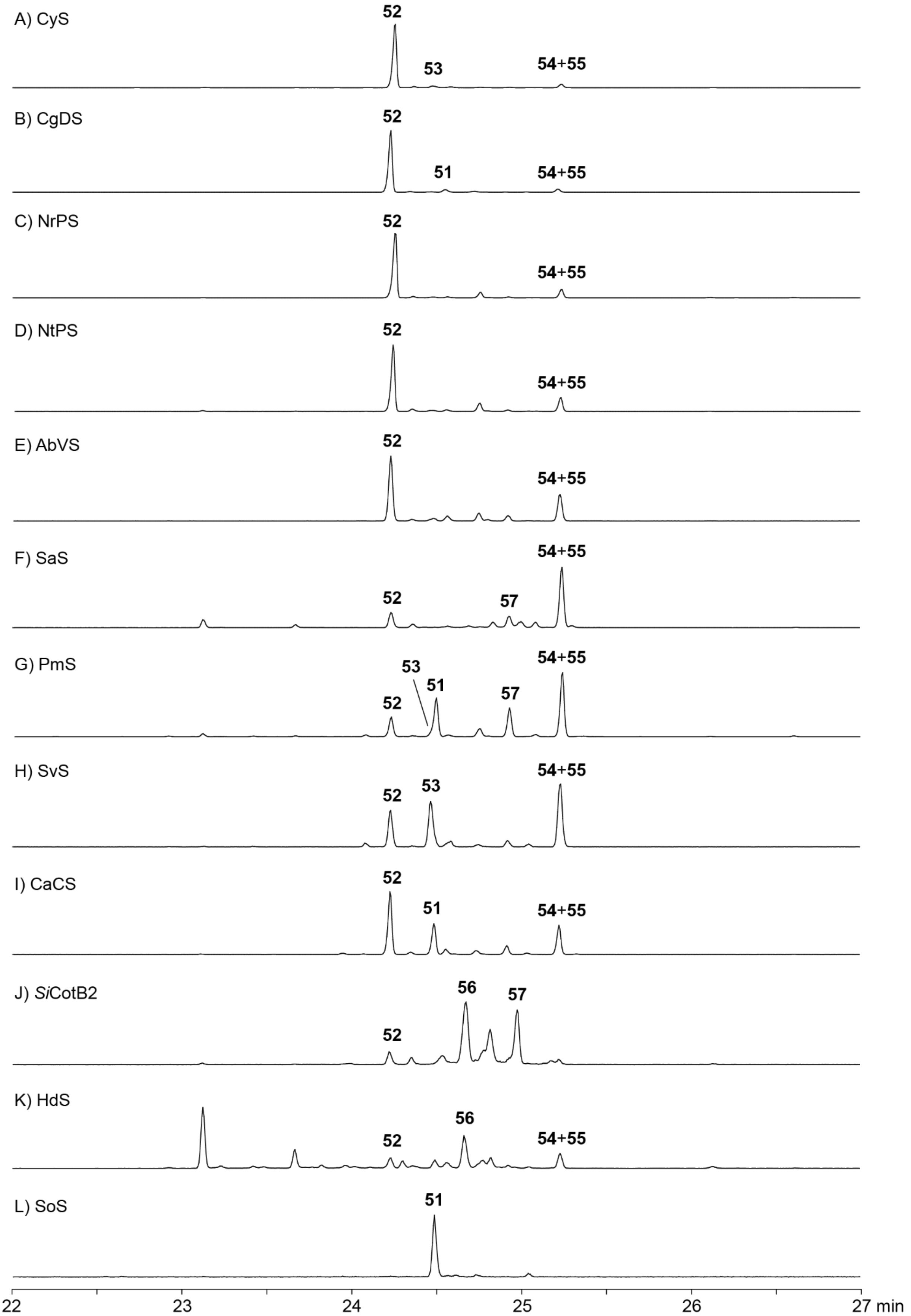

Figure S71. Enzymatic conversion of *iso*-GGPP VI with diverse diterpene synthases. Asterisks indicate spontaneous lysis and hydrolysis products of *iso*-GGPP VI and contaminants such as plasticisers.

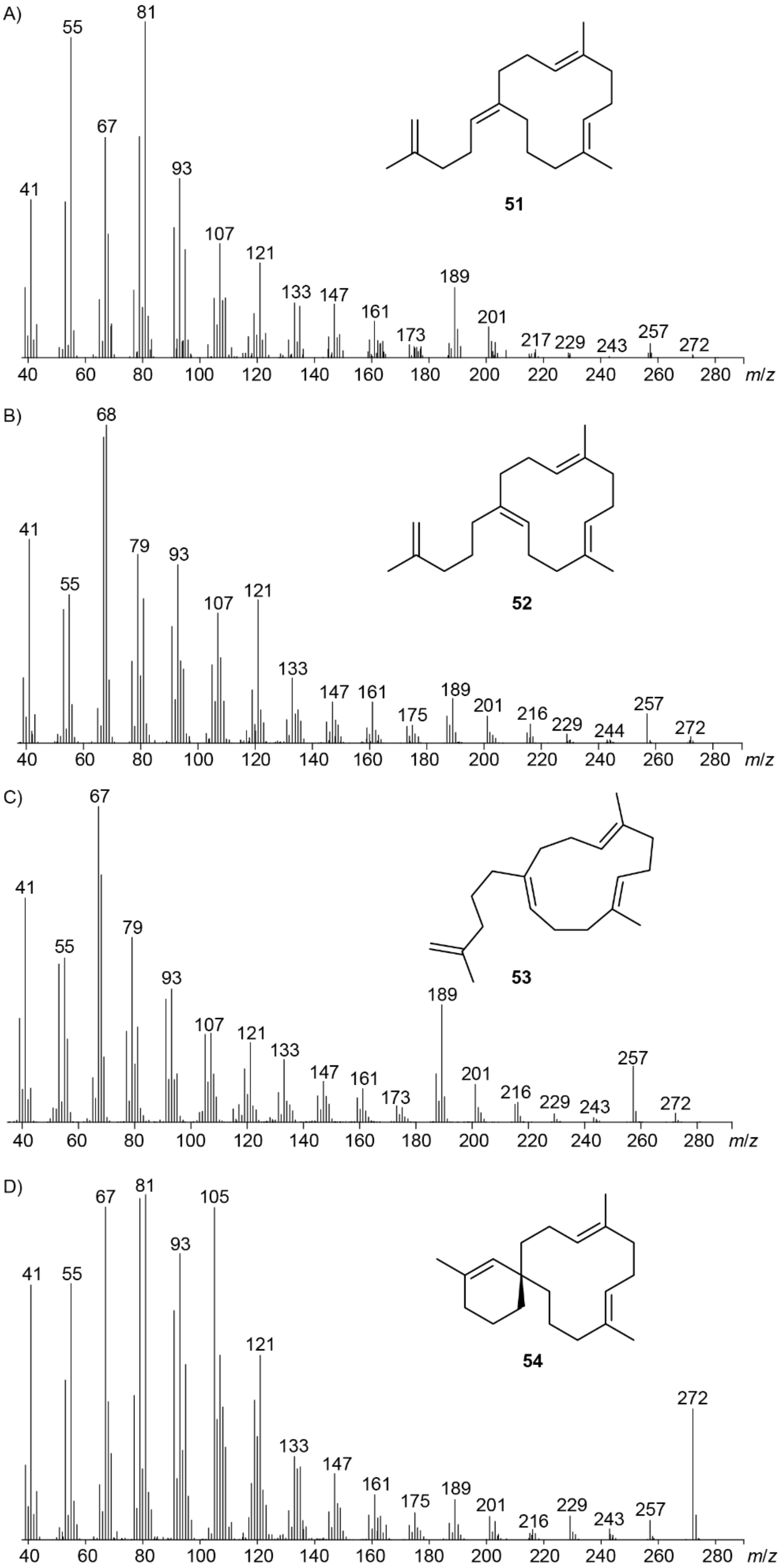

Figure S72. EI-MS spectra of A) isopentenylpseudogermacrene A (**51**), B) isopentenylpseudogermacrene B (**52**), C) isopentenylpseudogermacrene C (**53**), and D) α-spirocattleyaxenene (**54**).

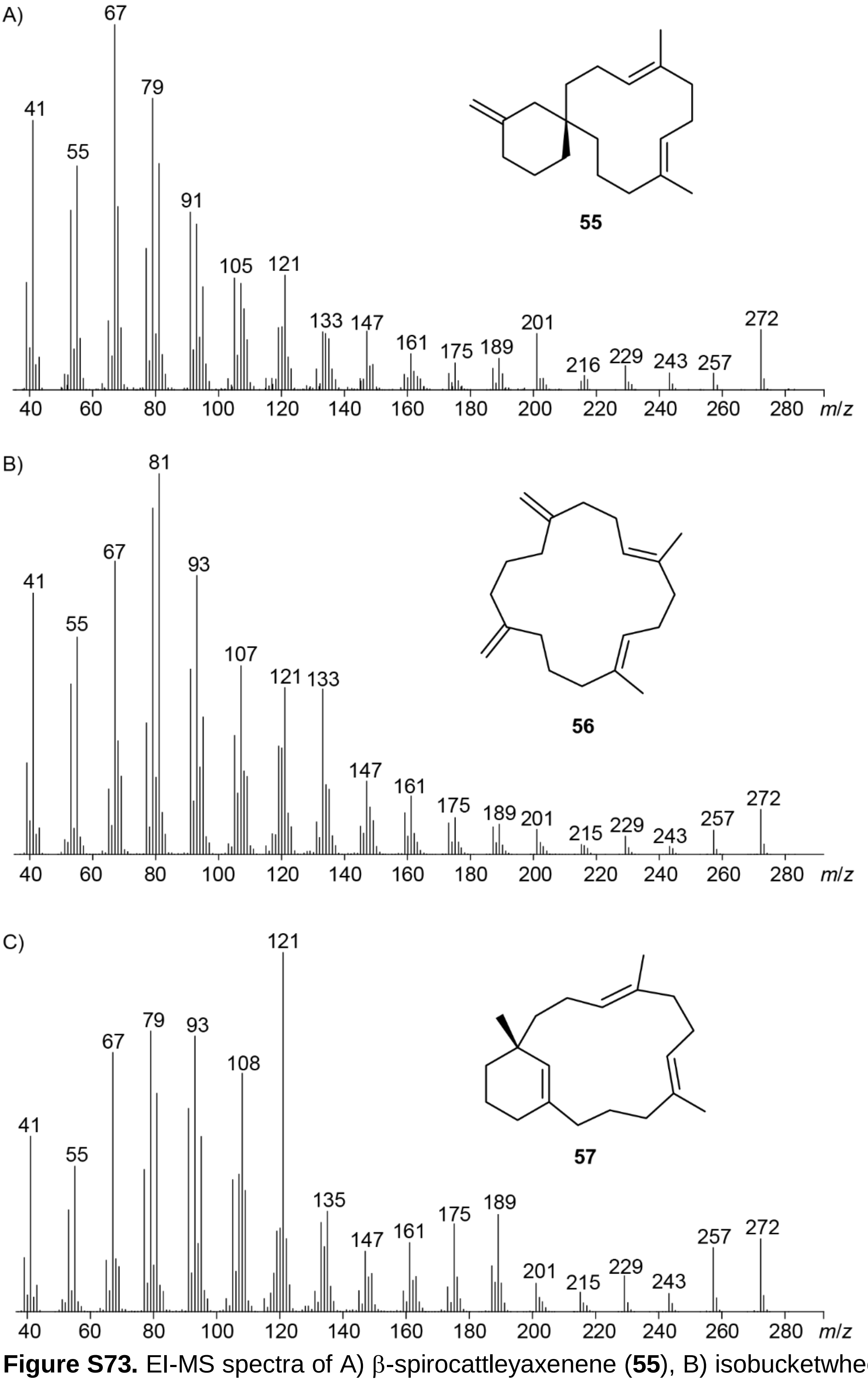

Figure S73. EI-MS spectra of A) β-spirocattleyaxenene (**55**), B) isobucketwheelene (**56**), and C) iakyroxenene (**57**).

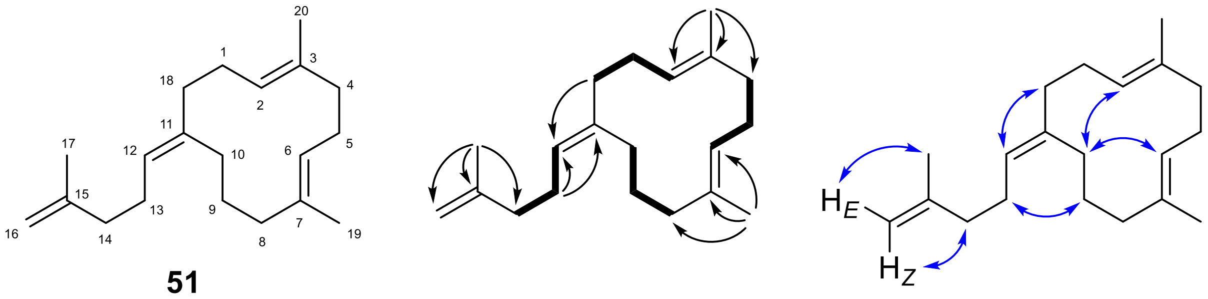

Figure S74. Structure elucidation of isopentenylpseudogermacrene A (**51**). Bold: ^{1}H,^{1}H-COSY, single headed arrows: key HMBC, and blue double headed arrows: NOESY correlations.

Table S12. NMR data of isopentenylpseudogermacrene A (**51**) in C_6D_6 recorded at 298 K.

C[a]	type	^{13}C[b]	^{1}H[b]
1	CH_2	30.11	2.16 (m, 2H)
2	CH	128.82	5.31 (dt, *J* = 7.5, 1.0)
3	C_q	133.19	–
4	CH_2	39.81	2.03 (m, 2H)
5	CH_2	26.12	2.15 (m, 2H)
6	CH	126.60	4.92 (t, *J* = 7.4)
7	C_q	134.16	–
8	CH_2	39.52	1.92 (m, 2H)
9	CH_2	25.94	1.35 (m, 2H)
10	CH_2	27.66	2.23 (m, 2H)
11	C_q	142.75	–
12	CH	125.48	5.25 (t, *J* = 7.1)
13	CH_2	26.36	2.26 (m, 2H)
14	CH_2	38.78	2.08 (m, 2H)
15	C_q	145.69	–
16	CH_2	110.49	4.86 (m, H_Z)<br>4.84 (m, H_E)
17	CH_3	22.64	1.69 (br s)
18	CH_2	37.10	2.07 (m, 2H)
19	CH_3	17.07	1.53 (br s)
20	CH_3	15.02	1.47 (br s)

[a] Carbon numbering as shown in Figure S74. [b] Chemical shifts δ in ppm, multiplicity: s = singlet, d = doublet, t = triplet, m = multiplet, br = broad, coupling constants *J* are given in Hertz.

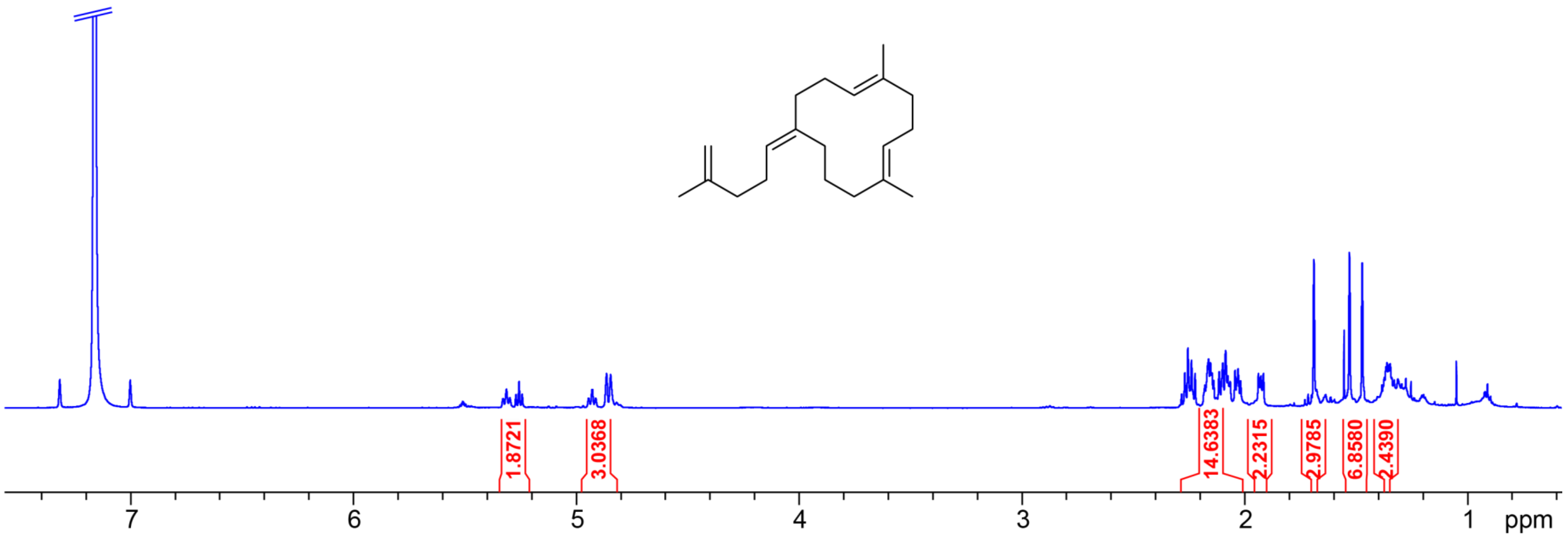

Figure S75. ^{1}H-NMR spectrum of **51** (700 MHz, C_6D_6).

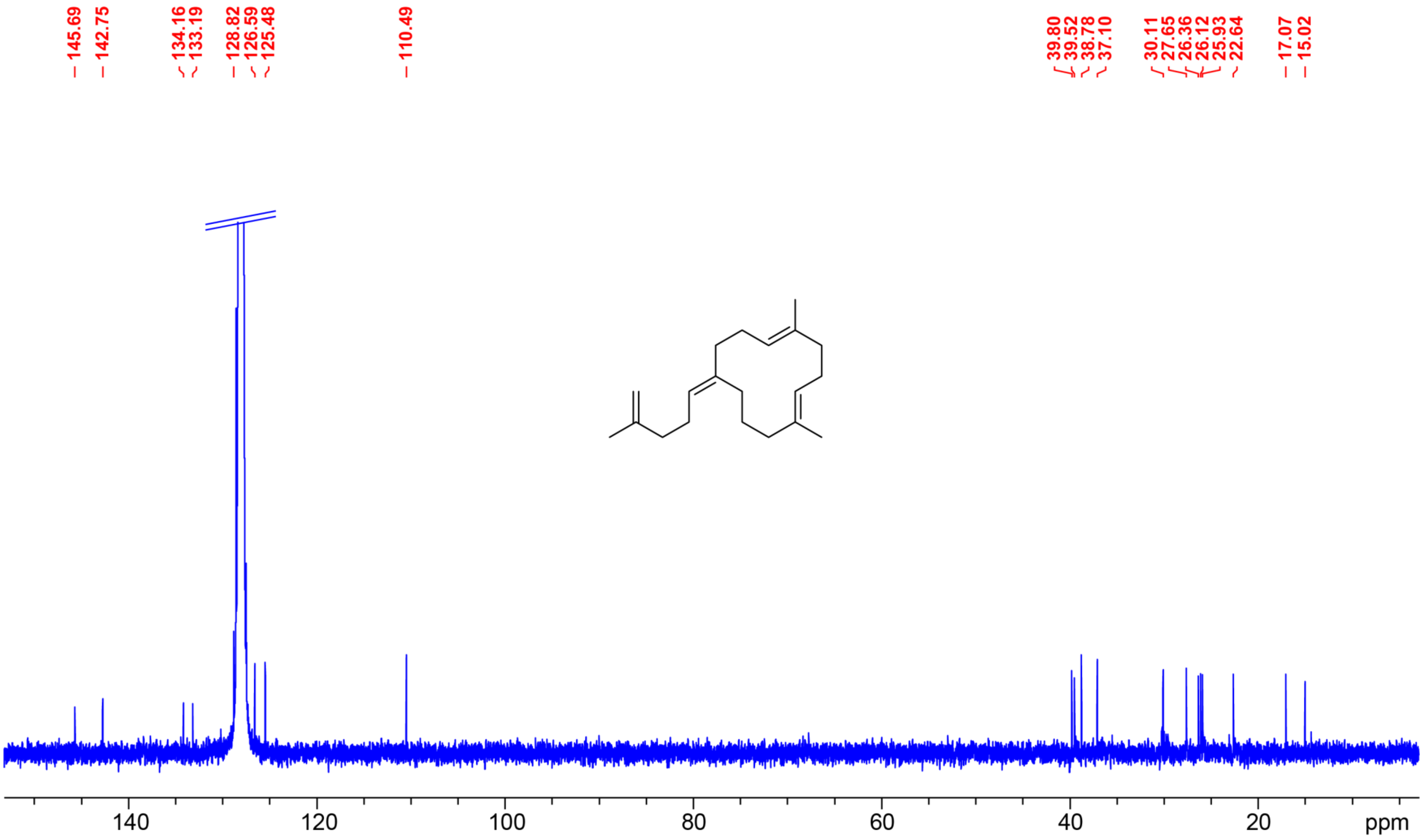

Figure S76. ^{13}C-NMR spectrum of **51** (176 MHz, C_6D_6).

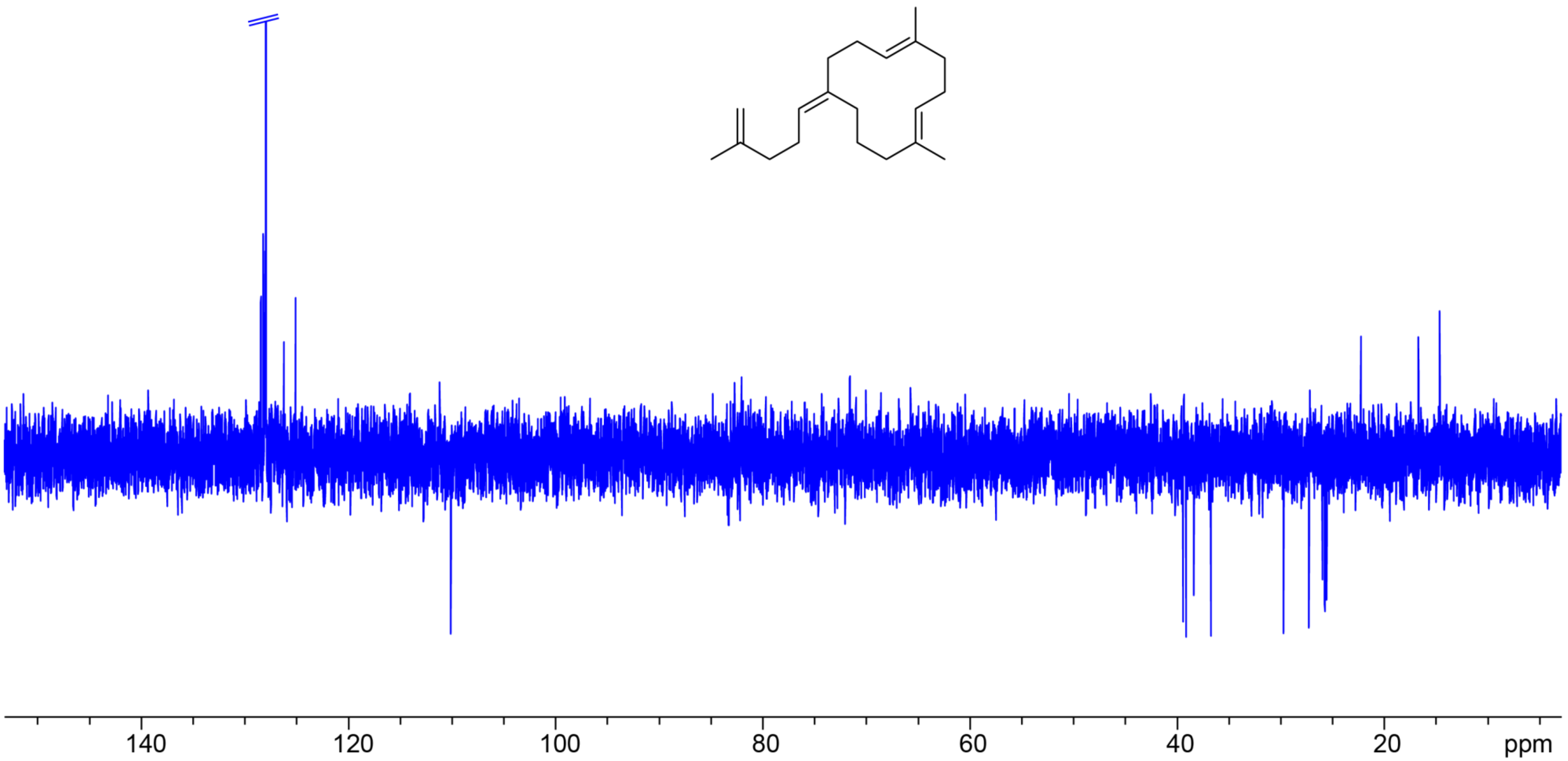

Figure S77. ^{13}C-DEPT135 spectrum of **51** (176 MHz, C_6D_6).

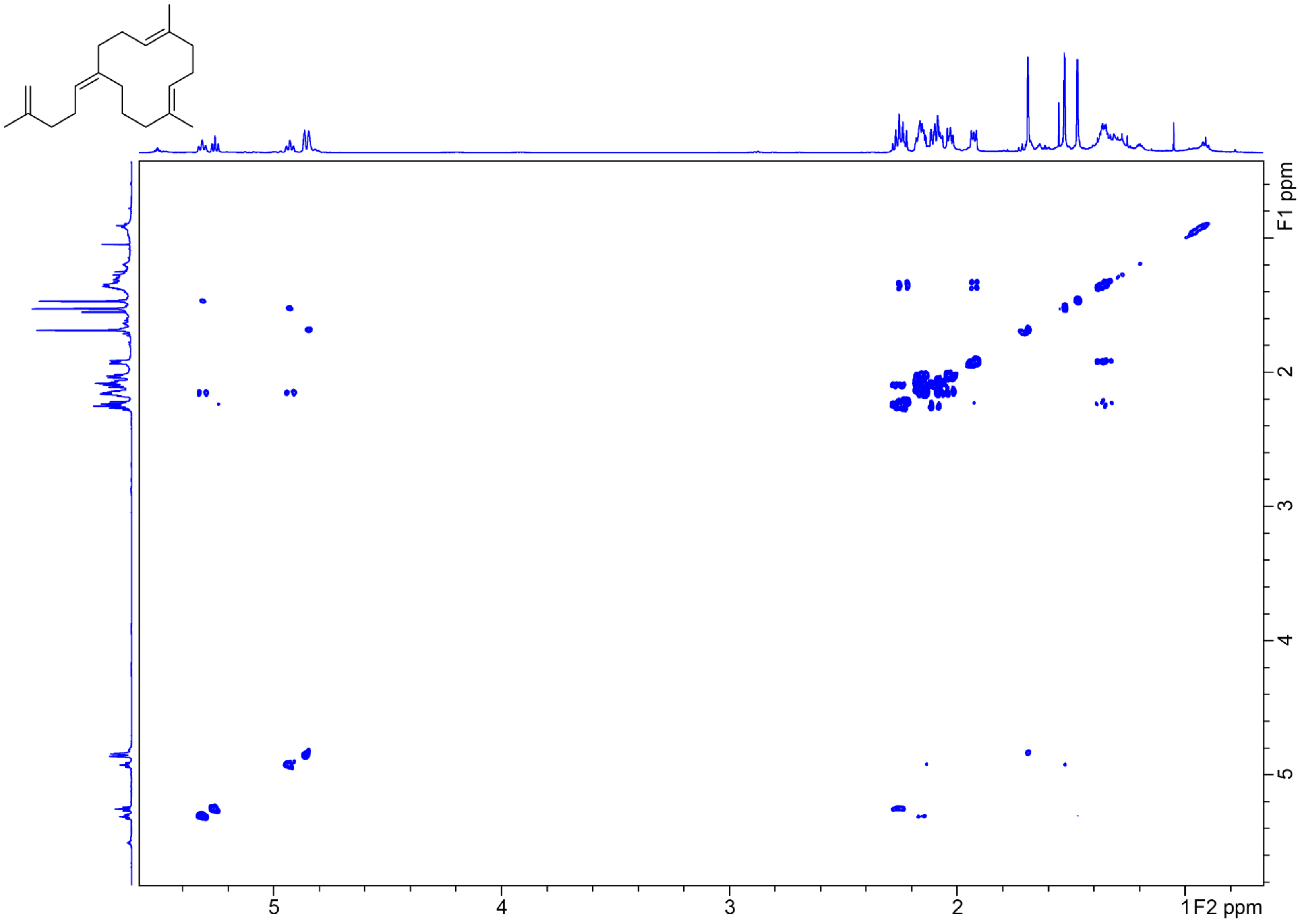

Figure S78. ^1H-^1H-COSY spectrum (C_6D_6) of **51**.

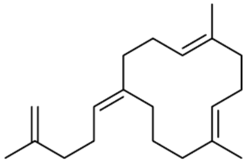

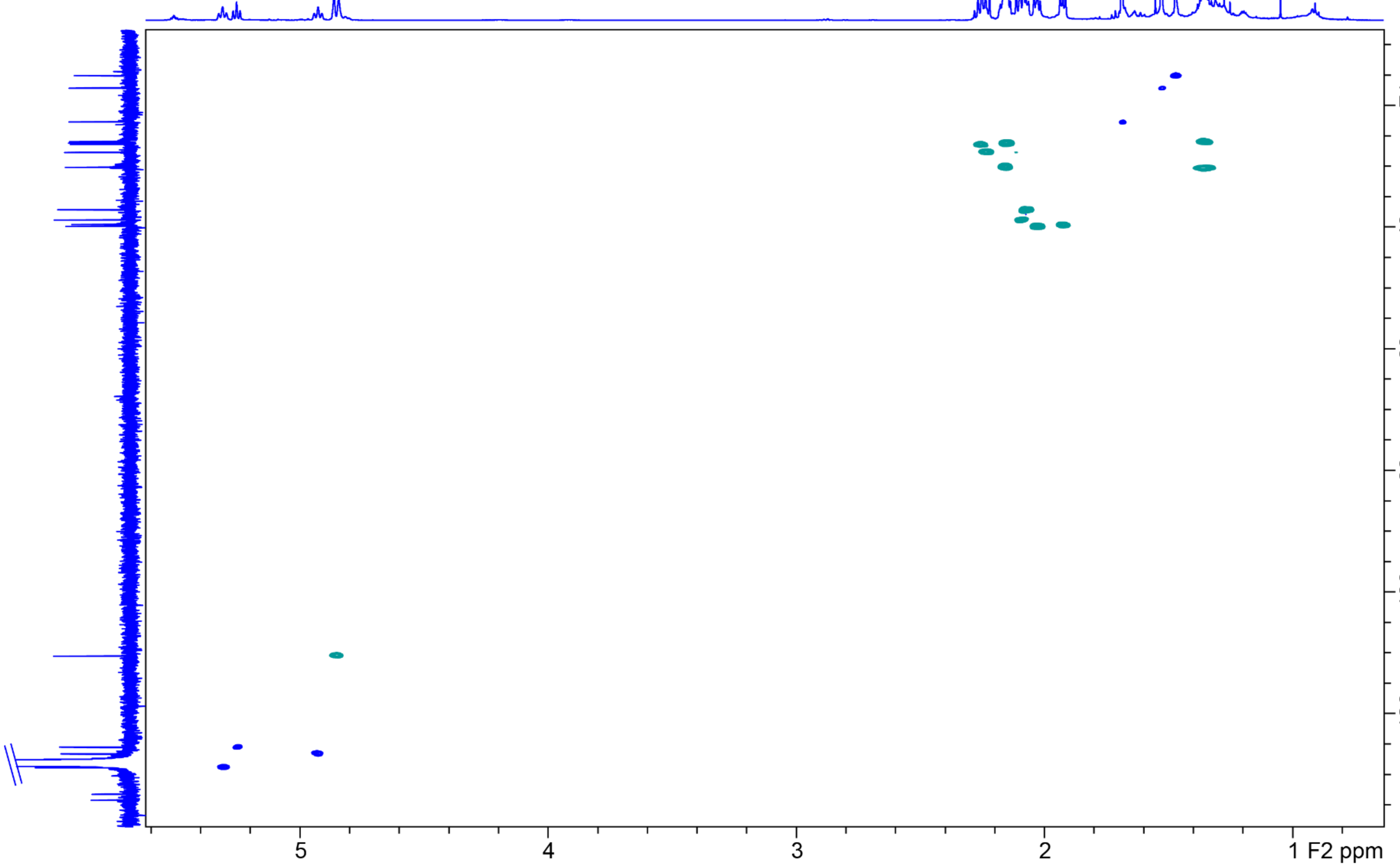

Figure S79. HSQC spectrum (C_6D_6) of **51**.

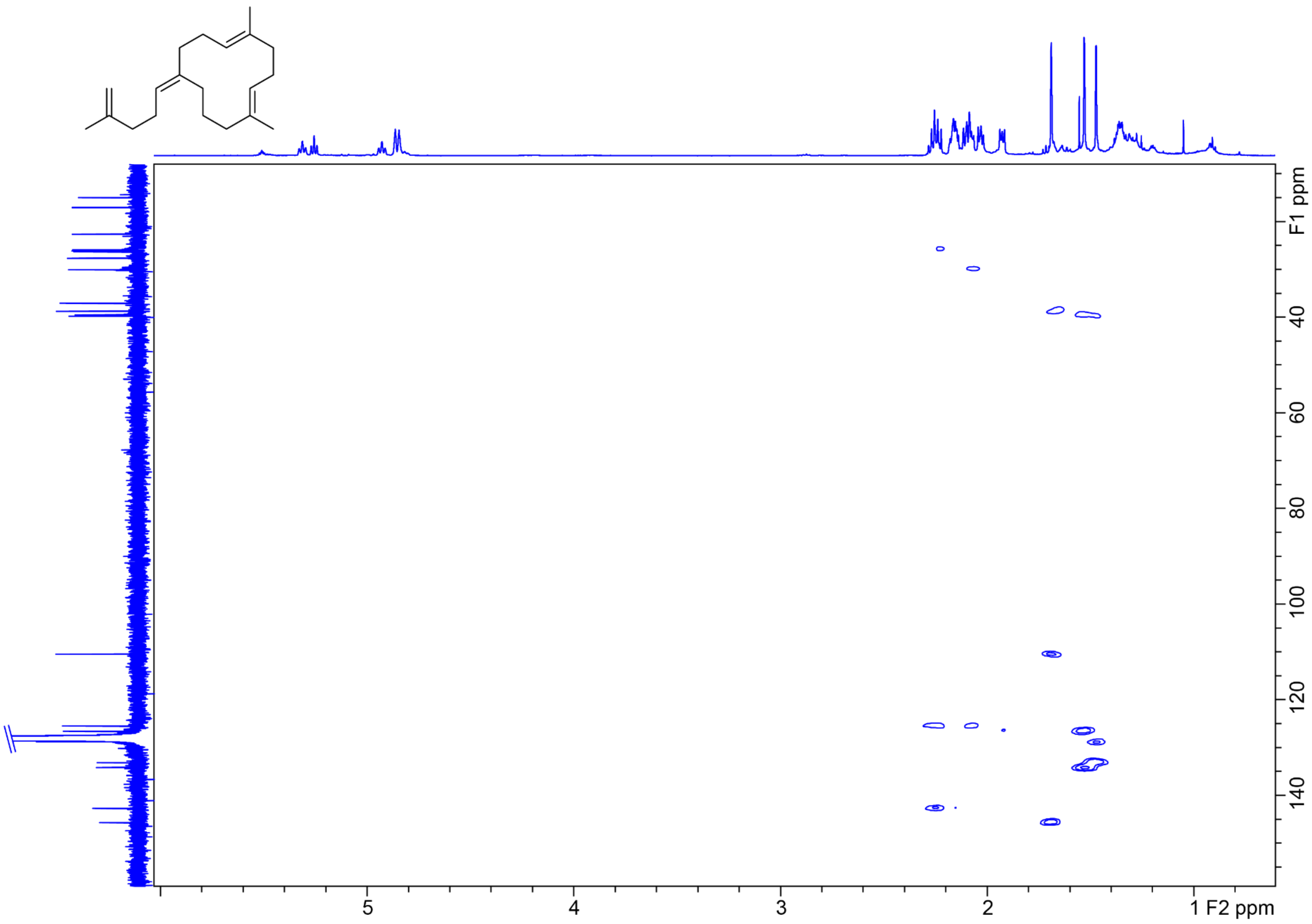

Figure S80. HMBC spectrum (C_6D_6) of **51**.

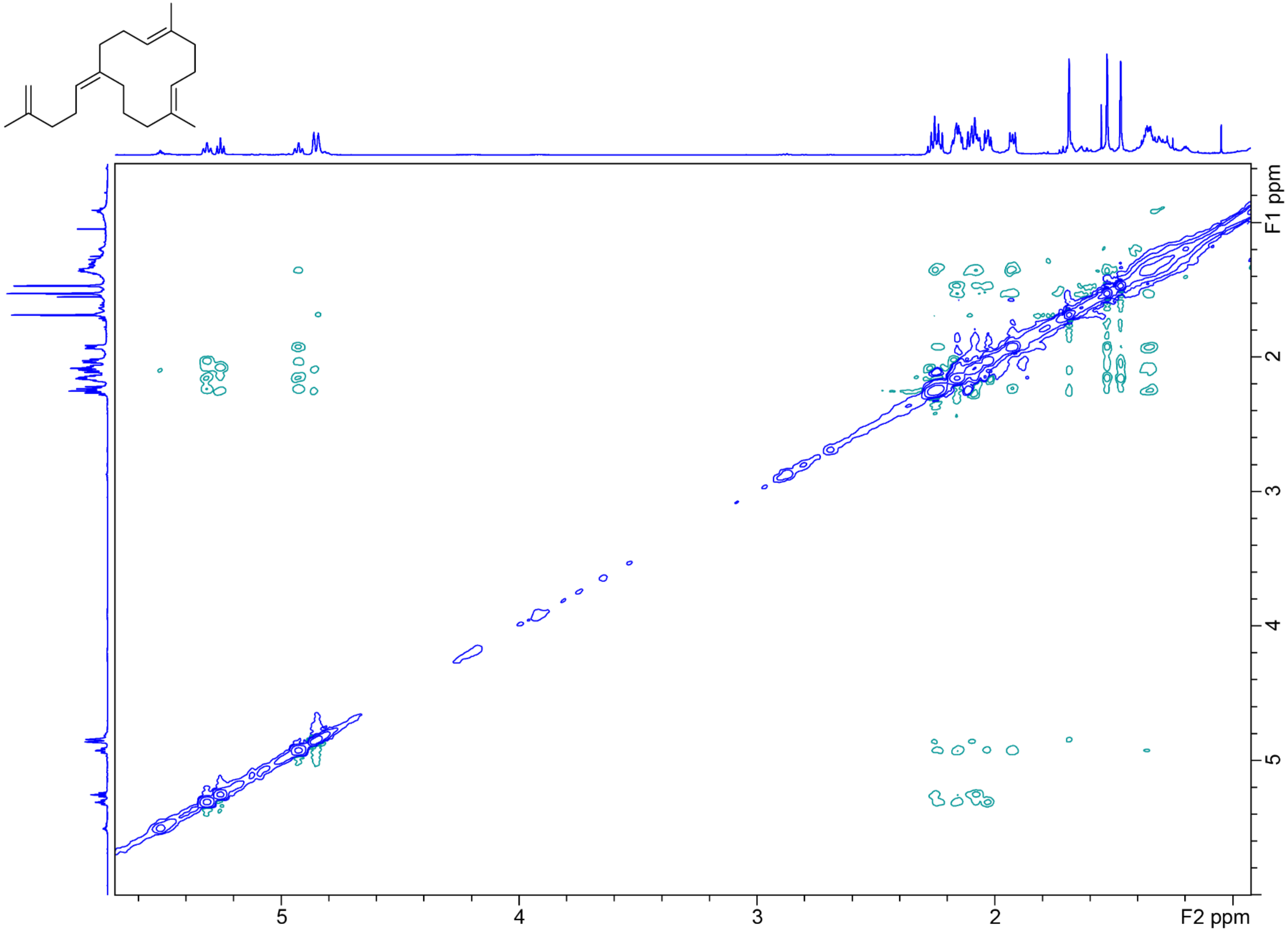

Figure S81. NOESY spectrum (C_6D_6) of **51**.

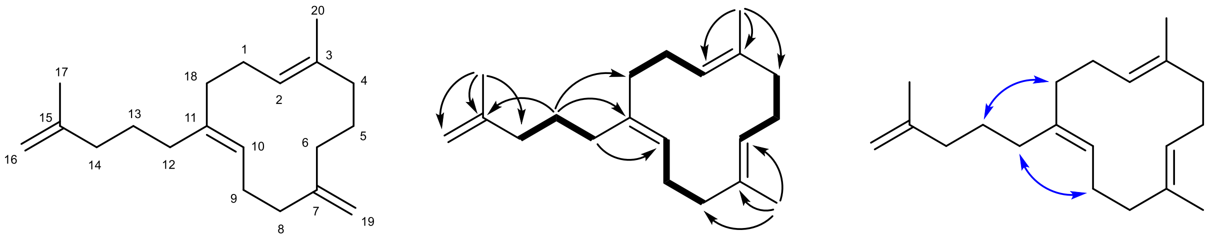

52

Figure S82. Structure elucidation of isopentenylpseudogermacrene B (**52**). Bold: ^{1}H,^{1}H-COSY, single headed arrows: key HMBC, and blue double headed arrows: NOESY correlations.

Table S13. NMR data of isopentenylpseudogermacrene B (**52**) in C_6D_6 recorded at 298 K.

C[a]	type	^{13}C[b]	^{1}H[b]
1	CH_2	26.01	2.10 (m, 2H)
2	CH	127.07	4.84 (m)
3	C_q	133.31	–
4	CH_2	40.21	2.05 (m, 2H)
5	CH_2	25.55	2.10 (m, 2H)
6	CH	126.95	4.84 (m)
7	C_q	133.11	–
8	CH_2	40.32	2.05 (m, 2H)
9	CH_2	25.26	2.10 (m, 2H)
10	CH	127.83	4.83 (m)
11	C_q	137.22	–
12	CH_2	28.65	1.92 (t, J = 7.6)
13	CH_2	27.06	1.53 (m)
14	CH_2	38.03	1.99 (t, J = 7.6)
15	C_q	145.87	–
16	CH_2	110.27	4.83 (m, 2H)
17	CH_3	22.61	1.67 (br s)
18	CH_2	37.10	2.07 (m, 2H)
19	CH_3	15.27	1.44 (br s)
20	CH_3	15.24	1.43 (br s)

[a] Carbon numbering as shown in Figure S82. [b] Chemical shifts δ in ppm, multiplicity: s = singlet, t = triplet, m = multiplet, br = broad, coupling constants J are given in Hertz.

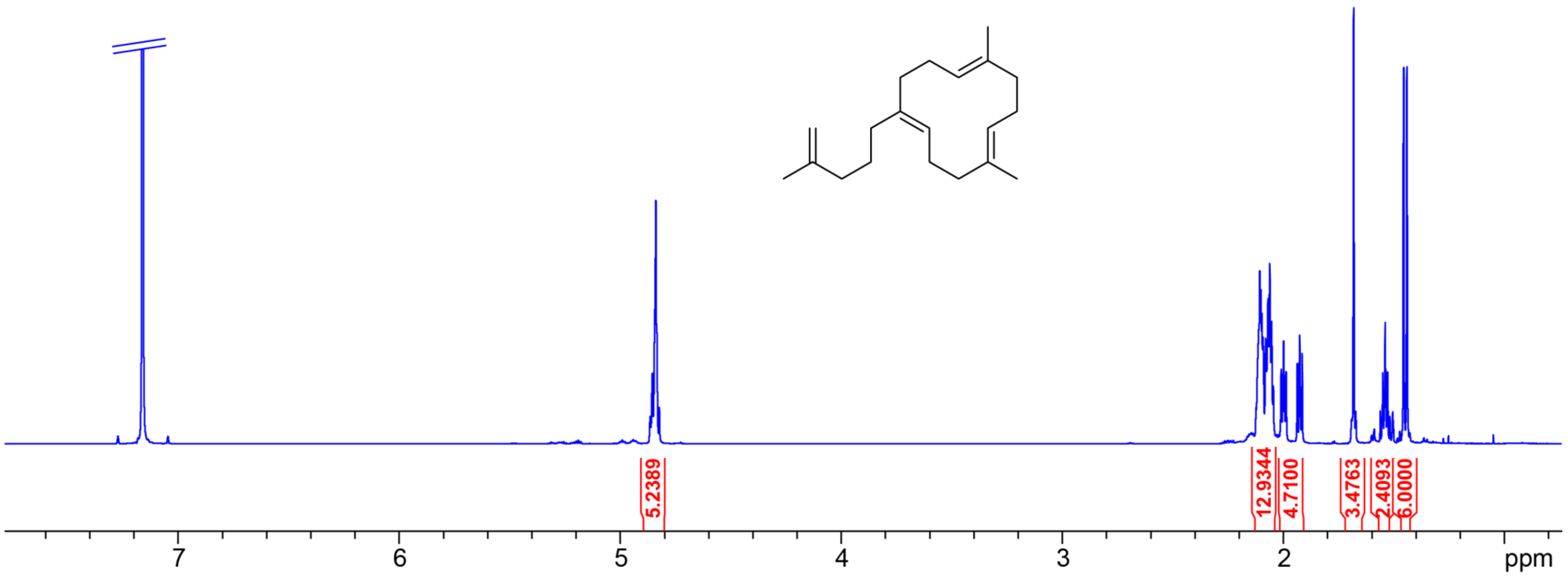

Figure S83. ^{1}H-NMR spectrum of **52** (700 MHz, C_6D_6).

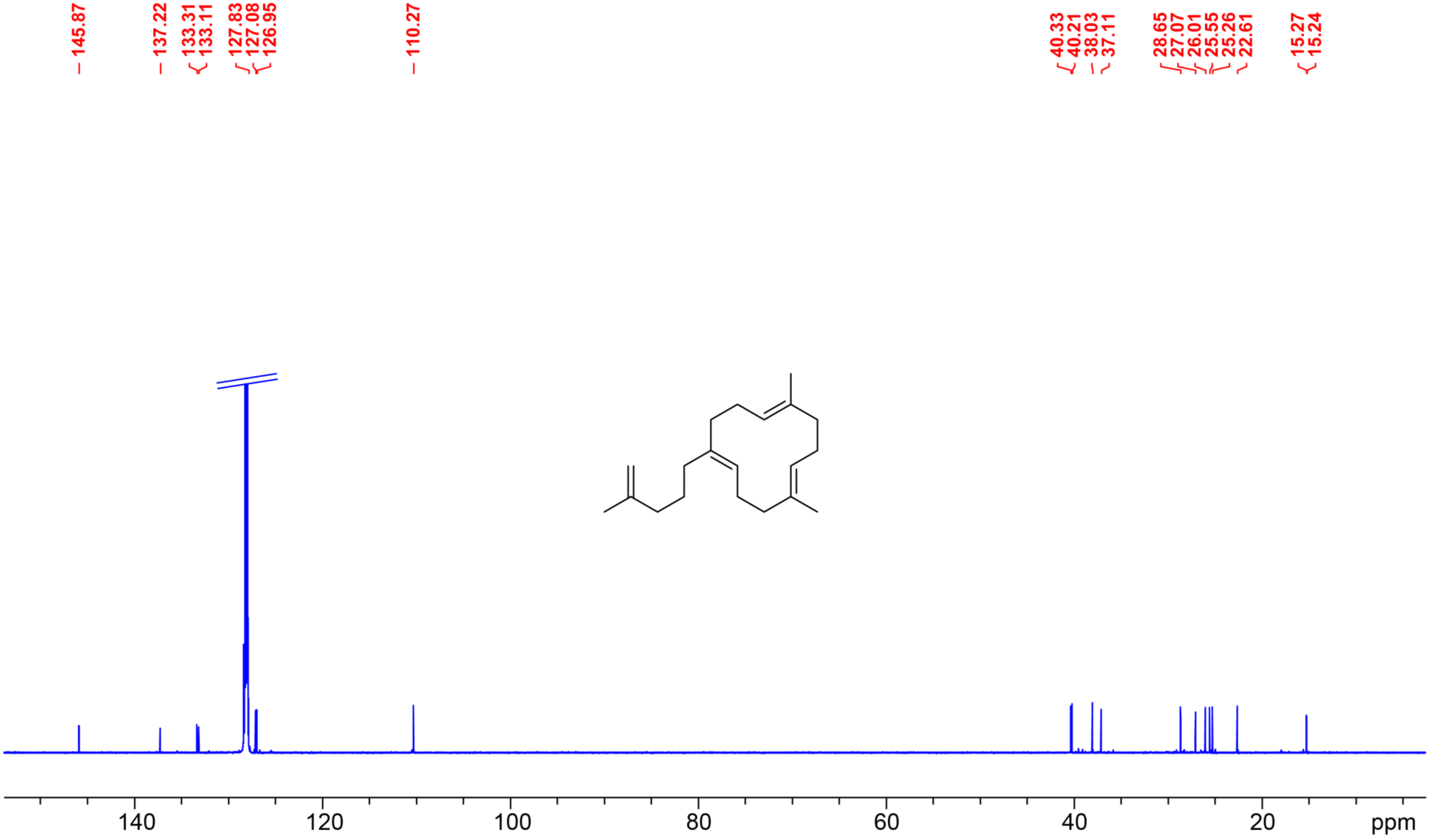

Figure S84. ^{13}C-NMR spectrum of **52** (176 MHz, C_6D_6).

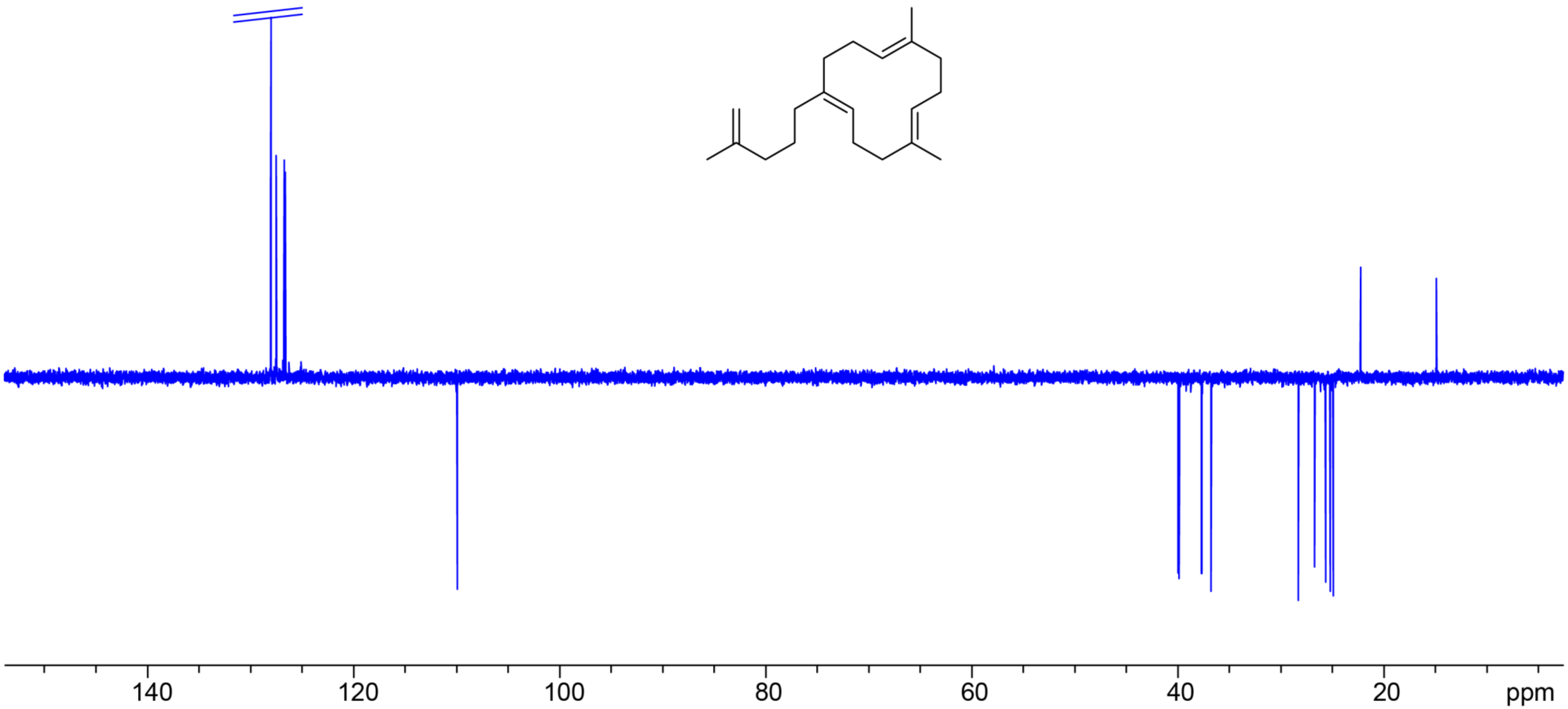

Figure S85. ^{13}C-DEPT135 spectrum of **52** (176 MHz, C_6D_6).

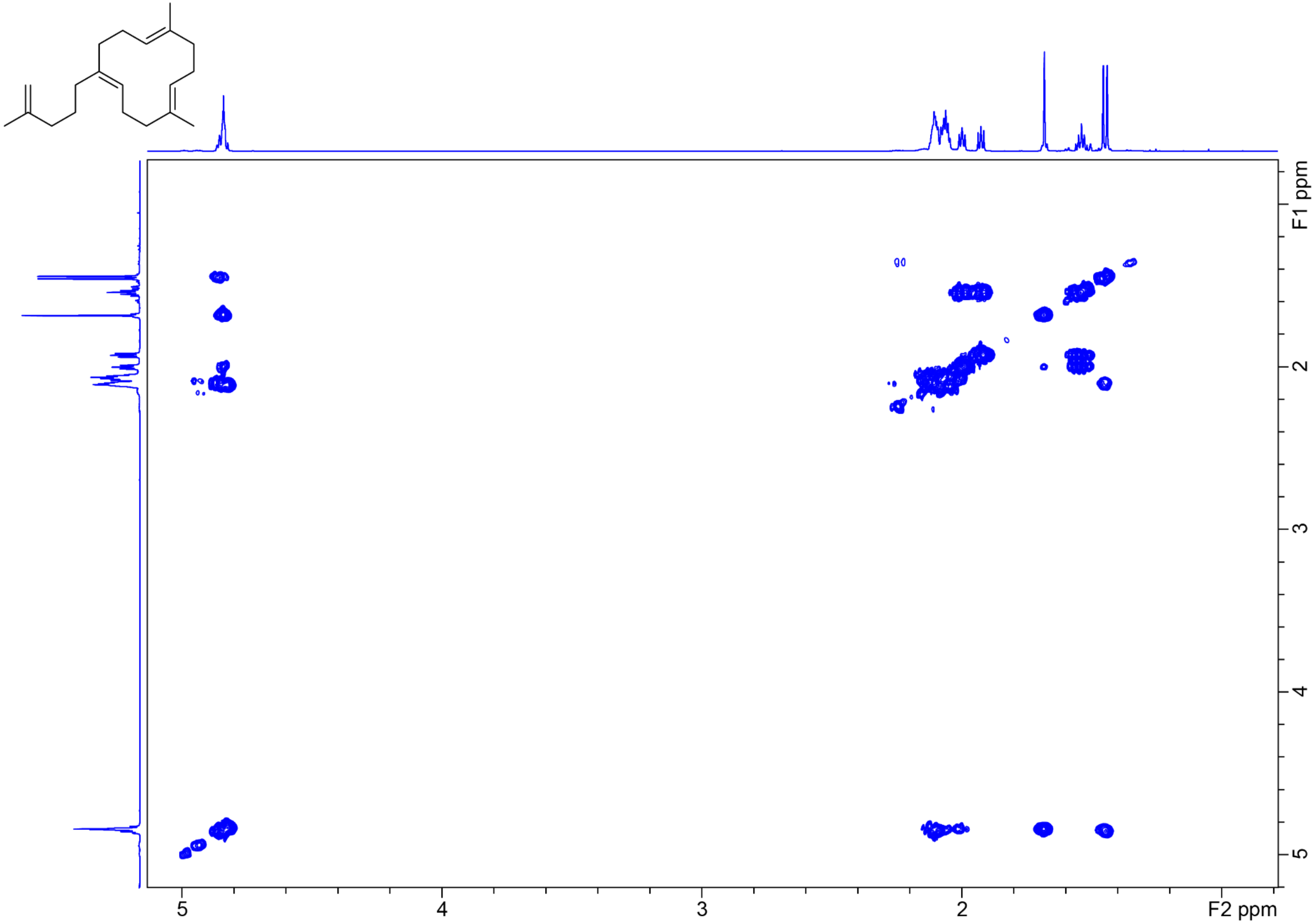

Figure S86. ^{1}H-^{1}H-COSY spectrum (C_6D_6) of **52**.

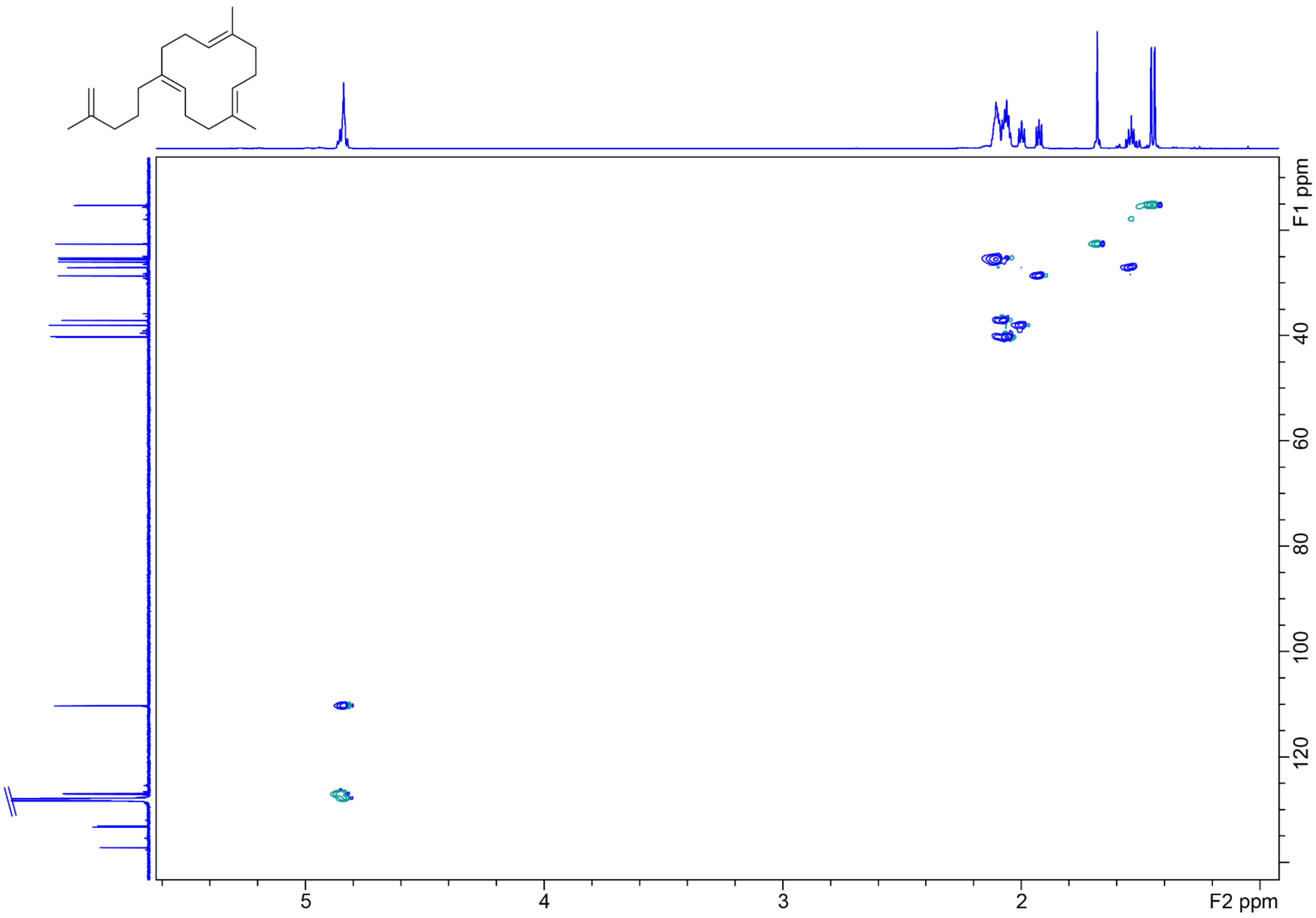

Figure S87. HSQC spectrum (C_6D_6) of **52**.

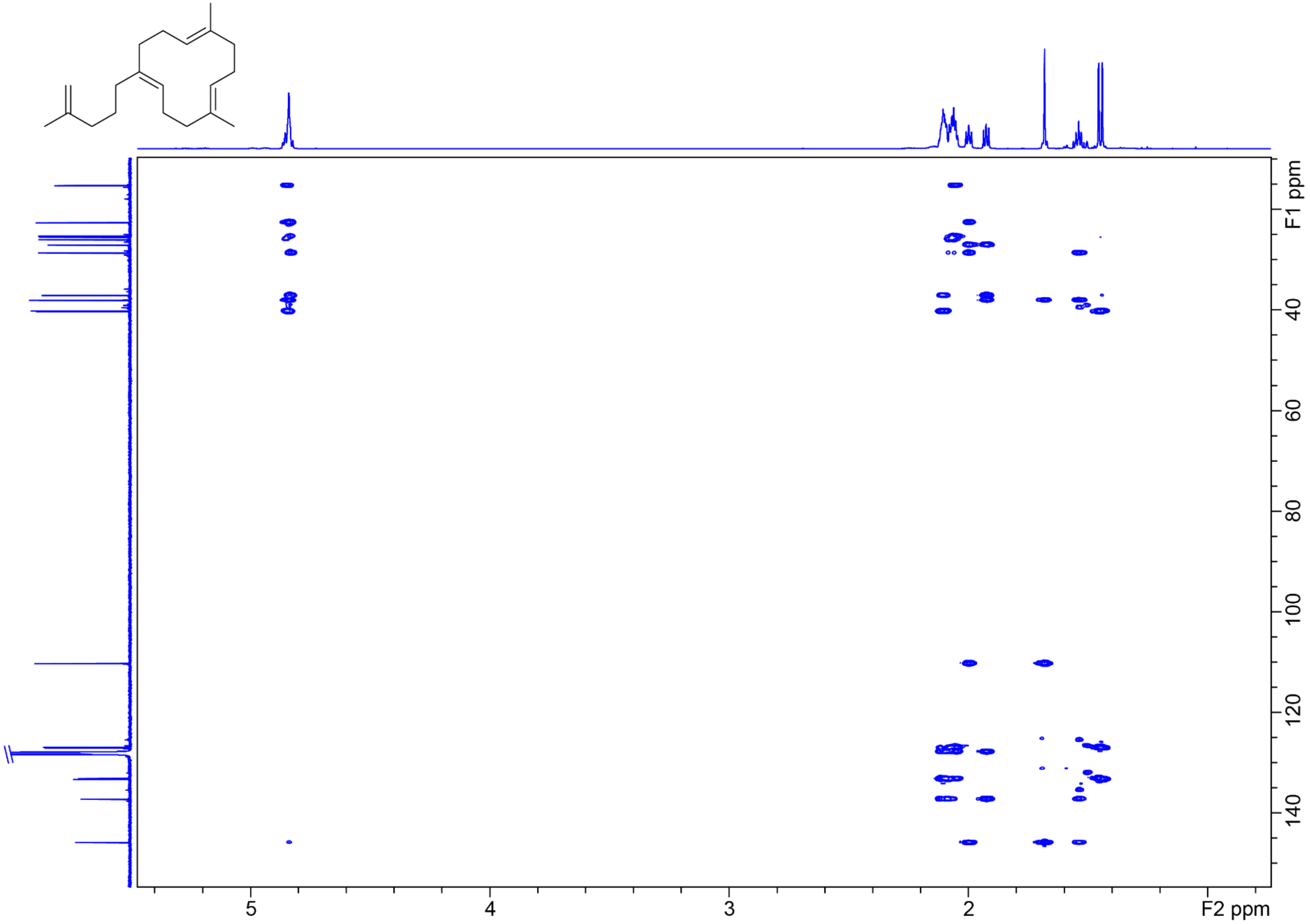

Figure S88. HMBC spectrum (C_6D_6) of **52**.

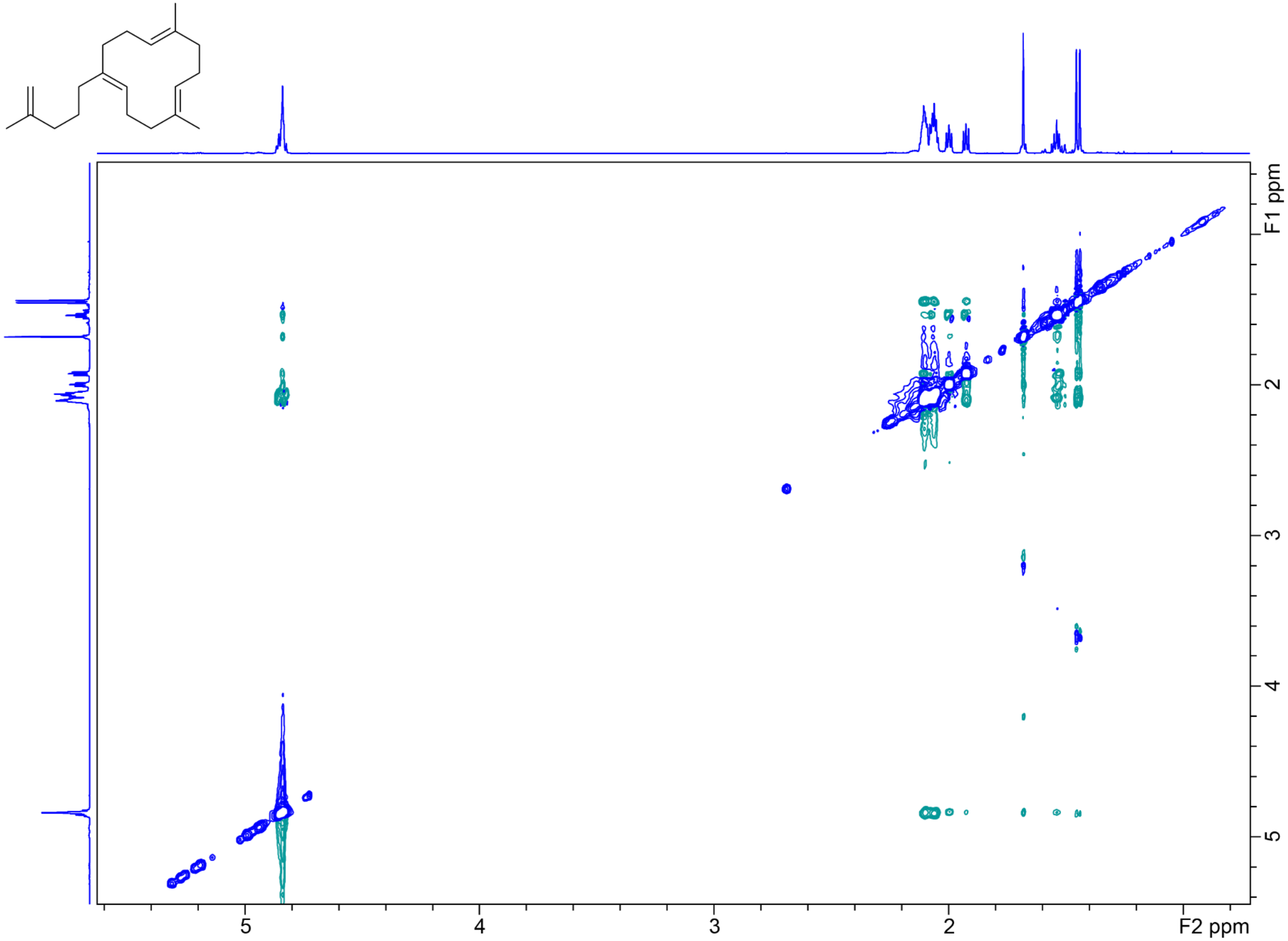

Figure S89. NOESY spectrum (C_6D_6) of **52**.

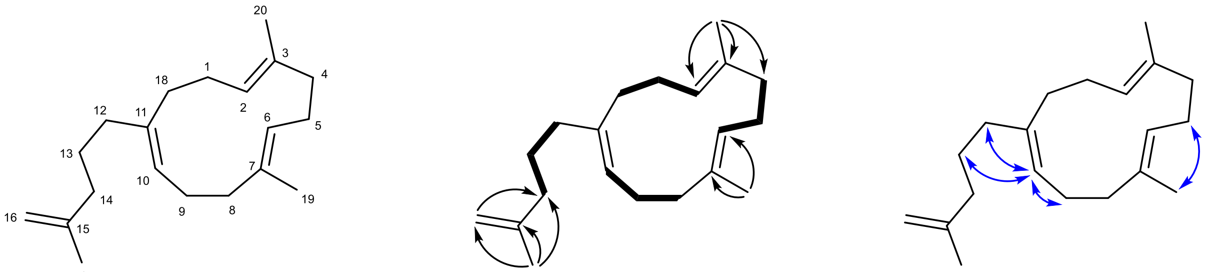

53

Figure S90. Structure elucidation of isopentenylpseudogermacrene C (**53**). Bold: ^{1}H,^{1}H-COSY, single headed arrows: key HMBC, and blue double headed arrows: NOESY correlations.

Table S14. NMR data of isopentenylpseudogermacrene C (**53**) in C_6D_6 recorded at 298 K.

C[a]	type	^{13}C[b]	^{1}H[b]
1	CH_2	24.95	2.15 (m, 2H)
2	CH	126.62	4.99 (br t, *J* = 7.3)
3	C_q	131.99	–
4	CH_2	39.07	2.01 (m, 2H)
5	CH_2	25.38	2.10 (m, 2H)
6	CH	125.41	4.94 (br t, *J* = 7.3)
7	C_q	135.40	–
8	CH_2	39.52	2.06 (m, 2H)
9	CH_2	28.24	2.13 (m, 2H)
10	CH	127.20	5.19 (br t, *J* = 7.5)
11	C_q	137.63	–
12	CH_2	35.81	2.06 (m, 2H)
13	CH_2	26.49	1.60 (m, 2H)
14	CH_2	37.97	2.01 (m, 2H)
15	C_q	145.87	–
16	CH_2	110.38	4.84 (m, 2H)
17	CH_3	22.54	1.67 (br s)
18	CH_2	29.06	2.09 (m, 2H)
19	CH_3	17.92	1.54 (d, *J* = 1.3)
20	CH_3	15.58	1.50 (d, *J* = 1.3)

[a] Carbon numbering as shown in Figure S90. [b] Chemical shifts δ in ppm, multiplicity: s = singlet, d = doublet, t = triplet, m = multiplet, br = broad, coupling constants *J* are given in Hertz.

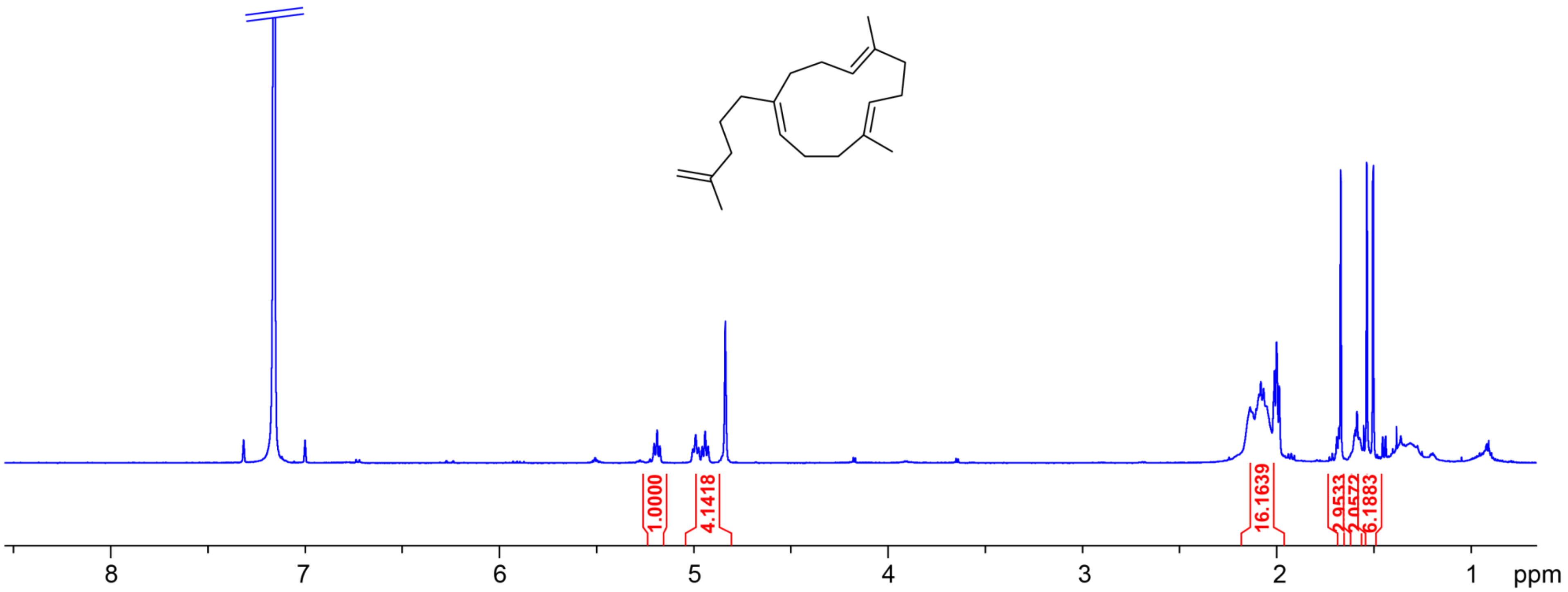

Figure S91. ^{1}H-NMR spectrum of **53** (700 MHz, C_6D_6).

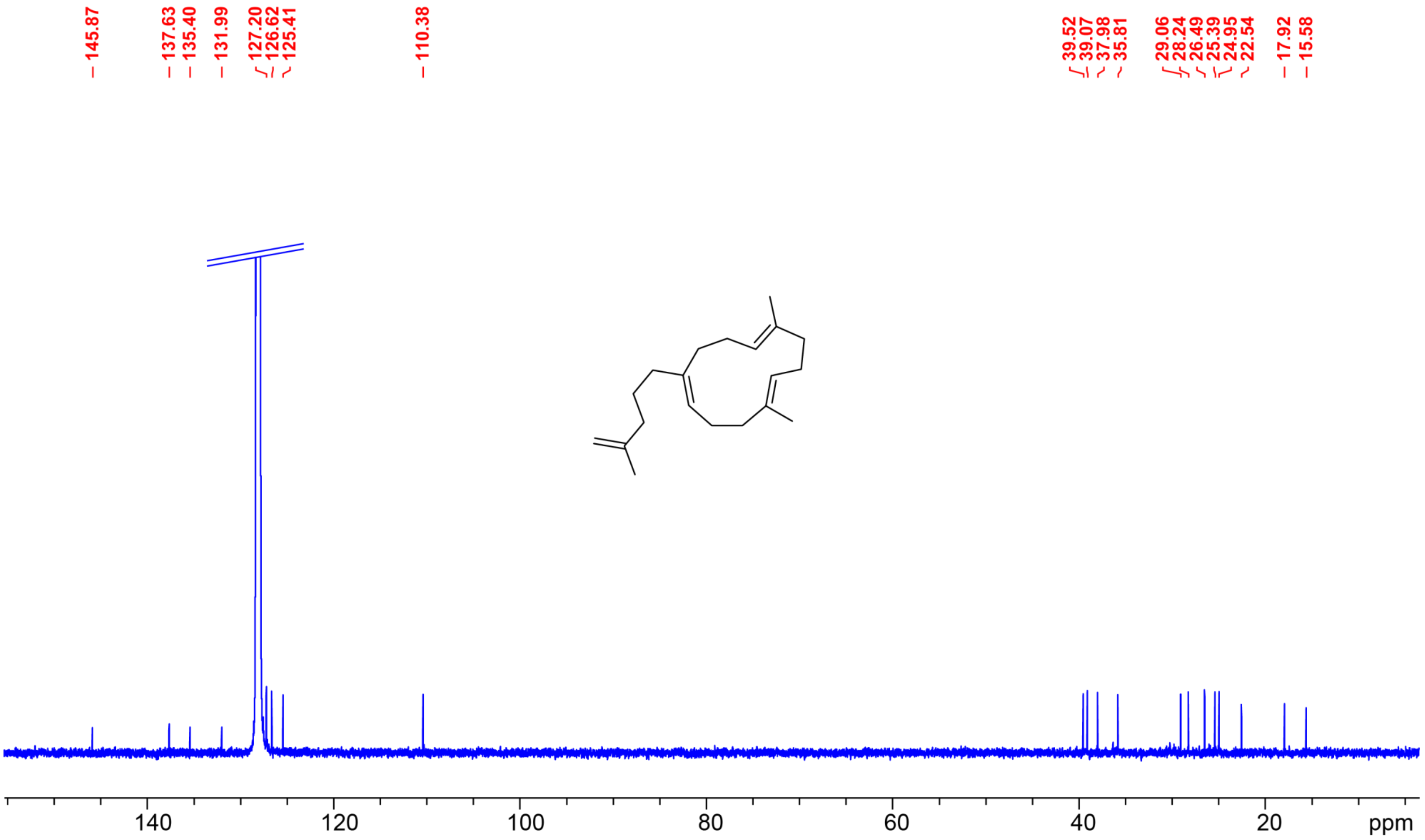

Figure S92. ^{13}C-NMR spectrum of **53** (176 MHz, C_6D_6).

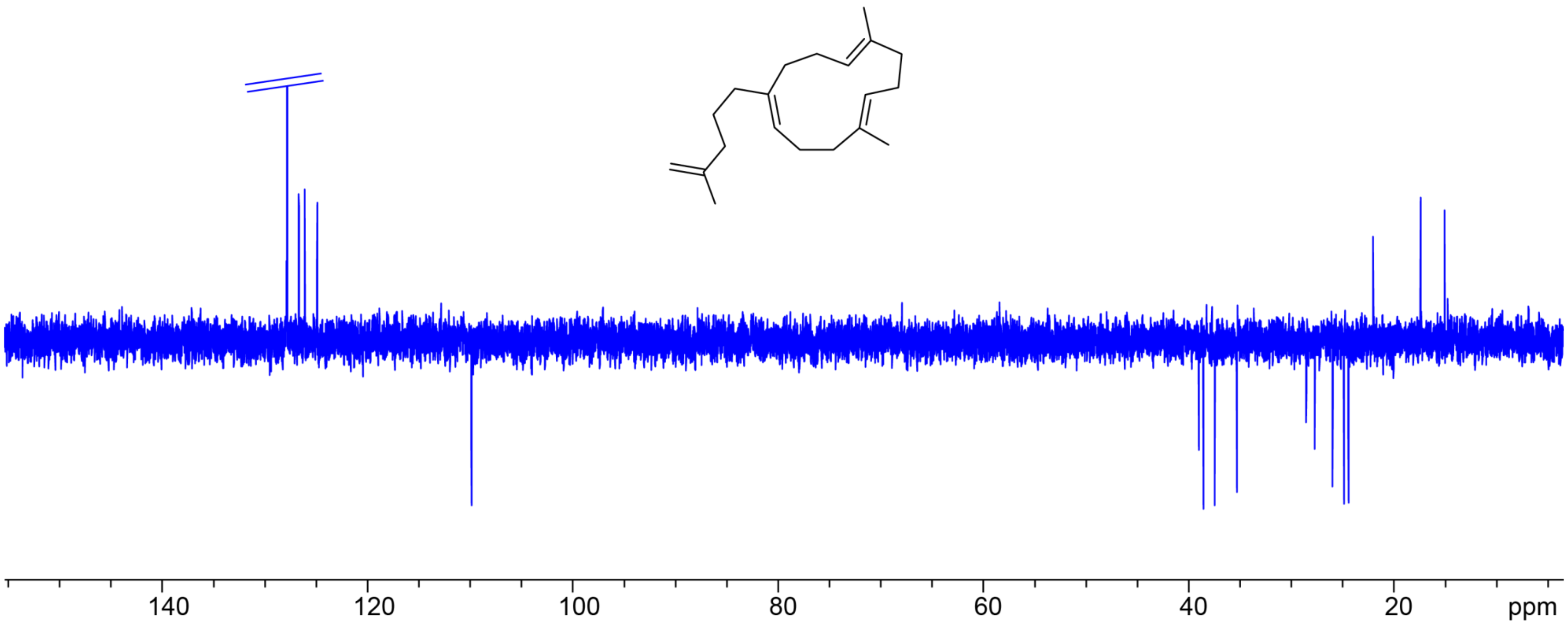

Figure S93. ^{13}C-DEPT135 spectrum of **53** (176 MHz, C_6D_6).

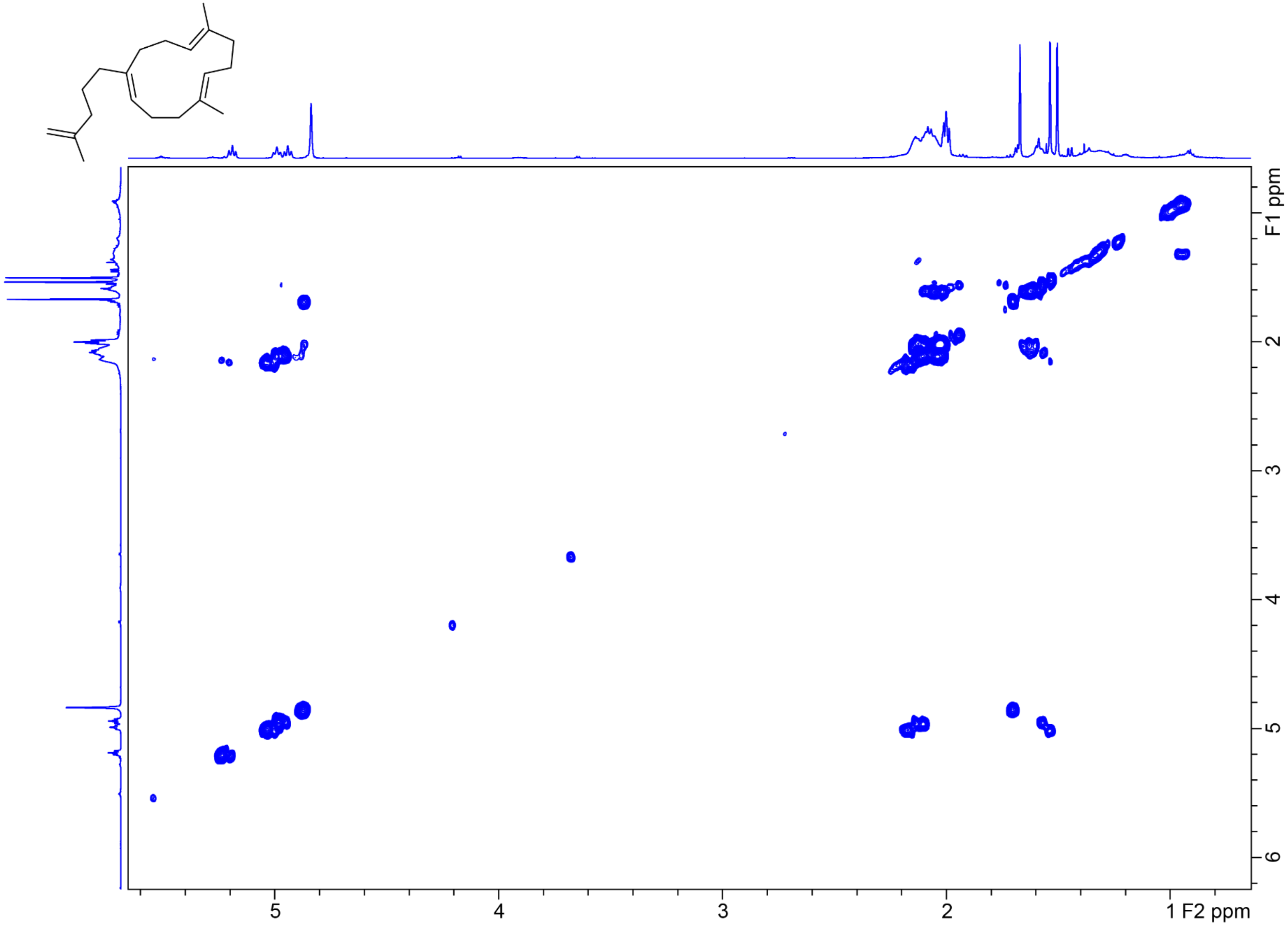

Figure S94. ^{1}H-^{1}H-COSY spectrum (C_6D_6) of **53**.

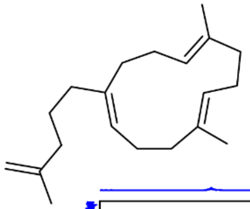

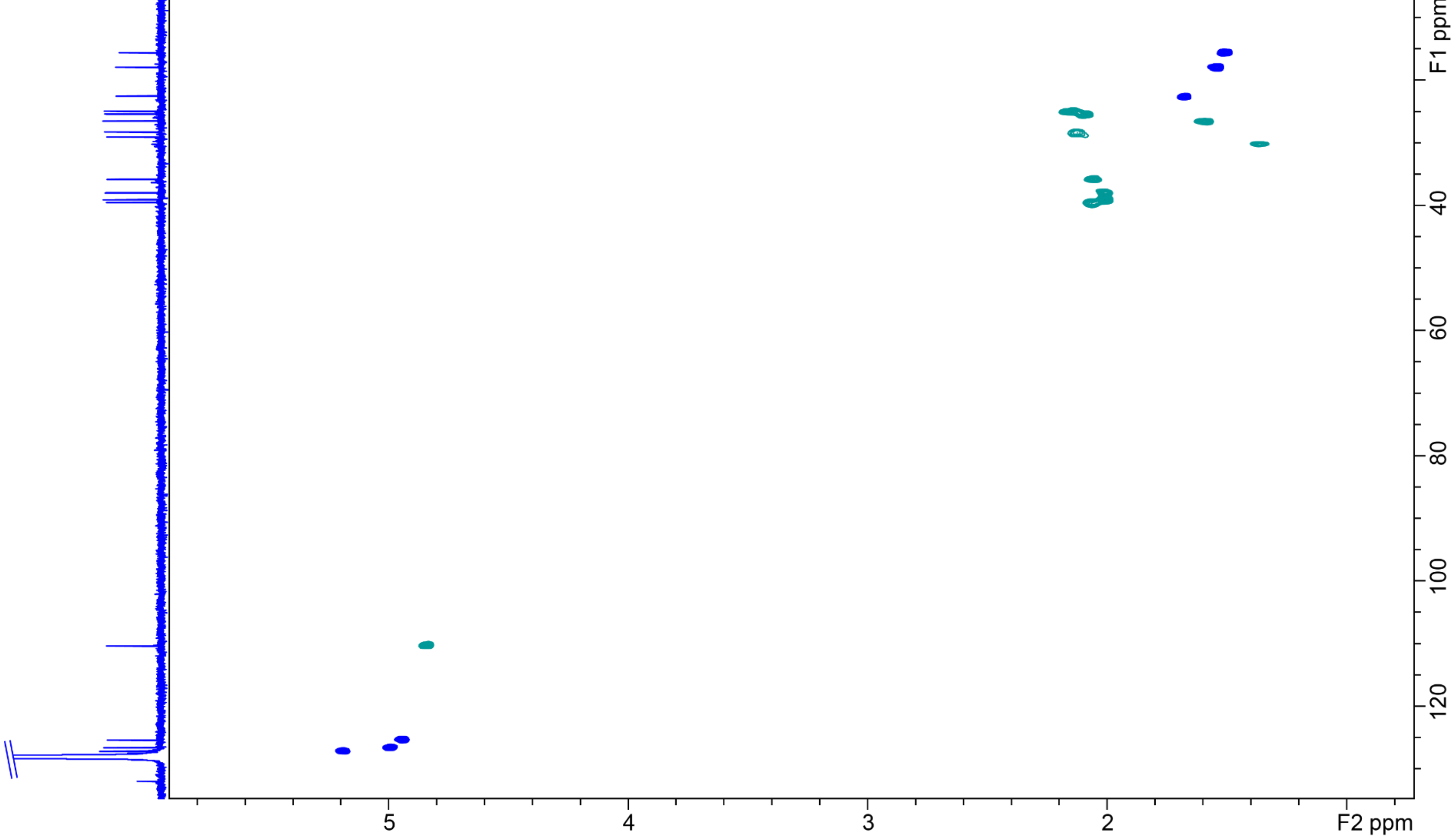

Figure S95. HSQC spectrum (C_6D_6) of **53**.

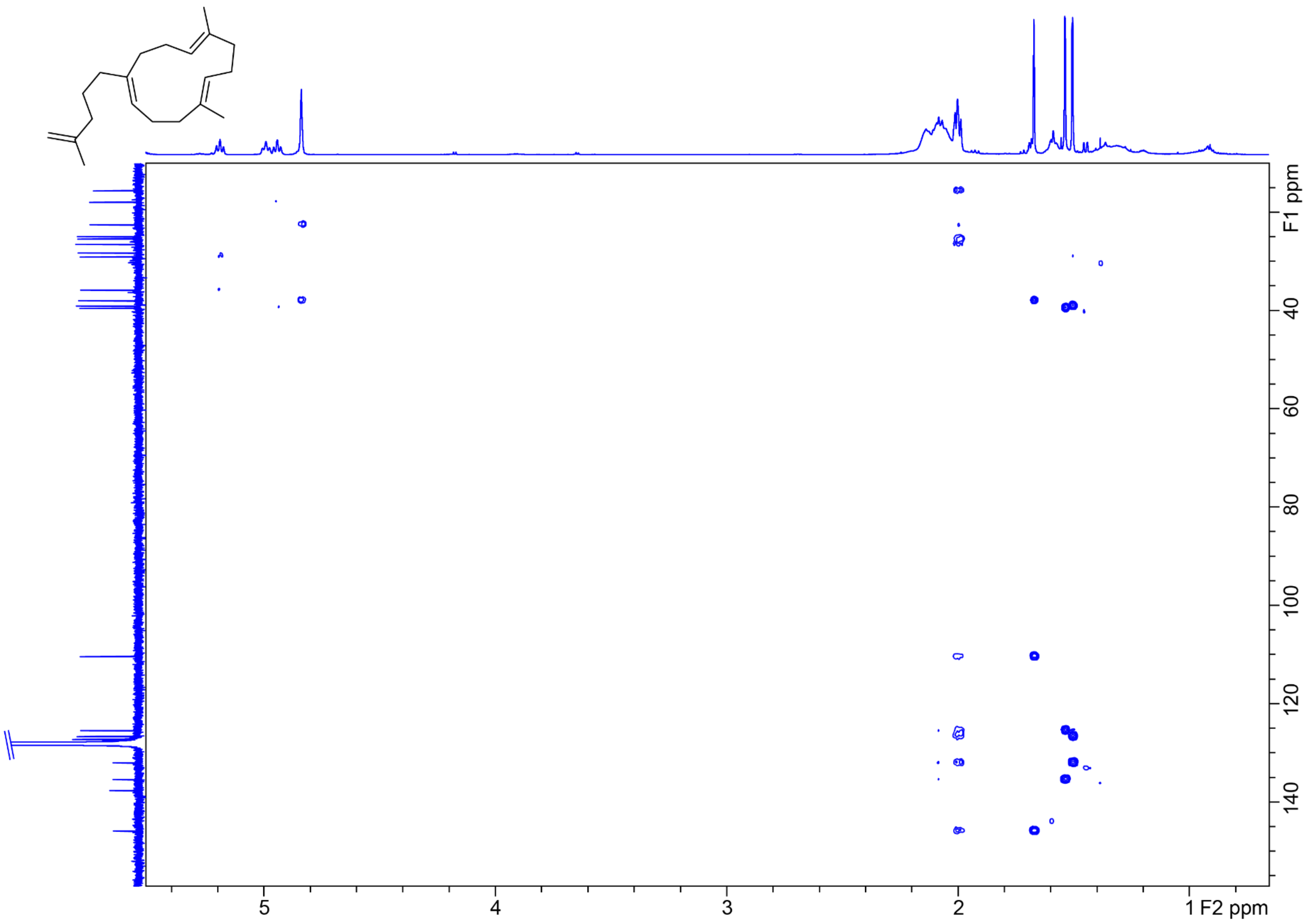

Figure S96. HMBC spectrum (C_6D_6) of **53**.

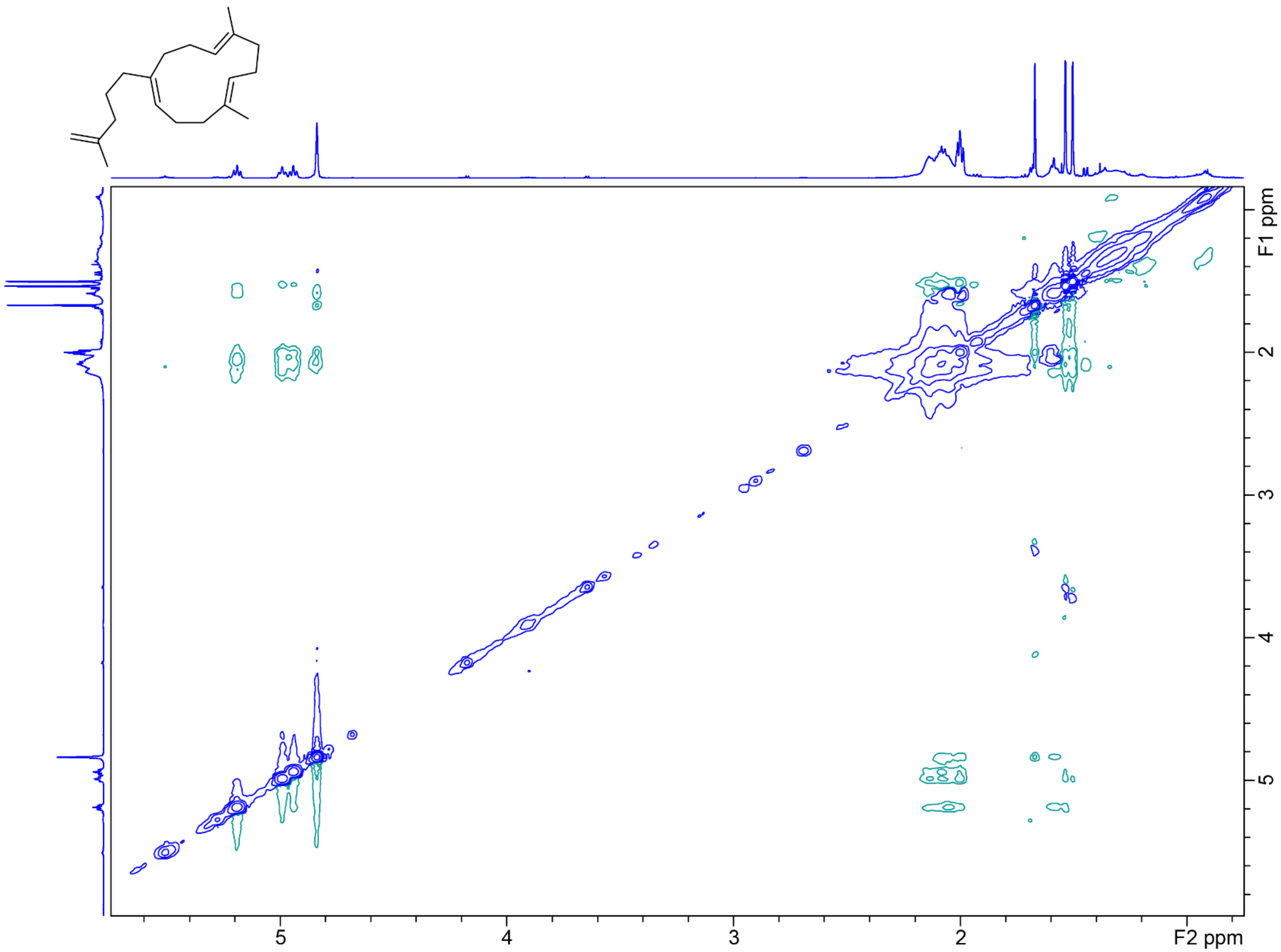

Figure S97. NOESY spectrum (C_6D_6) of **53**.

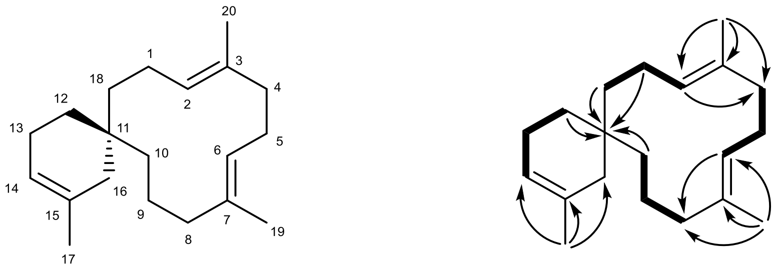

54

Figure S98. Structure elucidation of α-spirocattleyaxenene (**54**). Bold: ^{1}H,^{1}H-COSY, and single headed arrows: key HMBC correlations.

Table S15. NMR data of α-spirocattleyaxenene (**54**) in C_6D_6 recorded at 298 K.

C[a]	type	^{13}C[b]	^{1}H[b]
1	CH_2	23.58	2.08 (m, 2H)
2	CH	129.87	5.10 (dd, *J* = 7.7, 7.7)
3	C_q	131.59	–
4	CH_2	39.86	2.04 (m, 2H)
5	CH_2	25.65	2.17 (m)<br>2.13 (m)
6	CH	126.11	4.92 (dd, *J* = 7.7, 7.7)
7	C_q	134.63	–
8	CH_2	38.96	1.93 (m)<br>1.89 (m)
9	CH_2	22.09	1.23 (m)<br>1.20 (m)
10	CH_2	35.32	1.33 (m, 2H)
11	C_q	34.98	–
12	CH_2	32.07	1.37 (m)<br>1.33 (m)
13	CH_2	23.12	1.98 (m, 2H)
14	CH	120.73	5.43 (m)
15	C_q	132.47	–
16	CH_2	42.36	1.79 (d, *J* = 17.1)<br>1.58 (d, *J* = 17.1)
17	CH_3	24.39	1.67 (br s)
18	CH_2	35.36	1.54 (m)<br>1.18 (m)
19	CH_3	17.68	1.54 (br s)
20	CH_3	15.29	1.49 (br s)

[a] Carbon numbering as shown in Figure S98. [b] Chemical shifts δ in ppm, multiplicity: s = singlet, d = doublet, m = multiplet, br = broad, coupling constants *J* are given in Hertz.

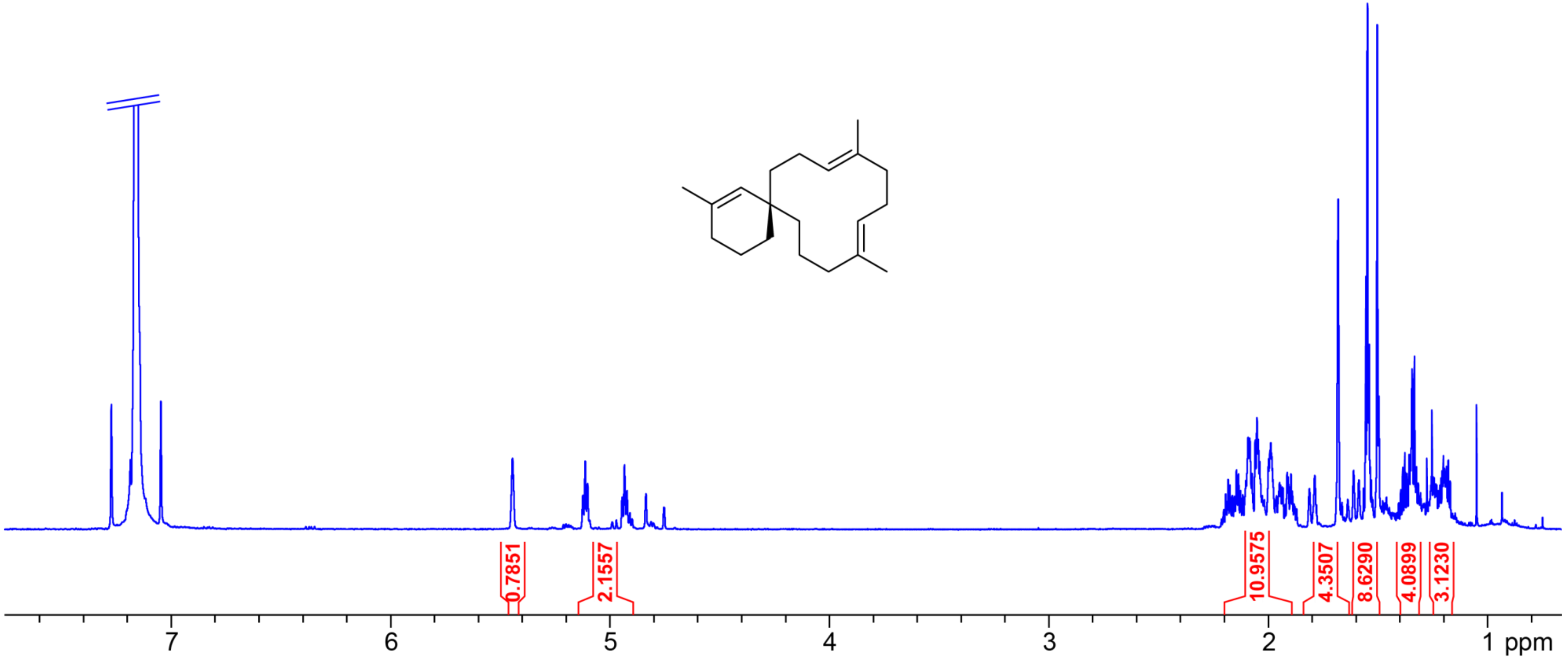

Figure S99. ^{1}H-NMR spectrum of **54** (700 MHz, C_6D_6).

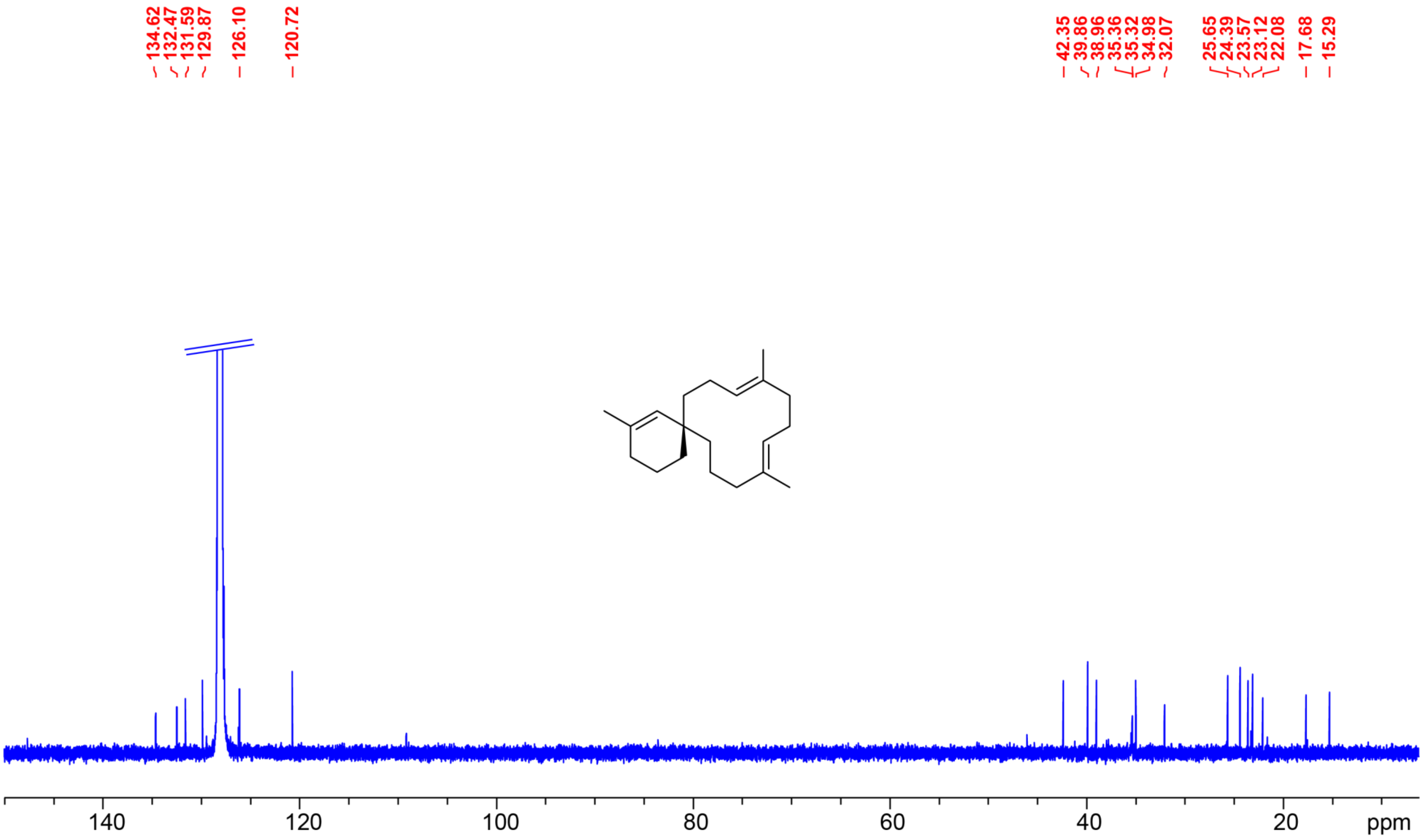

Figure S100. ^{13}C-NMR spectrum of **54** (176 MHz, C_6D_6).

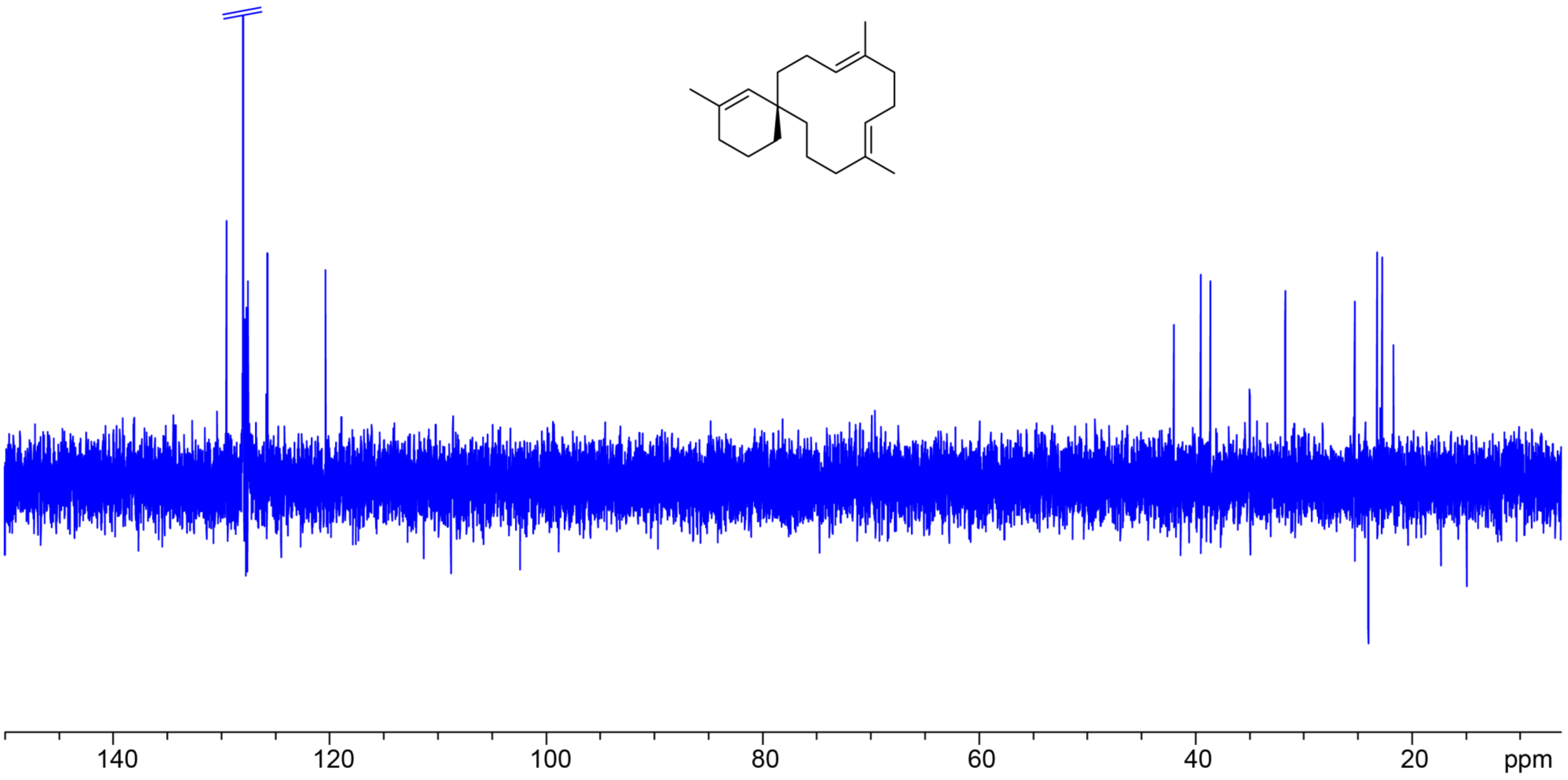

Figure S101. ^{13}C-DEPT135 spectrum of **54** (176 MHz, C_6D_6).

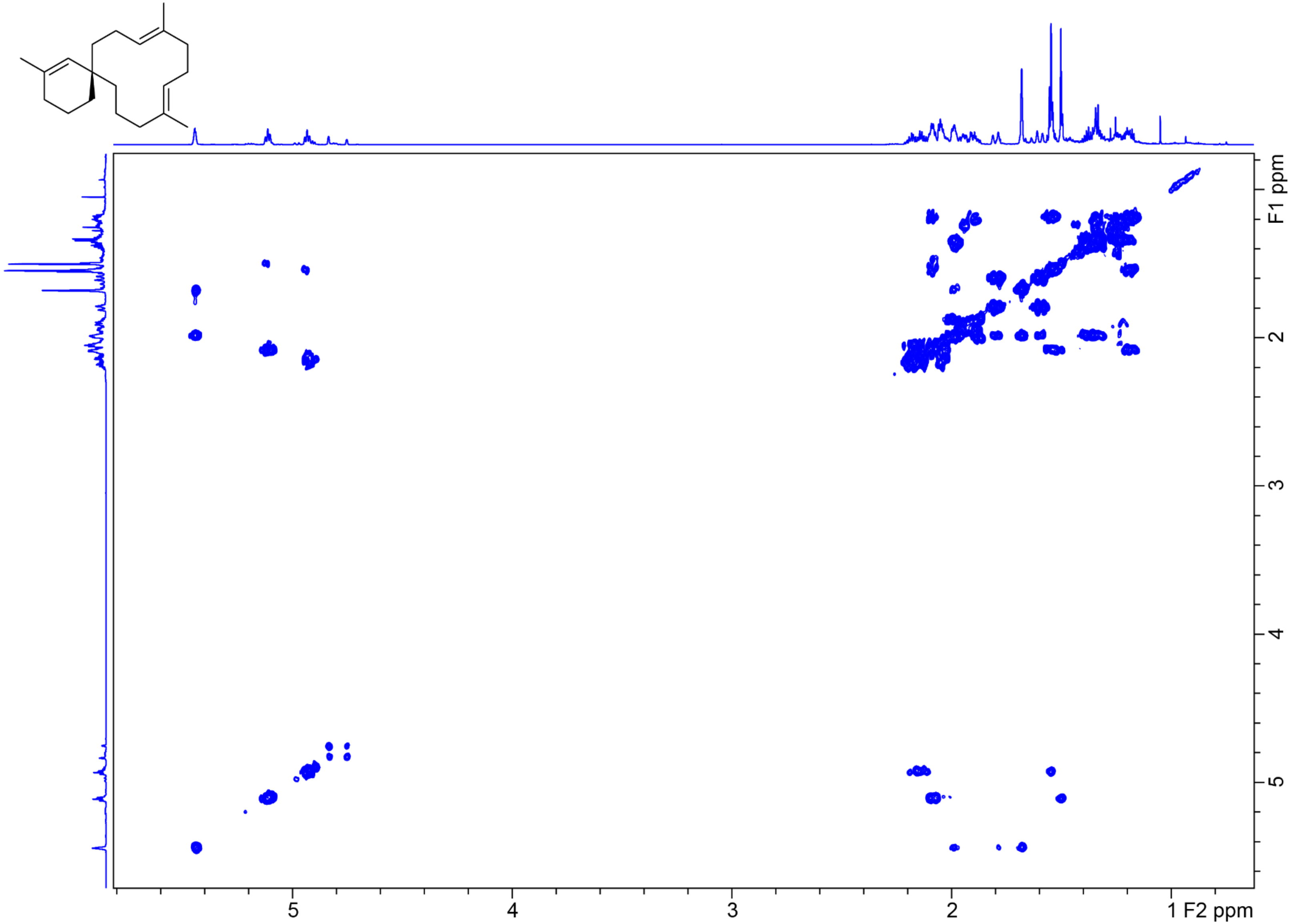

Figure S102. ^{1}H-^{1}H-COSY spectrum (C_6D_6) of **54**.

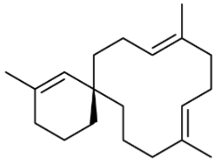

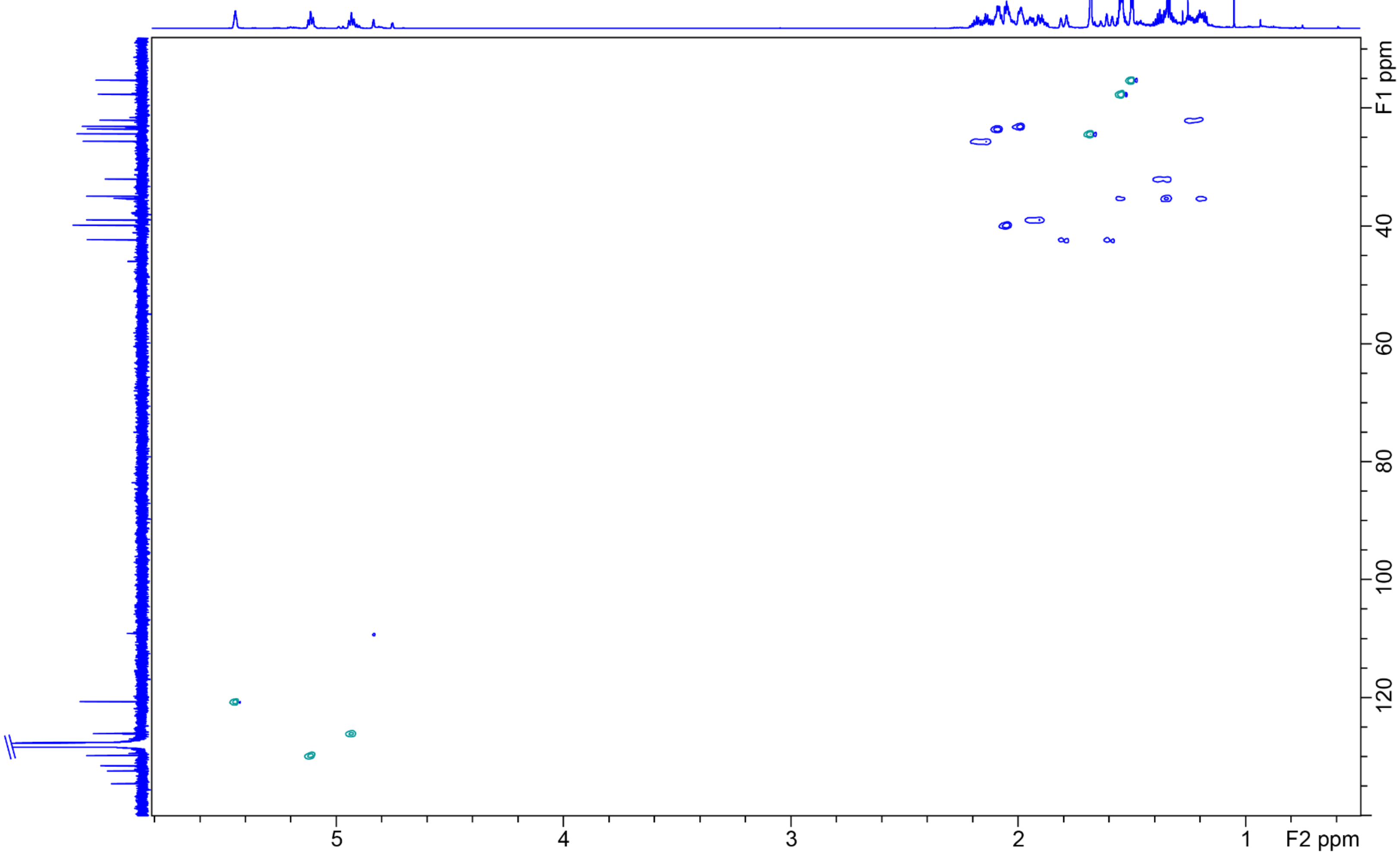

Figure S103. HSQC spectrum (C_6D_6) of **54**.

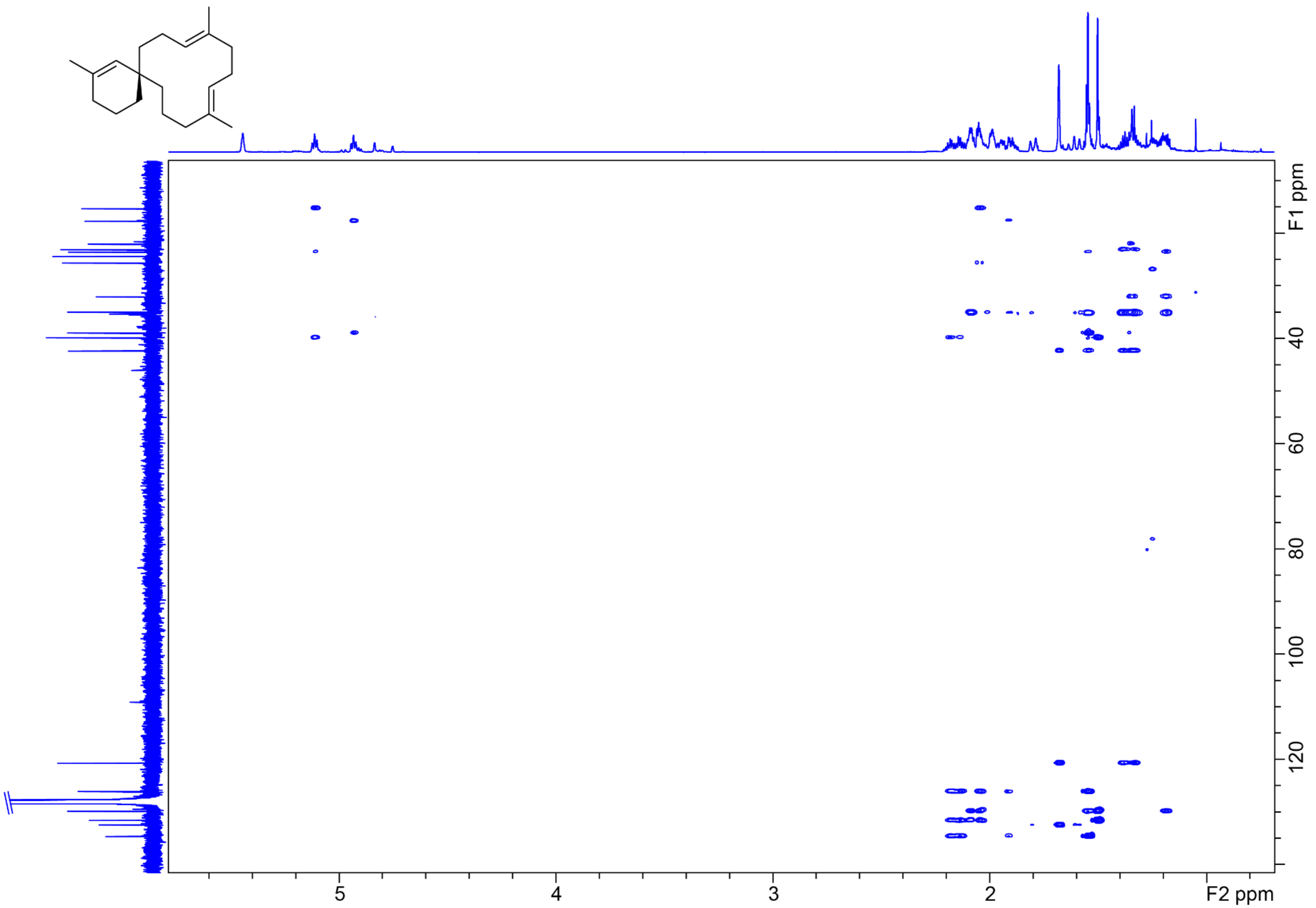

Figure S104. HMBC spectrum (C_6D_6) of **54**.

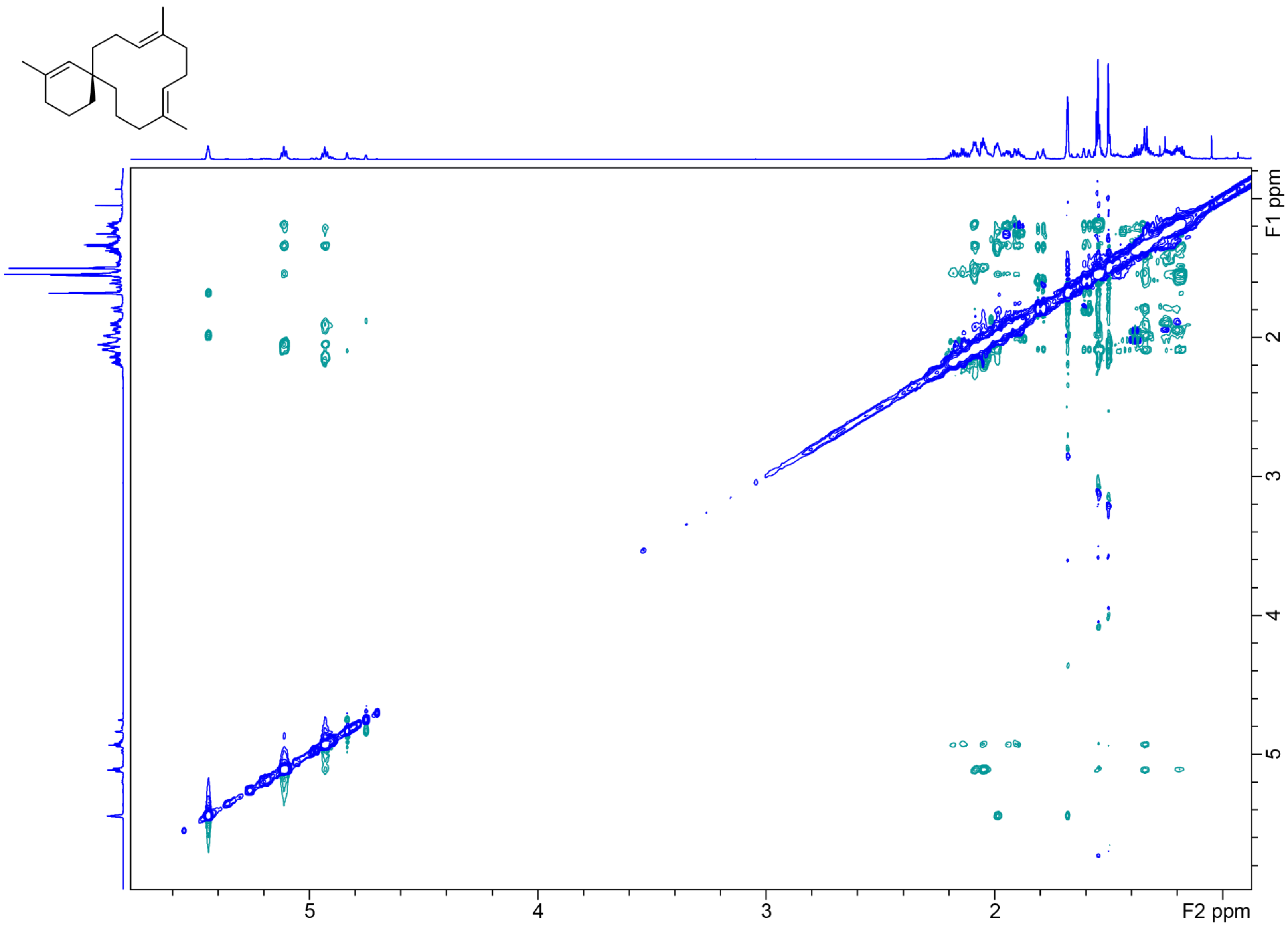

Figure S105. NOESY spectrum (C_6D_6) of **54**.

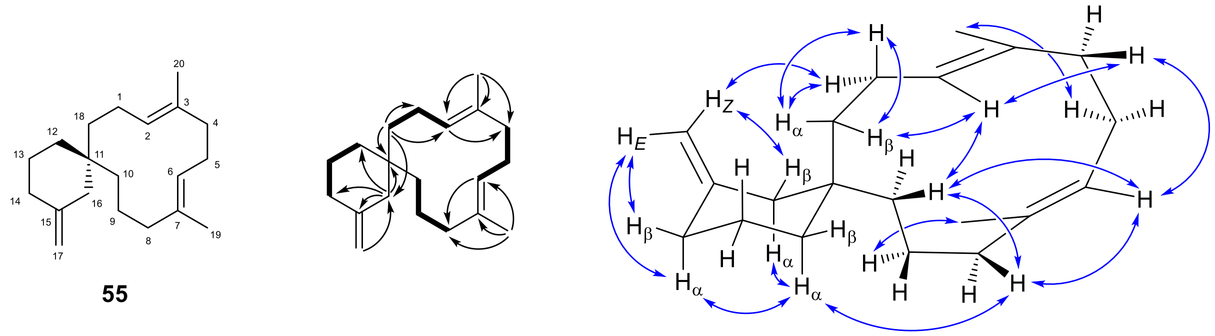

Figure S106. Structure elucidation of β-spirocattleyaxenene (**55**). Bold: $^{1}H,^{1}H$-COSY, single headed arrows: key HMBC, and blue double headed arrows: NOESY correlations.

Table S16. NMR data of β-spirocattleyaxenene (**55**) in C_6D_6 recorded at 298 K.

C[a]	type	^{13}C[b]	^{1}H[b]
1	CH_2	23.27	2.09 (m, $H_α$)<br>2.03 (m, $H_β$)
2	CH	129.46	5.10 (tqt, J = 7.7, 1.1, 1.1)
3	C_q	131.73	–
4	CH_2	39.86	2.04 (m, 2H)
5	CH_2	25.73	2.15 (m, 2H)
6	CH	126.22	4.91 (tm, J = 7.6)
7	C_q	134.52	–
8	CH_2	39.01	1.91 (m, 2H)
9	CH_2	21.60	1.18 (m, 2H)
10	CH_2	35.17	1.31 (m, 2H)
11	C_q	37.94	–
12	CH_2	35.84	1.30 (m, $H_α$)<br>1.26 (m, $H_β$)
13	CH_2	23.27	1.47 (m, 2H)
14	CH_2	35.47	2.10 (m, $H_β$)<br>2.05 (m, $H_α$)
15	C_q	147.67	–
16	CH_2	46.03	2.01 (d, J = 12.9, $H_α$)<br>1.88 (d, J = 12.9, $H_β$)
17	CH_2	109.15	4.83 (br s, H_E)<br>4.75 (br s, H_Z)
18	CH_2	34.98	1.43 (m, $H_α$)<br>1.24 (m, $H_β$)
19	CH_3	17.52	1.54 (m)
20	CH_3	15.30	1.49 (m)

[a] Carbon numbering as shown in Figure S106. [b] Chemical shifts δ in ppm, multiplicity: s = singlet, d = doublet, t = triplet, q = quartet, m = multiplet, br = broad, coupling constants J are given in Hertz.

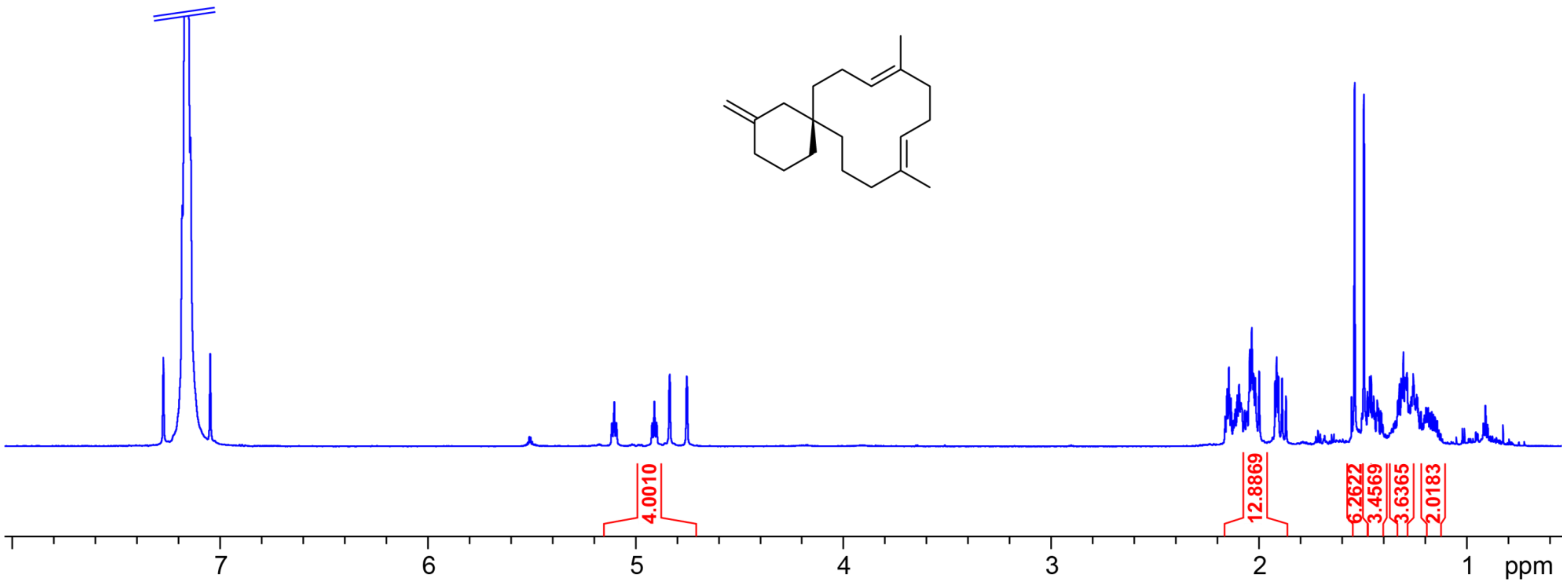

Figure S107. ^{1}H-NMR spectrum of **55** (700 MHz, C_6D_6).

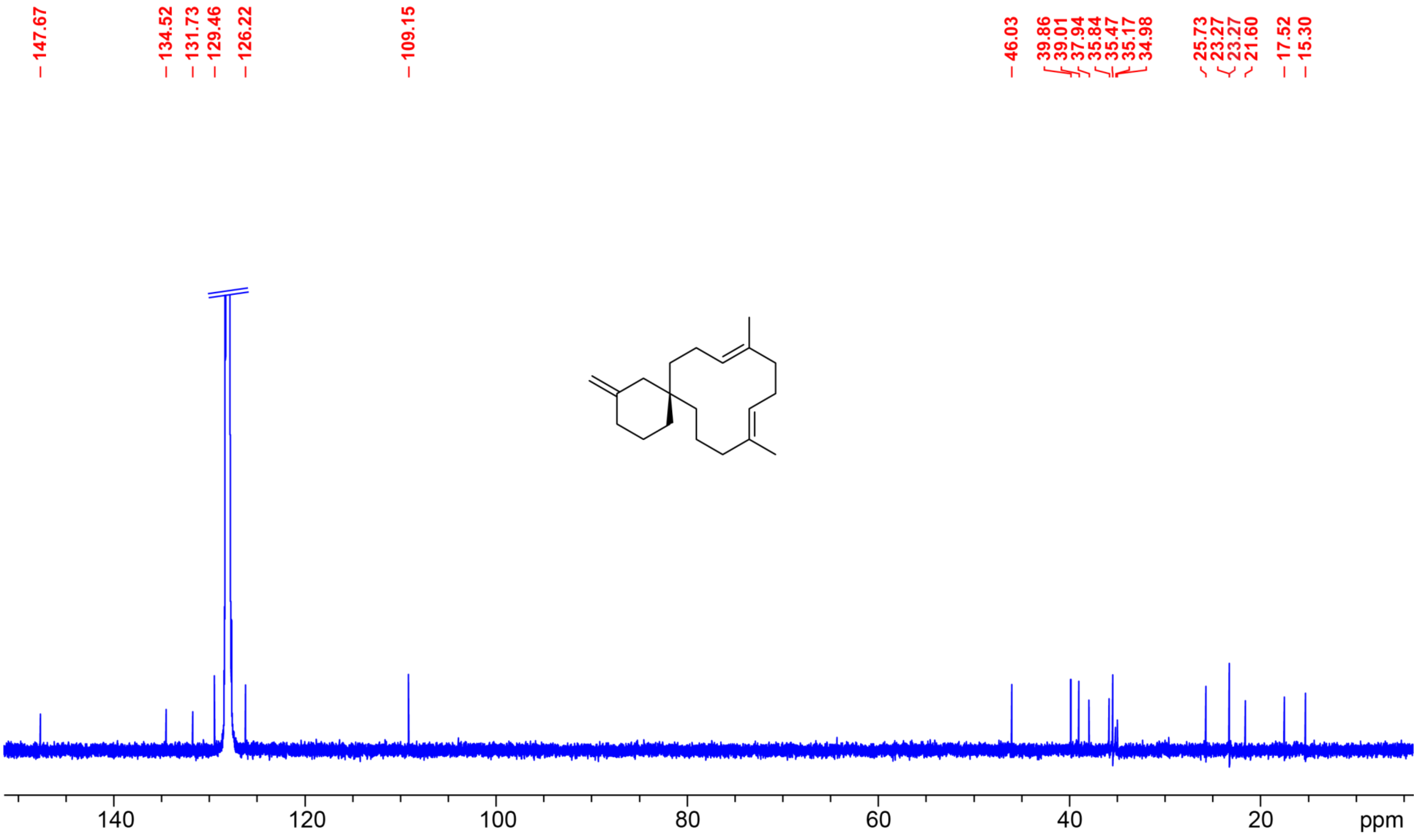

Figure S108. ^{13}C-NMR spectrum of **55** (176 MHz, C_6D_6).

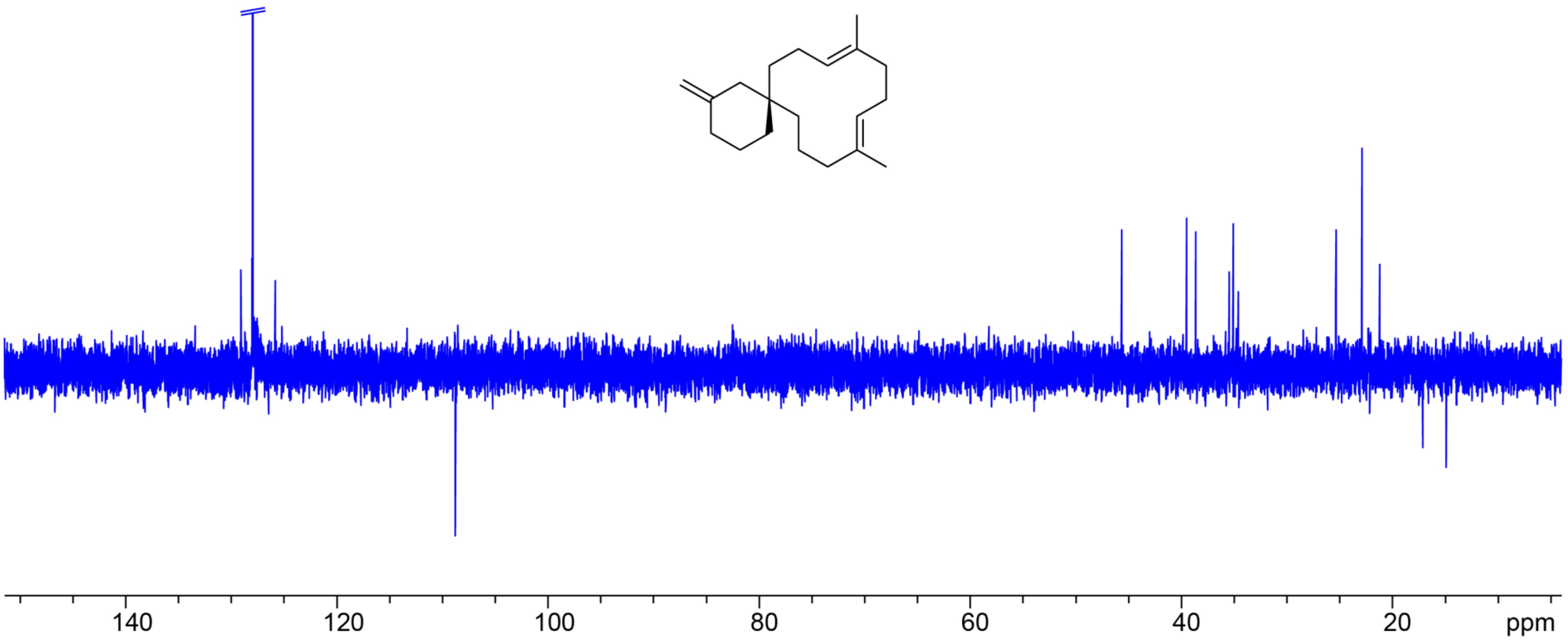

Figure S109. ^{13}C-DEPT135 spectrum of **55** (176 MHz, C_6D_6).

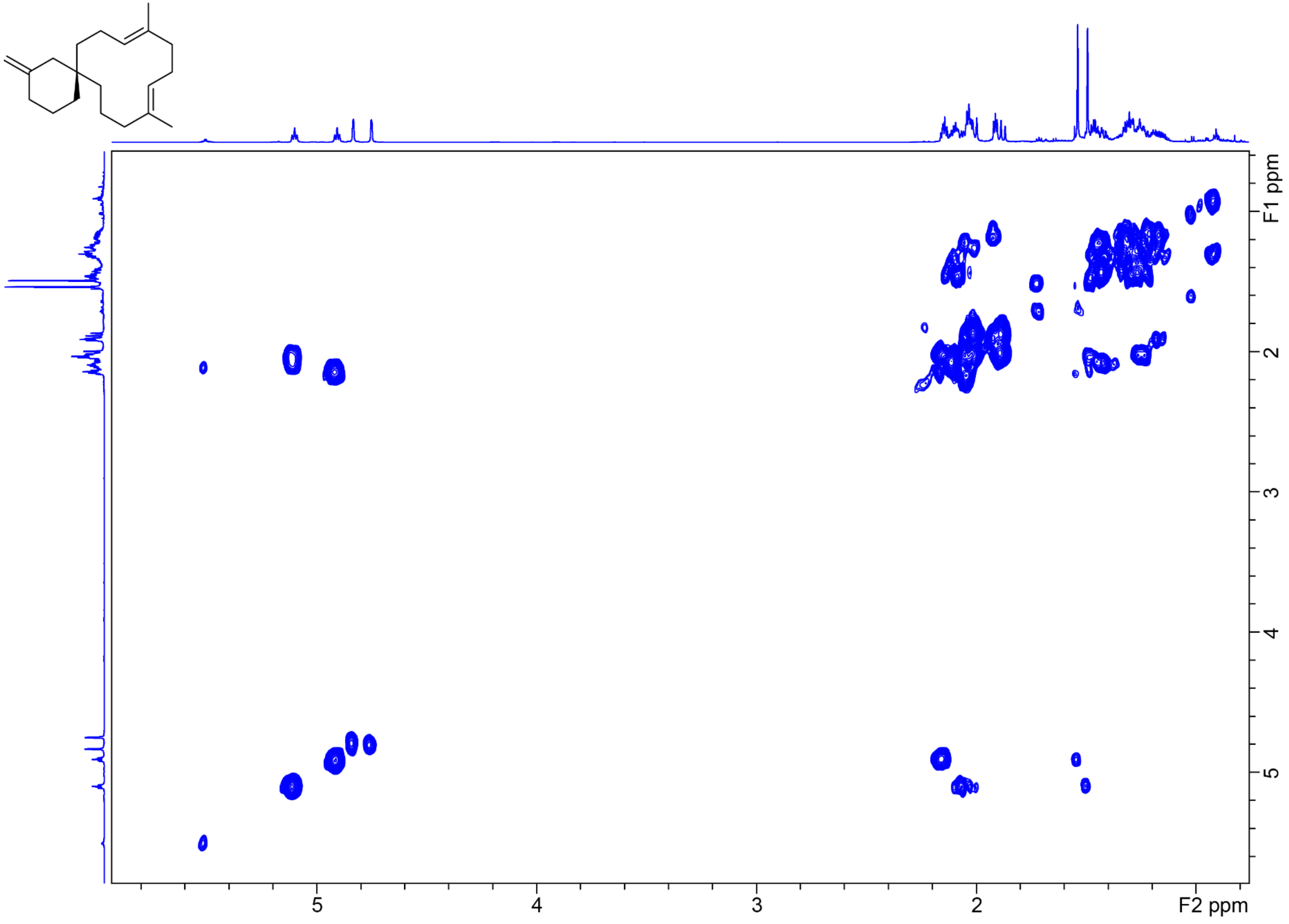

Figure S110. ^{1}H-^{1}H-COSY spectrum (C_6D_6) of **55**.

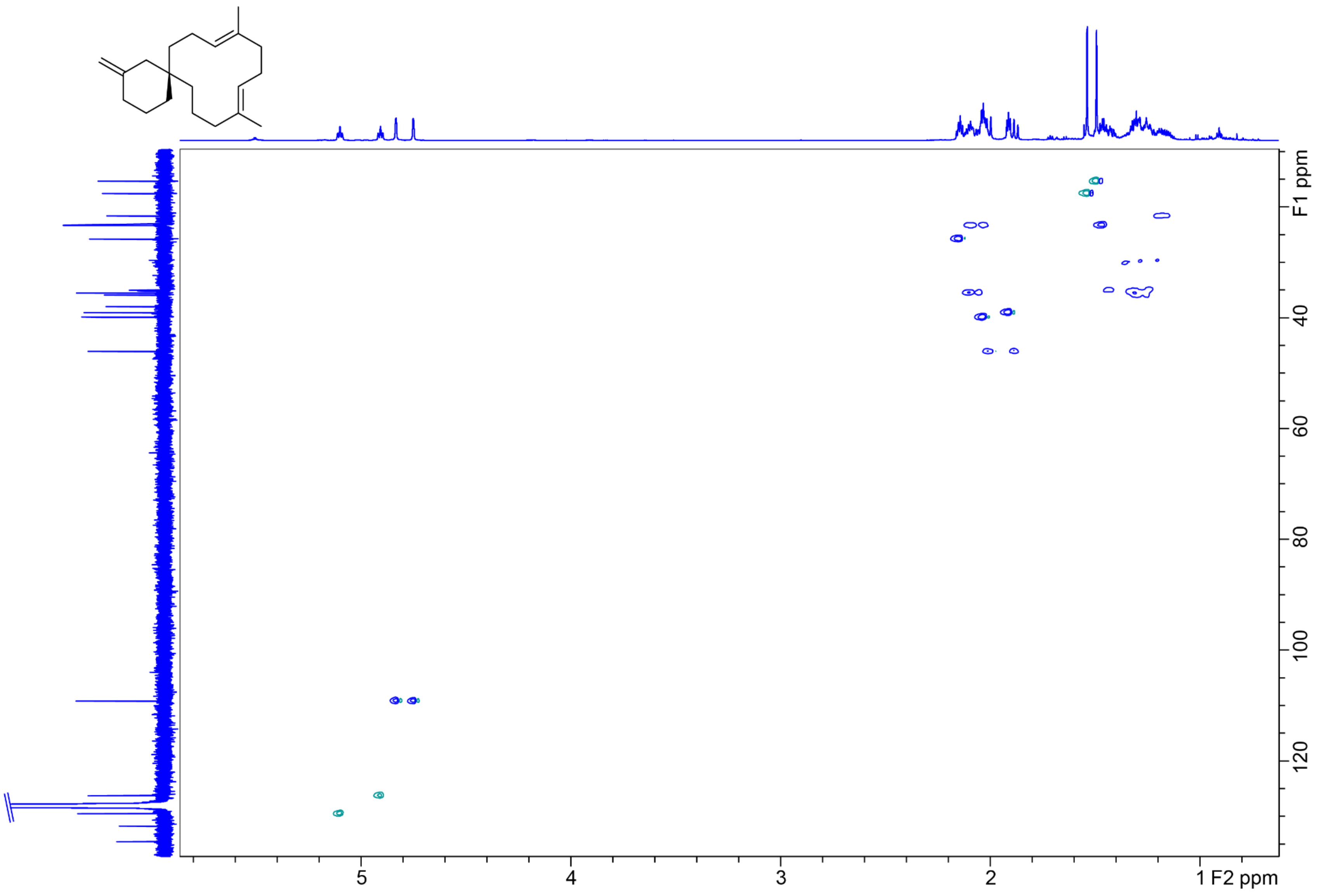

Figure S111. HSQC spectrum (C_6D_6) of **55**.

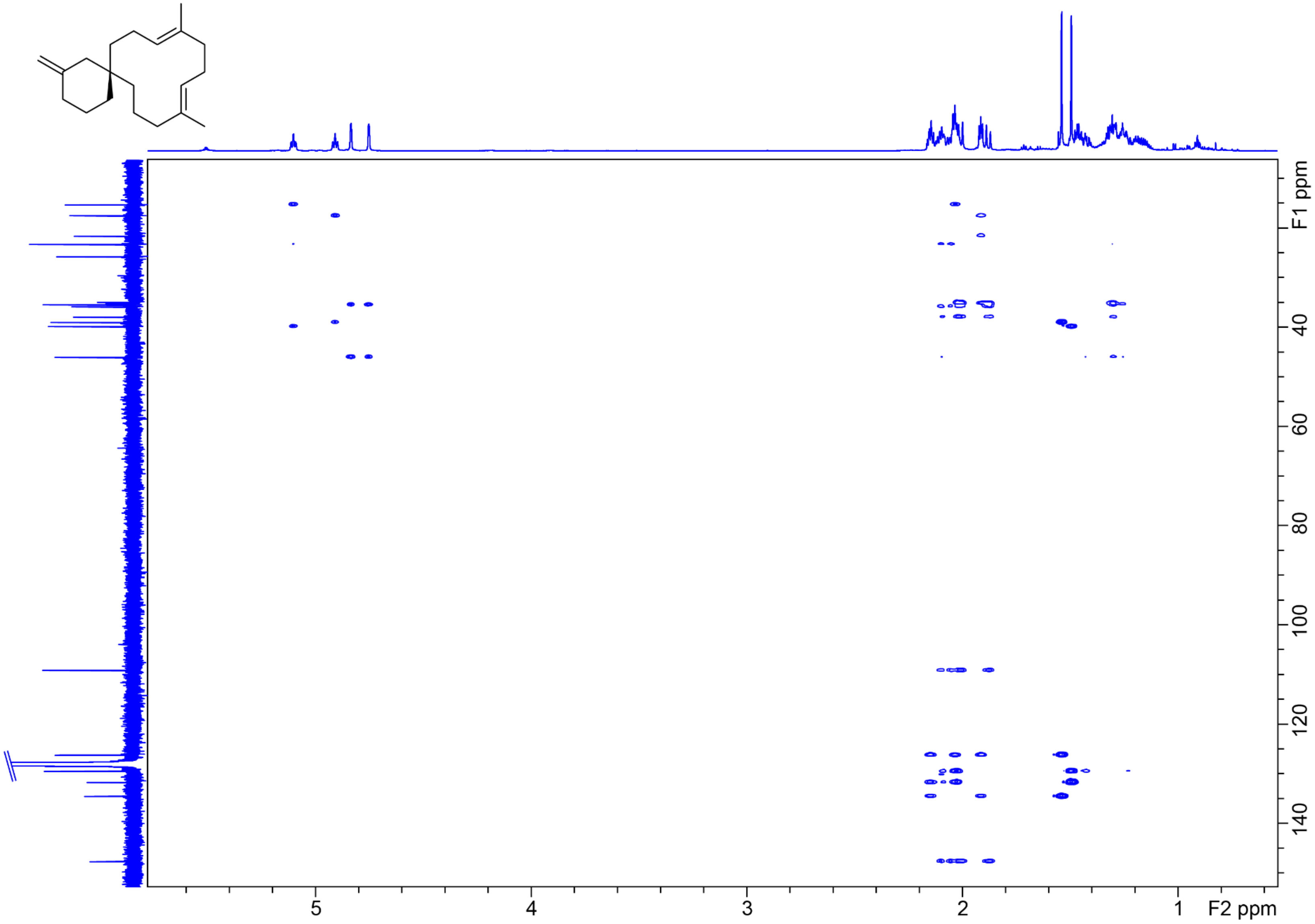

Figure S112. HMBC spectrum (C_6D_6) of **55**.

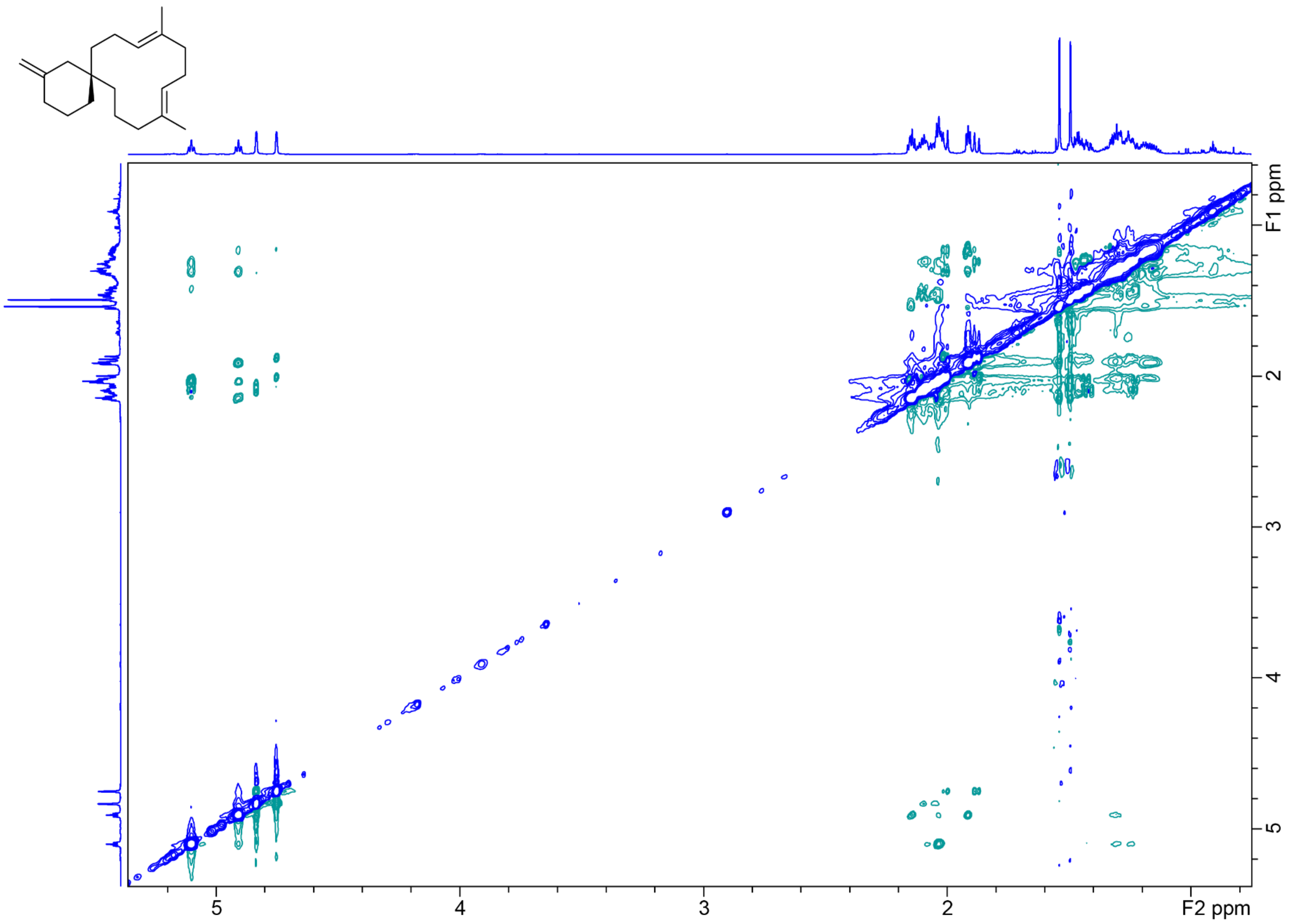

Figure S113. NOESY spectrum (C_6D_6) of **55**.

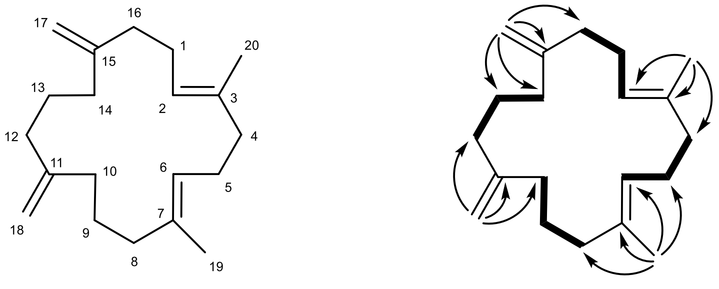

56

Figure S114. Structure elucidation of isobucketwheelene (**56**). Bold: ^{1}H,^{1}H-COSY, and single headed arrows: key HMBC correlations.

Table S17. NMR data of isobucketwheelene (**56**) in C_6D_6 recorded at 298 K.

C[a]	type	^{13}C[b]	^{1}H[b]
1	CH_2	25.33	2.19 (m, 2H)
2	CH	125.87	5.22 (tq, *J* = 6.6, 1.0)
3	C_q	133.48	–
4	CH_2	39.46	2.08 (m, 2H)
5	CH_2	25.04	2.12 (m, 2H)
6	CH	124.56	5.08 (tq, *J* = 6.6, 1.3)
7	C_q	134.73	–
8	CH_2	39.48	1.95 (m, 2H)
9	CH_2	26.79	1.50 (m, 2H)
10	CH_2	35.38	2.01 (m, 2H)
11	C_q	148.49	–
12	CH_2	36.00	2.06 (m, 2H)
13	CH_2	25.79	1.56 (m, 2H)
14	CH_2	35.38	2.01 (m, 2H)
15	C_q	149.98	–
16	CH_2	35.73	2.03 (m, 2H)
17	CH_2	110.08	4.87 (m, 2H)
18	CH_2	110.15	4.87 (m, 2H)
19	CH_3	16.19	1.52 (br s)
20	CH_3	15.49	1.50 (br s)

[a] Carbon numbering as shown in Figure S114. [b] Chemical shifts δ in ppm, multiplicity: s = singlet, t = triplet, q = quartet, m = multiplet, br = broad, coupling constants *J* are given in Hertz.

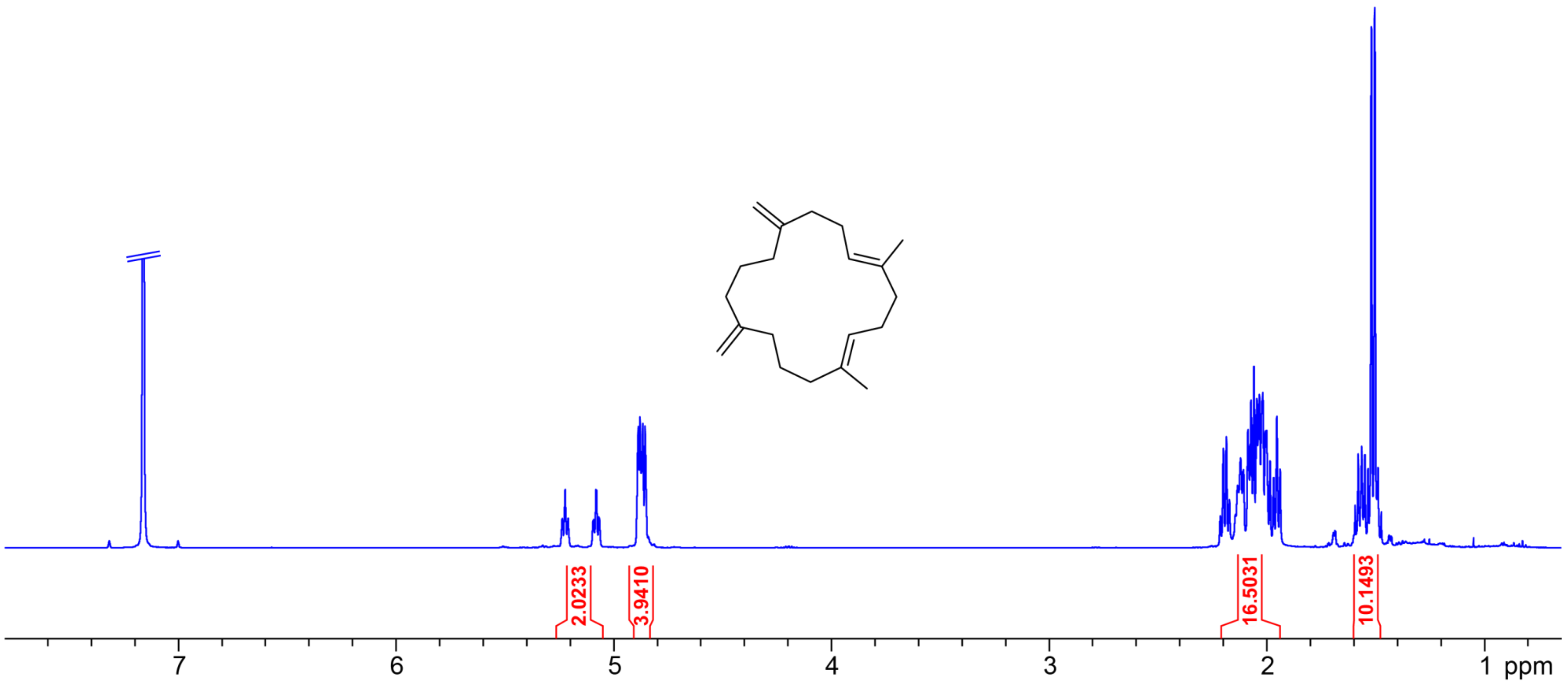

Figure S115. ^{1}H-NMR spectrum of **56** (500 MHz, C_6D_6).

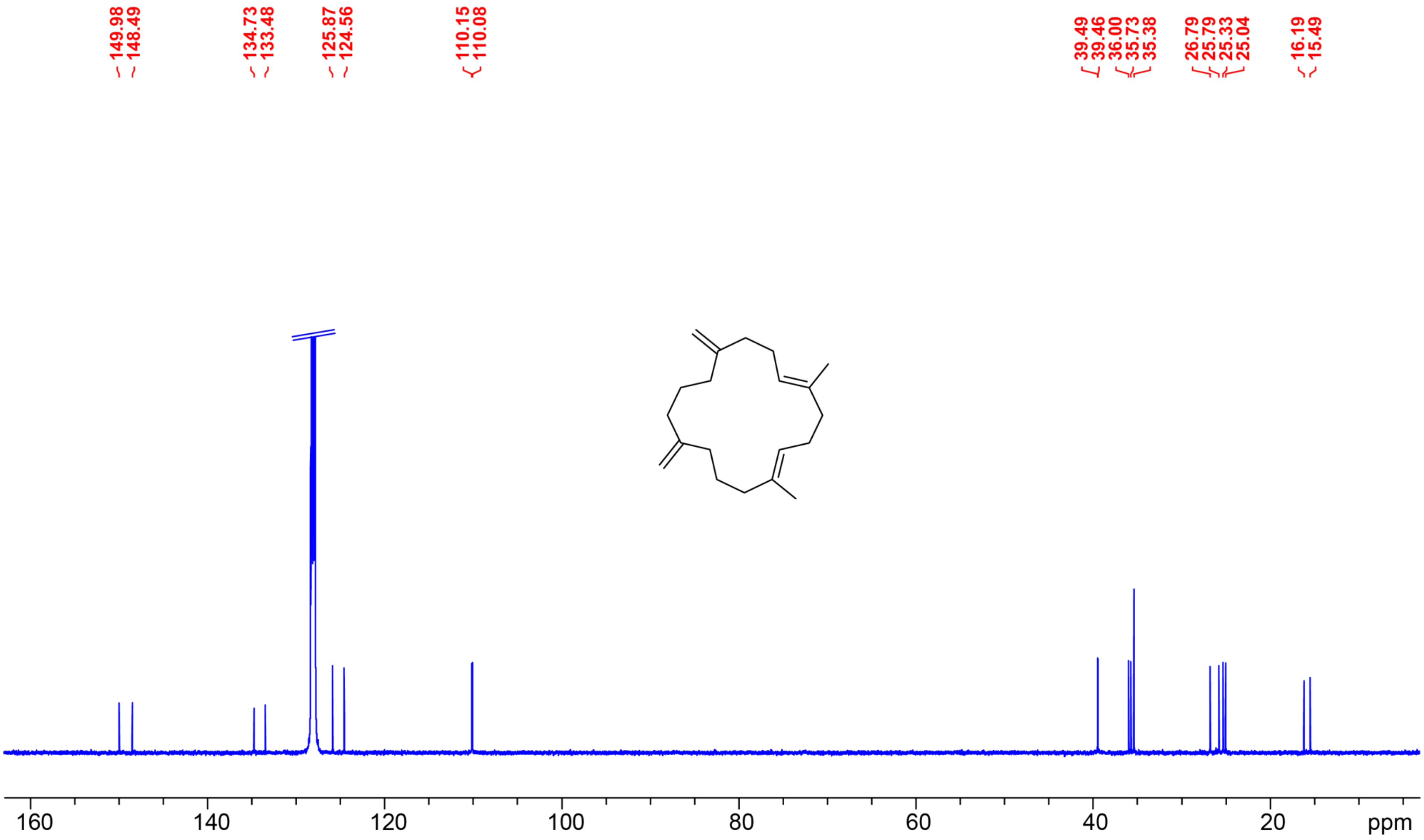

Figure S116. ^{13}C-NMR spectrum of **56** (125 MHz, C_6D_6).

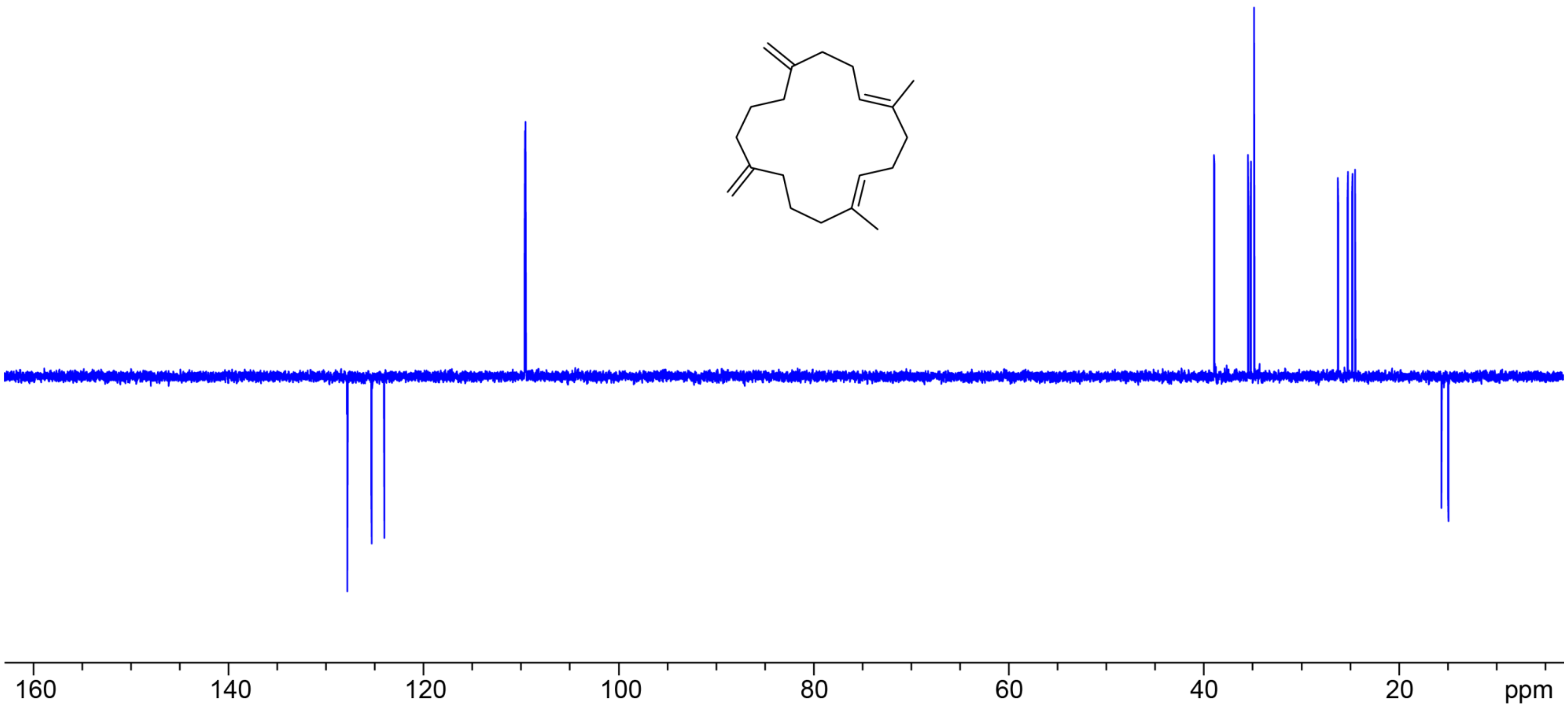

Figure S117. ^{13}C-DEPT135 spectrum of **56** (125 MHz, C_6D_6).

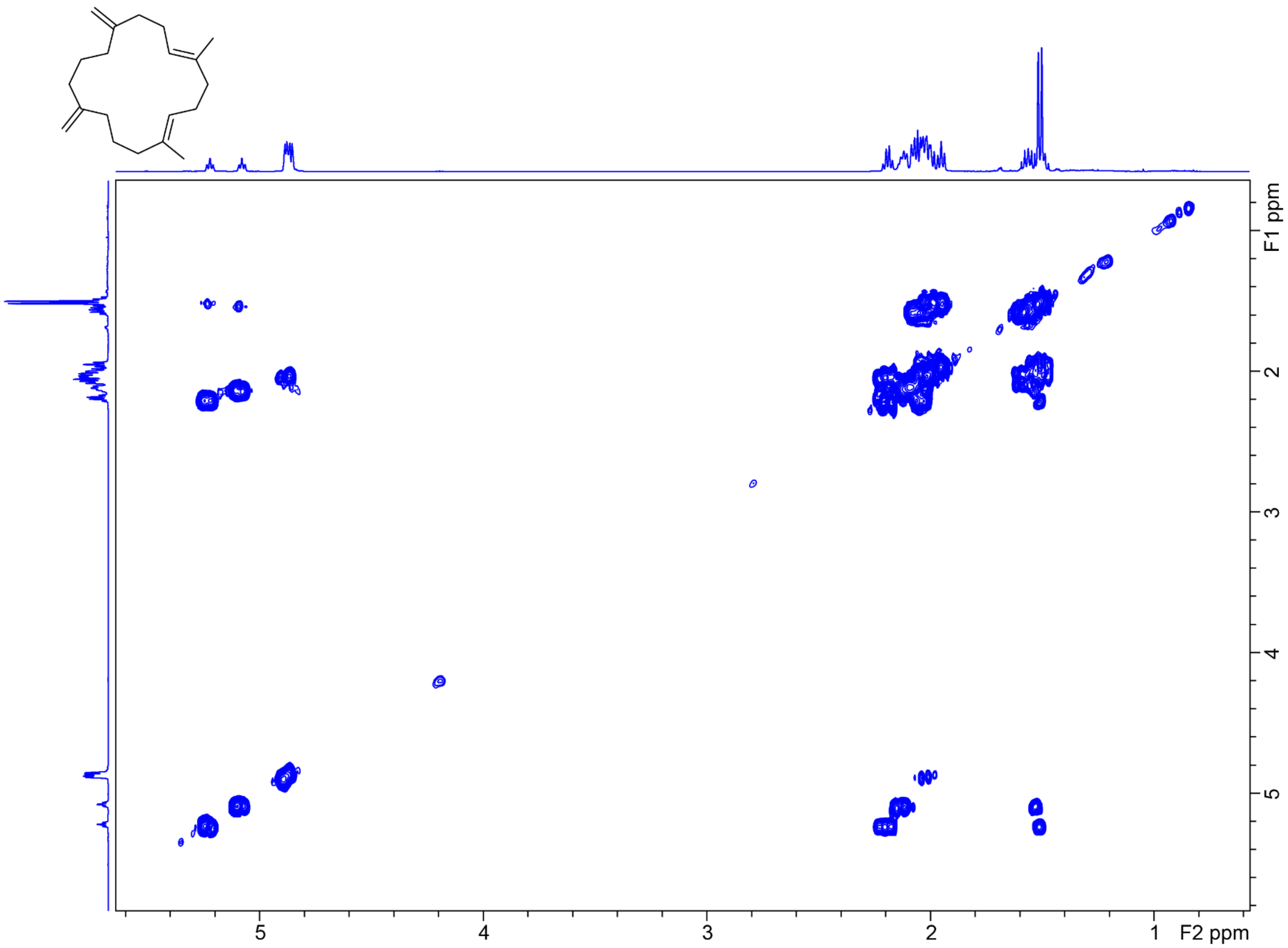

Figure S118. ^{1}H-^{1}H-COSY spectrum (C_6D_6) of **56**.

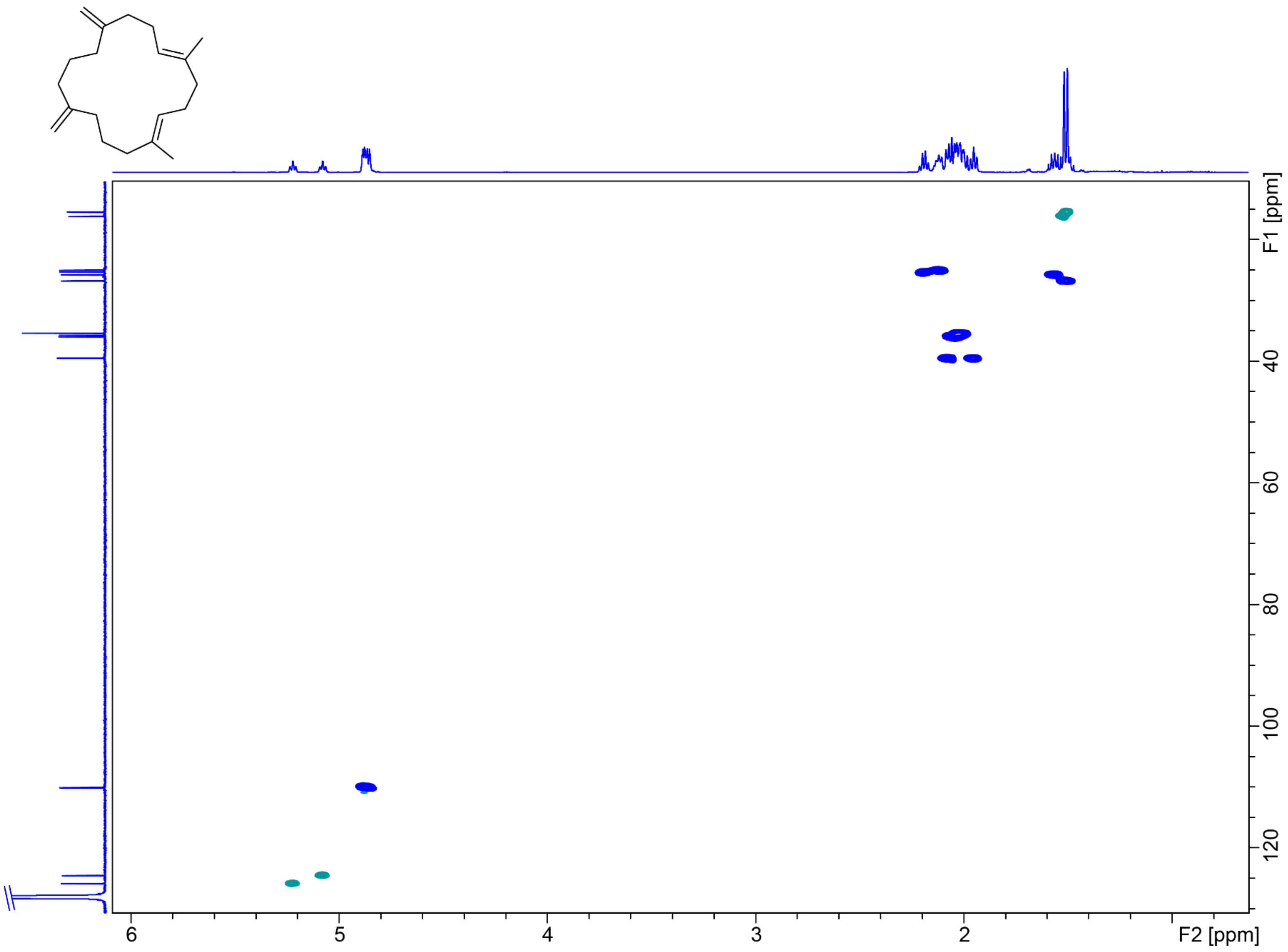

Figure S119. HSQC spectrum (C_6D_6) of **56**.

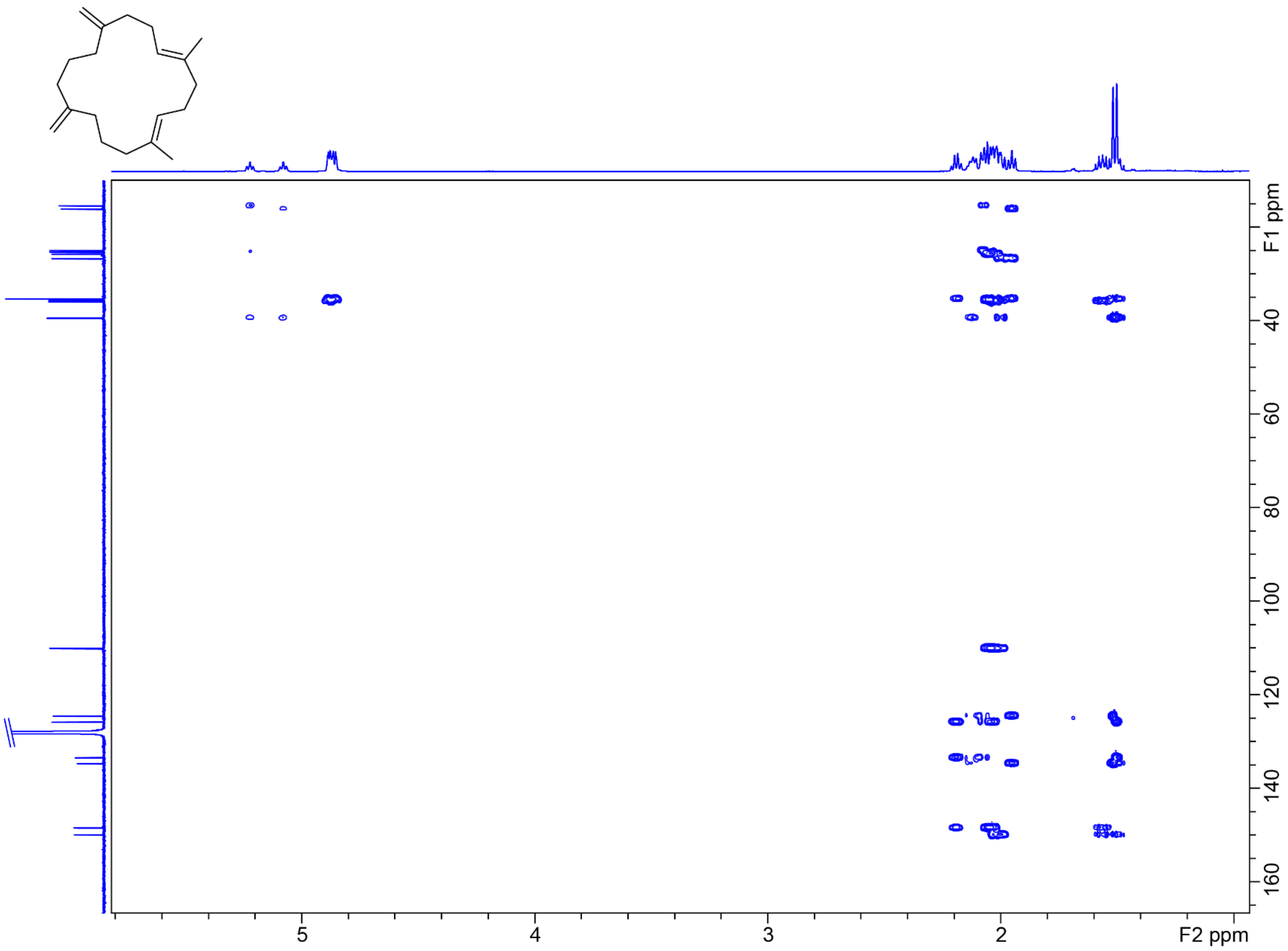

Figure S120. HMBC spectrum (C_6D_6) of **56**.

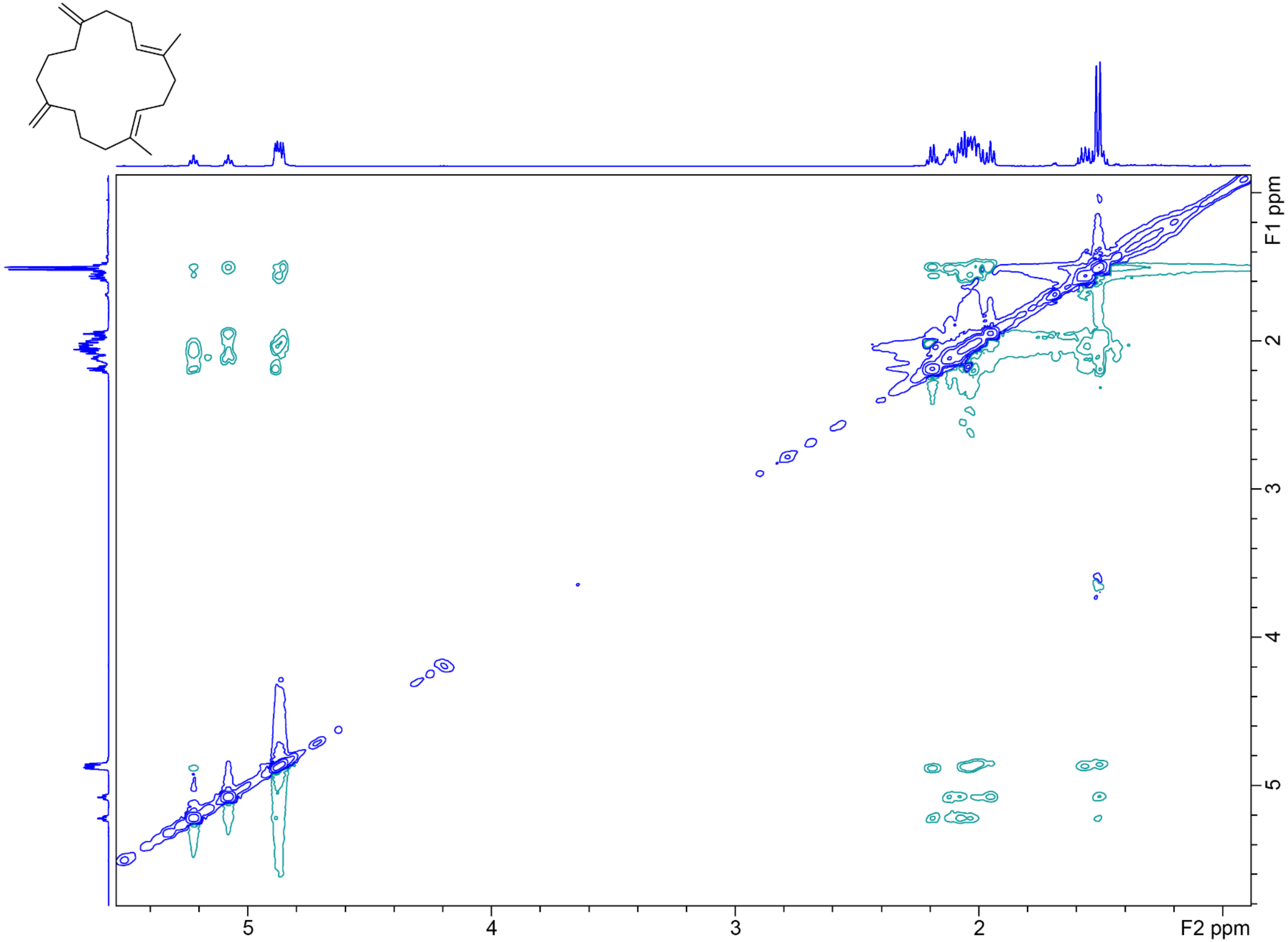

Figure S121. NOESY spectrum (C_6D_6) of **56**.

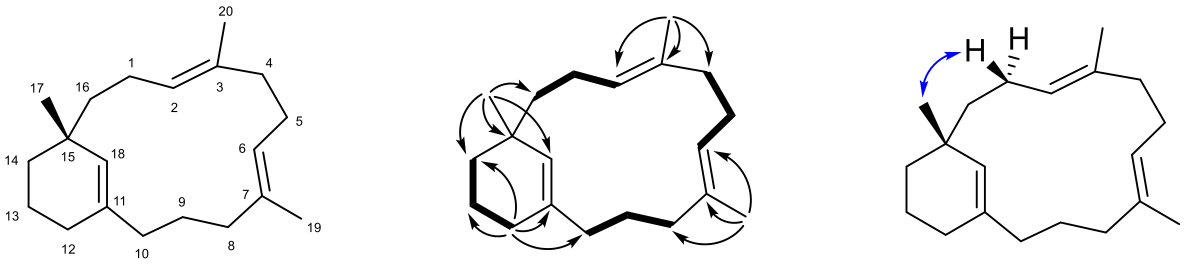

57

Figure S122. Structure elucidation of iakyroxenene (**57**). Bold: $^{1}H,^{1}H$-COSY, single headed arrows: key HMBC, and blue double headed arrows: NOESY correlations.

Table S18. NMR data of iakyroxenene (**57**) in C_6D_6 recorded at 298 K.

C[a]	type	^{13}C[b]	^{1}H[b]
1	CH_2	23.82	2.35 (m, H_β)
			2.10 (m, H_α)
2	CH	130.13	5.35 (ddqdd, J = 9.4, 5.3, 1.3, 1.3, 1.3)
3	C_q	131.45	–
4	CH_2	39.72	2.13 (m, 2H)
5	CH_2	24.33	2.19 (m, 2H)
6	CH	125.74	5.28 (ddqdd, J = 6.1, 6.1, 1.3, 1.3, 1.3)
7	C_q	132.29	–
8	CH_2	36.82	2.04 (m, 2H)
9	CH_2	22.55	1.52 (m, 2H)
10	CH_2	35.60	1.99 (m, 2H)
11	C_q	133.46	–
12	CH_2	27.80	1.86 (m)
			1.71 (m)
13	CH_2	20.21	1.64 (m, 2H)
14	CH_2	39.09	1.37 (m, 2H)
15	C_q	35.47	–
16	CH_2	43.97	1.46 (m, 2H)
17	CH_3	26.59	1.03 (s)
18	CH	132.21	5.58 (br s)
19	CH_3	15.37	1.54 (br s)
20	CH_3	15.03	1.58 (dd, J = 1.2, 1.2)

[a] Carbon numbering as shown in Figure S122. [b] Chemical shifts δ in ppm, multiplicity: s = singlet, d = doublet, q = quartet, m = multiplet, br = broad, coupling constants J are given in Hertz.

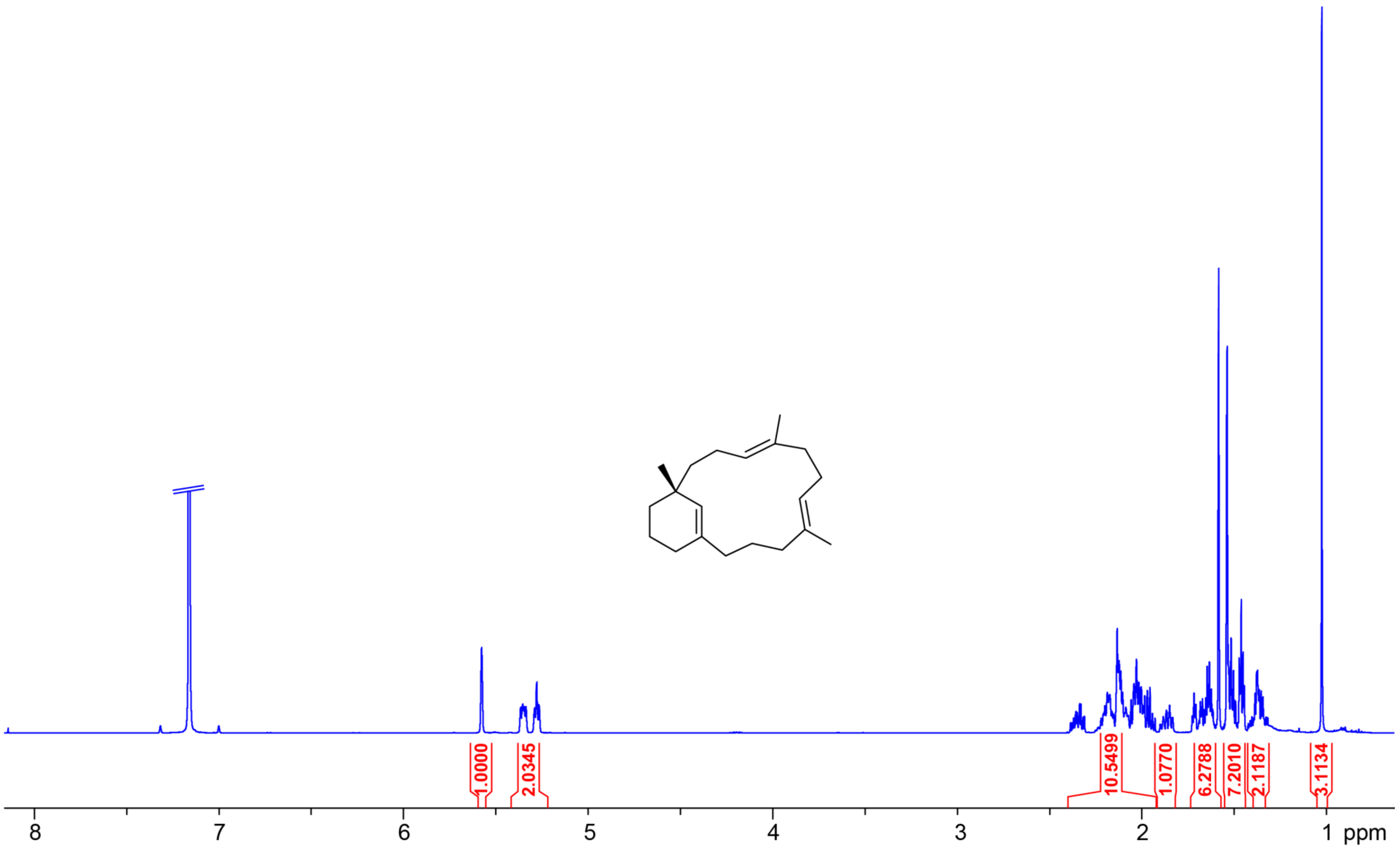

Figure S123. ^{1}H-NMR spectrum of **57** (500 MHz, C_6D_6).

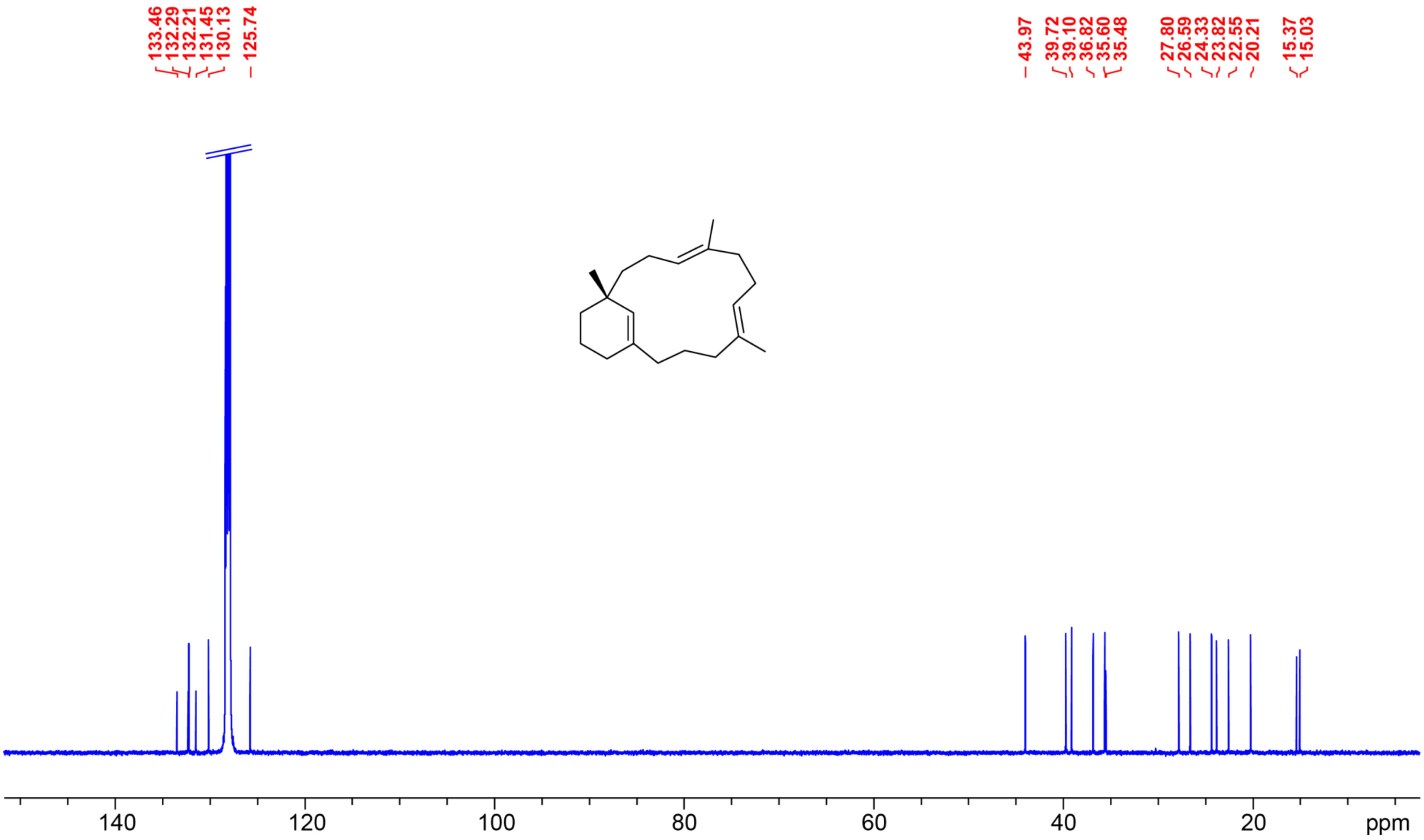

Figure S124. ^{13}C-NMR spectrum of **57** (125 MHz, C_6D_6).

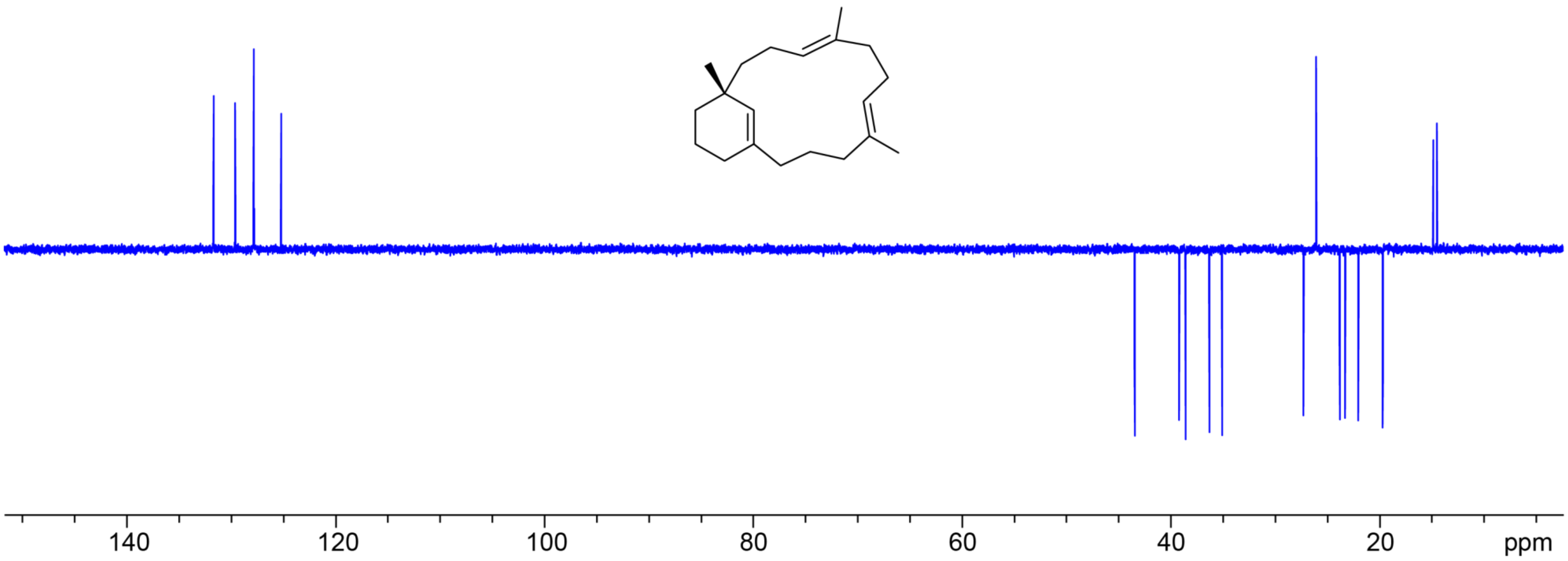

Figure S125. ^{13}C-DEPT135 spectrum of **57** (125 MHz, C_6D_6).

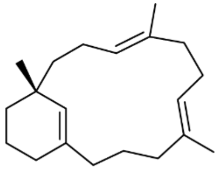

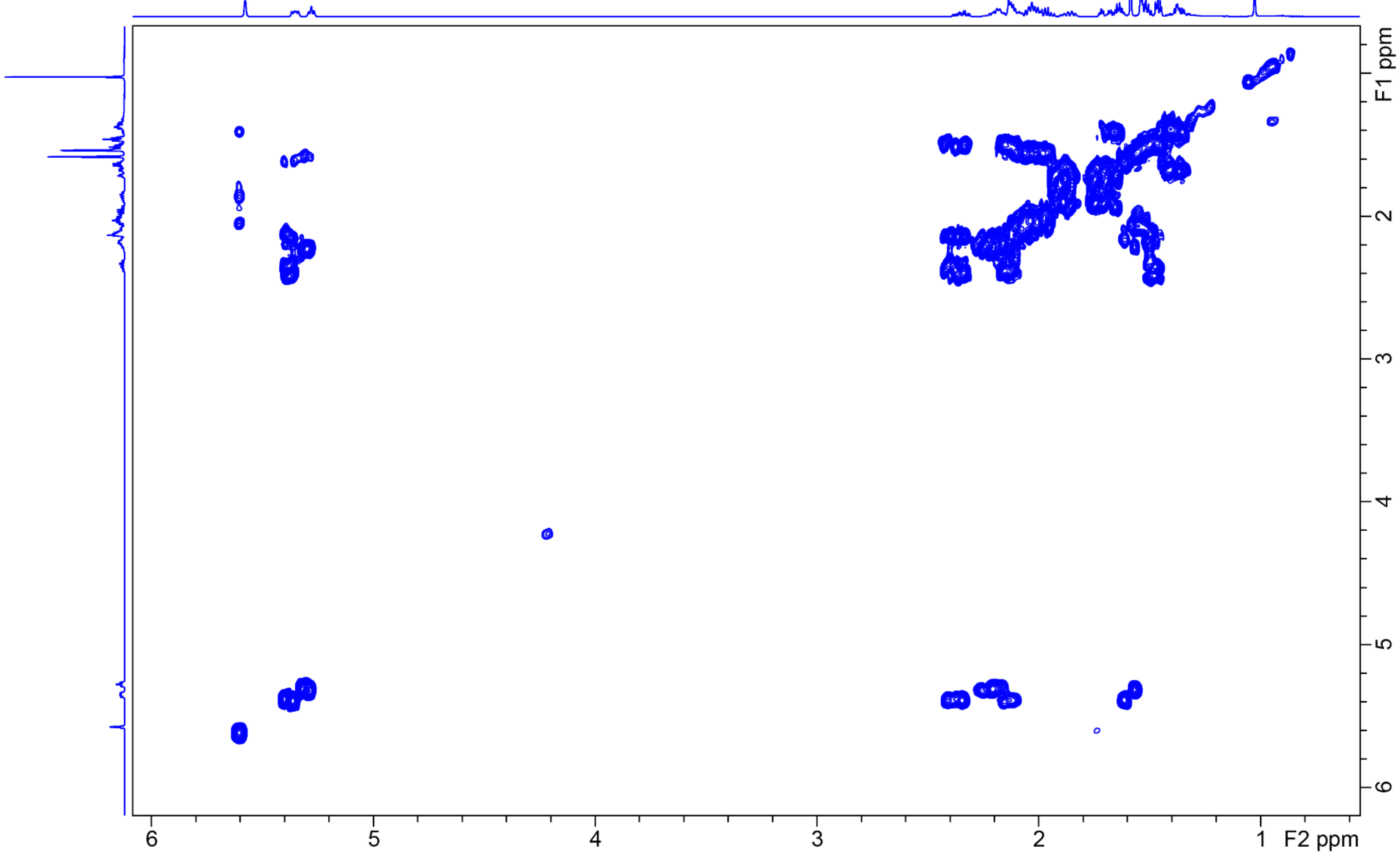

Figure S126. ^1H-^1H-COSY spectrum (C_6D_6) of **57**.

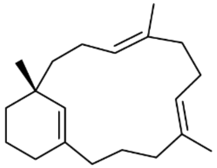

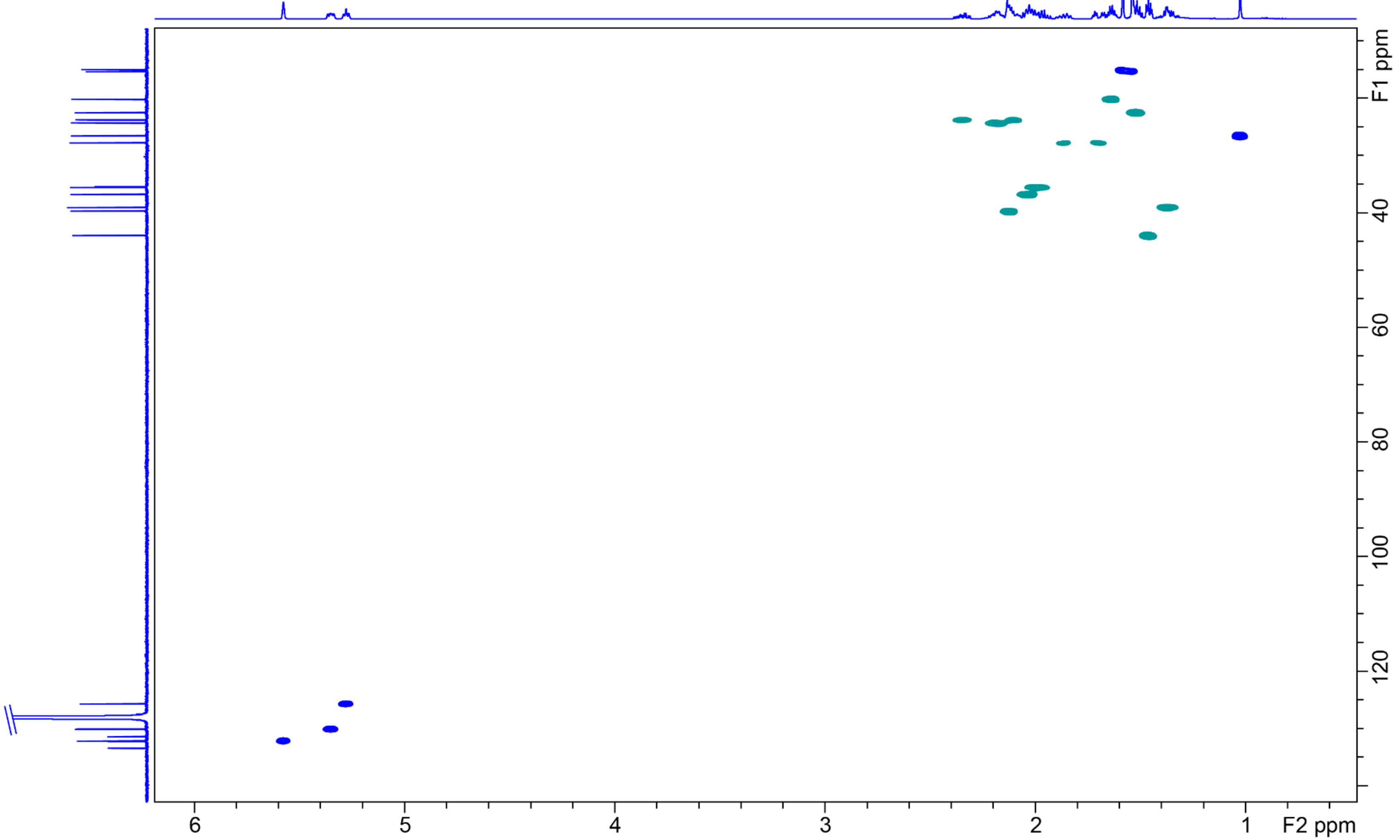

Figure S127. HSQC spectrum (C_6D_6) of **57**.

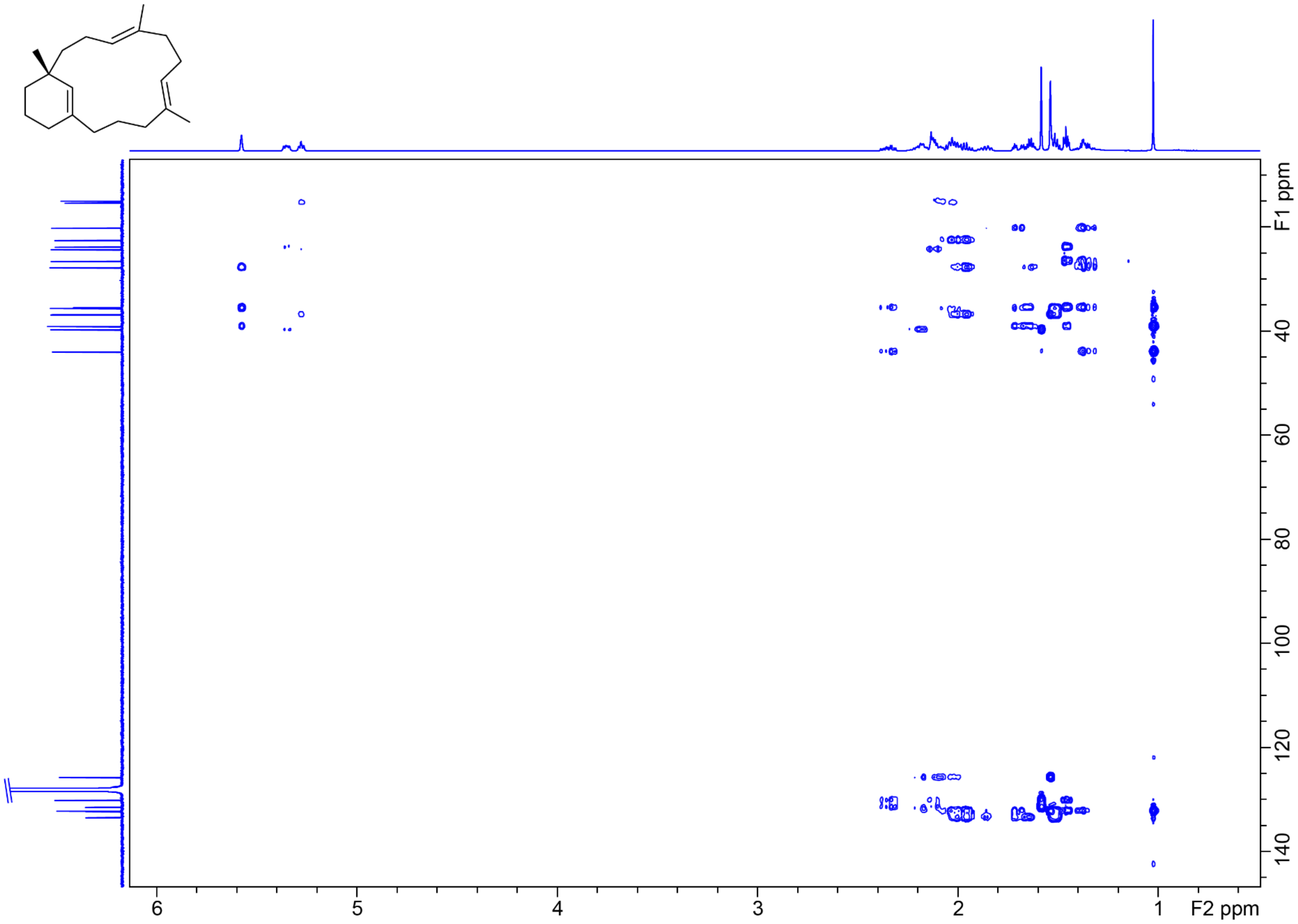

Figure S128. HMBC spectrum (C_6D_6) of **57**.

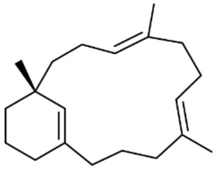

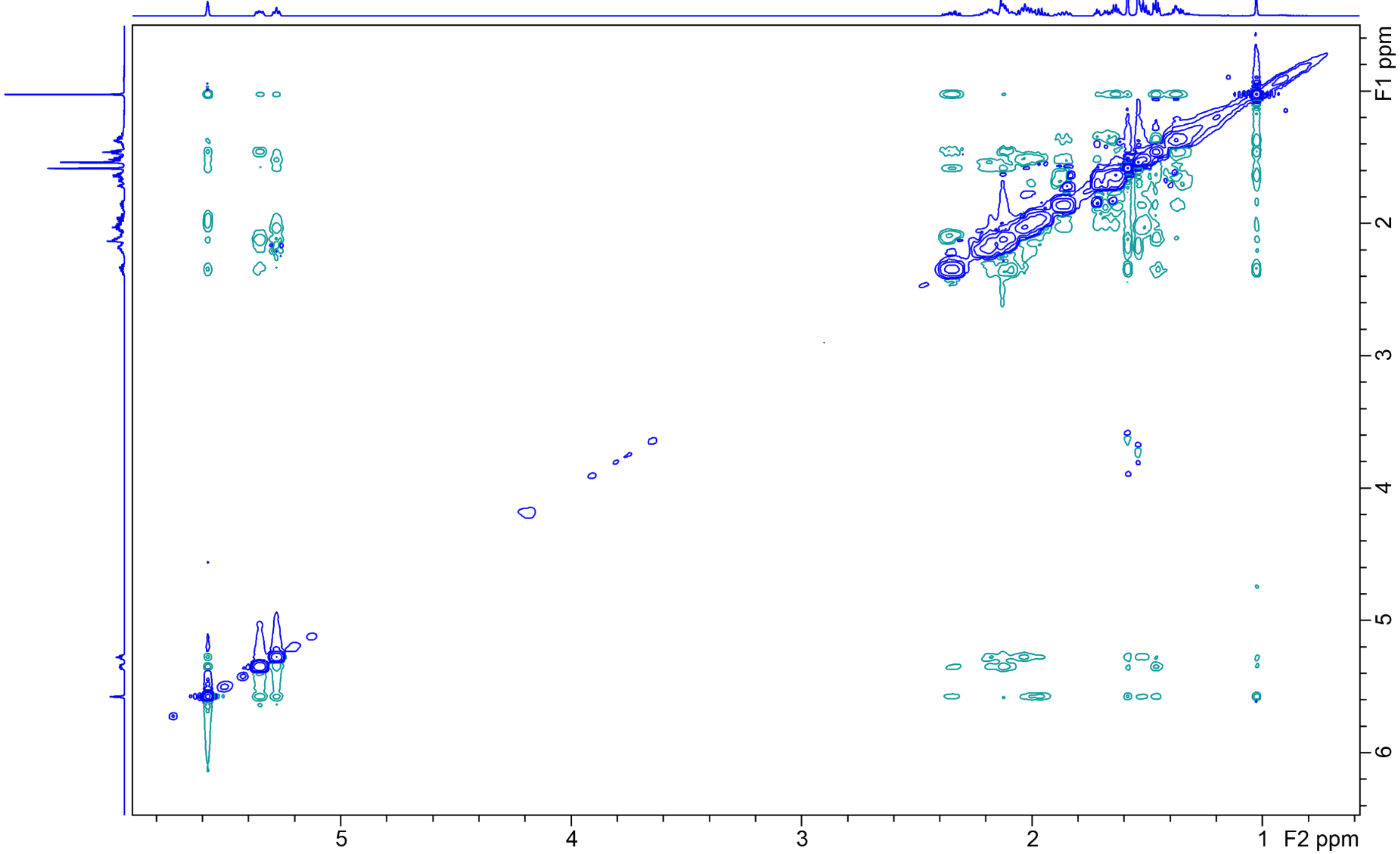

Figure S129. NOESY spectrum (C_6D_6) of **57**.

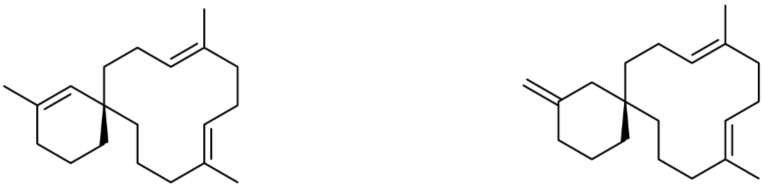

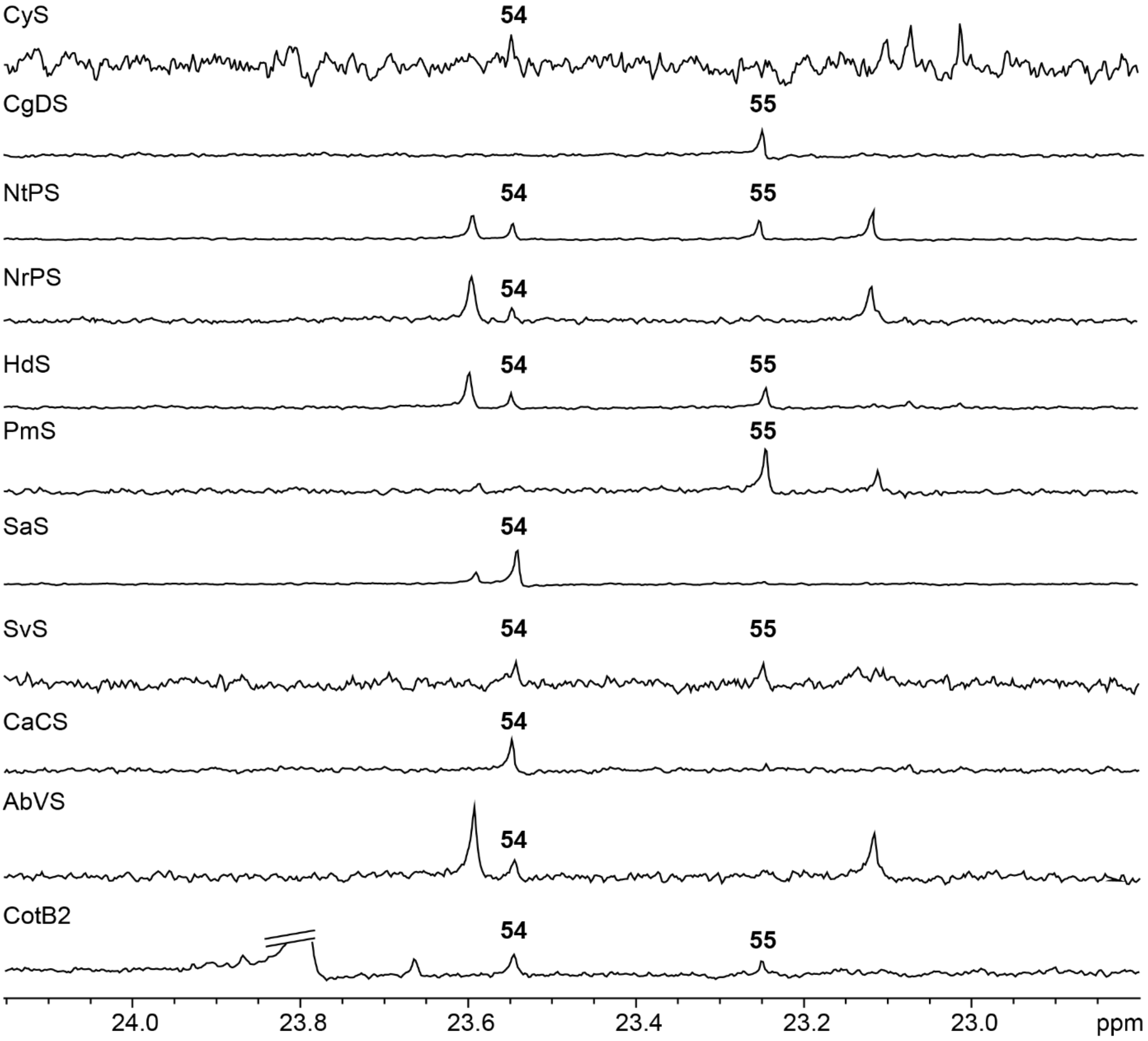

Figure S130. The production of **54** and **55** by diverse diterpene synthases. For each enzyme *iso*-FPP III and (1-^{13}C)IPP were converted using GGPPS and the diterpene synthase as mentioned at each ^{13}C-NMR spectrum. The production of **54** and **55** was indicated by the ^{13}C-NMR signals for C1 at 23.58 ppm and 23.27 ppm, respectively.

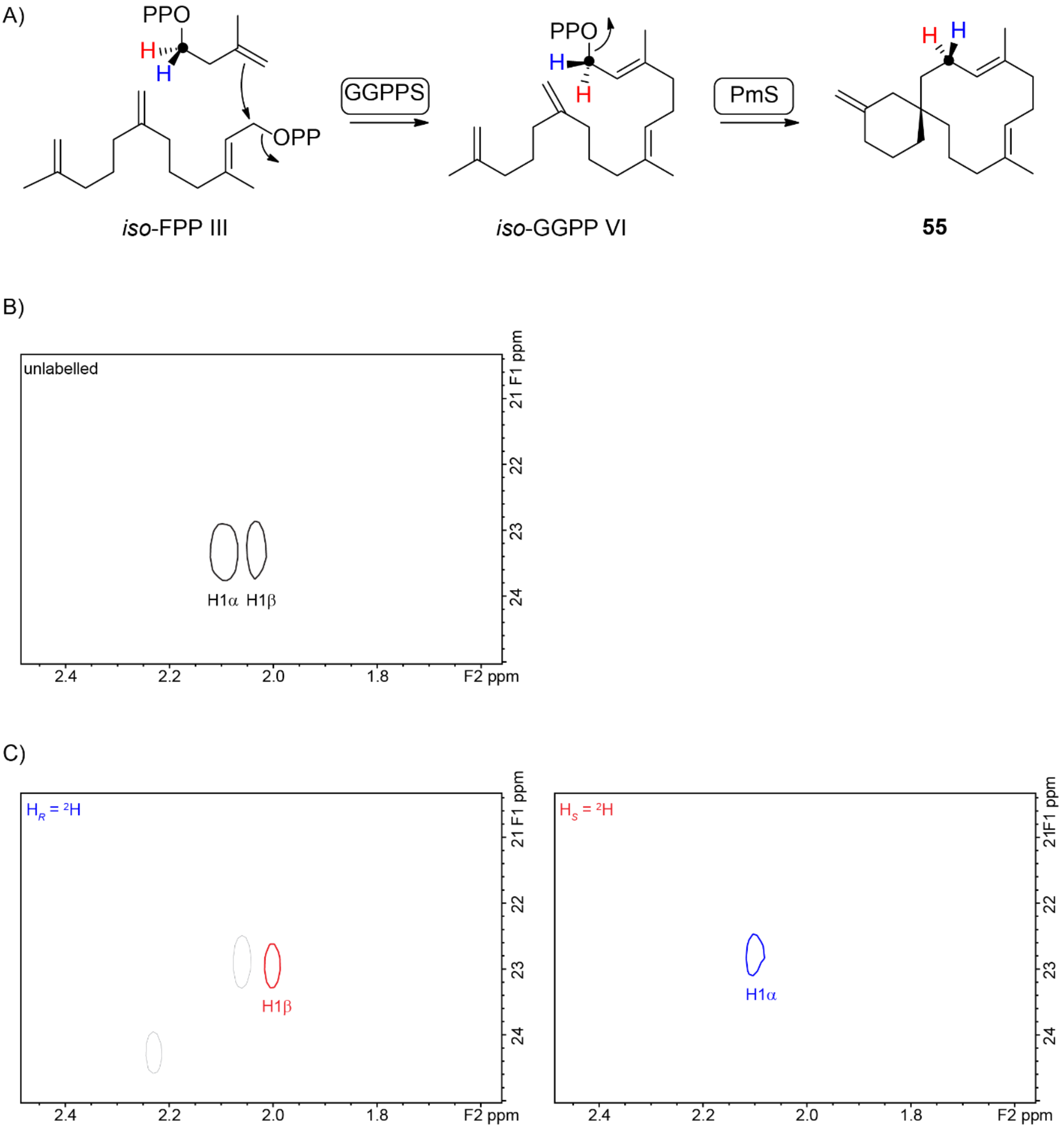

Figure S131. The absolute configuration of **55**. A) Cyclisation of labelled *iso*-GGPP VI obtained from *iso*-FPP III and (*R*)-(1-^{13}C,1-^{2}H)IPP (blue H = ^{2}H) or (*S*)-(1-^{13}C,1-^{2}H)IPP (red H = ^{2}H) with GGPPS and PmS. B) Partial HSQC spectrum of unlabelled **55** showing the region for C1, and C) HSQC spectra of labelled **55** obtained from (*R*)-(1-^{13}C,1-^{2}H)IPP (left) and from (*S*)-(1-^{13}C,1-^{2}H)IPP (right). Together with the NOESY based assignments for the hydrogens at C1 (Table S16) these data point to the shown absolute configuration of **55**.

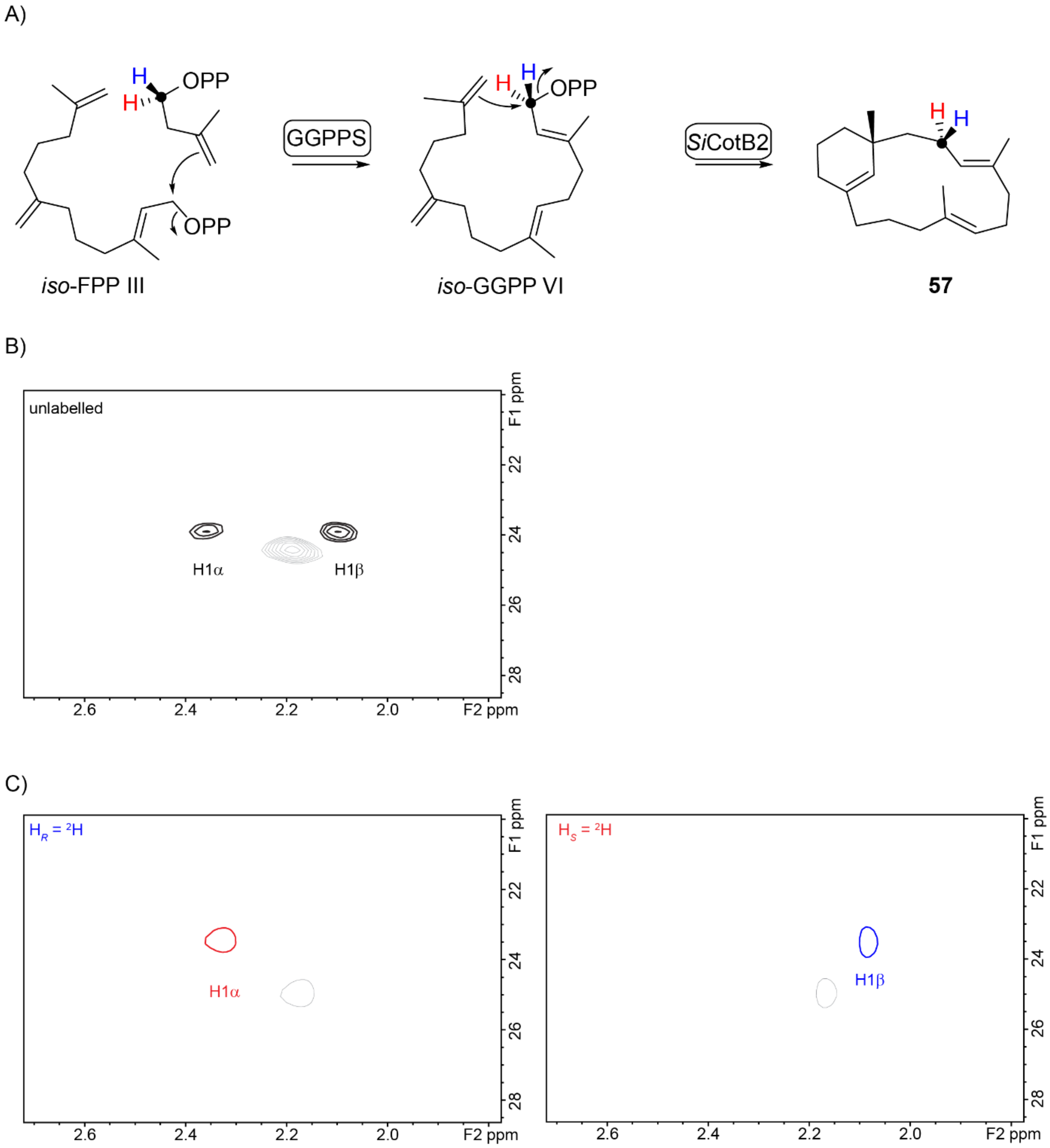

Figure S132. The absolute configuration of **57**. A) Cyclisation of labelled *iso*-GGPP VI obtained from *iso*-FPP III and (*R*)-(1-^{13}C,1-^{2}H)IPP (blue H = ^{2}H) or (*S*)-(1-^{13}C,1-^{2}H)IPP (red H = ^{2}H) with GGPPS and CotB2. B) Partial HSQC spectrum of unlabelled **57** showing the region for C1, and C) HSQC spectra of labelled **57** obtained from (*R*)-(1-^{13}C,1-^{2}H)IPP (left) and from (*S*)-(1-^{13}C,1-^{2}H)IPP (right). Together with the NOESY based assignments for the hydrogens at C1 (Table S18) these data point to the shown absolute configuration of **57**.

Cartesian coordinates of computed structures (bromination products, Table S5)

Gibbs energies (G"..." in Hartree) and imagininary frequencies of TS (T-"..." in cm^{-1}), mPW1PW91/6-311+G(d,p)//B97D3/6-31G(d,p)-sp-density-fitting, 1 bar, 298.15 K

**E**	G3355.442271		
C	-0.576773	-2.088835	-0.681376
C	-1.559439	-1.483936	0.348953
C	-1.663520	0.064704	0.282086
C	0.661181	-1.232300	-1.028553
C	-0.337294	0.799352	0.386734
C	1.343844	-0.537374	0.133744
C	2.388909	0.513183	-0.225803
H	-1.096817	-2.288544	-1.628023
H	-1.178572	-1.727549	1.355227
H	-2.263634	0.374591	1.154463
H	0.430074	-0.497123	-1.811430
C	0.311179	1.205803	1.553108
C	1.459483	2.179244	1.421311
H	1.670564	2.662652	2.383796
C	2.747125	1.460819	0.918534
H	3.478677	2.202981	0.577037
C	1.651245	-1.421513	1.320040
H	0.752230	-1.878272	1.740488
H	2.284135	-2.244160	0.954146
H	2.199931	-0.914946	2.117794
H	2.077026	1.068298	-1.120041
H	3.222426	0.905451	1.737528
H	1.183136	2.960875	0.698721
C	-0.084429	0.746761	2.916896
H	-0.588242	1.592623	3.417324
H	-0.773993	-0.102163	2.916585
H	0.794421	0.515302	3.534508
H	-0.254028	-3.070075	-0.305630
H	1.439790	-1.889595	-1.457968
C	-2.420581	0.543075	-0.995911
H	-1.863305	0.165499	-1.872863
C	-2.962927	-2.147664	0.245370
H	-2.789954	-3.228546	0.104999
C	-3.740265	-1.630706	-0.986262
C	-3.824222	-0.098167	-1.028128
H	-0.080552	1.382922	-0.502884
H	-4.417766	0.269046	-0.176724
H	-4.346665	0.231507	-1.937233
H	-4.751395	-2.063081	-0.984525
H	-3.257988	-1.986509	-1.911167
C	-3.766805	-1.984306	1.548571
H	-3.985661	-0.932284	1.779568
H	-4.731543	-2.502983	1.473394
H	-3.225628	-2.409581	2.406023
C	-2.459717	2.063738	-1.098857

C	-1.864216	2.697264	-2.127084
H	-1.892286	3.783420	-2.223401
H	-1.365298	2.146592	-2.927481
C	-3.181339	2.828520	-0.011182
H	-2.780499	2.597540	0.989663
H	-3.102408	3.911229	-0.162626
H	-4.249952	2.569919	0.017907
Br	4.021004	-0.470600	-0.791152

E-F-TS G3355.444553, T-230

C	-0.590093	-2.130640	-0.630893
C	-1.572568	-1.490642	0.379539
C	-1.637384	0.056191	0.282189
C	0.647132	-1.281997	-0.996961
C	-0.287527	0.764417	0.370798
C	1.292378	-0.519988	0.150999
C	2.365242	0.495140	-0.245993
H	-1.109923	-2.357835	-1.571487
H	-1.207169	-1.725042	1.393829
H	-2.233551	0.402017	1.143092
H	0.421114	-0.583259	-1.814342
C	0.311715	1.260353	1.537297
C	1.461739	2.219482	1.375690
H	1.693217	2.720849	2.323888
C	2.737560	1.475335	0.866610
H	3.465118	2.207654	0.496667
C	1.606314	-1.369583	1.367451
H	0.711166	-1.824785	1.797299
H	2.255616	-2.191441	1.032846
H	2.139899	-0.830996	2.155070
H	2.064609	1.025182	-1.159331
H	3.221368	0.942861	1.695550
H	1.181452	2.985598	0.637606
C	-0.113716	0.867319	2.908786
H	-0.636519	1.734941	3.350699
H	-0.796741	0.013643	2.933878
H	0.751885	0.680876	3.559807
H	-0.268918	-3.100408	-0.225480
H	1.441041	-1.947562	-1.380535
C	-2.376543	0.527452	-1.009514
H	-1.825393	0.118409	-1.875911
C	-2.988767	-2.126107	0.275547
H	-2.837869	-3.212548	0.154271
C	-3.746089	-1.613047	-0.969930
C	-3.795349	-0.079931	-1.037180
H	-0.046240	1.342560	-0.527610
H	-4.383837	0.315268	-0.194802
H	-4.305445	0.246836	-1.954334
H	-4.766570	-2.022852	-0.968796
H	-3.264976	-1.994078	-1.885354
C	-3.798978	-1.924202	1.569589

H	-3.995139	-0.863852	1.782588
H	-4.774881	-2.421658	1.494908
H	-3.274317	-2.348263	2.437813
C	-2.378257	2.046036	-1.145363
C	-1.774270	2.642493	-2.190977
H	-1.777659	3.726621	-2.311574
H	-1.294765	2.062352	-2.982294
C	-3.072004	2.852321	-0.069889
H	-2.662581	2.639718	0.931907
H	-2.975540	3.929172	-0.250243
H	-4.144503	2.613883	-0.019678
Br	3.985510	-0.523037	-0.769335

F G3355.451037

C	-0.648921	-2.239325	-0.484719
C	-1.625523	-1.499094	0.466613
C	-1.566440	0.033239	0.287289
C	0.596799	-1.424119	-0.896431
C	-0.134914	0.630948	0.346333
C	1.116465	-0.468290	0.193007
C	2.288970	0.421381	-0.292493
H	-1.171310	-2.536312	-1.404501
H	-1.307916	-1.709224	1.501944
H	-2.153056	0.487346	1.101574
H	0.394570	-0.834525	-1.803313
C	0.302135	1.400487	1.484963
C	1.449232	2.311624	1.241447
H	1.729683	2.886702	2.131962
C	2.695753	1.490323	0.725225
H	3.410054	2.194038	0.281631
C	1.466512	-1.229888	1.484808
H	0.589090	-1.693010	1.943303
H	2.173630	-2.031043	1.240041
H	1.942965	-0.598037	2.243500
H	2.024400	0.879155	-1.256361
H	3.195261	1.027635	1.585577
H	1.156043	3.010738	0.440283
C	-0.245031	1.241355	2.847599
H	-0.795246	2.169738	3.093784
H	-0.928541	0.395460	2.957265
H	0.567446	1.193583	3.588384
H	-0.340567	-3.176066	-0.000067
H	1.425815	-2.104840	-1.144226
C	-2.235785	0.479318	-1.050078
H	-1.710211	-0.032590	-1.875123
C	-3.079855	-2.038615	0.350315
H	-3.004517	-3.137319	0.282149
C	-3.770744	-1.532356	-0.935798
C	-3.701126	-0.004590	-1.077206
H	0.016673	1.245124	-0.559759
H	-4.269737	0.478282	-0.267106

H	-4.166763	0.314825	-2.020438
H	-4.819197	-1.864106	-0.941487
H	-3.298958	-1.990787	-1.819936
C	-3.905153	-1.718963	1.611224
H	-4.027618	-0.637920	1.770525
H	-4.913130	-2.147286	1.532883
H	-3.433988	-2.138194	2.511782
C	-2.094721	1.978295	-1.288276
C	-1.464261	2.447436	-2.384503
H	-1.371163	3.517148	-2.577303
H	-1.065083	1.774022	-3.145441
C	-2.662635	2.915753	-0.248912
H	-2.185337	2.765648	0.736108
H	-2.525342	3.966205	-0.530037
H	-3.736882	2.741620	-0.090955
Br	3.883268	-0.673872	-0.679688

**F**	G3355.451037		
C	-0.649027	-2.239203	-0.484821
C	-1.625652	-1.498997	0.466526
C	-1.566500	0.033342	0.287224
C	0.596679	-1.423973	-0.896488
C	-0.134942	0.630948	0.346505
C	1.116443	-0.468302	0.193066
C	2.288999	0.421381	-0.292373
H	-1.171411	-2.536179	-1.404610
H	-1.308067	-1.709152	1.501856
H	-2.153187	0.487479	1.101447
H	0.394433	-0.834225	-1.803263
C	0.302124	1.400172	1.485291
C	1.449352	2.311254	1.242068
H	1.729820	2.886037	2.132769
C	2.695830	1.490147	0.725524
H	3.410069	2.193971	0.282006
C	1.466501	-1.230109	1.484739
H	0.589073	-1.693273	1.943180
H	2.173575	-2.031261	1.239838
H	1.943002	-0.598420	2.243537
H	2.024344	0.879330	-1.256135
H	3.195455	1.027286	1.585717
H	1.156172	3.010658	0.441142
C	-0.245250	1.240995	2.847856
H	-0.795081	2.169573	3.094150
H	-0.929098	0.395340	2.957285
H	0.567147	1.192737	3.588693
H	-0.340687	-3.175954	-0.000180
H	1.425668	-2.104677	-1.144423
C	-2.235757	0.479442	-1.050199
H	-1.710250	-0.032538	-1.875241
C	-3.079989	-2.038496	0.350168
H	-3.004653	-3.137194	0.281932

C	-3.770834	-1.532140	-0.935905
C	-3.701131	-0.004353	-1.077268
H	0.016716	1.245310	-0.559467
H	-4.269671	0.478492	-0.267100
H	-4.166836	0.315080	-2.020456
H	-4.819317	-1.863803	-0.941643
H	-3.299082	-1.990551	-1.820073
C	-3.905293	-1.718958	1.611103
H	-4.027750	-0.637931	1.770523
H	-4.913276	-2.147263	1.532713
H	-3.434143	-2.138293	2.511623
C	-2.094460	1.978394	-1.288416
C	-1.463376	2.447402	-2.384343
H	-1.369955	3.517095	-2.577113
H	-1.063952	1.773927	-3.145104
C	-2.662655	2.915926	-0.249285
H	-2.185813	2.765675	0.735939
H	-2.525071	3.966366	-0.530306
H	-3.736989	2.741935	-0.091794
Br	3.883212	-0.673851	-0.679808

F-G-TS G3355.451471, T-39

C	-0.515983	-2.322174	-0.497568
C	-1.510378	-1.592778	0.435166
C	-1.527790	-0.056005	0.253301
C	0.757533	-1.524847	-0.853653
C	-0.117777	0.596573	0.135386
C	1.176218	-0.448407	0.169293
C	2.349301	0.411890	-0.382347
H	-1.014370	-2.594440	-1.438490
H	-1.173957	-1.770233	1.469282
H	-2.026633	0.358850	1.143006
H	0.632185	-1.025997	-1.827100
C	0.279487	1.622522	1.075492
C	1.347903	2.540166	0.624700
H	1.548211	3.349735	1.336254
C	2.651391	1.657636	0.449969
H	3.411051	2.280527	-0.038150
C	1.488066	-1.034835	1.555196
H	0.596187	-1.433134	2.045804
H	2.199959	-1.860695	1.443853
H	1.940569	-0.303983	2.236644
H	2.129834	0.696604	-1.421818
H	3.043998	1.381964	1.436907
H	1.087623	2.961367	-0.356770
C	-0.201043	1.680146	2.468483
H	-0.978101	2.471335	2.487998
H	-0.671139	0.755111	2.814809
H	0.582722	2.020869	3.158401
H	-0.243967	-3.272130	-0.016788
H	1.614117	-2.207993	-0.961912

C	-2.355023	0.430030	-0.984302
H	-1.829457	0.075192	-1.889845
C	-2.933674	-2.216758	0.350232
H	-2.793216	-3.307910	0.269057
C	-3.678683	-1.739805	-0.913237
C	-3.760247	-0.211398	-0.985532
H	-0.008581	1.009247	-0.876886
H	-4.345946	0.171308	-0.136382
H	-4.287724	0.106617	-1.896280
H	-4.690761	-2.169986	-0.925827
H	-3.171554	-2.117966	-1.815809
C	-3.744455	-1.959992	1.634716
H	-3.894319	-0.888640	1.830498
H	-4.740519	-2.416963	1.563927
H	-3.240974	-2.390403	2.511817
C	-2.414990	1.953548	-1.038387
C	-1.756552	2.644760	-1.989368
H	-1.802868	3.733653	-2.041289
H	-1.195296	2.139761	-2.778715
C	-3.256125	2.665555	0.000806
H	-3.084043	2.282248	1.019109
H	-3.065529	3.745620	0.001402
H	-4.328010	2.519434	-0.197881
Br	4.003927	-0.653541	-0.491397

**G**	G3355.486467		
C	-0.527761	-2.173910	-0.960436
C	-1.495087	-1.698611	0.131271
C	-1.447739	-0.142599	0.195772
C	0.926605	-1.859790	-0.544383
C	-0.095329	0.436278	-0.266675
C	1.133826	-0.454140	0.143189
C	2.376017	0.237591	-0.488733
H	-0.772265	-1.709356	-1.930763
H	-1.051569	-2.011214	1.092126
H	-1.669192	0.161790	1.229419
H	1.578134	-1.935892	-1.425710
C	0.012272	1.953922	0.024593
C	1.312457	2.513603	-0.598681
H	1.428453	3.577178	-0.344219
C	2.543946	1.713001	-0.127697
H	3.440234	2.109257	-0.623161
C	1.296199	-0.612420	1.673548
H	0.345384	-0.844712	2.169106
H	1.992995	-1.428242	1.892279
H	1.702964	0.285111	2.151179
H	2.304618	0.147949	-1.583798
H	2.713453	1.831992	0.951088
H	1.248564	2.448554	-1.696881
C	-0.098170	2.381316	1.500870
H	-0.205107	3.472185	1.567804

H	-0.947541	1.929806	2.026933
H	0.804075	2.113307	2.056246
H	-0.638209	-3.256790	-1.113622
H	1.269686	-2.638321	0.149893
C	-2.589646	0.527952	-0.771015
H	-2.196451	0.372204	-1.787992
C	-2.913363	-2.326785	0.107264
H	-2.783166	-3.375159	-0.205656
C	-3.831041	-1.643274	-0.923349
C	-3.961330	-0.142403	-0.643177
H	-0.099159	0.371044	-1.371246
H	-4.393121	0.014142	0.355090
H	-4.646910	0.329921	-1.362496
H	-4.825977	-2.109338	-0.899031
H	-3.436287	-1.793477	-1.941138
C	-3.540264	-2.345741	1.515533
H	-3.615510	-1.341361	1.957733
H	-4.553396	-2.769024	1.490301
H	-2.938372	-2.957200	2.200692
C	-2.441244	1.926169	-0.374571
C	-1.227452	2.625767	-0.798320
H	-1.248765	3.699620	-0.580207
H	-1.012192	2.455060	-1.860063
C	-3.381723	2.565079	0.571563
H	-3.794108	1.862071	1.306280
H	-2.963995	3.451469	1.058935
H	-4.250804	2.894640	-0.033120
Br	4.025900	-0.749807	-0.031297

H G3355.412059

C	-1.029809	-1.944473	0.746039
C	-0.674646	-0.794001	0.064803
C	-3.476535	-0.480280	-0.689595
C	-0.473009	0.626458	0.561521
C	1.023480	0.811857	0.213076
C	1.540228	-0.587585	0.504956
C	1.639596	2.024264	0.937408
H	2.505862	2.376465	0.360586
C	0.557417	3.126772	1.075228
C	-0.499990	3.097276	-0.051850
C	-1.345799	1.777500	-0.031978
C	-1.955095	1.491752	-1.427850
C	-3.363017	0.835927	-1.448446
H	-3.640623	0.679897	-2.501649
H	-2.042102	2.446321	-1.966545
H	0.040607	3.028676	2.044514
H	-1.257292	0.887648	-2.031780
H	-4.083443	1.559821	-1.036450
H	-2.174858	1.943288	0.677073
H	0.035494	3.133327	-1.019384
C	-1.412649	4.330183	0.030618

H	-0.838083	5.255509	-0.107840
H	-2.201044	4.310697	-0.733931
H	-1.903519	4.383780	1.014024
H	1.046270	4.110943	1.092262
H	2.015953	1.744066	1.931541
H	1.092962	0.968886	-0.875790
H	-0.566334	0.649896	1.657770
H	-0.585225	-0.900149	-1.017033
C	-3.073445	-1.637187	-1.267773
C	-1.393785	-3.145524	-0.085569
H	-2.719728	-1.585796	-2.304588
H	-1.304877	-4.083477	0.478997
H	-0.744382	-3.212042	-0.971466
C	-4.046406	-0.387203	0.707644
H	-5.033096	0.098571	0.671493
H	-4.173722	-1.357274	1.199448
H	-3.421786	0.241540	1.360068
C	-1.258637	-2.026672	2.221116
H	-1.252208	-1.057398	2.728292
H	-2.222868	-2.518370	2.417044
H	-0.498377	-2.673605	2.690196
C	1.855920	-0.986725	1.908504
H	2.890825	-0.659172	2.108712
H	1.216810	-0.490359	2.644821
H	1.831073	-2.073307	2.048923
C	2.182369	-1.368025	-0.592760
H	2.350354	-2.423783	-0.362160
H	1.708558	-1.235341	-1.568192
C	-2.885753	-2.966187	-0.579424
H	-3.102257	-3.800132	-1.261805
H	-3.553980	-3.078655	0.284490
Br	3.974927	-0.498835	-0.755019

H-J-TS G3355.404370, T-147

C	-0.551454	-1.908315	0.606115
C	-0.874113	-0.699427	-0.044839
C	-3.284650	-0.758442	-0.518789
C	-0.732383	0.652365	0.582755
C	0.794763	0.773180	0.328404
C	1.360362	-0.660700	0.598710
C	1.391994	1.975495	1.077218
H	2.337079	2.256266	0.593010
C	0.369784	3.147453	1.061320
C	-0.640719	3.103823	-0.110340
C	-1.550480	1.838607	-0.006385
C	-2.246762	1.497348	-1.341403
C	-3.497932	0.584447	-1.219942
H	-3.883492	0.401285	-2.234071
H	-2.580106	2.429696	-1.816602
H	-0.205064	3.149714	2.002668
H	-1.522645	1.058789	-2.048917

H	-4.277993	1.142526	-0.681247
H	-2.342699	2.075953	0.725364
H	-0.075871	3.040510	-1.059234
C	-1.474613	4.394140	-0.139209
H	-0.830937	5.270749	-0.289226
H	-2.219691	4.391050	-0.945640
H	-2.010676	4.532047	0.811855
H	0.914192	4.101303	1.037692
H	1.632943	1.716578	2.117437
H	0.927506	0.958972	-0.750260
H	-0.903004	0.589792	1.667939
H	-0.655784	-0.689006	-1.112885
C	-2.721042	-1.803378	-1.211239
C	-0.724590	-3.097904	-0.318431
H	-2.459816	-1.603798	-2.257679
H	-0.433910	-4.047558	0.148072
H	-0.146979	-2.984459	-1.247272
C	-3.851352	-0.869109	0.874953
H	-4.935131	-0.683199	0.827986
H	-3.699905	-1.847172	1.340086
H	-3.437104	-0.097034	1.538615
C	-0.828201	-2.180485	2.064687
H	-0.923348	-1.278228	2.674780
H	-1.766498	-2.746222	2.154657
H	-0.046938	-2.816449	2.501136
C	1.888737	-0.943281	1.975740
H	2.867225	-0.439553	2.046670
H	1.259486	-0.536667	2.773132
H	2.062573	-2.011659	2.145295
C	2.122238	-1.300071	-0.525165
H	2.441407	-2.323809	-0.307267
H	1.603429	-1.254148	-1.486737
C	-2.269864	-3.138734	-0.684473
H	-2.418772	-3.915934	-1.446320
H	-2.836828	-3.440767	0.205436
Br	3.785401	-0.229761	-0.797903

**J**	G3355.443379		
C	-0.157829	-1.791981	0.429580
C	-1.001978	-0.752677	-0.326663
C	-3.215522	-0.804169	-0.398396
C	-0.921016	0.515975	0.511814
C	0.630155	0.596397	0.488308
C	1.189408	-0.902690	0.580979
C	1.118634	1.696146	1.428140
H	2.182609	1.882415	1.231750
C	0.271632	2.991122	1.188826
C	-0.623752	2.981746	-0.081068
C	-1.635004	1.804409	0.017901
C	-2.450634	1.530163	-1.260589
C	-3.615088	0.518729	-1.004723

H	-4.100050	0.296429	-1.971083
H	-2.923283	2.448910	-1.629690
H	-0.389257	3.165010	2.054320
H	-1.802138	1.173065	-2.075933
H	-4.363427	0.998455	-0.360091
H	-2.362174	2.098276	0.796993
H	0.013616	2.823437	-0.969392
C	-1.330018	4.336455	-0.244242
H	-0.596352	5.151968	-0.286475
H	-1.927221	4.388468	-1.164473
H	-1.999464	4.536226	0.606263
H	0.937945	3.863018	1.139990
H	1.035486	1.386961	2.480039
H	0.898780	0.940695	-0.524254
H	-1.245292	0.304680	1.541509
H	-0.500332	-0.486131	-1.267131
C	-2.241012	-1.603375	-1.070983
C	-0.296102	-3.032714	-0.493071
H	-2.172619	-1.335516	-2.131550
H	0.035937	-3.968079	-0.025202
H	0.266162	-2.912638	-1.430189
C	-3.832104	-1.160333	0.904167
H	-4.925106	-1.101831	0.762493
H	-3.572633	-2.150654	1.280108
H	-3.599754	-0.394342	1.660092
C	-0.677237	-2.192974	1.829190
H	-0.768392	-1.352734	2.523361
H	-1.644343	-2.702775	1.789129
H	0.027913	-2.903230	2.275204
C	1.977740	-1.180620	1.874222
H	2.902546	-0.591917	1.868487
H	1.424708	-0.915849	2.781055
H	2.261368	-2.239637	1.940203
C	2.105854	-1.198701	-0.623969
H	2.554567	-2.196702	-0.557042
H	1.586322	-1.106702	-1.585036
C	-1.825377	-3.068936	-0.814086
H	-2.042723	-3.669952	-1.706105
H	-2.391726	-3.510801	0.014446
Br	3.610681	0.068276	-0.762109

J G3355.443378

C	-0.157895	-1.791998	0.429471
C	-1.002081	-0.752625	-0.326768
C	-3.215653	-0.803900	-0.398400
C	-0.921001	0.515971	0.511760
C	0.630225	0.596273	0.488384
C	1.189359	-0.902897	0.580878
C	1.118564	1.695859	1.428426
H	2.182671	1.881925	1.232469
C	0.271963	2.991049	1.188827

C	-0.623458	2.981734	-0.081063
C	-1.634835	1.804509	0.017902
C	-2.450451	1.530275	-1.260659
C	-3.614990	0.519039	-1.004721
H	-4.100102	0.296765	-1.971009
H	-2.922878	2.449086	-1.629847
H	-0.388851	3.165311	2.054296
H	-1.801887	1.172994	-2.075866
H	-4.363276	0.998802	-0.360038
H	-2.362060	2.098469	0.796916
H	0.013869	2.823369	-0.969396
C	-1.329532	4.336541	-0.244169
H	-0.595717	5.151944	-0.286288
H	-1.926678	4.388817	-1.164395
H	-1.998989	4.536339	0.606327
H	0.938501	3.862731	1.139779
H	1.034946	1.386682	2.480285
H	0.899000	0.940741	-0.524095
H	-1.245252	0.304608	1.541426
H	-0.500347	-0.486159	-1.267181
C	-2.241249	-1.603314	-1.070963
C	-0.296348	-3.032680	-0.493167
H	-2.172784	-1.335614	-2.131550
H	0.035860	-3.968106	-0.025559
H	0.265578	-2.912377	-1.430465
C	-3.832086	-1.159888	0.904276
H	-4.925093	-1.101210	0.762711
H	-3.572735	-2.150271	1.280212
H	-3.599553	-0.393873	1.660102
C	-0.677372	-2.192908	1.829061
H	-0.768402	-1.352603	2.523110
H	-1.644534	-2.702580	1.788961
H	0.027718	-2.903242	2.275087
C	1.977808	-1.181074	1.874059
H	2.902809	-0.592645	1.868137
H	1.424944	-0.916024	2.780896
H	2.261085	-2.240170	1.940078
C	2.105747	-1.198755	-0.624155
H	2.554399	-2.196797	-0.557418
H	1.586217	-1.106593	-1.585192
C	-1.825786	-3.068896	-0.813826
H	-2.043339	-3.670024	-1.705710
H	-2.391935	-3.510551	0.014918
Br	3.610607	0.068182	-0.762075

J-K-TS G3355.442001, T-87

C	-0.277553	-1.738015	0.538059
C	-1.118788	-0.685671	-0.207808
C	-2.935403	-0.801284	-0.449149
C	-0.932018	0.628982	0.551678
C	0.615032	0.627713	0.479907

C	1.108492	-0.886850	0.611514
C	1.162541	1.729557	1.393768
H	2.211853	1.908475	1.122337
C	0.310376	3.027462	1.223497
C	-0.561241	3.071341	-0.058141
C	-1.587793	1.902101	-0.025907
C	-2.248960	1.578115	-1.376218
C	-3.334723	0.480398	-1.229740
H	-3.689654	0.184700	-2.227559
H	-2.735415	2.468797	-1.794427
H	-0.365153	3.142216	2.087816
H	-1.486322	1.279940	-2.114693
H	-4.193643	0.919461	-0.704106
H	-2.393483	2.206129	0.666111
H	0.092461	2.933557	-0.938295
C	-1.258223	4.432543	-0.198049
H	-0.520235	5.243734	-0.248441
H	-1.872947	4.490773	-1.106359
H	-1.911518	4.629833	0.665252
H	0.970393	3.905276	1.239127
H	1.151326	1.414970	2.446560
H	0.873944	0.938888	-0.545203
H	-1.249747	0.480542	1.593352
H	-0.680726	-0.518951	-1.198345
C	-2.371536	-1.875829	-1.173903
C	-0.366569	-3.024363	-0.325852
H	-2.157955	-1.674280	-2.230186
H	0.007620	-3.917796	0.190563
H	0.190173	-2.930733	-1.268132
C	-3.676345	-0.992996	0.858604
H	-4.749748	-0.959913	0.623904
H	-3.470403	-1.941033	1.357495
H	-3.473442	-0.170104	1.553778
C	-0.755289	-2.087716	1.965360
H	-0.858700	-1.215405	2.617500
H	-1.710240	-2.619813	1.967217
H	-0.024741	-2.757101	2.431826
C	1.927228	-1.157206	1.886477
H	2.867436	-0.595685	1.841041
H	1.408315	-0.858532	2.802672
H	2.184226	-2.222035	1.967480
C	1.979581	-1.245965	-0.611890
H	2.392564	-2.258701	-0.537787
H	1.437920	-1.148449	-1.560364
C	-1.889494	-3.203912	-0.667964
H	-2.014363	-3.956961	-1.457375
H	-2.450022	-3.536460	0.212648
Br	3.528124	-0.043879	-0.816025

K G3355.440871

C	-0.304839	-1.725840	0.545927

C	-1.139593	-0.663651	-0.192959
C	-2.903320	-0.788606	-0.444850
C	-0.925179	0.652019	0.558293
C	0.619757	0.630694	0.476911
C	1.093292	-0.889627	0.613968
C	1.184123	1.731256	1.383115
H	2.232901	1.899544	1.102575
C	0.342734	3.036359	1.215286
C	-0.533134	3.086462	-0.063242
C	-1.569664	1.925663	-0.026659
C	-2.225200	1.596239	-1.378941
C	-3.304940	0.492503	-1.237123
H	-3.651113	0.193705	-2.237018
H	-2.713310	2.484912	-1.799652
H	-0.328309	3.157635	2.082164
H	-1.457110	1.301540	-2.113199
H	-4.171093	0.927461	-0.719981
H	-2.376413	2.239078	0.659758
H	0.116578	2.941278	-0.945150
C	-1.219012	4.453000	-0.203757
H	-0.474554	5.257828	-0.261692
H	-1.838762	4.512709	-1.108536
H	-1.865561	4.658935	0.662601
H	1.010538	3.908322	1.227677
H	1.178236	1.420130	2.436927
H	0.876796	0.933568	-0.551102
H	-1.239943	0.512284	1.602253
H	-0.700566	-0.512837	-1.186794
C	-2.409942	-1.894951	-1.185187
C	-0.397464	-3.011641	-0.318515
H	-2.195994	-1.703182	-2.244094
H	-0.023640	-3.906261	0.196461
H	0.155591	-2.919122	-1.262781
C	-3.668120	-0.967629	0.857001
H	-4.738066	-0.927184	0.610728
H	-3.476041	-1.914362	1.364092
H	-3.462951	-0.144719	1.550963
C	-0.778026	-2.075437	1.974644
H	-0.880869	-1.202044	2.625528
H	-1.733141	-2.607232	1.979535
H	-0.046877	-2.744167	2.440984
C	1.909921	-1.164559	1.889141
H	2.855498	-0.612268	1.841701
H	1.394160	-0.858055	2.804546
H	2.156908	-2.231525	1.973396
C	1.959015	-1.262671	-0.609070
H	2.359488	-2.280349	-0.533430
H	1.417913	-1.159299	-1.557173
C	-1.924002	-3.207083	-0.660329
H	-2.034763	-3.975464	-1.436906
H	-2.479303	-3.528667	0.227454

Br	3.522724	-0.081063	-0.816964

K G3355.440853

C	-0.304544	-1.725878	0.545909
C	-1.139201	-0.663589	-0.192761
C	-2.903350	-0.789500	-0.444668
C	-0.925173	0.652009	0.558410
C	0.619649	0.631024	0.476719
C	1.093607	-0.889137	0.613992
C	1.183935	1.731924	1.382609
H	2.232578	1.900421	1.101798
C	0.342064	3.036764	1.214774
C	-0.534046	3.086536	-0.063561
C	-1.570232	1.925469	-0.026684
C	-2.225873	1.595748	-1.378739
C	-3.305262	0.491664	-1.237017
H	-3.651215	0.192832	-2.236992
H	-2.714376	2.484211	-1.799527
H	-0.328857	3.157859	2.081785
H	-1.457815	1.301293	-2.113180
H	-4.171551	0.926299	-0.719881
H	-2.376823	2.238695	0.659986
H	0.115555	2.941509	-0.945595
C	-1.220331	4.452935	-0.204040
H	-0.476130	5.257950	-0.262066
H	-1.840218	4.512353	-1.108780
H	-1.866793	4.658658	0.662406
H	1.009684	3.908902	1.227042
H	1.178339	1.421003	2.436477
H	0.876485	0.933761	-0.551385
H	-1.239836	0.512303	1.602437
H	-0.700565	-0.512807	-1.186785
C	-2.410030	-1.895802	-1.185160
C	-0.396715	-3.011469	-0.318636
H	-2.196133	-1.703865	-2.244067
H	-0.022789	-3.906173	0.196132
H	0.156000	-2.918801	-1.263053
C	-3.667674	-0.968343	0.857419
H	-4.737750	-0.927193	0.611747
H	-3.475997	-1.915336	1.364114
H	-3.461686	-0.145818	1.551620
C	-0.777605	-2.075624	1.974608
H	-0.880791	-1.202256	2.625500
H	-1.732583	-2.607727	1.979451
H	-0.046296	-2.744162	2.440992
C	1.910012	-1.163664	1.889258
H	2.855424	-0.611094	1.841993
H	1.394043	-0.857234	2.804587
H	2.157450	-2.230494	1.973729
C	1.959349	-1.262251	-0.608977
H	2.359612	-2.280022	-0.533258

H	1.418129	-1.158871	-1.557039
C	-1.923547	-3.207640	-0.660446
H	-2.033375	-3.976111	-1.437068
H	-2.478389	-3.529675	0.227489
Br	3.523042	-0.080785	-0.816819

K-L-TS G3355.392006, T-144

C	-0.699663	-1.743722	0.586725
C	-1.277905	-0.519754	-0.104969
C	-2.841328	-0.571933	-0.389617
C	-0.765515	0.751783	0.565684
C	0.758712	0.648270	0.358222
C	1.221378	-0.803792	0.614441
C	1.492291	1.763534	1.137386
H	2.484596	1.900561	0.686374
C	0.664861	3.077463	1.098644
C	-0.291082	3.180851	-0.109367
C	-1.362610	2.054384	-0.030489
C	-2.029973	1.745378	-1.382478
C	-3.170525	0.711828	-1.258115
H	-3.489919	0.407756	-2.265762
H	-2.445429	2.663707	-1.818904
H	0.061210	3.165292	2.017471
H	-1.266582	1.396494	-2.099083
H	-4.039601	1.205134	-0.800258
H	-2.151262	2.406114	0.658730
H	0.296326	3.028713	-1.034102
C	-0.934774	4.573033	-0.184506
H	-0.170998	5.351753	-0.310911
H	-1.634632	4.656606	-1.026593
H	-1.490983	4.797054	0.738217
H	1.351964	3.935283	1.103468
H	1.657807	1.472016	2.183516
H	0.936611	0.836444	-0.714258
H	-0.973000	0.715475	1.647777
H	-0.829978	-0.524133	-1.115012
C	-2.983077	-1.835234	-1.192092
C	-0.670814	-2.878838	-0.264026
H	-2.899411	-1.729940	-2.279574
H	-0.601301	-3.890555	0.142425
H	-0.409808	-2.783127	-1.316650
C	-3.701936	-0.550445	0.893004
H	-4.761425	-0.473502	0.618816
H	-3.589763	-1.439900	1.519636
H	-3.451689	0.323968	1.506059
C	-1.029481	-2.044116	2.043719
H	-1.076153	-1.144459	2.663119
H	-2.002994	-2.544626	2.122871
H	-0.292114	-2.725285	2.483295
C	1.788242	-1.144659	1.967176
H	2.793125	-0.695319	2.018010

H	1.208596	-0.731227	2.797776
H	1.913334	-2.225337	2.100752
C	1.949114	-1.452515	-0.542854
H	2.203140	-2.501780	-0.361633
H	1.424933	-1.339086	-1.495969
C	-2.969922	-3.106928	-0.678349
H	-2.994094	-3.968559	-1.345162
H	-3.176785	-3.305229	0.372278
Br	3.672581	-0.496224	-0.804730

**L**	G3355.438893		
C	-0.942035	-1.880419	-0.047728
C	-1.638569	-0.644379	-0.379379
C	-3.166131	-0.447071	-0.097006
C	-0.713662	0.494342	0.327835
C	0.741704	0.408672	-0.176685
C	1.373661	-1.013601	-0.031466
C	1.607407	1.515788	0.506637
H	2.418040	1.775819	-0.184887
C	0.804191	2.775535	0.874661
C	-0.353207	3.026748	-0.096299
C	-1.401558	1.887191	0.061347
C	-2.379868	1.831538	-1.119518
C	-3.559914	0.873618	-0.865701
H	-4.027024	0.616276	-1.826868
H	-2.775868	2.834747	-1.319534
H	0.389121	2.684618	1.892985
H	-1.830006	1.548344	-2.033531
H	-4.334056	1.386028	-0.277752
H	-1.984592	2.124617	0.966923
H	0.037298	2.997376	-1.130592
C	-0.992369	4.404682	0.140649
H	-0.248481	5.197147	-0.015633
H	-1.831977	4.603281	-0.537097
H	-1.361553	4.493287	1.173284
H	1.482321	3.640159	0.897169
H	2.092723	1.120254	1.406176
H	0.714469	0.628323	-1.259691
H	-0.725761	0.297288	1.410936
H	-1.462833	-0.464300	-1.453137
C	-3.901745	-1.606841	-0.756959
C	0.345743	-2.068739	-0.717123
H	-3.647746	-1.755980	-1.813712
H	0.750322	-3.078588	-0.588343
H	0.293286	-1.810868	-1.783448
C	-3.530763	-0.305010	1.397158
H	-4.593822	-0.049890	1.482780
H	-3.379024	-1.226276	1.970733
H	-2.966573	0.495856	1.886048
C	-1.399671	-2.828994	0.980383
H	-1.634551	-2.315962	1.923126

H	-2.380135	-3.216212	0.633283
H	-0.703439	-3.652821	1.155961
C	1.627661	-1.449668	1.424896
H	2.484177	-0.917372	1.848830
H	0.762563	-1.264235	2.073227
H	1.863578	-2.521175	1.468737
C	2.640070	-1.205744	-0.911448
H	2.875845	-2.270830	-1.014794
H	2.489638	-0.772685	-1.907358
C	-4.824071	-2.405342	-0.199355
H	-5.316806	-3.179441	-0.787246
H	-5.146184	-2.302955	0.836599
Br	4.276452	-0.394791	-0.199502

Cartesian coordinates of computed structures (venezuelaxenene, Table S9)

Gibbs energies (G"..." in Hartree) and imagininary frequencies of TS (T-"..." in cm^{-1}), mPW1PW91/6-311+G(d,p)//B97D3/6-31G(d,p)-sp-density-fitting, 1 bar, 298.15 K

**M’**	G781.673512		
C	-4.800366	0.227071	0.802757
C	0.582873	1.756739	-0.304908
C	1.792750	1.351359	-0.926669
C	-5.812947	0.029550	-0.071271
H	-4.943402	0.972493	1.591370
H	-0.290178	1.908599	-0.936234
H	1.696085	0.844909	-1.889718
C	-7.115659	0.780209	0.075636
H	-7.333479	1.364669	-0.832677
H	-7.955784	0.078782	0.199857
H	-7.109617	1.463894	0.933355
C	-5.758344	-0.930083	-1.236830
H	-4.809501	-1.472772	-1.324098
H	-6.567173	-1.673513	-1.161999
H	-5.925974	-0.389732	-2.182014
C	3.069892	1.519060	-0.423073
C	3.396078	2.314136	0.805791
H	2.525770	2.673556	1.364023
H	4.029050	1.717101	1.475243
H	3.994812	3.192121	0.511238
C	4.249517	0.991557	-1.214158
H	4.364297	1.649407	-2.094941
H	5.168341	1.112287	-0.622299
C	4.046376	-1.113002	1.847794
H	5.130131	-1.070379	1.960214
H	3.462683	-1.001677	2.762317
C	4.135088	-0.470140	-1.707893
C	3.481801	-1.327424	0.644671
C	4.341484	-1.520113	-0.590040
H	0.612955	2.408312	0.566782
C	1.142220	-0.575755	1.432702
C	1.973961	-1.440588	0.453195
H	4.145208	-2.515494	-1.024783
H	5.399552	-1.515691	-0.289463
H	4.899099	-0.634986	-2.480782
H	3.168707	-0.626716	-2.210226
C	-3.460415	-0.455988	0.784760
H	-3.093212	-0.598206	1.811900
H	-3.512019	-1.450879	0.322755
C	-2.398050	0.416057	0.004469
H	-2.766416	0.543914	-1.023724
H	-2.350726	1.403726	0.484338
C	-1.066814	-0.265055	0.012956
H	-0.506219	0.818725	1.708678
C	-0.866988	-1.359497	-0.995429

H	-1.825452	-1.673940	-1.423715
H	-0.250372	-0.988563	-1.831235
H	-0.353044	-2.233455	-0.581185
H	0.840204	-1.178219	2.306024
H	1.792110	0.206663	1.847763
H	1.735329	-1.181011	-0.583223
H	1.691569	-2.501710	0.557508
C	-0.109678	0.129871	0.953696

M’-N’-TS G781.676064, T-380

C	-4.777422	0.314564	0.772803
C	0.610349	1.719379	-0.340693
C	1.836264	1.292682	-0.964314
C	-5.818992	0.012366	-0.036485
H	-4.900213	1.140641	1.480035
H	-0.220152	1.939193	-1.012410
H	1.733784	0.749461	-1.907233
C	-7.127366	0.756879	0.084194
H	-7.389173	1.239527	-0.871010
H	-7.949390	0.060665	0.313382
H	-7.100620	1.526485	0.865082
C	-5.792195	-1.063459	-1.096097
H	-4.837388	-1.597783	-1.167802
H	-6.582646	-1.806794	-0.909224
H	-6.009834	-0.627493	-2.083896
C	3.107521	1.475992	-0.474958
C	3.442751	2.305817	0.731254
H	2.575362	2.696180	1.273733
H	4.058808	1.718865	1.425215
H	4.056980	3.164993	0.415460
C	4.288597	0.909385	-1.243585
H	4.410615	1.514260	-2.159496
H	5.207004	1.057126	-0.656655
C	3.975535	-1.058897	1.944371
H	5.055765	-1.024681	2.088528
H	3.365654	-0.907229	2.835931
C	4.167757	-0.577821	-1.648487
C	3.444481	-1.307158	0.732935
C	4.336927	-1.563109	-0.467264
H	0.683575	2.452367	0.463714
C	1.085819	-0.483369	1.406184
C	1.940772	-1.411829	0.504136
H	4.150415	-2.581355	-0.851095
H	5.386104	-1.544426	-0.137071
H	4.945499	-0.799950	-2.393227
H	3.209982	-0.752868	-2.161853
C	-3.433116	-0.354341	0.779261
H	-3.027527	-0.387377	1.800713
H	-3.481592	-1.388436	0.414741
C	-2.406321	0.458712	-0.126487
H	-2.813472	0.472975	-1.146626

H	-2.361699	1.486271	0.259181
C	-1.081095	-0.210957	-0.079255
H	-0.532306	0.959508	1.556495
C	-0.875517	-1.377296	-0.992769
H	-1.819180	-1.698261	-1.447261
H	-0.201181	-1.081666	-1.814726
H	-0.399494	-2.227980	-0.491069
H	0.685912	-1.052892	2.260554
H	1.742401	0.275813	1.850649
H	1.738592	-1.212759	-0.553036
H	1.643033	-2.462043	0.663776
C	-0.095353	0.279072	0.814673

N’ G781.681337

C	-4.719287	0.435821	0.708152
C	0.632599	1.652483	-0.222716
C	1.845449	1.223001	-0.946402
C	-5.793936	0.021687	-0.008485
H	-4.815274	1.353692	1.295722
H	-0.134714	2.067472	-0.887525
H	1.683251	0.650616	-1.865400
C	-7.103797	0.765348	0.078852
H	-7.413166	1.119800	-0.917399
H	-7.904822	0.096149	0.429708
H	-7.053079	1.627528	0.754230
C	-5.798225	-1.182158	-0.918066
H	-4.836998	-1.706076	-0.975424
H	-6.560820	-1.906046	-0.591637
H	-6.080607	-0.881307	-1.939072
C	3.134448	1.424773	-0.551830
C	3.550723	2.284718	0.611389
H	2.721273	2.662654	1.218887
H	4.232348	1.727430	1.266772
H	4.111398	3.155457	0.234029
C	4.268545	0.816546	-1.366539
H	4.313897	1.324092	-2.344907
H	5.226403	1.026837	-0.867827
C	3.897301	-0.916292	2.040386
H	4.973643	-0.852293	2.202094
H	3.268162	-0.726443	2.911086
C	4.139846	-0.704526	-1.604504
C	3.394606	-1.235637	0.834106
C	4.315657	-1.561620	-0.329158
H	0.847889	2.401237	0.546527
C	0.972035	-0.448638	1.401236
C	1.898473	-1.395271	0.586206
H	4.169320	-2.618406	-0.615360
H	5.357847	-1.478166	0.013747
H	4.907635	-1.012449	-2.329557
H	3.174307	-0.925738	-2.086078
C	-3.380395	-0.227791	0.753429

H	-2.922133	-0.111301	1.744736
H	-3.429932	-1.299680	0.528495
C	-2.391208	0.471313	-0.322429
H	-2.850606	0.327380	-1.307731
H	-2.350911	1.538873	-0.071387
C	-1.085181	-0.187849	-0.208437
H	-0.621225	1.003101	1.411537
C	-0.874412	-1.446652	-0.967379
H	-1.792346	-1.798901	-1.449735
H	-0.128073	-1.254842	-1.759481
H	-0.441625	-2.241199	-0.344765
H	0.425612	-1.024403	2.162791
H	1.595190	0.264964	1.955922
H	1.719877	-1.276252	-0.486804
H	1.633941	-2.443436	0.809743
C	-0.074186	0.412354	0.657866

N’ G781.682447

C	-4.292907	-0.884106	-0.260879
C	0.370513	-1.027111	-1.070496
C	1.365784	-1.373848	-0.008481
C	-5.484011	-0.425899	0.207233
H	-4.128625	-1.965857	-0.271566
H	-0.558750	-1.610510	-0.976002
H	1.015801	-1.218704	1.018406
C	-6.567021	-1.395437	0.607848
H	-6.849624	-1.241982	1.661612
H	-7.479341	-1.218826	0.017099
H	-6.265922	-2.441256	0.476493
C	-5.839645	1.031770	0.356599
H	-5.039356	1.723757	0.070614
H	-6.728976	1.270967	-0.246564
H	-6.114272	1.244821	1.401430
C	2.665102	-1.713864	-0.177009
C	3.310799	-2.008059	-1.506185
H	2.630006	-1.946898	-2.362727
H	4.146843	-1.315741	-1.680194
H	3.737002	-3.023560	-1.491467
C	3.572883	-1.851235	1.042652
H	3.305610	-2.771665	1.588585
H	4.610347	-1.991929	0.702495
C	4.335120	1.355859	-0.919205
H	5.390916	1.084908	-0.902252
H	3.964379	1.789880	-1.848971
C	3.521085	-0.658420	2.020247
C	3.555672	1.176478	0.162639
C	4.136813	0.645994	1.462030
H	0.763335	-1.237242	-2.072430
C	1.310505	1.393967	-1.147146
C	2.090816	1.604789	0.184188
H	4.031285	1.424275	2.239032

H	5.217997	0.494744	1.324287
H	4.074840	-0.926293	2.932646
H	2.484145	-0.482718	2.348343
C	-3.155765	-0.054441	-0.741595
H	-2.658130	-0.521953	-1.602066
H	-3.456283	0.960015	-1.028842
C	-2.026951	0.064052	0.431523
H	-2.533894	0.469729	1.313698
H	-1.678045	-0.955398	0.626606
C	-0.977145	0.957542	-0.052464
H	-0.551870	0.683282	-1.969800
C	-1.063217	2.391115	0.328477
H	-2.080620	2.675138	0.623594
H	-0.424685	2.534905	1.220343
H	-0.671744	3.072674	-0.435619
H	1.050062	2.363409	-1.594078
H	1.958795	0.893283	-1.876451
H	1.581301	1.082291	1.006210
H	2.058237	2.673612	0.457771
C	0.038106	0.508994	-1.031681

N’-O’-TS G781.676536, T-748

C	-4.369124	-0.885814	-0.284104
C	0.346957	-1.001952	-1.046791
C	1.359800	-1.397850	0.002604
C	-5.550589	-0.440434	0.196327
H	-4.203639	-1.967435	-0.315010
H	-0.596657	-1.554582	-0.954618
H	1.007474	-1.285173	1.032651
C	-6.615390	-1.414767	0.643493
H	-6.878566	-1.247073	1.699898
H	-7.542011	-1.266469	0.066944
H	-6.301867	-2.459330	0.526840
C	-5.927367	1.016705	0.331175
H	-5.145671	1.715316	0.008955
H	-6.834831	1.234029	-0.253128
H	-6.174798	1.248992	1.378889
C	2.659644	-1.718917	-0.196901
C	3.280651	-1.994529	-1.541390
H	2.590191	-1.903982	-2.388114
H	4.129978	-1.319545	-1.714168
H	3.683561	-3.019436	-1.550604
C	3.578254	-1.885583	1.010922
H	3.291070	-2.800300	1.555167
H	4.607658	-2.051271	0.658985
C	4.414791	1.302326	-0.936486
H	5.457731	0.985502	-0.939025
H	4.046686	1.753565	-1.859683
C	3.561983	-0.695187	1.994411
C	3.647138	1.157786	0.158993
C	4.218811	0.594007	1.447989

H	0.732114	-1.155384	-2.061658
C	1.374251	1.368524	-1.099880
C	2.209097	1.666656	0.195039
H	4.144683	1.365961	2.234282
H	5.293198	0.408248	1.300777
H	4.105909	-0.985779	2.905457
H	2.529771	-0.490564	2.320167
C	-3.220427	-0.040599	-0.763178
H	-2.755785	-0.510379	-1.647556
H	-3.551377	0.961104	-1.074148
C	-2.120177	0.115886	0.338229
H	-2.584226	0.586516	1.214706
H	-1.764334	-0.874643	0.647587
C	-0.970832	1.001160	-0.099473
H	-0.893564	0.936561	-1.440398
C	-1.062899	2.448620	0.318978
H	-2.096825	2.806078	0.241678
H	-0.781713	2.496355	1.382502
H	-0.399853	3.123477	-0.229338
H	1.052003	2.306982	-1.569220
H	1.998785	0.847793	-1.832835
H	1.695330	1.247914	1.071115
H	2.227891	2.756231	0.357017
C	0.170710	0.489084	-0.798978

**O’**	G781.687942		
C	3.988794	-0.004711	-0.792855
C	-1.073134	-1.348987	-1.878703
C	-1.268818	0.126029	-1.575749
C	4.524410	0.938118	0.020521
H	4.119839	0.123166	-1.872366
H	-0.286327	-1.537803	-2.619458
H	-0.352583	0.675235	-1.359571
C	5.319906	2.086940	-0.551055
H	4.873676	3.052759	-0.263817
H	6.343334	2.089338	-0.143489
H	5.386313	2.046994	-1.645089
C	4.403442	0.930590	1.525905
H	3.793676	0.110019	1.921599
H	5.400340	0.866527	1.989721
H	3.965382	1.878288	1.877082
C	-2.454671	0.786790	-1.428087
C	-3.796246	0.239451	-1.832347
H	-3.780517	-0.809731	-2.146964
H	-4.524434	0.348620	-1.019654
H	-4.176863	0.836778	-2.676999
C	-2.417920	2.234406	-0.958277
H	-1.954868	2.850314	-1.747341
H	-3.446389	2.606832	-0.842988
C	-3.973915	0.000430	1.650332
H	-4.793417	0.716386	1.713213

H	-4.251799	-1.055076	1.664141
C	-1.635234	2.451032	0.357716
C	-2.691604	0.410493	1.578702
C	-2.343118	1.887635	1.610412
H	-2.005318	-1.829608	-2.200097
C	-1.730408	-1.784424	0.559992
C	-1.549008	-0.596637	1.598652
H	-1.691569	2.079315	2.480340
H	-3.265740	2.461283	1.782633
H	-1.492463	3.531353	0.506569
H	-0.623916	2.025084	0.267230
C	3.181488	-1.201028	-0.367271
H	3.398382	-2.059850	-1.023207
H	3.432573	-1.506109	0.655458
C	1.676455	-0.858451	-0.469231
H	1.434387	-0.063341	0.251637
H	1.480571	-0.459253	-1.472980
C	0.732198	-2.100672	-0.201505
H	1.013516	-2.818095	-1.001513
C	0.999363	-2.736545	1.171556
H	2.025859	-3.114814	1.211183
H	0.879507	-2.014051	1.989114
H	0.335759	-3.589188	1.360781
H	-1.650488	-2.736639	1.105258
H	-2.728956	-1.729768	0.116765
H	-0.597061	-0.081987	1.418417
H	-1.474065	-1.033613	2.604435
C	-0.689704	-1.770356	-0.498713

**O’**	G781.695302		
C	4.567554	1.009539	-0.010130
C	-0.440293	-1.170817	1.243210
C	-1.660983	-1.510187	0.402034
C	5.559691	0.098697	0.136358
H	4.666700	1.960225	0.523086
H	0.342956	-1.934127	1.192902
H	-1.454607	-1.910898	-0.590612
C	6.783157	0.417010	0.961685
H	6.901064	-0.310650	1.780764
H	7.695143	0.339781	0.348692
H	6.744928	1.423590	1.395588
C	5.554293	-1.272185	-0.497504
H	4.649416	-1.493351	-1.075524
H	6.421344	-1.389378	-1.166393
H	5.659443	-2.048794	0.276488
C	-2.973640	-1.290386	0.744978
C	-3.438904	-0.990935	2.143467
H	-2.675387	-0.544665	2.788493
H	-4.320383	-0.342980	2.142750
H	-3.743067	-1.945960	2.606837
C	-4.030916	-1.546581	-0.312845

H	-3.972077	-2.601122	-0.628892
H	-5.032827	-1.400898	0.114603
C	-3.908209	2.040916	0.849871
H	-4.980613	1.951674	1.024424
H	-3.338405	2.581791	1.607722
C	-3.859748	-0.654109	-1.568186
C	-3.332754	1.523621	-0.253697
C	-4.168447	0.837304	-1.318723
H	-0.682909	-0.937493	2.283795
C	-0.894475	1.319338	0.650971
C	-1.849491	1.742937	-0.528566
H	-4.030709	1.372589	-2.273888
H	-5.232083	0.940099	-1.057057
H	-4.538555	-1.021593	-2.351369
H	-2.839907	-0.772882	-1.966539
C	3.302506	0.825638	-0.803284
H	2.970928	1.790239	-1.217241
H	3.453602	0.148038	-1.653597
C	2.183872	0.264001	0.116844
H	2.510452	-0.679238	0.577326
H	2.020384	0.977261	0.938390
C	0.837904	-0.001563	-0.682495
H	0.687045	0.862744	-1.348148
C	0.949096	-1.276941	-1.533438
H	1.814982	-1.203721	-2.200536
H	1.081484	-2.177774	-0.920537
H	0.062077	-1.408859	-2.165429
H	-0.106919	2.085663	0.743571
H	-1.441461	1.300697	1.599738
H	-1.554700	1.219016	-1.448091
H	-1.690218	2.812972	-0.725453
C	-0.188066	0.027706	0.400138

O‘-P‘-TS G781.668771, T-832

C	-4.293987	-0.589879	-0.857710
C	0.849801	1.529355	0.951957
C	2.074682	0.946473	1.465869
C	-5.076129	-0.997423	0.166506
H	-4.187813	-1.262774	-1.715095
H	1.075937	2.286514	-0.098088
H	2.812238	1.660817	1.843187
C	-5.808155	-2.317870	0.102332
H	-5.518178	-2.968482	0.943180
H	-6.895221	-2.164063	0.193126
H	-5.615864	-2.856551	-0.833807
C	-5.306588	-0.203661	1.431922
H	-4.749848	0.740035	1.474718
H	-6.376439	0.029640	1.550395
H	-5.025240	-0.801425	2.313485
C	2.381380	-0.384714	1.496049
C	1.356878	-1.475386	1.378607

H	0.338684	-1.111499	1.200132
H	1.639030	-2.195829	0.600108
H	1.343828	-2.030377	2.331081
C	3.819081	-0.784185	1.766307
H	4.094647	-0.486609	2.791644
H	3.915224	-1.878652	1.721635
C	2.890435	-1.955739	-1.757182
H	3.485888	-2.861152	-1.639698
H	1.941186	-2.071317	-2.283621
C	4.797396	-0.112757	0.770981
C	3.333584	-0.766780	-1.306890
C	4.712526	-0.656698	-0.676907
H	0.314859	2.262967	1.565027
C	0.975787	0.346447	-1.347802
C	2.521263	0.510899	-1.532310
H	5.344649	-0.009141	-1.309454
H	5.176065	-1.653825	-0.704180
H	5.826260	-0.263911	1.128304
H	4.634423	0.975679	0.779499
C	-3.507434	0.694055	-0.932456
H	-3.500650	1.070049	-1.968670
H	-3.967011	1.475908	-0.314553
C	-2.046455	0.457417	-0.478178
H	-2.038430	0.171188	0.586101
H	-1.642257	-0.396892	-1.040712
C	-1.124350	1.709291	-0.673231
H	-1.135734	1.956515	-1.747617
C	-1.629462	2.924184	0.120436
H	-2.569493	3.280658	-0.313123
H	-1.830424	2.677022	1.172333
H	-0.920475	3.763575	0.083599
H	0.463304	0.487033	-2.307540
H	0.764417	-0.690521	-1.046248
H	2.912794	1.317315	-0.897848
H	2.693558	0.848375	-2.566653
C	0.279094	1.243594	-0.336741

P’ G781.687363

C	-3.985097	-0.289305	-1.006734
C	0.866286	1.252080	1.306380
C	1.551612	0.096288	1.593885
C	-4.534461	-1.161738	-0.130054
H	-3.979253	-0.572278	-2.064642
H	0.909743	2.921233	0.139761
H	2.048867	0.115115	2.573558
C	-5.150400	-2.456682	-0.605162
H	-4.667942	-3.323278	-0.123738
H	-6.215345	-2.506527	-0.326791
H	-5.075845	-2.579588	-1.692832
C	-4.600857	-0.930901	1.361978
H	-4.141315	0.010279	1.685828

H	-5.647140	-0.932524	1.706560
H	-4.103457	-1.754318	1.900247
C	1.794951	-1.124632	0.888687
C	0.672120	-1.857351	0.235983
H	-0.038490	-1.190887	-0.262704
H	1.013044	-2.646260	-0.439453
H	0.099500	-2.333938	1.054535
C	3.081241	-1.826601	1.219733
H	3.020005	-2.136511	2.280251
H	3.182852	-2.744920	0.627347
C	2.723210	-1.160716	-2.000527
H	3.178135	-2.145746	-2.109689
H	1.826674	-0.975174	-2.590763
C	4.307948	-0.898210	1.051331
C	3.280440	-0.222415	-1.194300
C	4.553848	-0.558298	-0.443068
H	0.714314	1.902548	2.173442
C	1.188459	1.331920	-1.253348
C	2.731194	1.193800	-1.109326
H	5.263346	0.281595	-0.498210
H	5.037598	-1.420513	-0.923319
H	5.197806	-1.385623	1.471419
H	4.152120	0.024907	1.628618
C	-3.333720	1.028867	-0.668270
H	-3.450146	1.728393	-1.511097
H	-3.822496	1.496883	0.196904
C	-1.825114	0.840034	-0.372659
H	-1.722524	0.196784	0.518042
H	-1.390955	0.282963	-1.217994
C	-1.047357	2.148298	-0.150036
H	-1.134902	2.762507	-1.063335
C	-1.608039	2.963758	1.027898
H	-2.607612	3.346805	0.792806
H	-1.697970	2.351164	1.937302
H	-0.973885	3.831029	1.259405
H	0.945488	2.011822	-2.080953
H	0.745047	0.367931	-1.528358
H	3.067676	1.652808	-0.166853
H	3.230845	1.777146	-1.900570
C	0.506437	1.896168	0.006822

P’ G781.699057

C	2.247836	-1.113017	0.938126
C	0.298541	-0.223445	0.145806
C	-0.563716	-1.161760	-0.475137
C	2.535677	-1.815786	-0.215111
H	1.669572	-1.673798	1.677883
H	0.133889	-0.049688	1.209853
H	-0.587433	-1.170919	-1.567117
C	1.863114	-3.130540	-0.475522
H	1.354003	-3.119417	-1.453270

H	2.619982	-3.928505	-0.536552
H	1.137622	-3.400153	0.299386
C	3.487463	-1.336993	-1.273064
H	4.283094	-0.700008	-0.872782
H	3.940470	-2.185705	-1.801498
H	2.947391	-0.750408	-2.036991
C	-1.630325	-1.722405	0.192809
C	-1.675094	-1.969750	1.664759
H	-0.773541	-1.666423	2.205906
H	-2.548429	-1.480890	2.116684
H	-1.820271	-3.052575	1.817552
C	-2.894115	-1.959631	-0.591109
H	-2.700579	-2.636951	-1.438397
H	-3.656200	-2.432469	0.043842
C	-2.296382	1.335499	1.794383
H	-2.940038	0.785423	2.481838
H	-1.532803	1.969810	2.248628
C	-3.434870	-0.610399	-1.163286
C	-2.470337	1.288083	0.458844
C	-3.658906	0.527347	-0.134533
C	0.731813	0.989371	-0.642030
H	1.363260	0.656348	-1.487637
C	-0.529084	1.623381	-1.339967
H	-0.155937	2.437394	-1.975815
H	-0.949689	0.882474	-2.030526
C	-1.637151	2.206720	-0.434112
H	-2.331107	2.737940	-1.107507
H	-1.189137	2.982305	0.203819
H	-4.289314	1.278579	-0.641007
H	-4.271324	0.135129	0.692607
H	-4.403137	-0.832666	-1.634846
H	-2.771742	-0.272659	-1.971972
C	2.944039	0.128650	1.462977
H	2.488963	0.376335	2.434127
H	3.991663	-0.137257	1.684642
C	2.939285	1.396334	0.585454
H	3.503155	2.165316	1.133430
H	3.497186	1.228103	-0.348252
C	1.558014	1.981845	0.221216
H	0.997904	2.159100	1.158490
C	1.766775	3.337638	-0.477590
H	2.424183	3.976453	0.125962
H	2.243535	3.207268	-1.460700
H	0.829348	3.885673	-0.627259

P‘-Q‘-TS G781.701393, T-389

C	2.050320	-1.081497	0.860194
C	0.391561	-0.252149	0.188168
C	-0.538622	-1.166413	-0.432703
C	2.514918	-1.828445	-0.242410
H	1.522820	-1.709463	1.586408

H	0.098042	0.018730	1.207664
H	-0.550599	-1.188838	-1.525389
C	1.871413	-3.143865	-0.515192
H	2.207958	-3.606410	-1.448473
H	2.047101	-3.842117	0.317576
H	0.769572	-3.003144	-0.545638
C	3.546404	-1.338957	-1.202432
H	4.236306	-0.610661	-0.766576
H	4.111668	-2.179135	-1.625918
H	3.043967	-0.847983	-2.057231
C	-1.625225	-1.692806	0.218248
C	-1.704575	-1.908205	1.697123
H	-0.827991	-1.564119	2.255838
H	-2.599990	-1.429992	2.114538
H	-1.823118	-2.989936	1.876616
C	-2.875431	-1.950001	-0.586521
H	-2.667629	-2.643552	-1.416782
H	-3.647050	-2.414242	0.043633
C	-2.316897	1.373637	1.756870
H	-2.980676	0.845484	2.442292
H	-1.556145	2.009795	2.213567
C	-3.410481	-0.615103	-1.190118
C	-2.461845	1.297127	0.419394
C	-3.646647	0.538689	-0.182806
C	0.774474	0.975780	-0.639520
H	1.433113	0.653030	-1.468638
C	-0.481354	1.579733	-1.353487
H	-0.110410	2.381232	-2.007156
H	-0.896710	0.824636	-2.031941
C	-1.601610	2.187434	-0.477020
H	-2.279757	2.710390	-1.172669
H	-1.159625	2.973450	0.152580
H	-4.261880	1.287876	-0.710783
H	-4.276282	0.162624	0.638866
H	-4.371993	-0.843300	-1.673051
H	-2.735448	-0.289202	-1.994216
C	2.800755	0.090548	1.495838
H	2.290700	0.315452	2.444963
H	3.811683	-0.252157	1.768161
C	2.906277	1.392147	0.681383
H	3.433383	2.129630	1.304247
H	3.539409	1.248466	-0.208095
C	1.564286	1.995089	0.222839
H	0.959410	2.211262	1.123313
C	1.828605	3.321466	-0.509842
H	2.473569	3.969070	0.098365
H	2.340940	3.148573	-1.468251
H	0.907697	3.879293	-0.715865

**Q‘**	G781.706428		
C	1.861986	-1.047592	0.804956

C	0.469290	-0.285172	0.209812
C	-0.534704	-1.152146	-0.438120
C	2.429650	-1.871928	-0.243915
H	1.408201	-1.714135	1.551943
H	0.060187	0.062905	1.169723
H	-0.551309	-1.145821	-1.531560
C	1.756722	-3.151049	-0.513757
H	2.147634	-3.701873	-1.373848
H	1.743819	-3.795352	0.378985
H	0.669272	-2.904996	-0.659369
C	3.533646	-1.423289	-1.127425
H	4.288283	-0.838039	-0.587980
H	4.002363	-2.259213	-1.657715
H	3.116014	-0.741933	-1.893543
C	-1.637831	-1.655197	0.193035
C	-1.731795	-1.881240	1.672064
H	-0.869929	-1.520114	2.243910
H	-2.638446	-1.418669	2.082175
H	-1.833104	-2.965069	1.849980
C	-2.888337	-1.889568	-0.621482
H	-2.686880	-2.569828	-1.463979
H	-3.665629	-2.359121	-0.001959
C	-2.271554	1.390594	1.772646
H	-2.944498	0.871122	2.455663
H	-1.497129	2.008113	2.232150
C	-3.410766	-0.539920	-1.199560
C	-2.419514	1.325607	0.434835
C	-3.621846	0.599452	-0.170961
C	0.818050	0.959666	-0.644224
H	1.470812	0.639544	-1.481146
C	-0.433936	1.574936	-1.340016
H	-0.062749	2.373094	-1.997906
H	-0.869195	0.830538	-2.017524
C	-1.541050	2.201530	-0.458086
H	-2.209749	2.741068	-1.150147
H	-1.082782	2.976203	0.174206
H	-4.226722	1.369166	-0.681314
H	-4.252667	0.220458	0.648492
H	-4.378566	-0.744717	-1.680845
H	-2.735926	-0.208658	-2.001865
C	2.720326	0.032663	1.494920
H	2.203959	0.276957	2.436044
H	3.689477	-0.404098	1.779226
C	2.929146	1.331451	0.703717
H	3.465441	2.042719	1.348843
H	3.587658	1.163095	-0.163603
C	1.622712	1.976402	0.205485
H	1.010271	2.228827	1.091278
C	1.940503	3.278200	-0.547707
H	2.601126	3.914936	0.055555
H	2.455753	3.069990	-1.497601

H	1.040835	3.862506	-0.773572

Q' G781.706428

C	1.862161	-1.047507	0.804781
C	0.469378	-0.285137	0.209846
C	-0.534711	-1.152142	-0.437922
C	2.429899	-1.871865	-0.244025
H	1.408535	-1.714114	1.551805
H	0.060403	0.062907	1.169829
H	-0.551438	-1.145939	-1.531358
C	1.757074	-3.151165	-0.513709
H	2.147951	-3.701798	-1.373953
H	1.744995	-3.795522	0.379000
H	0.669617	-2.905453	-0.659012
C	3.533658	-1.423196	-1.127701
H	4.287929	-0.836979	-0.588844
H	4.002815	-2.259073	-1.657650
H	3.115314	-0.742585	-1.894153
C	-1.637645	-1.655310	0.193401
C	-1.731510	-1.881170	1.672455
H	-0.869211	-1.520741	2.244103
H	-2.637638	-1.417699	2.082707
H	-1.833759	-2.964872	1.850532
C	-2.888285	-1.889853	-0.620982
H	-2.686988	-2.570763	-1.463003
H	-3.665582	-2.358975	-0.001116
C	-2.272039	1.390844	1.772332
H	-2.945105	0.871516	2.455341
H	-1.497741	2.008515	2.231845
C	-3.410558	-0.540524	-1.199662
C	-2.419743	1.325546	0.434504
C	-3.621882	0.599166	-0.171384
C	0.818042	0.959749	-0.644141
H	1.470964	0.639787	-1.481005
C	-0.433937	1.574849	-1.340157
H	-0.062784	2.373014	-1.998062
H	-0.869039	0.830371	-2.017672
C	-1.541197	2.201412	-0.458403
H	-2.209874	2.740830	-1.150581
H	-1.083073	2.976182	0.173871
H	-4.226693	1.368713	-0.682061
H	-4.252849	0.220299	0.648014
H	-4.378291	-0.745373	-1.681061
H	-2.735612	-0.209413	-2.001947
C	2.720454	0.032820	1.494796
H	2.204097	0.276888	2.435988
H	3.689690	-0.403818	1.779003
C	2.929032	1.331782	0.703841
H	3.465166	2.043031	1.349125
H	3.587615	1.163761	-0.163500
C	1.622485	1.976565	0.205663

H	1.010013	2.228795	1.091489
C	1.940054	3.278488	-0.547388
H	2.600689	3.915214	0.055872
H	2.455206	3.070462	-1.497369
H	1.040290	3.862723	-0.773050

Q‘-R‘-TS G781.697257, T-109

C	-1.718780	1.059825	0.817548
C	-0.507760	0.180104	0.270863
C	0.528595	0.987168	-0.499733
C	-1.875774	2.088509	-0.233174
H	-1.325802	1.575809	1.705433
H	-0.026361	-0.192032	1.193248
H	0.511790	0.854071	-1.585071
C	-1.380287	3.464111	-0.009593
H	-0.999979	3.927025	-0.929128
H	-2.276960	4.055300	0.276716
H	-0.656139	3.541247	0.805640
C	-2.621971	1.812052	-1.480186
H	-3.622274	1.419242	-1.237328
H	-2.703786	2.681915	-2.139018
H	-2.133809	0.987379	-2.027574
C	1.655717	1.516152	0.035729
C	1.813370	1.872360	1.488672
H	1.023446	1.468988	2.133709
H	2.783355	1.545283	1.883000
H	1.801536	2.971724	1.587522
C	2.884252	1.631961	-0.842726
H	2.670711	2.203454	-1.759820
H	3.690676	2.160212	-0.313593
C	2.141432	-1.307881	1.928987
H	2.821873	-0.714328	2.539223
H	1.360114	-1.853567	2.461316
C	3.359101	0.207920	-1.257289
C	2.286187	-1.413236	0.586792
C	3.513971	-0.813749	-0.099635
C	-0.918273	-1.049750	-0.568049
H	-1.449106	-0.697968	-1.479221
C	0.312225	-1.809469	-1.149563
H	-0.085278	-2.657721	-1.723074
H	0.806290	-1.172740	-1.892269
C	1.367961	-2.369689	-0.169650
H	2.010213	-3.041903	-0.763861
H	0.858912	-3.011468	0.565130
H	4.080073	-1.666923	-0.512787
H	4.162902	-0.372285	0.672605
H	4.337713	0.305856	-1.750919
H	2.672499	-0.189807	-2.018397
C	-2.946803	0.210179	1.244564
H	-2.736499	-0.093329	2.281748
H	-3.842852	0.847682	1.289299

C	-3.210319	-1.056112	0.422545
H	-3.979694	-1.646920	0.940992
H	-3.637113	-0.809164	-0.565052
C	-1.944120	-1.910706	0.207430
H	-1.517624	-2.149870	1.199968
C	-2.302839	-3.227848	-0.495928
H	-3.116169	-3.736108	0.038930
H	-2.643580	-3.048864	-1.526969
H	-1.454392	-3.921580	-0.536012

R‘ G781.720212

C	-1.548001	1.138544	0.786323
C	-0.535391	0.030053	0.429244
C	0.322870	1.073038	-0.472974
C	-0.963580	2.061486	-0.331965
H	-1.251869	1.583771	1.750003
H	0.037971	-0.339211	1.285550
H	0.437251	0.711269	-1.500791
C	-0.726138	3.520695	0.065564
H	-0.051504	4.029414	-0.638221
H	-1.687524	4.052980	0.036831
H	-0.335299	3.639820	1.081806
C	-1.716326	2.033195	-1.672470
H	-2.704750	2.493996	-1.542099
H	-1.172739	2.614531	-2.430156
H	-1.875224	1.024912	-2.069307
C	1.619236	1.348034	0.112372
C	1.772016	2.061617	1.409147
H	0.935423	1.900724	2.095422
H	2.721434	1.834560	1.901258
H	1.784915	3.141963	1.172549
C	2.852614	1.115940	-0.699511
H	2.821448	1.932685	-1.451776
H	3.752688	1.282684	-0.092851
C	2.518193	-1.007699	1.777427
H	3.441676	-0.450592	1.939043
H	1.857535	-1.119248	2.638446
C	2.961583	-0.212032	-1.482586
C	2.253436	-1.603456	0.590465
C	3.225776	-1.434195	-0.563785
C	-1.037995	-1.154117	-0.401061
H	-1.382632	-0.788460	-1.383768
C	0.073780	-2.185230	-0.718581
H	-0.406576	-3.109625	-1.061809
H	0.642688	-1.829280	-1.590011
C	1.061390	-2.531703	0.419498
H	1.482892	-3.528137	0.201883
H	0.527393	-2.633725	1.376101
H	3.220153	-2.333097	-1.197971
H	4.243405	-1.335774	-0.158153
H	3.798473	-0.117367	-2.187893

H	2.063802	-0.368011	-2.094155
C	-3.031198	0.741806	0.890429
H	-3.258376	0.597076	1.958019
H	-3.672112	1.572701	0.558071
C	-3.401897	-0.558740	0.151799
H	-4.365809	-0.920978	0.537364
H	-3.554962	-0.369287	-0.923700
C	-2.336071	-1.671071	0.295644
H	-2.129375	-1.825120	1.371893
C	-2.852241	-2.991786	-0.293759
H	-3.828508	-3.240964	0.142968
H	-2.984508	-2.916499	-1.383474
H	-2.179714	-3.833978	-0.088395

**R'**	G781.720199		
C	-1.547404	1.139815	0.785956
C	-0.535672	0.030283	0.429374
C	0.323893	1.071680	-0.472138
C	-0.961408	2.061953	-0.332082
H	-1.251219	1.584783	1.749754
H	0.036652	-0.339560	1.286169
H	0.438300	0.709896	-1.499971
C	-0.722610	3.520973	0.065202
H	-0.047529	4.028980	-0.638675
H	-1.683503	4.054142	0.036422
H	-0.331679	3.639931	1.081441
C	-1.713343	2.034128	-1.673092
H	-2.701228	2.496353	-1.543578
H	-1.168497	2.614373	-2.430717
H	-1.873382	1.025849	-2.069524
C	1.620717	1.346120	0.113061
C	1.773941	2.060272	1.409447
H	0.937585	1.899639	2.096091
H	2.723584	1.833738	1.901339
H	1.786531	3.140494	1.172208
C	2.853708	1.113763	-0.699373
H	2.822545	1.930593	-1.451546
H	3.754103	1.280152	-0.093082
C	2.516931	-1.007758	1.776750
H	3.440801	-0.451138	1.937825
H	1.856228	-1.118073	2.637891
C	2.961662	-0.214126	-1.482613
C	2.251852	-1.604613	0.590348
C	3.224279	-1.436842	-0.564036
C	-1.039264	-1.153686	-0.400710
H	-1.383339	-0.787971	-1.383614
C	0.071800	-2.185707	-0.717938
H	-0.409159	-3.109913	-1.060860
H	0.640892	-1.830361	-1.589492
C	1.059314	-2.532315	0.420243
H	1.480192	-3.529121	0.203154

H	0.525395	-2.633396	1.376971
H	3.217139	-2.335603	-1.198377
H	4.242096	-1.339985	-0.158520
H	3.798830	-0.120194	-2.187690
H	2.063880	-0.369231	-2.094419
C	-3.031078	0.744601	0.889465
H	-3.258993	0.600706	1.957002
H	-3.671032	1.575886	0.556247
C	-3.402680	-0.556008	0.151324
H	-4.366985	-0.917269	0.536827
H	-3.555354	-0.366860	-0.924288
C	-2.337861	-1.669262	0.295800
H	-2.131600	-1.823136	1.372164
C	-2.855028	-2.989714	-0.293294
H	-3.831504	-3.238102	0.143406
H	-2.987157	-2.914610	-1.383037
H	-2.183095	-3.832338	-0.087697

R‘-S‘-TS G781.721144, T-408

C	1.374542	1.386865	-0.718305
C	0.604125	0.055141	-0.559249
C	-0.520032	0.773351	0.253700
C	0.471988	2.050081	0.378839
H	1.073838	1.839705	-1.677762
H	0.283511	-0.359804	-1.526058
H	-0.610070	0.305768	1.244368
C	0.009374	3.475187	0.054169
H	-0.819675	3.797887	0.701380
H	0.844448	4.166367	0.238315
H	-0.283921	3.609704	-0.992504
C	1.072678	2.067504	1.798111
H	1.944220	2.735454	1.827315
H	0.335901	2.451074	2.518933
H	1.403882	1.084585	2.151955
C	-1.929023	0.817290	-0.324386
C	-2.259454	1.867093	-1.368563
H	-1.487283	1.938356	-2.142300
H	-3.231746	1.689541	-1.841526
H	-2.316663	2.842137	-0.865152
C	-3.027194	0.591635	0.715016
H	-2.945342	1.430045	1.430581
H	-4.016950	0.683014	0.243202
C	-2.251139	-0.778310	-1.474180
H	-3.309589	-0.561927	-1.615821
H	-1.631940	-0.632711	-2.358806
C	-2.922614	-0.720646	1.505561
C	-1.917101	-1.790482	-0.563624
C	-2.852223	-1.994998	0.586900
C	1.306711	-1.048723	0.240228
H	1.466333	-0.679136	1.267856
C	0.411422	-2.310441	0.435743

H	1.037945	-3.206858	0.509837
H	-0.110635	-2.238722	1.401295
C	-0.655981	-2.587395	-0.669396
H	-0.970918	-3.638989	-0.546243
H	-0.217435	-2.488822	-1.670331
H	-2.568351	-2.864231	1.192128
H	-3.861771	-2.171354	0.183100
H	-3.795277	-0.827340	2.162495
H	-2.040081	-0.715754	2.159227
C	2.911560	1.334651	-0.680919
H	3.267166	1.350627	-1.723241
H	3.316653	2.245249	-0.213042
C	3.503515	0.080447	-0.004020
H	4.554429	-0.023613	-0.311090
H	3.517081	0.201997	1.092371
C	2.740832	-1.231986	-0.321981
H	2.688184	-1.354127	-1.421165
C	3.492624	-2.433670	0.268924
H	4.538282	-2.430617	-0.066105
H	3.497030	-2.397255	1.368912
H	3.061099	-3.395817	-0.037539

**S‘**	G781.730189		
C	1.303766	1.445077	-0.709030
C	0.610259	0.065609	-0.605494
C	-0.567550	0.711331	0.189731
C	0.335002	2.024126	0.383059
H	0.996110	1.903856	-1.663405
H	0.363730	-0.349677	-1.595945
H	-0.648075	0.207619	1.167115
C	-0.186866	3.434710	0.078106
H	-1.033808	3.709470	0.723773
H	0.615793	4.159219	0.278476
H	-0.482846	3.569876	-0.967409
C	0.910466	2.049488	1.814312
H	1.736851	2.770107	1.880589
H	0.135804	2.367874	2.527579
H	1.294335	1.080716	2.155241
C	-2.007680	0.652561	-0.413005
C	-2.414624	1.795229	-1.354747
H	-1.667743	1.956528	-2.141406
H	-3.379153	1.580339	-1.833446
H	-2.525456	2.727860	-0.792152
C	-3.056324	0.464985	0.711269
H	-2.976774	1.319743	1.401904
H	-4.066445	0.507959	0.274023
C	-2.148095	-0.692751	-1.339698
H	-3.217705	-0.715576	-1.589011
H	-1.548348	-0.585642	-2.247170
C	-2.888677	-0.829945	1.520283
C	-1.786895	-1.838177	-0.523507

C	-2.682369	-2.113120	0.622403
C	1.365285	-1.012331	0.188652
H	1.451642	-0.659787	1.230758
C	0.575337	-2.349098	0.321454
H	1.248358	-3.210337	0.240180
H	0.114930	-2.419549	1.316531
C	-0.564104	-2.627963	-0.746929
H	-0.833758	-3.687292	-0.597227
H	-0.173793	-2.490063	-1.761829
H	-2.345456	-2.963987	1.226022
H	-3.669812	-2.364879	0.194377
H	-3.771470	-1.007729	2.147033
H	-2.027438	-0.768119	2.199356
C	2.838921	1.485928	-0.637441
H	3.219009	1.530384	-1.670447
H	3.174985	2.416237	-0.153731
C	3.488128	0.267319	0.048537
H	4.554834	0.236217	-0.217829
H	3.451331	0.374324	1.145796
C	2.830425	-1.089397	-0.314851
H	2.829515	-1.200125	-1.416435
C	3.642825	-2.241688	0.295902
H	4.697978	-2.159255	0.003938
H	3.600885	-2.216642	1.395445
H	3.298596	-3.231017	-0.034720

References

[1] J. Rinkel, S. T. Steiner, G. Bian, R. Chen, T. Liu, J. S. Dickschat, *ChemBioChem* **2020**, *21*, 486-491.

[2] C. Zhu, B. Xu, D. A. Adpressa, J. D. Rudolf, S. Loesgen, *Angew. Chem. Int. Ed.* **2021**, *60*, 14163-14170; *Angew. Chem.* **2021**, *133*, 14282-14289.

[3] G. Li, Y.-W. Guo, J. S. Dickschat, *Angew. Chem. Int. Ed.* **2021**, *60*, 1488-1492; *Angew. Chem.* **2021**, *133*, 1510-1514.

[4] G. Bian, J. Rinkel, Z. Wang, L. Lauterbach, A. Hou, Y. Yuan, Z. Deng, T. Liu, J. S. Dickschat, *Angew. Chem. Int. Ed.* **2018**, *57*, 15887-15890; *Angew. Chem.* **2018**, *130*, 16113-16117.

[5] J. Rinkel, S. T. Steiner, J. S. Dickschat, *Angew. Chem. Int. Ed.* **2019**, *58*, 9230-9233; *Angew. Chem.* **2019**, *131*, 9328-9332.

[6] J. S. Dickschat, J. Rinkel, P. Rabe, A. Beyraghdar Kashkooli, H. J. Bouwmeester, *Beilstein J. Org. Chem.* **2017**, *13*, 1770-1780.

[7] L. Lauterbach, J. Rinkel, J. S. Dickschat, *Angew. Chem. Int. Ed.* **2018**, *57*, 8280-8283; *Angew. Chem.* **2018**, *130*, 8412-8415.

[8] J. Rinkel, L. Lauterbach, P. Rabe, J. S. Dickschat, *Angew. Chem. Int. Ed.* **2018**, *57*, 3238-3241; *Angew. Chem.* **2018**, *130*, 3292-3296.

[9] K. A. Taizoumbe, S. T. Steiner, J. S. Dickschat, *Chem. Eur. J.* **2023**, *29*, e202302469.

[10] J. Rinkel, L. Lauterbach, J. S. Dickschat, *Angew. Chem. Int. Ed.* **2019**, *58*, 452-455; *Angew. Chem.* **2019**, *131*, 461-465.

[11] P. Rabe, J. Rinkel, E. Dolja, T. Schmitz, B. Nubbemeyer, T. H. Luu, J. S. Dickschat, *Angew. Chem. Int. Ed.* **2017**, *56*, 2776-2779; *Angew. Chem.* **2017**, *129*, 2820-2823.

[12] Z. Li, Y. Jiang, X. Zhang, Y. Chang, S. Li, X. Zhang, S. Zheng, C. Geng, P. Men, L. Ma, Y. Yang, Z. Gao, Y.-J. Tang, S. Li, *ACS Catal.* **2020**, *10*, 5846-5851.

[13] S. Grimme, S. Ehrlich, L. Goerigk, *J. Comp. Chem.* **2011**, *32*, 1456-1465.

[14] Gaussian 16, Revision C.01, M. J. Frisch, G. W. Trucks, H. B. Schlegel, G. E. Scuseria, M. A. Robb, J. R. Cheeseman, G. Scalmani, V. Barone, G. A. Petersson, H. Nakatsuji, X. Li, M. Caricato, A. V. Marenich, J. Bloino, B. G. Janesko, R. Gomperts, B. Mennucci, H. P. Hratchian, J. V. Ortiz, A. F. Izmaylov, J. L. Sonnenberg, D. Williams-Young, F. Ding, F. Lipparini, F. Egidi, J. Goings, B. Peng, A. Petrone, T. Henderson, D. Ranasinghe, V. G. Zakrzewski, J. Gao, N. Rega, G. Zheng, W. Liang, M. Hada, M. Ehara, K. Toyota, R. Fukuda, J. Hasegawa, M. Ishida, T. Nakajima, Y. Honda, O. Kitao, H. Nakai, T. Vreven, K. Throssell, J. A. Montgomery, Jr., J. E. Peralta, F. Ogliaro, M. J. Bearpark, J. J. Heyd, E. N. Brothers, K. N. Kudin, V. N. Staroverov, T. A. Keith, R. Kobayashi, J. Normand, K. Raghavachari, A. P. Rendell, J. C. Burant, S. S. Iyengar, J. Tomasi, M. Cossi, J. M. Millam, M. Klene, C. Adamo, R. Cammi, J. W. Ochterski, R. L. Martin, K. Morokuma, O. Farkas, J. B. Foresman, and D. J. Fox, Gaussian, Inc., Wallingford CT, 2019.

[15] S. Grimme, *Chem. Eur. J.* **2012**, *18*, 9955-9964.

[16] GoodVibes v3.0.1, G. Luchini, J. V. Alegre-Requena, Y. Guan, I. Funes-Ardoiz, R. S. Paton, 2019.

[17] C. Adamo, V. Barone, *J. Chem. Phys.* **1998**, *108*, 664.

[18] S. P. T. Matsuda, W. K. Wilson, Q. Xiong, *Org. Biomol. Chem.* **2006**, *4*, 530-543.

[19] Y. J. Hong, D. J. Tantillo, *J. Org. Chem.* **2018**, *83*, 3780-3793.

[20] L. Lauterbach, B. Goldfuss, J. S. Dickschat, *Angew. Chem.* **2020**, *132*, 12041-12045; *Angew. Chem. Int. Ed.* **2020**, *59*, 11943-11947.

[21] H. Xu, B. Goldfuss, J. S. Dickschat, *Chem. Eur. J.* **2021**, *27*, 9758-9762.

[22] P. Pracht, F. Bohle, S. Grimme, *Phys. Chem. Chem. Phys.* **2020**, *22*, 7169-7192.

[23] S. Grimme, *J. Chem. Theory Comput.* **2019**, *155*, 2847-2862.

[24] P. Pracht, S. Grimme, *Chem. Sci.* **2021**, *12*, 6551-6568.
[25] P. Pracht, C.A. Bauer, S. Grimme, *J. Comput. Chem.* **2017**, *38*, 2618-2631.
[26] S. Spicher, C. Plett, P. Pracht, A. Hansen, S. Grimme, *J. Chem. Theory Comput.* **2022**, *18*, 3174-3189.
[27] V. M. Dixit, F. M. Lascovics, W. I. Noal, C. D. Poulter, *J. Org. Chem.* **1981**, *46*, 1967-1969.
[28] H. Li, J. S. Dickschat, *Angew. Chem. Int. Ed.* **2022**, *61*, e202211054; *Angew. Chem.* **2022**, *134*, e202211054.
[29] T. Lou, A. Li, H. Xu, J. Pan, B. Xing, R. Wu, J. S. Dickschat, D. Yang, M. Ma, *J. Am. Chem. Soc.* **2023**, *145*, 8474-8485.
[30] J. Rinkel, P. Rabe, X. Chen, T. G. Köllner, F. Chen, J. S. Dickschat, *Chem. Eur. J.* **2017**, *23*, 10501-10505.
[31] J. Rinkel, J. S. Dickschat, *Org. Lett.* **2019**, *21*, 2426-2429.
